# Biophysical and biological properties of splice-switching oligonucleotides and click conjugates containing LNA-phosphothiotriester linkages

**DOI:** 10.1093/nar/gkaf1263

**Published:** 2025-11-24

**Authors:** Debashis Dhara, Alyssa C Hill, Abinaya Ramesh, Diallo Traore, Ewa Radzikowska-Cieciura, Matthew J A Wood, Tom Brown

**Affiliations:** Department of Chemistry, University of Oxford, Chemistry Research Laboratory, 12 Mansfield Road, Oxford OX1 3TA, United Kingdom; Department of Paediatrics, Institute of Developmental and Regenerative Medicine (IDRM), University of Oxford, Oxford OX3 7TY, United Kingdom; Department of Paediatrics, Institute of Developmental and Regenerative Medicine (IDRM), University of Oxford, Oxford OX3 7TY, United Kingdom; Department of Chemistry, University of Oxford, Chemistry Research Laboratory, 12 Mansfield Road, Oxford OX1 3TA, United Kingdom; Department of Chemistry, University of Oxford, Chemistry Research Laboratory, 12 Mansfield Road, Oxford OX1 3TA, United Kingdom; Division of Bioorganic Chemistry, Centre of Molecular and Macromolecular Studies, Polish Academy of Sciences, Sienkiewicza 112, Lodz 90-363, Poland; Department of Paediatrics, Institute of Developmental and Regenerative Medicine (IDRM), University of Oxford, Oxford OX3 7TY, United Kingdom; Department of Chemistry, University of Oxford, Chemistry Research Laboratory, 12 Mansfield Road, Oxford OX1 3TA, United Kingdom

## Abstract

Antisense oligonucleotides hold great promise in the treatment of disease, but their efficacy is limited by modest bioavailability and toxicity. Charge-neutral phosphorus-based backbones can potentially improve biological properties, but oligonucleotides with such modifications are challenging to synthesize. Here, we report the straightforward synthesis of a range of oligonucleotides containing multiple LNA alkyl phosphothiotriester nucleotides and evaluate their biophysical and biological properties. Several functional groups were incorporated into the triester linkages, including 2-butyl, 2-hexyl, 3-octyl, 4-trifluoromethyl cyclohex-1-yl, hexadecyl, and 4-pentyn-2-yl. The alkyne triesters were functionalized with carbohydrates, amino acids, heptaethylene glycol, spermine, and thiazole orange through CuAAC click chemistry. Analysis of over 60 oligonucleotides showed that almost all displayed excellent duplex stability with both complementary DNA and RNA and good splice-switching activity in an *in vitro* reporter assay. Amino acid conjugates showed significantly higher activity than carbohydrate conjugates via gymnosis.

## Graphical Abstract

**Graphical Abstract:**
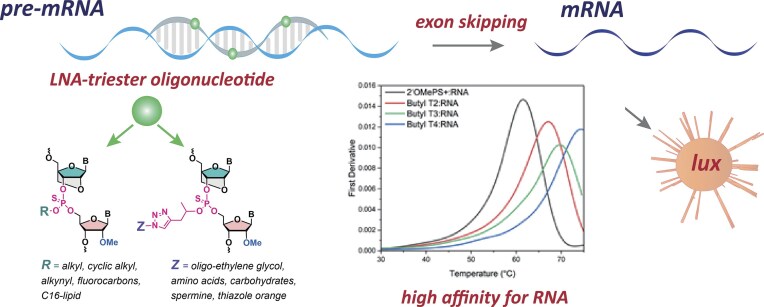

## Introduction

Chemically modified single- and double-stranded oligonucleotides, including antisense oligonucleotides (ASOs), splice-switching oligonucleotides (SSOs), and small interfering RNAs (siRNAs), are revolutionary medicines for treating rare genetic disorders, in particular spinal muscular atrophy (SMA) [1, 2] and Duchenne muscular dystrophy (DMD) [3], as well as more common conditions such as hypercholesterolaemia [4]. Around 20 oligonucleotides are currently in the clinic [5]. They alter gene expression via interactions with RNA [6–8] by modulating pre-messenger RNA (pre-mRNA) splicing [9] or promoting pre-mRNA or mature mRNA degradation [10, 11]. Unmodified oligonucleotides are rapidly digested by nucleases in the biological milieu and must be chemically modified for *in vivo* applications [12]. A range of ribose sugar modifications, including 2′-*O*-methyl (OMe [13]), 2′-*O*-(2-methoxyethyl) (MOE [14]), locked nucleic acid (LNA [15]), constrained ethyl (cEt [16]), tricyclo [17], 2′-fluoro (2′-F [18]), and 2′-*O*-[2-(methylamino)-2-oxoethyl] (NMA) [19], have been developed to improve the chemical and enzymatic stability of oligonucleotides and enhance therapeutic index, but fewer phosphodiester backbone modifications have gained traction [20]. In particular, the phosphorothioate (PS) modification has made a huge contribution to the therapeutic oligonucleotide field [21], with most approved oligonucleotide drugs featuring this linkage [5]. The various interactions of PS-oligonucleotides with proteins have been studied extensively [22–24]. They are beneficial, improving cellular uptake, but can also be detrimental, leading to toxicity [25]. In the latter case reducing the PS content of ASOs has been shown to improve their toxicological properties [26]. The PS modification greatly increases the stability of oligonucleotides to digestion by nuclease enzymes but also reduces RNA target-binding affinity.

**Figure 1.**
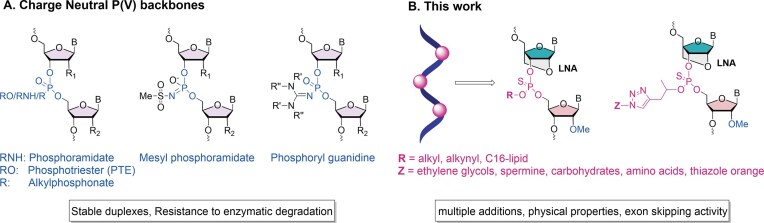
(**A**) Phosphorus-modified oligonucleotide backbones. (**B**) Current work on LNA PTTE oligonucleotides and their conjugates.

Distinct from oligonucleotides bearing a charged PS backbone are SSOs of the phosphorodiamidate morpholino (PMO) chemical class, which have a charge-neutral backbone. Eteplirsen, golodirsen, viltolarsen, and casimersen are all approved PMOs designed to promote exon skipping for the treatment of DMD [5, 27], a genetic disease affecting 1 in 5000 boys globally. These drugs act by binding dystrophin pre-mRNA, forcing the skipping of exons 51, 53, or 45, and restoring the open reading frame to produce a shorter, but functional, dystrophin protein. Other charge-neutral backbones have shown promise in preclinical studies. Peptide nucleic acid (PNA), first described by Nielsen and colleagues, consists of nucleobases linked by an N(2-aminoethyl) glycine backbone [28]. The lack of negative charges in PNAs improves binding to complementary DNA and RNA and provides resistance to nuclease digestion. PNA has applications in molecular diagnostics [29] but has not yet entered the therapeutic field. Notably, access to both PMOs and PNAs is limited owing to their specialized synthetic routes. Artificial charge-neutral backbones, such as amide [30–33], amine [34], sulfamate [35], and triazole [36], are possible alternatives; however, these backbones are typically introduced during oligonucleotide synthesis as dinucleotide phosphoramidites, so the synthesis of any oligonucleotide sequence requires 16 building blocks. In contrast, the phosphorus (P-based) backbones alkyl phosphonate, phosphotriester, phosphoramidate, mesyl and alkyl sulfonyl phosphoramidate [37, 38], and phosphoryl guanidine [39] are synthesized using conventional nucleoside phosphoramidite monomers (Fig. 1). However, synthesizing P-based charge-neutral backbone oligonucleotides is not straightforward, and to address this, we recently developed a simple method for synthesizing LNA-alkyl phosphothiotriester (PTTE) and LNA-phosphotriester (PTE) oligonucleotides [40]. Importantly, these backbones offer the possibility of flexible functionalization with various ligands and lowering of PS content without compromising stability to nucleases or duplex stability.

Achieving efficient biodistribution, cellular uptake, and intracellular trafficking remain major barriers to the clinical translation of oligonucleotides. PS-modified oligonucleotides can enter cells in the absence of transfection reagents by gymnosis [41], a phenomenon that is incompletely understood [22]. Conjugate groups that enable receptor-mediated endocytosis can enhance the delivery of oligonucleotides into cells. For instance, tris-*N*-acetylgalactosamine (GalNAc)_3_ conjugates of oligonucleotides efficiently enter hepatocytes *via* the asialoglycoprotein receptor (ASGPR), which is both highly expressed and rapidly recycled [42–44]. Other receptors may in future prove to be prime candidates for enhancing oligonucleotide delivery. For example, glucose receptors are expressed across most cell types, but there is limited evidence supporting the efficient cellular delivery of glucose-conjugated oligonucleotides [45]. Amino acid transporters facilitate the influx of amino acids in exchange for intracellular substrates and are often overexpressed in cancer cell lines [46]. However, it is yet to be demonstrated that amino acid-conjugated oligonucleotides improve cellular delivery. On the other hand, lipid-conjugated oligonucleotides are relatively well studied, and some are known to improve oligonucleotide biodistribution, cellular uptake, and activity [47, 48].

**Scheme 1.**
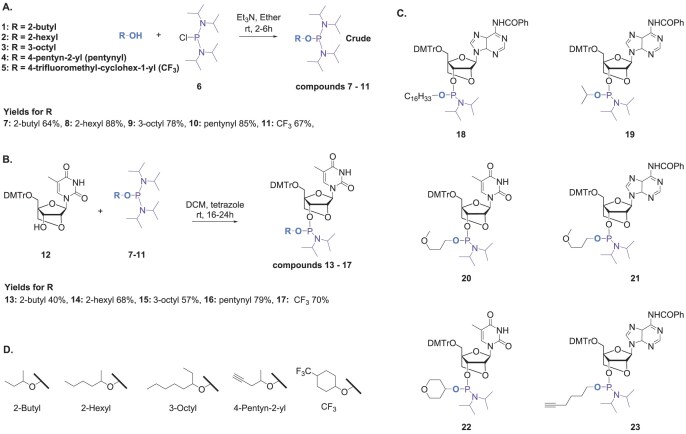
(**A**) Chemical synthesis of P(III) reagents 7–11. (**B**) Synthesis of P(III) nucleoside phosphoramidite monomers 13–17. (**C**) Monomers 18–23 were prepared as previously reported [40]. (**D**) New alkyl groups introduced in this study into LNA-T triester phosphoramidites. Alcohols 1–4 are mixtures of (R/S)-isomers.

The rapidly developing therapeutic oligonucleotide field has an urgent need for highly functional oligonucleotides based on simple, scalable, and flexible chemistry. Until now, the conjugation of carbohydrates, peptides/proteins, lipids, and other ligands has been mainly limited to the 5′- and 3′-positions of the oligonucleotide, and our objective was to broaden this to include single and multiple internal sites. Here, we report the synthesis of SSOs with a wide variety of charge-neutral alkyl and alkynyl PTTE backbones attached to duplex-stabilizing LNA sugars. The methodology is fully compatible with standard solid-phase synthesis and deprotection. These novel oligonucleotides have various alkyl groups in the PTTE internucleoside bridge, including 2-butyl, 2-hexyl, 3-octyl, 4-trifluoromethylcyclohex-1-yl (CF_3_), methoxypropyl (MOP), isopropyl (iPr), 4-pentyn-2-yl, C16 lipid (hexadecyl), and alkynyl (Scheme 1, Table 1). The alkynyl-PTTE oligonucleotides were conjugated to carbohydrates (glucose, galactose, and lactose), amino acids (lysine, leucine, phenylalanine, and valine), heptaethylene glycol, a groove binder (spermine), and an intercalator (thiazole orange; TO). The oligonucleotides were analysed by UV melting and circular dichroism, and their splice-switching activities were measured in the reporter HeLa pLuc/705 cell line.

**Table 1.** Oligonucleotides used in this study, duplex melting data, and splice-switching activity data

Oligonucleotide	Sequence (5′→3′)	SpSw	ΔTm RNA	Oligonucleotide	Sequence (5′→3′)	SpSw	ΔTm RNA
Alkyl phosphothiotriesters				Alkynyl and clicked phosphothiotriesters			
Butyl T2	CCUCUtACCUCAGUtACA	1.5	5.7	Hexyn A1	CCUCUUACCUCaGUUACA	1.3	2.4*
Butyl T3	CCUCUtACCtCAGUtACA	0.9	8.5	Hexyn A2	CCUCUUaCCUCaGUUACA	2.1	3.0*
Butyl T4	CCtCUtACCtCAGUtACA	1.3	13.0	Pentyn T1	CCUCUUACCUCAGUtACA	1.7	2.1
C16 A1	CCUCUUACCUCaGUUACA	0.2	−0.3*	Pentyn T2	CCUCUtACCUCAGUtACA	2.2	4.6
C16 A2	CCUCUUaCCUCaGUUACA	0.2	−5.2*	Pentyn T3	CCUCUtACCtCAGUtACA	1.7	6.3
C16 A2b	CCUCUUaCCUCAGUUaCA	0.2	−5.7*	Glycol T1	CCUCUUACCUCAGUtACA	1.6	1.6
C16 A3	CCUCUUaCCUCaGUUaCA	0.3	−14.1*	Glycol T2	CCUCUtACCUCAGUtACA	0.4	4.0
CF_3_ T1	CCUCUUACCUCAGUtACA	2.3	1.4	Glycol T3	CCUCUtACCtCAGUtACA	0.5	4.7
CF_3_ T2	CCUCUtACCUCAGUtACA	1.1	3.5	Gluc A1	CCUCUUACCUCaGUUACA	0.6	2.2
CF_3_ T3	CCUCUtACCtCAGUtACA	0.3	5.5	Gluc A2	CCUCUUaCCUCaGUUACA	1.1	3.2
CF_3_ T4	CCtCUtACCtCAGUtACA	0.4	6.2	Gluc T3	CCUCUtACCtCAGUtACA	0.4	7.3
Hexyl T2	CCUCUtACCUCAGUtACA	0.7	3.4	Gal T3	CCUCUtACCtCAGUtACA	0.6	7.5
Hexyl T3	CCUCUtACCtCAGUtACA	0.4	6.4	Lac T3	CCUCUtACCtCAGUtACA	0.3	6.9
Hexyl T4	CCtCUtACCtCAGUtACA	0.2	10.3	Lys T3	CCUCUtACCtCAGUtACA	1.5	7.2
iPr A1	CCUCUUACCUCaGUUACA	1.5	3.6*	Leu T3	CCUCUtACCtCAGUtACA	1.7	4.7
iPr A2	CCUCUUaCCUCaGUUACA	1.7	5.2*	Phe T3	CCUCUtACCtCAGUtACA	1.9	5.6
iPr A2b	CCUCUUaCCUCAGUUaCA	1.3	4.5*	Val T3	CCUCUtACCtCAGUtACA	1.2	5.0
iPr A3	CCUCUUaCCUCaGUUaCA	2.2	7.0*	Sper T1	CCUCUUACCUCAGUtACA	1.2	3.2
MOP A1	CCUCUUACCUCaGUUACA	1.5	2.8*	Sper T2	CCUCUtACCUCAGUtACA	Nd	13.3
MOP A2	CCUCUUaCCUCaGUUACA	2.3	3.5*	Sper T3	CCUCUtACCtCAGUtACA	Nd	Nd
MOP A2b	CCUCUUaCCUCAGUUaCA	1.5	2.8*	TO A1	CCUCUUACCUCaGUUACA	0.8	3.5
MOP A3	CCUCUUaCCUCaGUUaCA	2.3	4.9*	TO A2	CCUCUUaCCUCaGUUACA	Nd	2.9
MOP T1	CCUCUUACCUCAGUtACA	0.9	3.0*	TO T1	CCUCUUACCUCAGUtACA	0.9	2.5
MOP T2	CCUCUtACCUCAGUtACA	1.1	5.5*	TO T2	CCUCUtACCUCAGUtACA	0.3	−0.6
MOP T3	CCUCUtACCtCAGUtACA	2.2	6.5	LNA phosphothiodiesters			
MOP T4	CCtCUtACCtCAGUtACA	1.2	9.4*	LNA A1	CCUCUUACCUCaGUUACA	1.6	4.4*
MOP T6	CCtCttACCtCAGttACA	1.0	17.0*	LNA A2	CCUCUUaCCUCaGUUACA	2.5	6.5*
Octyl T2	CCUCUtACCUCAGUtACA	0.6	4.2	LNA A3	CCUCUUaCCUCaGUUaCA	2.3	8.9*
Octyl T3	CCUCUtACCtCAGUtACA	0.2	7.9	LNA T1	CCUCUUACCUCAGUtACA	2.3	5.3*
Octyl T4	CCtCUtACCtCAGUtACA	0.3	5.8	LNA T2	CCUCUtACCUCAGUtACA	4.9	9.1*
THP T4	CCtCUtACCtCAGUtACA	1.8	11.4*	LNA T3	CCUCUtACCtCAGUtACA	1.9	11.3
THP T6	CCtCttACCtCAGttACA	0.8*	18.1*	LNA T4	CCtCUtACCtCAGUtACA	2.2	17.2*
THP T6 15	tCttACCtCAGttAC	0.7	7.7	LNA T6	CCtCttACCtCAGttACA	1.0	22.7*
2′OMePS+	CCUCUUACCUCAGUUACA			Complementary DNA	TGTAACTGAGGTAAGAGG		
2′OMePS−	CCUCAUUCACUCGAUUCA			Complementary RNA	UGUAACUGAGGUAAGAGG		

Nucleotides in upper case (A, C, G, U) have 2′-*O*-methyl ribose sugars and PS linkages (2′-OMe PS). Nucleotides in lowercase (a, t) have LNA sugars and PS-triester or PS-diester linkages, as indicated in the relevant column headings. ΔTm = difference in duplex melting temperature against RNA compared to the 2′-OMe PS positive control (2′OMePS+). Tm of control versus RNA = 61.3°C. Melting temperatures were recorded in 10 mM Na-phosphate buffer, pH = 7.0, containing 25 mM NaCl. Tm values are an average of three experiments with an error of ±0.50°C. SpSw = Fold increase in splice-switching activity relative to 2′OMePS+ under gymnosis conditions at 20 µM. *Indicates SpSw or UV melting data taken from the literature [40] and Nd, not determined.

## Materials and methods

Our synthetic approach to assembling phosphotriester oligonucleotides [40] is essentially identical to conventional solid-phase phosphoramidite synthesis [49]. It simply requires the preparation of nucleoside 3′-phosphoramidites in which the chosen alkyl group replaces the 2-cyanoethyl group of standard nucleoside phosphoramidites. To prepare the required monomers, alcohols 1–5 were reacted with bis(diisopropylamino)chlorophosphine 6 to give the P(III) reagents 7–11 in 64%–88% yield (Scheme 1, SI 1.0). Next, commercially available 5′-O-DMTr-protected locked thymidine nucleoside 12(15) was reacted with P(III) reagents 7–11 in the presence of tetrazole to afford the thymidine LNA phosphoramidites 13–17. Racemic alcohols 1–4 were used for the synthesis of compounds 13–16, and monomers 18–23 were prepared as previously reported [40]. All the P(III) reagents and phosphoramidite monomers were characterized by nuclear magnetic resonance spectroscopy (NMR) and high-resolution mass spectrometry (HRMS). Their synthesis is robust and high yielding, and the monomers can be produced on a large scale without any difficulties related to reaction conditions or purification. However, the preparation of P(III) monomers and phosphoramidite reagents that have amino or alkylamino functionalities has additional challenges (Supplementary Scheme S1, SI 1.3), and our laboratory is currently optimizing their synthesis (Supplementary Scheme S1).

Phosphoramidite monomers 13–23 were used along with 2′-*O*-methyl derivatives of *N*^6^-benzoyl-A, *N*^2^-isobutyryl-G, *N*^4^-acetyl-C, and U (Supplementary Fig. S1) for the synthesis of oligonucleotides on the 1 µmole scale (Table 1) using EDITH (3-ethoxy-1,2,4-dithiazole-5-one) as a sulfurizing reagent. Coupling efficiencies of the monomers 13–23 were measured by the liberated DMT^+^ cations during solid-phase synthesis, all of which were near-quantitative. All oligonucleotides were cleaved from the solid support and deprotected with a 1:1 mixture of THF-ethylene diamine (EDA) for 2 h at RT, purified by reversed-phase HPLC, and analysed by UPLC-MS (SI 2.0, SI Table T1). Yields are in SI Table T2. NMR spectra of all compounds are in Supplementary Figs S159–188.

The UV-melting experiments were performed using a Cary 4000 UV-Vis spectrophotometer. Each oligonucleotide (2 nmoles) was dissolved in 1 ml of 10 mM phosphate buffer with 100 mM NaCl (for oligonucleotides paired with a DNA target) or 25 mM NaCl (for those paired with an RNA target) at pH 7.0. The samples were initially denatured at 85°C then annealed by slowly cooling. Six successive cycles of heating and cooling were then performed at 1°C/min while recording UV absorbance at 260 nm. Melting temperatures were calculated from the first derivatives of the melting curves.

## Results

### Oligonucleotide synthesis

Alkyl LNA PTTE oligonucleotides with varying numbers of 2-butyl, 2-hexyl, 3-octyl, CF_3_, MOP, iPr, and C16-lipid triesters were prepared (Table 1, Scheme 1D). These groups were chosen to determine if non-covalent interactions with the cell membrane can influence gymnotic uptake and splice-switching activity. The trifluoromethyl-cyclohexyl group was selected for the possibility that fluorophilic interactions might improve cellular uptake. Fluorine is also a sensitive NMR nucleus that can be used to monitor cell trafficking with applications in the protein field [50].

**Figure 2.**
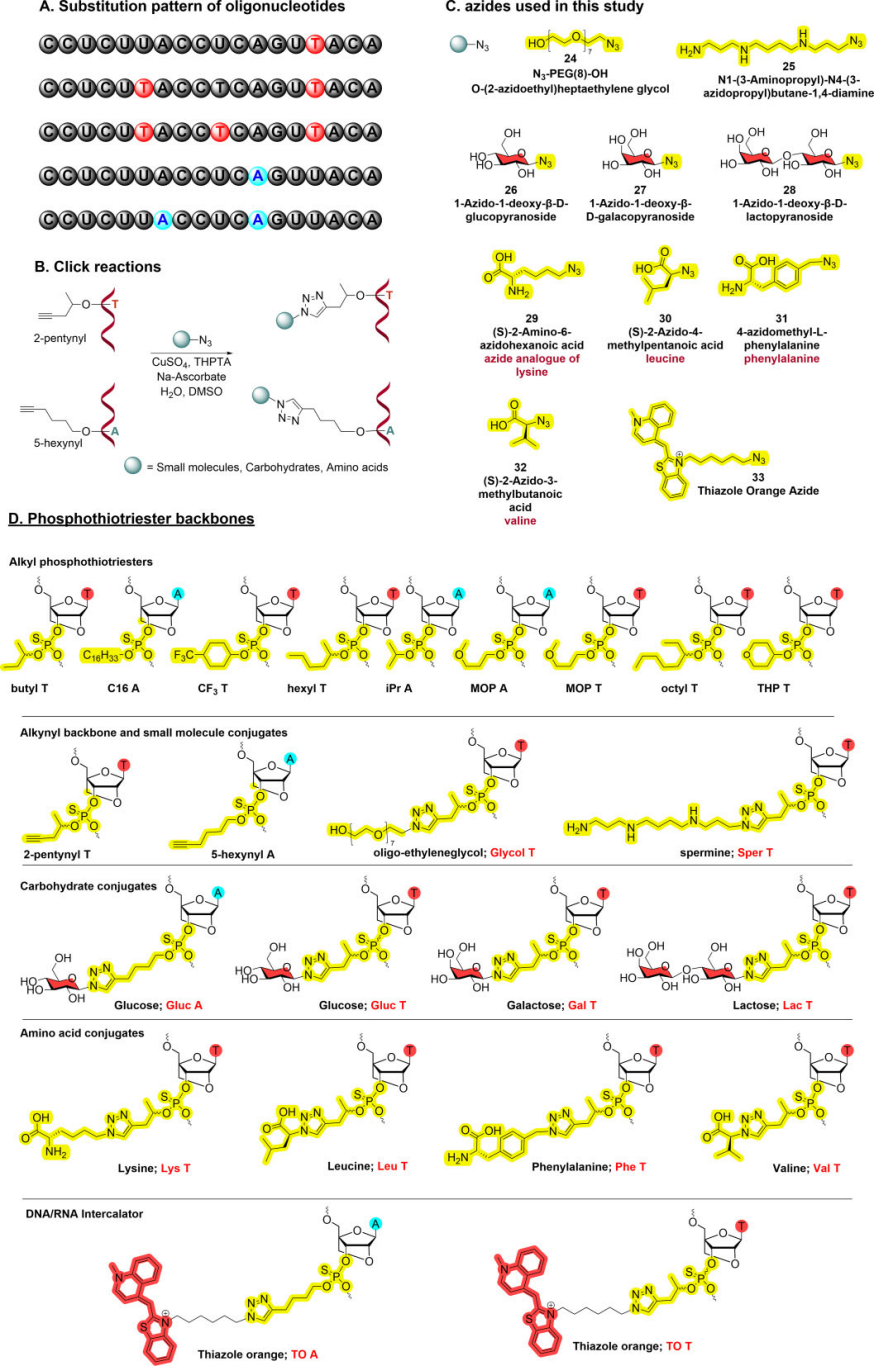
(**A**) Substitution patterns of alkynyl oligonucleotides used in the click reactions. (**B**) CuAAC labelling of 5-hexynyl and 2-pentynyl 18-mer 2′-OMePS oligonucleotides. (**C**) Azides used for click reactions. (**D**) Phosphothiotriester backbone structures.

Oligonucleotides were prepared with one to three 4-pentynyl and one or two 5-hexynyl LNA-T-PTTE linkages. As 5-hexynol is a primary alcohol, it is partly cleaved from the triester during oligonucleotide deprotection; thus, purifying oligonucleotides with multiple hexynyl groups requires care [40]. We used commercially available phosphoramidite monomers in oligonucleotide synthesis and were therefore constrained by their nucleobase protecting groups. However, it should be possible in future to obtain LNA-triester oligonucleotides based on primary alcohols in higher yield if more labile (i.e. ultramild) protecting groups are used [51]. In contrast, oligonucleotides with multiple pentynyl LNA-T PTTE bridges are stable during oligonucleotide deprotection and were conjugated to several small biomolecules *via* copper(I)-catalyzed azide alkyne cycloaddition (CuAAC) (Fig. 2A and B and Supplementary Fig. S2). A broad range of azides were chosen, including O-(2-azidoethyl)heptaethylene glycol 24 and spermine 25 (Fig. 2C). Glycol azide 24 was chosen because it is hydrophilic, and because oligonucleotides with terminal polyethylene glycol have shown increased nuclease resistance [52]. However, the effects of oligo-ethylene glycol on the triester backbone are not known. Pentyn T1, T2, and T3 oligonucleotides were converted to Glycol T1, T2, and T3 derivatives (Table 1) by reaction with azide 24. Spermine-conjugated oligonucleotides stabilize duplexes by minor groove binding and reducing electrostatic repulsion and can improve cellular uptake [53, 54]. Sper T1 and Sper T2 were readily obtained from Pentyn T1 and Pentyn T2 through CuAAC with spermine azide 25.

Monosaccharide- and disaccharide-conjugated oligonucleotides have shown improved biological activity and nuclease stability [55–57], and sugar conjugation has been proposed to enhance the uptake of oligonucleotides by specific cellular transporters. Gluc T3, Gal T3, and Lac T3 oligonucleotides were synthesized from Pentyn T3 *via* CuAAC reaction with glucose azide 26, galactose azide 27, and lactose azide 28. Pentyn T3 was also used to synthesize Lys T3, Leu T3, Phe T3, and Val T3 by reacting with amino acid-derived azides 29–32 (Fig. 2 and Table 1). Pentyn T1, Pentyn T2, Hexyn A1, and Hexyn A2 oligonucleotides were labelled with thiazole orange azide 33 to give TO T1, TO T2, TO A1, and TO A2, respectively. TO is a DNA/RNA intercalator, so it can potentially enhance duplex stability and might improve splice-switching activity when attached to a triester backbone [58].

The CuAAC click reaction [59] has achieved widespread adoption in the oligonucleotide field [60–65] because of its very high efficiency and chemoselectivity. Hence, we adopted this chemistry to functionalize the alkynyl triester oligonucleotides. The click reactions were performed on the 20–150 nmole scale, and high yields of the desired products were generally obtained (≥88%, SI Table T3). However, lower yields were observed with some azides. Multiple additions of heptaethylene glycol gave yields slightly above 60% and TO azide gave yields from 45% to 71%. The greatest difficulties were encountered with multiple additions of spermine azide. Two additions gave 42% yield, and we were unable to obtain a pure oligonucleotide with three spermines. We are currently exploring alternative ‘click’ strategies for DNA-binding azides such as spermine and TO. Importantly, in most cases the backbone click reaction is robust and should prove to be compatible with micromole to millimole or even higher scales using a catalytic amount of Cu(II) sulphate under standard click reaction conditions. To remove the copper ion contaminants, the crude products were treated with 0.1 M EDTA and then purified by reversed-phase HPLC. All oligonucleotides were characterized by UPLC MS (SI 2.2–2.5, SI Figs S3–S112). The structures of all nucleotides incorporated into oligonucleotides are in Fig. 2D.

### Duplex stability

Melting temperatures (Tms) of the modified oligonucleotides hybridized to complementary DNA and RNA were measured to assess the impact of the modifications in the PTTE backbone on duplex stability (Tm data versus RNA Table 1, versus DNA and RNA SI 3.0–3.11, Supplementary Figs S113–S134, SI Tables T4–T13). Except for C16-lipid and TO T2, all the investigated modifications were stabilizing against both DNA and RNA compared to the 2′OMePS+control (ΔTm of +1.5 to +15.6°C for DNA and +1.4 to +17.0°C for RNA). However, the duplexes were less stable than those formed by the corresponding LNA-phosphorothioate control oligonucleotide without triester linkages. For example, fully phosphodiester LNA T4 has a ΔTm of +17.2°C, whereas the most stabilizing equivalent alkyl triester butyl T4 has a ΔTm of +13.0°C. Thus, the stabilizing effect of the LNA sugar on phosphothiotriester linkages is positive, but it is smaller than that of the anionic phosphorothioate group. Multiple additions of C16 are strongly destabilizing, and the lack of stabilization by TO is possibly due to the duplex conformation being incompatible with efficient intercalation of triester-linked TO. Distinct trends were observed based on the type of modification and number of insertions. 2-Butyl-modified oligonucleotides exhibited exceptional duplex stability, particularly with four additions, which gave the highest Tm for both DNA (61.6°C) and RNA (74.3°C). 2-Hexyl-modified oligonucleotides also provided good duplex stability, again with four additions having the highest Tm value (57.0°C for the DNA target; 71.6°C for the RNA target). The 3-octyl group gave slightly lower Tm values, indicating that duplex stability decreases with the increasing lipophilic character and/or steric bulk of the alkyl groups attached to the backbone. Conforming with this trend, pentynyl modifications produced intermediate stabilizing effects. Single glycol and groove-binding spermine conjugates produced minor stabilization, whereas the attachment of two spermines (Sper T2) gave a large increase in duplex stability against both DNA (+10.5°C) and RNA (+13.3°C).

Carbohydrate-conjugated oligonucleotides all provided duplex stabilization with complementary DNA and RNA. Amino acid conjugates, except for the lysine analogue, gave a slight increase in duplex stability relative to the 2′OMePS+control, but a decrease relative to Pentyn T3. In general, the LNA sugars ensured an increased Tm relative to the 2′OMePS+ control proportional to the number of PTTE linkages. Overall, the study confirms that multiple LNA-PTTE functionalities can be introduced into the oligonucleotide backbone without compromising affinity for the intended RNA target.

### Duplex conformation

**Figure 3.**
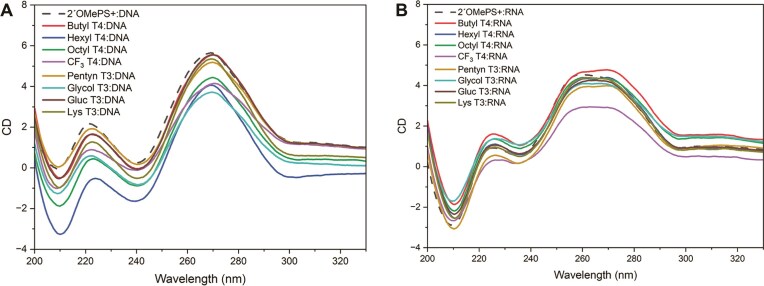
CD spectra of modified oligonucleotide:DNA duplexes (**A**) and oligonucleotide:RNA duplexes (**B**) aligned with 2′OMePS+:DNA and 2′OMePS+:RNA reference duplexes. The *y*-axis is ellipticity θ (10^−3^ deg·cm^2^/dmol).

Previously we found that LNA alkyl PTTE backbones cause minimal perturbation to the duplex structure, even when oligonucleotides are modified with >50% of these charge-neutral bridges. In this study, the CD spectra of modified oligonucleotide:DNA duplexes were compared to the 2′OMePS+ oligonucleotide:DNA duplex, which adopts a typical B-form DNA helical structure (Fig. 3A). A slight distortion was observed for oligonucleotides with four LNA hexyl and octyl triester bridges, suggesting a small change in duplex conformation/helicity related to hydrophobicity. The CD spectra of modified oligonucleotides hybridized to complementary RNA are almost perfectly aligned with the control:RNA duplex, which has an A-form RNA helical structure (Fig. 3B), except for the fluorinated triester, which displayed decreased ellipticity between 260 nm and 280 nm. Individual spectra are in SI 4.0, Supplementary Figs S135–S156 and SI Tables T14–T24.

### Fluorescence properties

**Figure 4.**
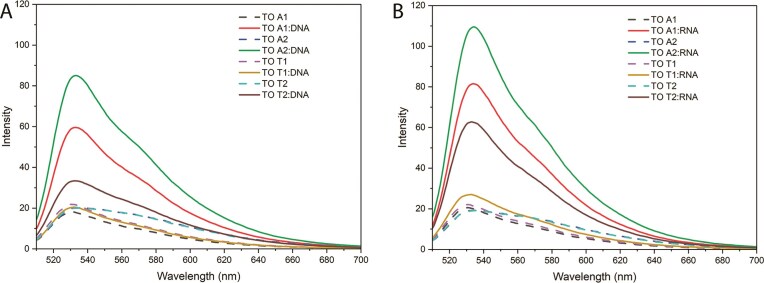
Fluorescence emission spectra of TO-labelled oligonucleotides. TO-labelled oligonucleotides paired with complementary DNA (**A**) or RNA (**B**). The *x*-axis represents the emission wavelength (520–700 nm), and the *y*-axis shows fluorescence intensity (fluorescence units).

Fluorescence spectra (Fig. 4) show that single-stranded TO PTTE derivatives (TO A1, TO A2, TO T1, and TO T2) exhibit very low fluorescence. Upon the addition of complementary DNA or RNA, large enhancements in fluorescence were observed, which is typical for TO dyes bound to nucleic acid duplexes, due to restricted intramolecular rotation of the dye caused by groove binding or intercalation [66]. The TO A2 derivative showed the greatest fluorescence enhancement upon binding to DNA and RNA, whereas TO A1 gave a lower fluorescence enhancement. The TO T2 derivatives displayed moderate fluorescence enhancements, while TO T1 exhibited the weakest fluorescence response, suggesting limited interaction of TO with the duplex or less restriction of rotation of the fluorophore. Overall, these results demonstrate that the structural differences among TO-PTTE derivatives influence fluorescence properties. The variation in fluorescent intensity of the four different TO derivatives indicates that DNA binding depends on several factors, such as the attached nucleoside (A or T), the surrounding base pairs, and the nature of the linker (pentynyl versus hexynyl).

### Biological studies

**Figure 5.**
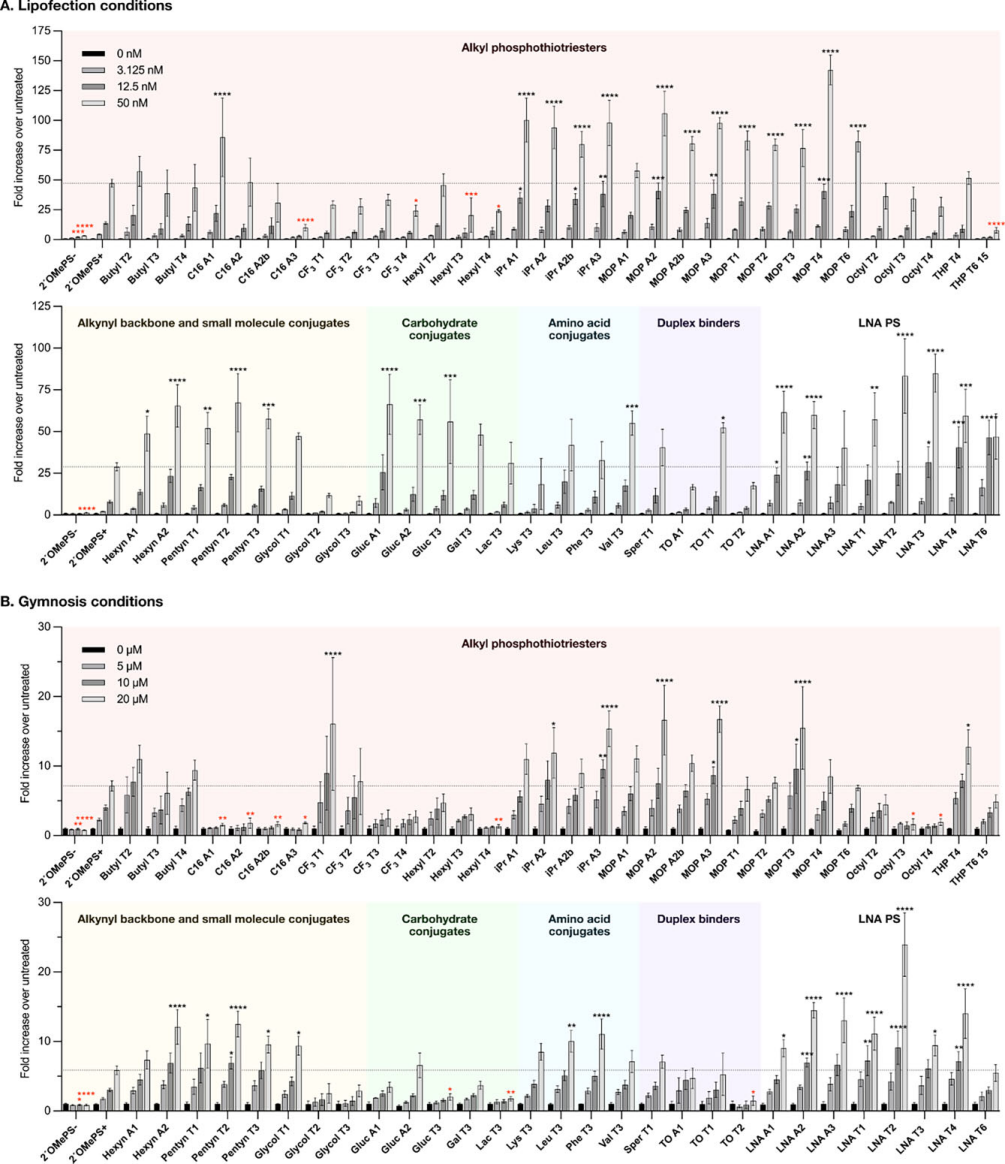
Splice-switching activities of oligonucleotides in HeLa pLuc/705 cells. (**A**) Activities of oligonucleotides under lipofection conditions. Oligonucleotides were transfected into cells using Lipofectamine 2000 and luminescence was measured 48 h later. (**B**) Activities of oligonucleotides under gymnosis conditions. Oligonucleotides were applied to cells in the absence of a transfection reagent and luminescence was measured 72 h later. Data are means ± SEM for three to 37 biological replicates (*n* = 3–37), except for MOP T6 under gymnosis conditions, where two biological replicates (*n* = 2) are plotted. In each plot, the dotted line indicates the mean activity level of 2′OMePS+ at the highest treatment concentration (i.e. 50 nM or 20 µM). Statistics are ordinary two-way analysis of variance (ANOVA) with Dunnett’s multiple comparisons test against 2′OMePS+: α = 0.05: * *P* ≤ .05, ** *P* ≤ .01, *** *P* ≤ .001, and **** *P* ≤ .0001. Red asterisks highlight compounds with lower activity than 2′OMePS+ at the same concentration.

As introduced earlier, SSOs bind pre-mRNA and modulate splicing, e.g. by forcing the skipping [67–69] or inclusion [2, 70] of exons. The oligonucleotide sequence used in this study is an SSO sequence designed for use in the HeLa pLuc/705 cell line, which expresses a luciferase pre-mRNA that has been intentionally interrupted by a mutated human beta-globin intron 2. This mutation activates alternative splice sites in the pre-mRNA; therefore, in the absence of the SSO, the luciferase pre-mRNA is aberrantly spliced, and luciferase protein is not produced. However, when the SSO is present, it binds the pre-mRNA at the mutation site and prevents aberrant splicing. As a result, luciferase protein is produced, and its activity can be measured [71, 72]. We investigated the ability of over 60 modified oligonucleotides to induce splice correction in HeLa puc/705 cells under both lipofection conditions (Fig. 5A) and gymnosis conditions (Fig. 5B). Remarkably, the majority of modifications did not negatively impact splice-switching activity relative to 2′OMePS+, and many modifications improved activity.

#### Lipofection

Under lipofection conditions, relative to the 2′OMePS+ control, the alkyl PTTE MOP modification improved activity when installed at two to three A nucleotides or one to six T nucleotides, and the alkyl PTTE iPr modification also improved activity relative to the 2′OMePS+ control when installed at one to three A nucleotides (Fig. 5A). Interestingly, the C16 (hexadecyl) modification improved activity when installed at a single A nucleotide; this improvement was lost with two C16s, and activity was further impaired with three C16s. These results align with the RNA target-binding affinities of the modified oligonucleotides. While one C16 insertion has a negligible effect on Tm (−0.3°C, i.e. within error), two to three insertions are destabilizing (ΔTms v RNA of −5.2, −5.7, and −14.1°C for C16 A2, C16 A2b, and C16 A3, respectively). Increased activity was observed for the alkynyl backbone pentyn and hexyn modifications, carbohydrate conjugates of glucose (Gluc), the amino acid conjugate of valine (Val), and the DNA/RNA intercalator TO T1. Several of the alkyl-PTTE oligonucleotides were more active than the best-performing LNA all-PS control oligonucleotides, LNA T2 and LNA T3.

#### Gymnosis

Under gymnosis conditions, fewer constructs improved on the activity of 2′OMePS+ (Fig. 5B). Butyl T2, butyl T3, butyl T4; hexyl T2, hexyl T3; and octyl T2 did not significantly impact splice-switching activity, although more insertions of bulkier groups trended toward reduced activity. Consistent with this trend, hexyl T4, octyl T3, and octyl T4 all reduced activity. Given that these compounds trigger splice switching under transfection conditions, we hypothesize that they do not efficiently enter cells or traffic to the nuclear compartment. Taken together, the results suggest that fewer insertions of smaller secondary alkyl groups (e.g. two butyl groups) enhance splice-switching activity, while more insertions of larger secondary alkyl groups (e.g. four octyl groups) hinder activity, relative to the 2′OMePS+ control. The alkyl PTTE MOP modification improved activity when installed at two to three A nucleotides (although the effect was dependent on position; see MOP A2 and MOP A2b). Likewise, two to three installations of the iPr modification at A nucleotides improved activity. Interestingly, all investigated C16 derivatives reduced activity; taken together with the corresponding transfection results, these data suggest that, unaided, the C16 derivatives are not efficiently taken up by cells or trafficked to the nuclear compartment. They may also form micelles, which could affect their availability to bind to mRNA.

The CF_3_ modification improved activity when installed at a single T nucleotide; more installations gave lower activity. Modest improvements were observed for the pentyn modification and hexyn derivative, as well as the glycol conjugate at a single T nucleotide. Carbohydrate conjugates and the DNA/RNA intercalators were either benign or showed reduced activity, while amino acid conjugates were either benign or increased activity. By contrast to what was observed under transfection conditions, under gymnosis conditions, none of the alkyl PTTE oligonucleotides were as active as the best-performing LNA all-PS control oligonucleotide, LNA T2. This is also discussed below.

Previously, we showed that up to four PTTE THP modifications enhanced splice-switching activity, while six to nine modifications reduced activity, relative to 2′OMePS+ [40]. We also showed that increases in RNA target-binding affinity positively correlate with splice-switching activity up to ΔTms of approximately +8°C, beyond which point additional gains in Tm are detrimental to splice-switching activity [40]. In an effort to incorporate many PTTE THP modifications but maintain an optimum Tm, we installed six PTTE THP modifications in a 15-mer oligonucleotide to yield THP T6 15. Reducing the length of the oligonucleotide from 18 to 15 nucleotides offset the increase in RNA-binding affinity caused by the modifications, thus bringing the ΔTm down from +18.1°C to +7.7°C (Table 1), which is close to the ΔTm we identified as optimal [40]. Under lipofection conditions, however, THP T6 15 was less active than both 2′OMePS+ and THP T4 (Fig. 5A), and under gymnosis conditions, a similar trend was observed (Fig. 5B). These results indicate, unsurprisingly, that Tm is not a sole determinant of splice-switching activity, even in this limited *in vitro* system.

**Figure 6.**
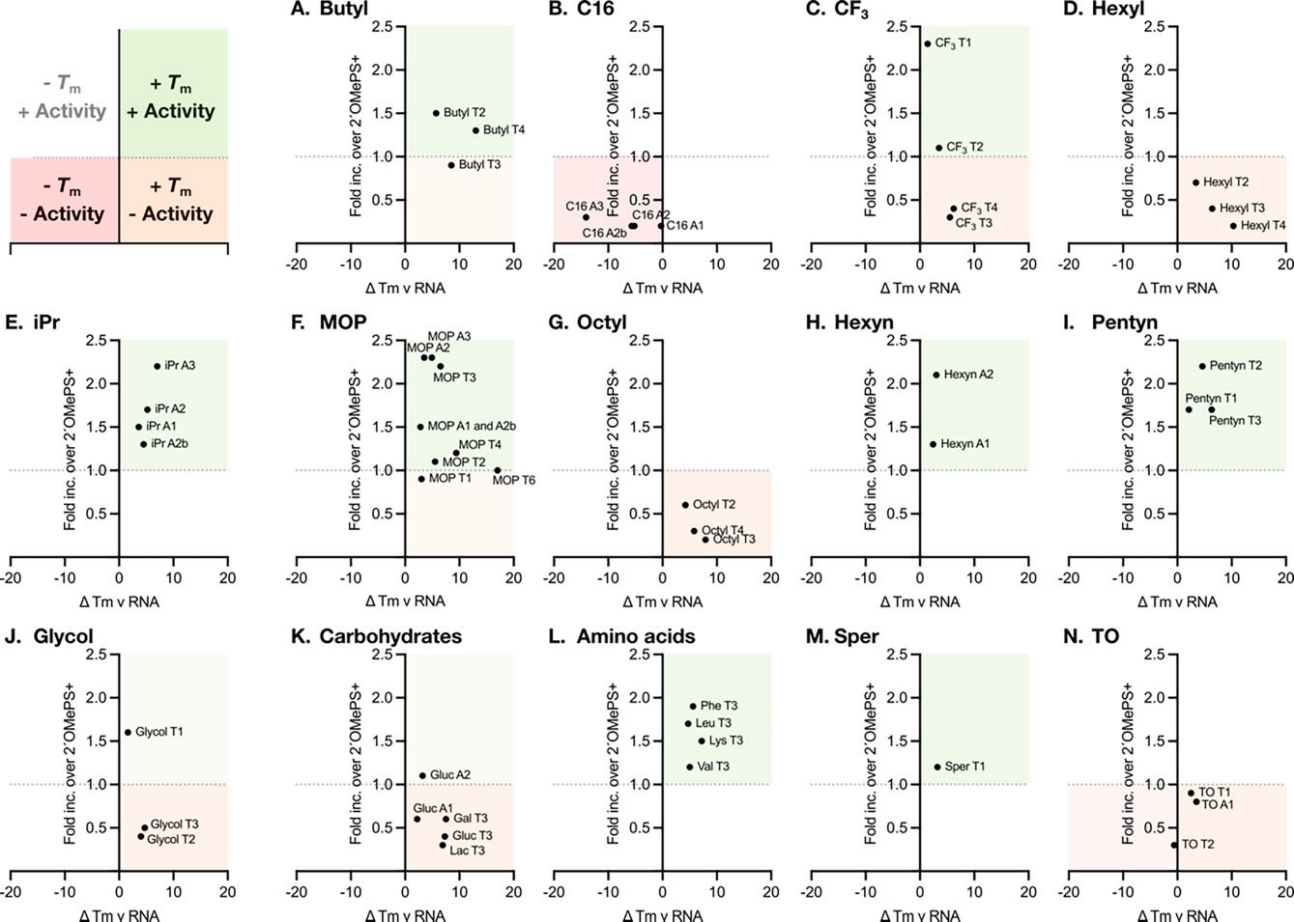
Splice-switching activities of oligonucleotides in HeLa pLuc/705 cells as a function of Tm. Data are mean fold increases over 2′OMePS+ under gymnosis conditions at 20 µM for three to four biological replicates (*n* = 3–4) as a function of ΔTm v RNA (Table 1), except for MOP T6, where the mean of two biological replicates (*n* = 2) is plotted. Oligonucleotides are grouped by class: (**A**) butyl, (**B**) C16, (**C**) CF_3_, (**D**) hexyl, (**E**) iPr, (**F**) MOP, (**G**) octyl, (**H**) hexyn, (**I**) pentyn, (**J**) glycol, (**K**) carbohydrate conjugates, (**L**) amino acid conjugates, (**M**) spermine, and (**N**) thiazole orange (TO) derivatives. In each plot, the dotted line indicates the mean splice-switching activity of 2′OMePS+ under gymnosis conditions at 20 µM for 21 to 23 biological replicates (*n* = 21–23), which was set to 1.0. Green indicates oligonucleotides that display improvements in both Tm and activity, orange indicates oligonucleotides that display improvements in Tm but reductions in activity, and red indicates oligonucleotides that display reductions in both Tm and activity. Reductions in Tm but improvements in activity (white) were not observed.

To understand the relationship between Tm and splice-switching activity, we plotted fold increase in splice switching relative to 2′OMePS+ under gymnosis conditions at 20 µM as a function of ΔTm v RNA (Table 1), and the analysis is presented in Fig. 6. We observed that some modifications improve both Tm and activity (green; MOP, iPr, butyl, pentyn, sper, hexyn, and amino acid conjugates), some modifications improve Tm but not activity (orange; hexyl, octyl, CF_3_, glycol, carbohydrate conjugates, and TO), and one modification reduced both Tm and activity (red; C16). Notably, we did not identify a compound that reduced Tm but improved activity.

## Discussion

We report the simple, robust, and scalable synthesis of oligonucleotides containing a broad range of charge-neutral LNA alkyl PTTE linkages in which the triester side chains were introduced as phosphoramidites or by post-synthesis click chemistry. The presence of the LNA sugar improves their chemical stability and RNA-binding affinity. Although LNA is a potent duplex-stabilizing modification, oligonucleotides containing LNA sugars have not yet entered the clinic, in part due to unfavourable toxicological properties caused by protein binding and off-target effects [73, 74]. Combining LNA with phosphotriesters or phosphothiotriesters might reverse these undesirable properties, and this will be the focus of future studies. In this limited cell study no oligonucleotides displayed toxicity under the transfection and gymnotic conditions used (Supplementary Figs S157 and S158). A wide variety of alkyl groups have been incorporated in the PTE/PTTE backbones, including multiple alkynes, enabling conjugation with azide derivatives of small molecules (glycol, spermine, glucose, galactose, and lactose) as well as the amino acids lysine, leucine, phenylalanine, and valine. The post-labelling click strategy offers significant potential for incorporating other carbohydrates, oligosaccharides, and peptide-based cell-receptor ligands and a plethora of other biologically active molecules [75–77]. Incorporating small and medium lipophilic groups (such as C3, C4, C6, and C8 alkyl groups) into the LNA PTTE backbone increases duplex stability compared to the 2′-OMe PS+ control, but their duplex stability is lower than the LNA PS control. This mediation of LNA potency might serve to reduce off-target effects of therapeutic oligonucleotide modalities containing LNA sugars. Among the modified oligonucleotides investigated in this study, only the C16 lipid shows negative ΔTm values relative to the 2′OMePS+ control. This indicates that internal lipid triesters require careful redesign to restore duplex stability, possibly by combining them with other modified nucleotides [40]. Amino acids and carbohydrates in the PTTE backbone provide good duplex stability, and glucose, galactose, lactose, and lysine gave the highest Tm values of the click modifications.

Activity data measured in the reporter HeLa pLuc/705 cell line reveal that when a transfection agent is employed, some triester-containing oligonucleotides outperform the fully anionic LNA PS controls. However, under gymnosis conditions, no triester-containing oligonucleotide was as active as the best-performing LNA control (LNA T2). Nevertheless, it is encouraging that multiple charge-neutral LNA-PTTE linkages are compatible with occupancy-only mechanisms, such as splicing modulation. Many modifications did increase splice-switching activity when compared with the all-2′-OMe PS positive control, at least partly due to the positive influence on duplex stability of the LNA sugars. Importantly, our approach allows for subtle variation of physical properties. Exploring this concept, we demonstrated that increasing the length of the lipophilic triester groups (C4→C8→C16) reduces splice-switching activity under gymnosis conditions. Small molecule conjugates, including glycol and spermine, showed slightly better activity under both transfection and gymnosis conditions. Carbohydrate conjugates showed similar activity to the 2′-OMe PS+ control under transfection conditions but surprisingly were not very active under gymnosis conditions. This implies a change in cell uptake or trafficking and could be dependent on cell type. In contrast, amino acid conjugates, particularly phenylalanine, exhibited promising splice-switching activity. In this study every oligonucleotide contains sulphur in all backbone positions. Sulphur is not required for the enzymatic stability of LNA-triester backbones, and future work will focus on replacing some PTTE linkages with PTE linkages to mediate albumin binding with reducing toxicity in mind. Finally, the PTTE triester moiety is a chiral centre, and our methodology does not produce chirally pure triesters. Hence, as for the phosphorothioate oligonucleotides currently in the clinic, each backbone linkage is diastereomeric. Therefore, an interesting avenue of future research is the synthesis and biological properties of stereopure triesters. Unlike stereoisomers of phosphorothioates, in which one enantiomer has reduced resistance to nucleases, the triester functionalities provide additional protection [40].

## Conclusions

Great opportunities exist to explore the vast chemical space offered by charge-neutral backbone-modified oligonucleotides. However, to date robust synthetic procedures have not been available. We have addressed this problem for triesters and produced a wide range of chemically stable charge-neutral PTTE bridges with potentially important chemical, biophysical, and biological properties. Multiple copies of various small molecules, carbohydrates, amino acids, etc. can be attached at any desired number of internal PTTE/PTE positions, whether clustered or dispersed, and the potential of this strategy is very broad, extending into and beyond therapeutics. The conjugation of oligonucleotides to ligands to improve their pharmacological properties is a growing strategy to achieve efficient oligonucleotide targeting and delivery, but to date these conjugates have been mainly limited to the 5′ and 3′ positions. We envisage that our methodology will change this, allowing the straightforward synthesis of oligonucleotides containing multiple internal ligands, leaving the termini for attachment of other molecules such as lipids and antibodies/nanobodies. The cell studies presented here show that PTTE linkages are compatible with occupancy-only splice-switching mechanisms *in vitro*; their effects on biodistribution and toxicology remain to be determined by animal studies. We plan to extend our phosphotriester methodology to RNase H-active ASOs, siRNAs, miRNAs, and anti-MiRs.

## Supplementary Material

gkaf1263_Supplemental_File

## Data Availability

The data underlying this article are available in the article and in its online supplementary material.
