# Supplementary material for: Biophysical and biological properties of splice-switching oligonucleotides and click conjugates containing LNA-phosphothiotriester linkages
Source: Nucleic Acids Res. 2025 Nov 24;53(21):gkaf1263. doi: 10.1093/nar/gkaf1263 (PMC12641260; doi:10.1093/nar/gkaf1263)
Supplement: gkaf1263_Supplemental_File [file gkaf1263_supplemental_file.pdf]

Biophysical and Biological Properties of Splice-Switching Oligonucleotides and Click Conjugates Containing LNA-Phosphothiotriester Linkages

Debashis Dhara[1]*, Alyssa C. Hill[2], Abinaya Ramesh[2], Diallo Traore[1], Ewa Radzikowska-Cieciura[1], Matthew J.A. Wood[2], and Tom Brown[1]*

[1]Department of Chemistry, University of Oxford, Chemistry Research Laboratory, 12 Mansfield Road, Oxford OX1 3TA, United Kingdom

[2]Department of Paediatrics, Institute of Developmental and Regenerative Medicine (IDRM), University of Oxford, Oxford OX3 7TY, United Kingdom

Contents

1.0 Chemical synthesis ... 4

1.1 General procedure ... 4

1.2 Experimental procedure ... 5

1.3 Unsuccessful P(III) reagent and monomer synthesis ... 11

2.0 Oligonucleotide synthesis and analysis ... 12

2.1 Oligonucleotide synthesis, deprotection and purification ... 12

2.2 Click reaction of oligonucleotides ... 14

2.3 Calculated and observed masses of oligonucleotides ... 16

2.4 Yields of solid phase synthesis and click reaction ... 18

2.5 UPLC MS of oligonucleotides ... 20

2.5.1 Butyl T2, Butyl T3 and Butyl T4 ... 21

2.5.2 C16 A1, C16 A2, C16 A2b and C16 A3 ... 24

2.5.3 CF_3 T1, CF_3 T2, CF_3 T3 and CF_3 T4 ... 28

2.5.4 Hexyl T2, Hexyl T3 and Hexyl T4 ... 32

2.5.5 iPr A1, iPr A2, iPr A2b and iPr A3 ... 35

2.5.6 MOP A1, MOP A2, MOP A2b, MOP A3, MOP T1, MOP T2, MOP T3, MOP T4 and MOP T6 ... 39

2.5.7 Octyl T2, Octyl T3 and Octyl T4 ... 48

2.5.8 THP T4 and THP T6 15 ... 51

2.5.9 Hexyn A1 and Hexyn A2 ... 53

2.5.10 Pentyn T1, Pentyn T2 and Pentyn T3 ... 55

2.5.11 Glycol T1, Glycol T2 and Glycol T3 ... 58

2.5.12 Gluc A1 and Gluc A2 ... 61

2.5.13 Gluc T3, Gal T3 and Lac T3 ... 63

2.5.14 Lys T3, Leu T3, Phe T3 and Val T3 ... 66

2.5.15 Sper T1 and Sper T2 ... 70

2.5.16 TO A1, TO A2, TO T1 and TO T2 ... 72

3.0 UV meting experiments ... 76

3.1 Butyl T2, Butyl T3 and Butyl T4 ... 77

3.2 CF_3 T1, CF_3 T2, CF_3 T3 and CF_3 T4 ... 78

3.3 Hexyl T2, Hexyl T3 and Hexyl T4 ... 79

3.4 Octyl T2, Octyl T3 and Octyl T4 ... 80

3.5 Pentyn T1, Pentyn T2 and Pentyn T3 ... 81

3.6 Glycol T1, Glycol T2 and Glycol T3 ... 82

3.7 Gluc A1 and Gluc A2 ... 83

3.8 Gluc T3, Gal T3 and Lac T3 84
3.9 Lys T3, Leu T3, Phe T3 and Val T3 85
3.10 Sper T1 and Sper T2 86
3.11 TO A1, TO A2, TO T1 and TO T2 87
4.0 Circular dichroism (CD) 88
4.1 Butyl T2, Butyl T3 and Butyl T4 89
4.2 CF_3 T1, CF_3 T2, CF_3 T3 and CF_3 T4 90
4.3 Hexyl T2, Hexyl T3 and Hexyl T4 91
4.4 Octyl T2, Octyl T3 and Octyl T4 92
4.5 Pentyn T1, Pentyn T2 and Pentyn T3 93
4.6 Glycol T1, Glycol T2 and Glycol T3 94
4.7 Gluc A1 and Gluc A2 95
4.8 Gluc T3, Gal T3 and Lac T3 96
4.9 Lys T3, Leu T3, Phe T3 and Val T3 97
4.10 Sper T1 and Sper T2 98
4.11 TO A1, TO A2, TO T1 and TO T2 99
5.0 Fluorescence measurements 100
6.0 Biological Assay 101
6.1 Experiments, materials and methods 101
7.0 NMR spectra of compounds 7-11 and 13-17 105
8.0 References 125

1.0 Chemical synthesis

1.1 General procedure

Solvents such as acetonitrile (MeCN), cyclohexane, dichloromethane (DCM), ether (Et_2O), ethyl acetate (EtOAc), hexane, pentane, triethylamine (Et_3N) were collected from an mBraun SPS-800 bench top solvent purification system, having passed through anhydrous alumina columns. Solvents for nucleoside phosphitylation reaction were degassed by bubbling argon through before use. Solvents for purification such as hexane (Hex) and ethyl acetate (EtOAc) were degassed by bubbling argon through and mixed with 1% dry triethylamine. Reactions requiring anhydrous conditions were run under completely an argon atmosphere, using oven-dried glassware which was allowed to attain room temperature whilst flowing dry argon through. Thin layer chromatography (TLC) was performed using Merck pre-coated 0.23 mm thick plates (Kieselgel 60 F254) and visualised using UV light (λ = 254 nm). Flash column chromatography was carried out using silica gel (60 µm particle size).

NMR spectroscopy: Each compound was confirmed by ^{1}H, ^{13}C and ^{31}P NMR spectra were recorded on a Bruker AVIIIHD 400 MHz spectrometer operating at 400 MHz respectively using an internal deuterium lock at ambient probe temperatures, internal service that were provided by the Department of Chemistry NMR Spectroscopy Service, University of Oxford. NMR chemical shifts (δ) are quoted to the nearest 0.01 ppm and are referenced relative to residual solvent peaks. Spectra were recorded in $CDCl_3$ or CD_3CN. Chemical shifts are reported in ppm (δ) relative to residual solvent peaks, $CHCl_3$ in the case of $CDCl_3$ at 7.28/77.0 ppm for the ^{1}H and ^{13}C spectra, respectively. Coupling constants are reported in Hertz (Hz). Signals are reported as s (singlet), d (doublet), t (triplet), dd (doublet of doublet), q (quartet), dt (doublet of triplet), dq (doublet of quarlet), ddd (doublet of doublet of doublet), m (multiplet).

Mass spectroscopy: High-resolution mass spectra (HRMS) were recorded by the Chemistry Department Mass Spectroscopy Service, University of Oxford on a Thermo Scientific Exactive Mass Spectrometer (Waters Equity autosampler and pump) - electrospray ionization (ESI) and an Agilent 7200 Accurate Mass QTOF GCMS (SIM Direct Insertion Probe) for electron ionization (EI) and chemical ionization (CI).

1.2 Experimental procedure

1-sec-Butoxy-N,N,N',N'-tetraisopropylphosphanediamine (7): To a suspension of bis(diisopropylamino)chlorophosphine (**6**, 1.00 g, 3.78 mmol, 1.0 equiv.) in anhydrous Et_2O (10.0 mL) was added triethylamine (1.57 mL, 11.24 mmol, 3.0 equiv.). (R/S)-Butan-2-ol (**1**, 0.69 mL, 7.49 mmol, 2.0 equiv.) was then added slowly dropwise by a syringe. The reaction mixture was left to stir vigorously at rt for 1.5 hrs under an atmosphere of argon. The solution was filtered from the triethylamine salt by cannula filtration. Solids were washed with anhydrous Et_2O (2 x 10 mL). The combined filtrate (~30 mL) was concentrated on a rotary evaporator. The crude product was dried under high vacuum for 30 minutes. To the crude material was added 10 mL of anhydrous MeCN and 10 mL of pentane. The mixture was transferred to a separatory funnel. The acetonitrile part was discarded and the pentane part was collected and concentrated to give the desired product **7** as a colourless oil (crude weight, 728 mg, 2.39 mmol, 64%). ^1H NMR (400 MHz, $CDCl_3$) δ 3.71 (ddt, J = 12.8, 6.4, 4.7 Hz, 1H), 3.51 – 3.47 (m, 4H), 1.22 – 1.11 (m, 30H), 0.89 (t, J = 7.5 Hz, 3H). ^{13}C NMR (101 MHz, $CDCl_3$) δ 71.07, 70.89, 44.67, 44.54, 44.50, 44.37, 31.32, 31.28, 31.18, 31.14, 24.63, 24.60, 24.51, 24.42, 24.32, 24.26, 24.20, 21.21, 21.16, 10.07, 9.56. **^{31}P NMR** (162 MHz, $CDCl_3$) δ 112.68. **HRMS (ESI^+):** m/z $[M+H]^+$ calc. for $C_{16}H_{37}N_2OP$ 305.2716; found 305.2714.

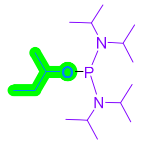

Chemical Formula: $C_{16}H_{37}N_2OP$
Exact Mass: 304.2644
7

1-(Hexan-2-yloxy)-N,N,N',N'-tetraisopropylphosphanediamine (**8**): To a suspension of bis(diisopropyl)chlorophosphine (**6**, 1.0 g, 3.784 mmol, 1.0 equiv.) in anhydrous Et_2O (10.0 mL) and anhydrous triethylamine (1.57 mL, 11.24 mmol, 3.0 equiv.) was added. Then, (R/S)-2-hexanol (**2**, 0.394 mL, 7.56 mmol, 2.0 equiv.) was added and stirred for 2h at room temperature under argon atmosphere. The suspension was filtered from the triethylamine salt by cannula filtration. Solids were washed with Et_2O (2 x 10 mL). The filtrate was concentrated on a rotary evaporator and then dried over high vacuum pump for one hour. The crude was diluted with 15 mL of MeCN and 20 mL of pentane. The mixture was transferred to a separatory funnel. The acetonitrile part was discarded and the pentane part was collected and concentrated to give the crude phosphitylation reagent **8**. Product obtained as a colourless oil (crude weight, 1.10 g, 3.31 mmol, 88%). ^1H NMR (400 MHz, $CDCl_3$) δ 3.76 (dtd, J = 11.2, 6.5, 4.9 Hz, 1H), 3.51 – 3.47 (m, 4H), 1.60 (ddd, J = 12.1, 8.4, 4.7 Hz, 1H), 1.44 (ddd, J = 17.1, 9.0, 5.2 Hz, 1H), 1.31 (tq, J = 6.5, 3.3 Hz, 5H), 1.21 – 1.13 (m, 28H), 0.94 – 0.83 (m, 3H). ^{13}C NMR (101 MHz, $CDCl_3$) δ 76.69, 70.01, 69.83, 44.52, 44.47, 44.39, 44.36, 44.24, 38.24, 38.19, 27.77, 27.45, 24.44, 24.35, 24.27, 24.16, 24.10, 24.05, 23.68, 23.45, 22.95, 22.73, 21.70, 21.65, 14.13. **^{31}P NMR** (162 MHz, $CDCl_3$) δ 112.97. **HRMS (ESI^+):** m/z $[M+NH_4]^+$ calc. for $C_{18}H_{45}N_3OP$ 350.3295; found 350.3306.

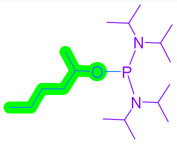

Chemical Formula: $C_{18}H_{41}N_2OP$
Exact Mass: 332.2957
8

1-(Octan-3-yloxy)-N,N,N',N'-tetraisopropylphosphanediamine (9): To a suspension of bis(diisopropyl)chlorophosphine (**6**, 1.0 g, 3.784 mmol, 1.0 equiv.) dissolved in anhydrous Et_2O (10.0 mL). Anhydrous triethylamine (1.57 mL, 11.24 mmol, 3.0 equiv.) was added followed by the addition of (R/S)-3-octanol (**3**, 0.394 mL, 3.75 mmol, 1.0 equiv.). The reaction mixture was left to stir for 2h under argon atmosphere. The reaction mixture was filtered from the triethylamine salt by cannula filtration. Solids washed with dry Et_2O (2 x 10 mL). The combined ether part was concentrated on a rotary evaporator and then dried over high vacuum pump for an hour. The crude was diluted with 20 mL of dry MeCN and 20 mL of dry pentane. The biphasic mixture was transferred to a separatory funnel. The pentane part was collected and the acetonitrile part was discarded. The pentane part was dried on a rotary evaporator and then under high vacuum to obtain the crude P(III) reagent **9** as a colourless oil which get solidified at low temperature quickly (1.0 g., 2.91 mmol, 78%). ^{1}H NMR (400 MHz, $CDCl_3$) δ 3.61 (dt, J = 10.7, 5.7 Hz, 1H), 3.51 – 3.47 (m, 4H), 1.65 – 1.47 (m, 4H), 1.36 – 1.23 (m, 8H), 1.16 (m, 23H), 0.91 – 0.83 (m, 8H). ^{13}C NMR (101 MHz, $CDCl_3$) δ 74.38, 74.23, 44.69, 44.67, 44.56, 44.54, 34.34, 34.29, 32.40, 31.06, 27.43, 27.38, 24.68, 24.52, 24.44, 24.43, 24.41, 24.36, 22.84, 22.80, 14.22, 9.09. **^{31}P NMR** (162 MHz, $CDCl_3$) δ 110.48. **HRMS (ESI$^+$):** *m/z* $[M+NH_4]^+$ calc. for $C_{20}H_{46}N_2OP$ 361.3342; found 361.3360.

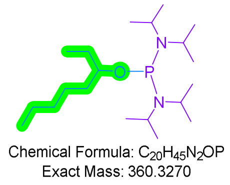

9

N,N,N',N'-tetraisopropyl-1-(pent-4-yn-2-yloxy)phosphanediamine (10): To a suspension of bis(diisopropyl)chlorophosphine (**6**, 1.0 g, 3.784 mmol, 1.0 equiv.) in anhydrous Et_2O (10.0 mL) was added anhydrous triethylamine (1.57 mL, 11.24 mmol, 3.0 equiv.). (R/S)-4-Pentyn-2-ol (**4**, 0.394 mL, 1.0 equiv.) was added and stirred for 2h at room temperature under argon atmosphere. The solution filtered from the triethylamine salt by cannula filtration. Solids washed with Et_2O (2 x 10 mL). The filtrate and washing part were combined and then concentrated on a rotary evaporator and then dried over high vacuum pump. The crude was diluted with anhydrous MeCN (20 mL) and anhydrous pentane (20 mL). The mixture was transferred to a separatory funnel. The acetonitrile part was discarded. The pentane part was collected and concentrated on a rotary evaporator and then dried under high vacuum to give the desired product **10** as a colourless oil (1.0 g, 3.18 mmol, 85%). ^{1}H NMR (400 MHz, $CDCl_3$) δ 3.81 (dddd, J = 12.3, 10.3, 8.2, 5.1 Hz, 1H), 3.54 – 3.38 (m, 4H), 2.48 (ddt, J = 16.4, 4.3, 2.2 Hz, 1H), 2.26 (ddd, J = 16.5, 8.0, 2.5 Hz, 1H), 1.89 (t, J = 2.7 Hz, 1H), 1.26 (d, J = 6.1 Hz, 3H), 1.13 – 1.02 (m, 28H). ^{13}C NMR (101 MHz, $CDCl_3$) δ 81.84, 77.36, 69.87, 68.54, 68.35, 44.72, 44.65, 44.60, 44.52, 28.09, 28.03, 24.56, 24.54, 24.47, 24.46, 24.26, 24.24, 24.21, 24.19, 21.57, 21.52. **^{31}P NMR** (162 MHz, $CDCl_3$) δ 113.84. **HRMS (ESI$^+$):** *m/z* $[M+NH_4]^+$ calc. for $C_{17}H_{36}N_2OP$ 361 315.2560; found 315.2573.

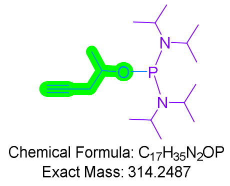

10

N,N,N',N'-tetraisopropyl-1-((tetrahydro-2H-pyran-4-yl)oxy)phosphanediamine (11): To a suspension of bis(diisopropyl)chlorophosphine (**6**, 1.0 g, 3.784 mmol, 1.0 equiv.) in anhydrous Et_2O (10.0 mL) was added anhydrous triethylamine (1.57 mL, 11.24 mmol, 3.0 equiv.). 4-(trifluoromethyl)cyclohexan-1-ol (**4**, 1.14 g, 2.0 equiv.) was added and stirred for 2h at room temperature under argon atmosphere. The solution filtered from the triethylamine salt by cannula filtration. Solids washed with Et_2O (2 x 10 mL). The filtrate and washing part were combined and then concentrated on a rotary evaporator and then dried over high vacuum pump. The crude was diluted with anhydrous MeCN (15 mL) and dry pentane (15 mL). The mixture was transferred to a separatory funnel. The acetonitrile part was discarded. The pentane part was collected and concentrated on a rotary evaporator and then dried under high vacuum to give the desired product **10** as a colourless oil (1.0 g, 3.18 mmol, 67%). ^{1}H NMR (400 MHz, $CDCl_3$) δ 3.81 (dt, J = 11.2, 3.0 Hz, 1H), 3.51 – 3.40 (m, 5H), 2.01 – 1.80 (m, 3H), 1.80 – 1.56 (m, 5H), 1.39 – 1.13 (m, 5H), 1.09 (m, 28H). ^{13}C NMR (101 MHz, $CDCl_3$) δ 129.23, 126.46, 71.82, 71.63, 66.22, 66.06, 44.53, 44.44, 44.40, 44.32, 41.82, 41.56, 41.30, 41.04, 33.04, 32.99, 30.38, 30.34, 24.46, 24.37, 24.35, 24.31, 24.26, 24.01, 23.96, 23.62, 23.60, 19.29, 19.26, 19.23, 19.20. ^{19}F NMR (377 MHz, $CDCl_3$) δ -73.33, -74.00. **^{31}P NMR** (162 MHz, $CDCl_3$) δ 108.80, 114.86. **HRMS (ESI$^+$):** *m/z* $[M+NH_4]^+$ calc. for $C_{19}H_{39}N_2OF_3P$ 361 399.2752; found 399.2755.

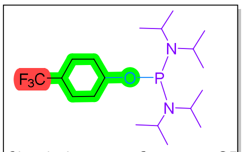

Chemical Formula: $C_{19}H_{38}F_3N_2OP$
Exact Mass: 398.2674
11

5′-*O*-(4,4′-dimethoxytrityl)-2′-*O*,4′-*C*-methylenethymidine-3′-*O*-[(*O*-(butan-2-yl)-*N,N′*-diisopropylphosphoramidite] (13): Nucleic acid monomer **12** (300 mg, 524 µmol, 1.0 equiv.) was dissolved in dry, degassed DCM (10 mL). Tetrazole (~0.45M in MeCN, 1.16 mL, 524 µmol, 1.0 equiv.) and phosphitylating reagent **7** (319 mg, 1.05 mmol, 2.0 equiv.) were added. The reaction was left to stir at rt for 18 h under an argon atmosphere. At completion, the reaction was diluted with dry DCM (10 mL). The reaction mixture was transferred to a separatory funnel and washed with saturated KCl solution (10 mL). The DCM part was collected and dried over Na_2SO_4 and then concentrated on a rotary evaporator. The residue was purified by flash chromatography (50:50 Hex:EtOAc, mixed with 1% triethylamine) to give the desired phosphoramidite **13** (160 mg, 341 µmol, 40%) as a white foam as a mixture of four diastereomers. The phosphoramidite **13** had R_f 0.68 (1:1 Hex:EtOAc, mixed with 1% triethylamine). ^{1}H NMR (400 MHz, CD_3CN) δ 7.61 (dt, J = 2.7, 1.4 Hz, 3H), 7.50 (ddt, J = 8.4, 4.3, 1.5 Hz, 8H), 7.37 (p, J = 1.9 Hz, 8H), 7.37 – 7.25 (m, 16H), 7.28 – 7.20 (m, 3H), 6.87 (dd, J = 9.1, 3.1 Hz, 15H), 5.58 – 5.50 (m, 4H), 4.41 (dd, J = 15.2, 12.7 Hz, 3H), 4.31 (dd, J = 9.7, 5.4 Hz, 1H), 4.19 (dd, J = 14.8, 7.1 Hz, 2H), 4.05 (p, J = 6.7 Hz, 3H), 3.92 – 3.79 (m, 4H), 3.79 (q, J = 4.5 Hz, 4H), 3.76 (s, 21H), 3.59 – 3.48 (m, 8H), 3.51 – 3.34 (m, 8H), 1.97 (s, 3H), 1.43 (ddt, J = 20.8, 13.8, 7.3 Hz, 5H), 1.25 – 0.91 (m, 75H), 0.90 – 0.69 (m, 10H). ^{13}C NMR (101 MHz, CD_3CN) δ 164.22, 159.40, 150.49, 145.46, 136.14, 136.07, 136.03, 134.81, 134.70, 130.79, 130.74, 130.70, 130.68, 130.66, 128.68, 128.58, 128.56, 128.53, 127.60, 127.54, 117.88, 113.74, 113.72, 110.15, 110.12, 88.53, 88.44, 88.38, 87.97, 87.82, 86.95, 79.26, 79.14, 73.05, 72.85, 72.76, 72.69, 72.44, 72.24, 71.55, 71.43, 60.55, 59.14, 59.07, 55.51, 46.58, 45.29, 45.14, 45.00, 44.83, 44.77, 43.62, 43.59, 43.50, 43.46, 31.41, 31.36, 24.71, 24.66, 24.63, 24.58, 24.40, 24.32, 24.29, 24.22, 24.15, 24.09, 24.02, 23.54, 23.51, 22.36, 22.34, 21.69, 21.55, 20.73, 14.10, 12.51, 12.45, 12.42, 9.62, 9.55, 9.52.

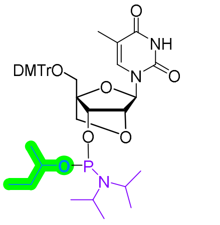

Chemical Formula: $C_{42}H_{54}N_3O_9P$
Exact Mass: 775.3598
13

^{31}**P NMR** (162 MHz, $CDCl_3$) δ 147.65, 147.55, 147.38, 146.37. **HRMS** (ESI^+): m/z $[M+Na]^+$ calc. $C_{42}H_{54}N_3O_9PNa$ 798.3490; found 798.3524.

5′-*O*-(4,4′-dimethoxytrityl)-2′-*O*,4′-*C*-methylenethymidine-3′-*O*-[(*O*-(hexan-2-yl)-*N*,*N*′-diisopropylphosphoramidite] (14): Locked nucleic acid monomer **12** (300 mg, 524 µmol, 1.0 equiv.) was dissolved in dry, degassed DCM (15 mL). Phosphitylating reagent **8** (378 mg, 1.05 mmol, 2.0 equiv.) was added followed by the addition of tetrazole (0.45 M in acetonitrile, 1.16 mL, 524 µmol, 1.0 equiv.). The reaction mixture was left to stir at rt for 16h under an argon atmosphere. At completion, the reaction mixture was diluted with DCM (10 mL) and washed with saturated NaCl solution (20 mL). The DCM part was collected and the aqueous part was washed with DCM (10 mL). The DCM parts were combined and was dried over anhydrous Na_2SO_4, filtered and concentrated under reduced pressure. The crude residue was purified by flash chromatography ((50:50 Hex:EtOAc), both solvent were mixed with 1% triethylamine) to give the desired phosphoramidite **14** (285 mg, 355 µmol, 68%) as a white solid as a mixture of four diastereomers. The phosphoramidite **14** had R_f 0.88 (1:1 Hex:EtOAc, mixed with 1% triethylamine). ^{1}H NMR (400 MHz, $CDCl_3$) δ 7.70 (t, J = 1.9 Hz, 3H), 7.50 – 7.43 (m, 5H), 7.34 (tdd, J = 10.6, 5.0, 3.1 Hz, 11H), 7.32 – 7.20 (m, 4H), 6.84 (dt, J = 9.1, 3.2 Hz, 10H), 5.66 (d, J = 3.7 Hz, 2H), 4.59 (s, 1H), 4.53 (d, J = 3.2 Hz, 1H), 4.32 (t, J = 8.5 Hz, 1H), 4.18 (dd, J = 9.1, 6.9 Hz, 1H), 3.93 – 3.78 (m, 5H), 3.80 (s, 3H), 3.79 (s, 12H), 3.62 – 3.40 (m, 10H), 1.69 – 1.59 (m, 4H), 1.57 (dd, J = 2.7, 1.1 Hz, 3H), 1.37 – 1.17 (m, 11H), 1.15 (s, 3H), 1.14 (d, J = 4.7 Hz, 4H), 1.14 – 1.04 (m, 15H), 1.04 (s, 4H), 1.03 – 0.77 (m, 22H). ^{13}C NMR (101 MHz, $CDCl_3$, partial) δ, 158.85, 149.61, 144.57, 134.84, 130.46, 130.41, 130.35, 130.30, 128.54, 128.37, 128.10, 127.20, 113.41, 113.38, 113.34, 110.33, 87.68, 86.82, 77.36, 72.56, 70.82, 58.52, 55.36, 43.22, 38.09, 27.81, 27.67, 24.43, 22.76, 22.48, 14.20, 12.60. ^{31}**P NMR** (162 MHz, $CDCl_3$) δ 148.08, 148.06, 147.22, 146.24. **HRMS** (ESI^+): m/z $[M+H]^+$ calc. $C_{44}H_{59}N_3O_9P$ 804.3984; found 804.3988.

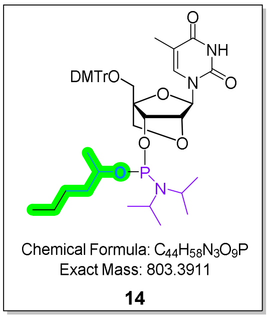

5′-*O*-(4,4′-dimethoxytrityl)-2′-*O*,4′-*C*-methylenethymidine-3′-*O*-[(*O*-(octan-3-yl)-*N*,*N*′-diisopropylphosphoramidite] (15): Locked nucleic acid monomer **12** (300 mg, 1.0 equiv., 524 µmol) was dissolved in dry, degassed DCM (15 ml). Phosphitylating reagent **9** (378 mg, 2.0 equiv., 1.05 mmol) was added followed by the addition of tetrazole (0.45 M in acetonitrile, 1.16 mL, 524 µmol, 1.0 equiv.). The reaction mixture was left to stir at rt for 24h under an atmosphere of argon. At completion, the reaction was diluted with 10 mL of DCM. The diluted reaction mixture was transferred ot a separatory funnel and washed with saturated NaCl solution (20 mL). The DCM part was collected and dried over anhydrous $MgSO_4$, filtered, and concentrated on a rotary evaporator. The crude residue was purified by flash chromatography ((50:50 Hex:EtOAc), both solvent were mixed with 1% triethylamine) to give the desired phosphoramidite **15** (250 mg, 300 µmol, 57%) as a white solid as a mixture of four diastereomers. The phosphoramidite **15** had R_f 0.82 (1:1 Hex:EtOAc, mixed with 1% triethylamine). ^{1}H NMR (400 MHz, $CDCl_3$) δ 8.14 (s, 2H), 7.41 – 7.33 (m, 4H), 7.31 – 7.11

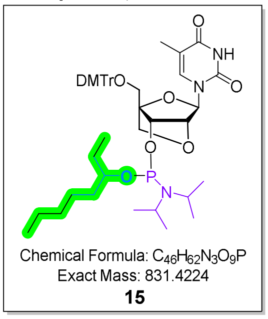

(m, 13H), 6.75 (dt, J = 9.0, 2.9 Hz, 9H), 5.57 (d, J = 2.9 Hz, 2H), 4.53 (s, 1H), 4.46 (s, 1H), 4.23 (d, J = 8.8 Hz, 1H), 4.08 (d, J = 6.6 Hz, 1H), 3.83 – 3.56 (m, 18H), 3.53 – 3.30 (m, 9H), 1.56 (d, J = 1.1 Hz, 1H), 1.48 (d, J = 1.1 Hz, 3H), 1.45 – 1.23 (m, 3H), 1.16 (s, 16H), 1.16 – 1.04 (m, 6H), 1.07 – 0.98 (m, 9H), 0.96 (d, J = 6.8 Hz, 6H), 0.90 (d, J = 6.8 Hz, 6H), 0.82 – 0.73 (m, 10H), 0.59 (t, J = 7.4 Hz, 3H). ^{13}C NMR (101 MHz, $CDCl_3$) δ 163.63, 163.56, 158.84, 149.61, 144.56, 144.42, 135.67, 135.52, 135.44, 134.86, 134.79, 130.45, 130.40, 130.33, 130.27, 128.53, 128.35, 128.09, 127.19, 113.40, 113.37, 113.34, 110.32, 110.26, 88.28, 87.69, 86.84, 79.75, 78.98, 78.70, 76.45, 75.84, 72.47, 70.86, 58.53, 55.35, 43.31, 43.19, 35.39, 35.10, 32.11, 31.99, 30.89, 28.26, 25.08, 24.99, 24.80, 24.61, 24.47, 24.41, 24.35, 22.77, 22.73, 14.22, 14.17, 12.63, 12.51, 9.45, 9.39. **^{31}P NMR** (162 MHz, $CDCl_3$) δ 148.03, 147.31. **HRMS** (ESI$^+$): m/z [M+H]$^+$ calc. $C_{46}H_{63}N_3O_9P$ 832.4297; found 832.4314.

5′-*O*-(4,4′-dimethoxytrityl)-2′-*O*,4′-*C*-methylenethymidine-3′-*O*-[(*O*-(4-pentyn-2-yl)-*N,N*′-diisopropylphosphoramidite] (16): Locked nucleic acid monomer **12** (600 mg, 1.05 mmol, 1.0 equiv.) was dissolved in dry, degassed DCM (15 ml). Phosphitylating reagent **10** (659 mg, 2.10 mmol, 2.0 equiv.) was added followed by the addition of tetrazole (0.45 M in acetonitrile, 2.62 mL, 1.05 mmol, 1.0 equiv.). The reaction mixture was left to stir at rt for 16h under an atmosphere of argon. At completion, the reaction was diluted with 25 mL of DCM and washed with saturated NaCl solution (50 mL). The DCM part was dried over anhydrous Na_2SO_4, filtered and concentrated under reduced pressure at rt. The crude residue was purified by flash chromatography (60:40→50:50 Hex:EtOAc, both solvent were mixed with 1% triethylamine) to give the desired phosphoramidite **16** (650 mg, 827 µmol, 79%) as a white solid as a mixture of four diastereomers. The phosphoramidite **16** had R_f 0.98 (1:4 Hex:EtOAc, mixed with 1% triethylamine). ^{1}H NMR (400 MHz, $CDCl_3$) δ 8.17 (s, 1H), 7.69 (d, J = 6.1 Hz, 1H), 7.51 – 7.42 (m, 2H), 7.33 (dtd, J = 16.7, 6.8, 4.5 Hz, 6H), 7.25 (d, J = 8.2 Hz, 1H), 6.84 (ddd, J = 8.6, 5.3, 2.8 Hz, 5H), 5.66 (d, J = 2.6 Hz, 1H), 4.66 – 4.53 (m, 1H), 4.38 – 4.25 (m, 1H), 4.20 (d, J = 7.0 Hz, 0H), 4.03 (dq, J = 11.3, 5.9 Hz, 1H), 3.92 – 3.83 (m, 1H), 3.79 (d, J = 1.8 Hz, 8H), 3.61 – 3.38 (m, 5H), 2.44 – 2.19 (m, 2H), 2.07 – 1.96 (m, 1H), 1.61 (d, J = 17.1 Hz, 2H), 1.49 – 1.39 (m, 1H), 1.37 – 1.18 (m, 9H), 1.18 – 0.97 (m, 14H), 0.88 (t, J = 6.9 Hz, 3H). ^{13}C NMR (101 MHz, $CDCl_3$) δ 163.63, 158.84, 149.60, 144.51, 135.44, 134.91, 134.79, 130.45, 130.33, 130.27, 128.48, 128.32, 128.11, 127.23, 124.38, 113.41, 110.39, 87.70, 86.83, 80.90, 78.91, 78.21, 77.36, 70.83, 70.70, 70.61, 70.04, 58.46, 55.36, 45.33, 45.27, 43.46, 43.33, 28.09, 27.88, 24.53, 23.15, 23.12, 22.96, 22.93, 21.72, 21.16, 12.60, 9.96. **^{31}P NMR** (162 MHz, $CDCl_3$) δ 148.09, 148.01, 147.80, 146.40. **HRMS** (ESI$^+$): m/z [M+Na]$^+$ calc. $C_{43}H_{52}N_3O_9P$ 808.3333; found 808.3359.

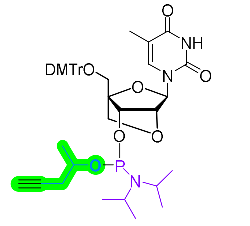

Chemical Formula: $C_{43}H_{52}N_3O_9P$
Exact Mass: 785.3441
16

5′-*O*-(4,4′-dimethoxytrityl)-2′-*O*,4′-*C*-methylenethymidine-3′-*O*-[(*O*-(4-trifluoromethylcyclohexyl-1-yl)-*N*,*N*′-diisopropylphosphoramidite] (17): Locked nucleic acid monomer **12** (300 mg, 524 µmol, 1.0 equiv.) was dissolved in dry, degassed DCM (10 mL). Phosphitylating reagent **11** (418 mg, 1.05 mmol, 2.0 equiv.) was added followed by the addition of tetrazole (0.45 M in acetonitrile, 1.16 mL, 524 µmol, 1.0 equiv.). The reaction mixture was left to stir at rt for 16h under an atmosphere of argon. At completion, the reaction was diluted with dry, degassed DCM (10 mL). The reaction mixture was washed with saturated NaCl solution (10 mL). The DCM part was passed though $MgSO_4$ and then concentrated in a rotary evaporator. The crude product was purified by flash chromatography (50:50 Hex:EtOAc, mixed with 1% triethylamine) to give the DMT-LNA-T-THP phosphoramidite **17** (320 mg, 368 µmol, 70%) as a white solid as a mixture of diastereomers. The phosphoramidite **17** had R_f 0.72 (1:1, Hex:EtOAc, mix with 1% Et_3N). ^{1}H NMR (400 MHz, $CDCl_3$) δ 8.16 (s, 2H), 7.77 – 7.66 (m, 3H), 7.46 (dq, J = 8.2, 3.2 Hz, 5H), 7.33 (ddt, J = 8.5, 6.7, 3.2 Hz, 11H), 7.31 – 7.20 (m, 4H), 6.83 (dt, J = 9.0, 3.6 Hz, 10H), 5.69 – 5.62 (m, 2H), 4.60 – 4.50 (m, 2H), 4.29 (dd, J = 9.1, 3.8 Hz, 1H), 4.18 (dd, J = 7.2, 2.6 Hz, 1H), 4.07 (d, J = 9.9 Hz, 1H), 3.88 – 3.73 (m, 19H), 3.64 – 3.43 (m, 8H), 3.40 (dd, J = 10.8, 3.4 Hz, 2H), 2.21 – 1.72 (m, 2H), 1.72 – 1.47 (m, 14H), 1.44 (d, J = 8.4 Hz, 0H), 1.35 – 1.20 (m, 13H), 1.20 – 1.03 (m, 21H), 1.00 (dd, J = 6.8, 3.5 Hz, 8H), 0.88 (t, J = 6.9 Hz, 5H). ^{13}C NMR (101 MHz, $CDCl_3$) δ 220.69, 163.53, 158.88, 144.52, 135.46, 134.63, 130.45, 130.35, 130.28, 128.52, 128.36, 128.10, 127.26, 113.38, 88.17, 87.61, 86.88, 77.36, 75.60, 72.51, 58.44, 55.36, 45.28, 43.31, 41.10, 30.72, 24.71, 24.46, 22.94, 22.48, 19.20, 14.20. **^{31}P NMR** (162 MHz, $CDCl_3$) δ 147.53, 147.36, 147.04, 146.31. **HRMS** (ESI^+): m/z $[M+Na]^+$ calc. $C_{45}H_{55}N_3O_9F_3PNa$ 892.3520; found 892.3543.

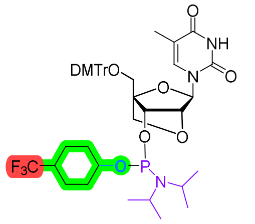

Chemical Formula: $C_{45}H_{55}F_3N_3O_9P$
Exact Mass: 869.3628
17

1.3 Unsuccessful P(III) reagent and monomer synthesis

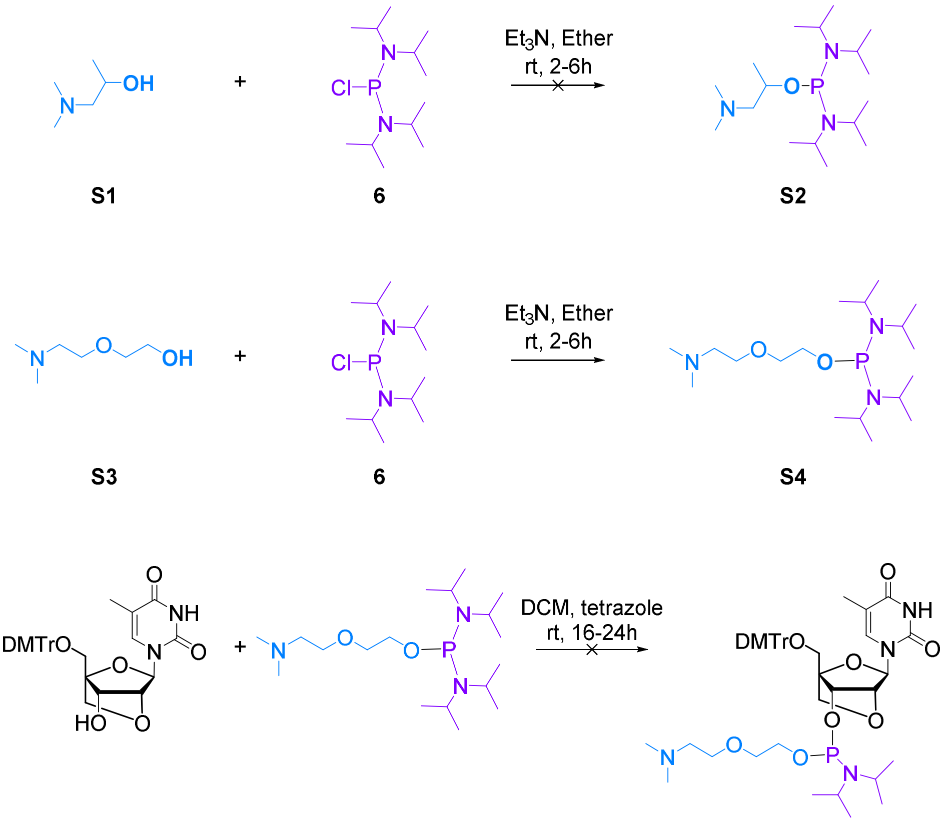

S1 6 S2 S3 6 S4 12 S4 S5

Supplementary Scheme S1: Unsuccessful reactions of synthesis of P(III) reagents and P(III) monomers.

2.0 Oligonucleotide synthesis and analysis

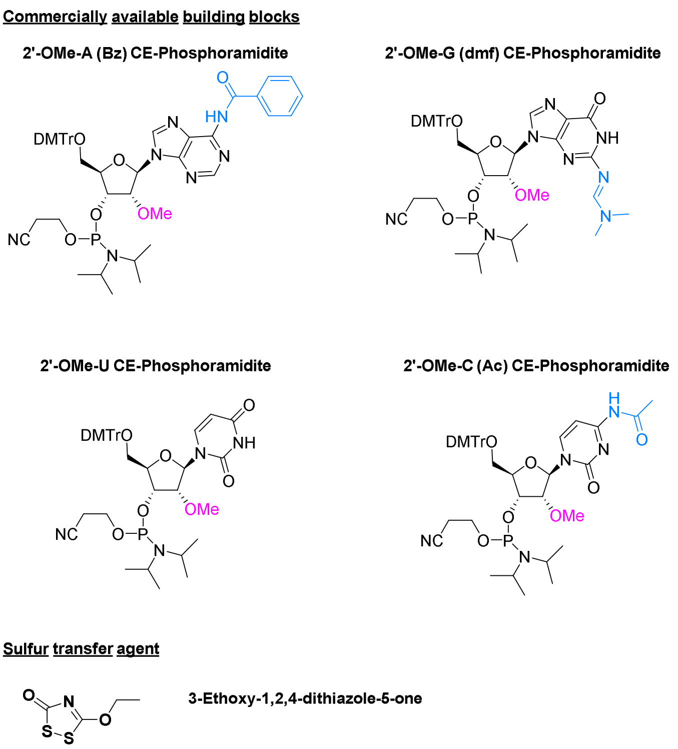

Supplementary Figure S1: List of monomers and sulfur transfer agent used in the solid phase oligonucleotide synthesis.

2.1 Oligonucleotide synthesis, deprotection and purification

2′OMePS+, 2′OMePS-, and modified oligonucleotide synthesis

2′-OMe oligonucleotides were synthesised on an Applied Biosystems 394 automated DNA/RNA synthesiser using a standard phosphoramidite cycle of detritylation, coupling, capping, and sulfurisation/oxidation on a 1.0 µmole scale. Detritylation, coupling, capping, oxidation and activation reagents are identical to those used for standard DNA/RNA synthesis. Trichloroacetic acid (TCA) (3% in CH_2Cl_2) was used for detritylation, 5-Benzylthio-1*H*-tetrazole (BTT) (0.3 M in MeCN) was used as an activator, and sulfurization was achieved using 3-ethoxy-1,2,4-dithiazole-5-one. Pre-packed nucleoside SynBase™ CPG 1000/110 resins (Link Technologies) were used, and β-cyanoethyl phosphoramidite monomers (DMT-2′O-methyl-rA(Bz), DMT-2′O-methyl-rG(dmf), DMT-2′-O-methyl-rC(Ac) and DMT-2′O-methyl-rU were dissolved in anhydrous MeCN (10% CH_2Cl_2 was added when 2′OMe U phosphoramidite was used) to a concentration of 0.1 M immediately prior to use with a coupling time of 6 min. LNA phosphoramidite monomers (**13-23**) were dissolved to a concentration of 0.1 M MeCN immediately prior to use with a coupling time of 6 min. Stepwise coupling efficiencies were determined by automated trityl cation conductivity monitoring and were >97% in all cases. In all cases after solid phase oligonucleotide synthesis the DMT group

at the 5′-position was not removed from the oligonucleotide. The oligonucleotide was subject to cleavage from the solid support and deprotected and purified by HPLC to give highly purified DMT intact oligonucleotides. The DMT group was then cleaved in liquid phase by treatment with 80% AcOH.

Deprotection from the solid support (1 µM Scale)

Cleavage and deprotection were achieved by treatment with 0.5 mL of THF and 0.5 mL of ethylenediamine (EDA) for 2 hrs room temperature. The mixture of EDA-THF was discarded washed with THF (1mL) if needed and dried and then the resin was washed with 1 mL of DNase free water to give the crude oligonucleotides. The DMTON oligonucleotides were purified by HPLC and then DMT group was removed by using the procedure below.

Condition of DMT-group removal (1 µM Scale)

100 µL of 80% AcOH was added to the dry purified DMTON oligonucleotide. The reaction was left for 30 mins at rt. The solution became light red colour. The reaction was quenched by 600 µL of water and 300 µL of 2M TEAA buffer (pH 8.5). The de-tritylated oligonucleotide were desalted using NAP-10 (Cytiva) and then freeze dried or purified by HPLC to give the final pure oligonucleotides. The oligonucleotides were characterised by UPLC-MS.

Synthesis of Complementary DNA, dTGTAACTGAGGTAAGAGG (5′→3′)

DNA synthesis was performed on an Applied Biosystems 394 automated DNA/RNA synthesiser using a standard phosphoramidite cycle of detritylation, coupling, capping, and oxidation on a 1.0 µmole scale. Trichloroacetic acid (TCA) (3% in dichloromethane) was used for detritylation, ethylthiotetrazole (ETT) (0.25 M in MeCN) was used as an activator, and oxidation was achieved using iodine (0.02 M in THF, pyridine and water). Pre-packed nucleoside SynBase™ CPG 1000/110 (Link Technologies) were used and β-cyanoethyl phosphoramidite monomers (dA(Bz), dG(iBu), dC+(Bz) and dT, Sigma-Aldrich) were dissolved in anhydrous MeCN (0.1 M) immediately prior to use with coupling time of 40 s. Stepwise coupling efficiencies were determined by automated trityl cation conductivity monitoring and were >98% in all cases. Deprotection from the solid support was achieved by heating with concentrated aqueous ammonia in a sealed tube for 5 h at 55 °C. The suspension was filtered and washed with water. The filtrate was concentrated under reduced pressure and dissolved in water (1 mL). The solution was transferred to a 10 mL falcon tube and then lyophilised. The crude product was purified by reversed-phase HPLC to give the target DNA. The complementary DNA was characterised by UPLC-MS.

Synthesis of Complementary RNA, rUGUAACUGAGGUAAGAGG (5′→3′)

RNA synthesis was performed on an Applied Biosystems 394 automated DNA/RNA synthesiser using a standard phosphoramidite cycle of detritylation, coupling, capping, and oxidation on a 1.0 µmole scale. Coupling, capping and oxidation reagents were identical to those used for DNA synthesis except a solution of 5-benzylthio-1*H*-tetrazole (BTT) (0.3 M in MeCN, Link Technologies) was used. Standard CPG resin (Link Technologies) was used and

2'-OTBDMS protected monomers (A(Bz), C(Ac), G(iBu) and U, Sigma-Aldrich) were dissolved in anhydrous MeCN (0.1 M) immediately prior to use. The coupling time for all monomers was 6 min. Stepwise coupling efficiencies were determined by automated trityl cation conductivity monitoring and in all cases were >96%. The complementary RNA was deprotected from solid support in two steps.

Step 1: 1 mL of NH_4OH and 1 mL of methylamine were added to the solid support in a 5 mL sealed glass vial. The vial was placed in heating bath at 65 °C for 20-25 mins. The reaction mixture was allowed to attain rt. The suspension was filtered and washed with 40% MeCN in water (2*1 mL). The filtrate was concentrated under vacuum (55 °C) until the smell of ammonia goes off and the volume reduced to ~1-2 mL. The residual solution was transferred to a falcon tube and then lyophilised.

Step 2: The crude lyophilised mass was treated with 300 µL of anhydrous DMSO and 300 µL of $Et_3N.HF$. The mixture was left at 65 °C for 3h. The mixture was allowed to attain rt and 50 µL of NaOAc (3M, pH 5.2) and 3 mL of 1-butanol were added. The tube was centrifuged for 30 mins (rt, 5000 rpm). The supernatants were discarded**.** Then, 1 mL 70% aqueous ethanol was added and centrifuged for 30 mins (rt, 5000 rpm). The supernatant was discarded and the procedure repeated two more times. The crude product was then lyophilised, dissolved in water (1.0 mL) and then centrifuged. The precipitate was discarded and the solution was collected which contained the desired RNA. The crude product was purified by reversed-phase HPLC, fractions were collected and lyophilised to give the target RNA. The target RNA was characterised by UPLC-MS.

2.2 Click reaction of oligonucleotides

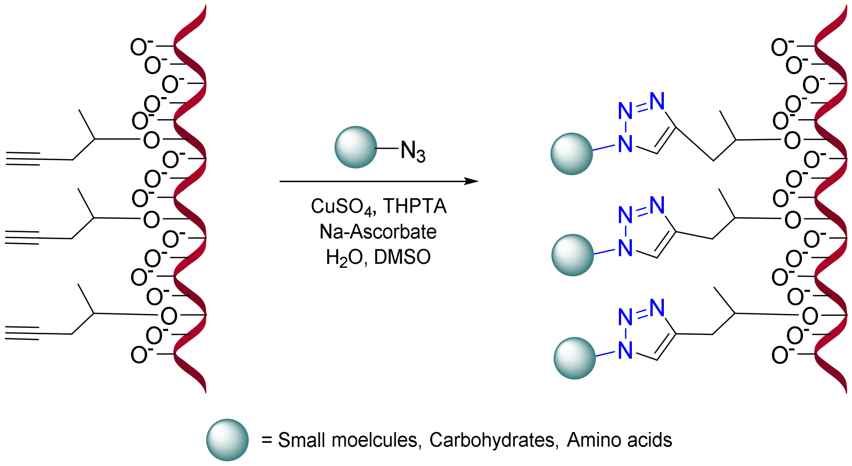

Supplementary Figure S2: CuAAC reaction between pentynyl oligonucleotides and azide small molecules

Procedure 1 (scale 5 nmole): A solution of modified oligonucleotide (5 nmoles, 1 equiv.) in TEAA buffer (0.1 M, pH = 7.0; 20 µL), and stock of azide (100 nmoles in water, 20 equiv., 10 µL of 10 mM concentration) and sodium ascorbate (250 nmoles in water, 50 equiv., 25 µL of

10 mM concentration) were vortexed together. The mixture was degassed by bubbling argon gas through for 5 min followed by addition of Cu (II): THPTA (tris(3-hydroxypropyltriazolylmethyl)amine) complex (50 nmoles in 55%/45% DMSO/H_2O, 10.0 equiv., 5 µL of 10 mM concentration). Reaction was shaken at 25 °C for 3h for single labelling, longer for multiple labelling (up to 24h). The mixture was desalted by NAP 10 gel filtration, then lyophilised. The crude oligonucleotide was purified by HPLC to obtain the desired oligonucleotide. The oligonucleotide was analysed by UPLC-MS analysis.

Procedure 1 (scale 50-150 nmole): A solution of modified oligonucleotide (150 nmoles in 200 µL water, 1 equiv.) in TEAA buffer (0.1 M, pH = 7.0; 200 µL), and stock of azide (1.5 µmoles in water (or DMSO, for the azides which are not soluble in water), 10 equiv., 30 µL of 500 mM concentration) and sodium ascorbate (1.5 µmoles in water, 10 equiv., 30 µL of 500 mM concentration) were added. Freshly prepared THPTA (tris(3-hydroxypropyltriazolylmethyl)amine) (1.5 µmoles in H_2O, 10 equiv., 7.5 µL of 200 mM concentration) was added to the reaction mixture. The mixture was degassed by bubbling argon gas through for 5-10 min followed by addition of Freshly prepared $CuSO_4.5H_2O$ (II) (750 nmoles in H_2O, 5 equiv., 3.75 µL of 200 mM concentration) was added. The reaction mixture was degassed by purging argon gas through for 1-2 mins. Reaction was shaken at 25 °C for 24h for to ensure multiple labelling. The reaction mixture desalted using Amicon® Ultra Centrifugal Filter, 3 kDa MWCO and washed with 0.1 M EDTA three times to remove any intact Cu(II) salts. Th oligonucleotide was finally purified by HPLC to obtain the desired oligonucleotide. The final oligonucleotide was analysed by UPLC-MS analysis.

2.3 Calculated and observed masses of oligonucleotides

<u>Supplementary Table T1:</u> Nucleotides in black have 2´-OMe ribose sugars and phosphorothioate internucleoside linkages. Nucleotides in red are LNA T-alkyl phosphothiotriesters, and in blue are LNA A-alkyl phosphothiotriesters.

ON	Sequence (5’→3’)	Mass Expected	Mass Observed
Butyl T2	CCU CUT ACC UCA GUT ACA	6235.12	6235.70
Butyl T3	CCU CUT ACC TCA GUT ACA	6303.23	6303.40
Butyl T4	CCT CUT ACC TCA GUT ACA	6371.35	6369.80
C16 A1	CCU CUU ACC UCA GUU ACA	6321.29	6321.50
C16 A2	CCU CUU ACC UCA GUU ACA	6543.71	6543.80
C16 A2b	CCU CUU ACC UCA GUU ACA	6543.71	6543.90
C16 A3	CCU CUU ACC UCA GUU ACA	6766.13	6765.50
CF_3 T1	CCU CUU ACC UCA GUT ACA	6261.03	6261.00
CF_3 T2	CCU CUT ACC UCA GUT ACA	6423.19	6423.10
CF_3 T3	CCU CUT ACC TCA GUT ACA	6585.34	6585.50
CF_3 T4	CCT CUT ACC TCA GUT ACA	6747.50	6747.50
Hexyl T2	CCU CUT ACC UCA GUT ACA	6291.22	6290.70
Hexyl T3	CCU CUT ACC TCA GUT ACA	6387.40	6387.40
Hexyl T4	CCT CUT ACC TCA GUT ACA	6483.57	6483.90
iPr A1	CCU CUU ACC UCA GUU ACA	6138.94	6138.80
iPr A2	CCU CUU ACC UCA GUU ACA	6179.01	6179.00
iPr A2b	CCU CUU ACC UCA GUU ACA	6179.01	6179.66
iPr A3	CCU CUU ACC UCA GUU ACA	6219.07	6219.10
MOP A1	CCU CUU ACC UCA GUU ACA	6168.97	6169.00
MOP A2	CCU CUU ACC UCA GUU ACA	6239.06	6239.10
MOP A2b	CCU CUU ACC UCA GUU ACA	6239.06	6238.73
MOP A3	CCU CUU ACC UCA GUU ACA	6309.15	6308.80
MOP T1	CCU CUU ACC UCA GUT ACA	6183.00	6183.20
MOP T2	CCU CUT ACC UCA GUT ACA	6267.11	6267.20
MOP T3	CCU CUT ACC TCA GUT ACA	6351.23	6351.30
MOP T4	CCT CUT ACC TCA GUT ACA	6435.35	6435.40
MOP T6	CCT CTT ACC TCA GTT ACA	6603.59	6603.30
Octyl T2	CCU CUT ACC UCA GUT ACA	6347.33	6347.90
Octyl T3	CCU CUT ACC TCA GUT ACA	6471.56	6471.50
Octyl T4	CCT CUT ACC TCA GUT ACA	6595.79	6595.10
THP T4	CCT CUT ACC TCA GUT ACA	6483.39	6483.40
THP T6 15	T CTT ACC TCA GTT AC	5645.81	5645.83
Hexyn A1	CCU CUU ACC UCA GUU ACA	6176.99	6177.10
Hexyn A2	CCU CUU ACC UCA GUU ACA	6255.10	6255.00
Pentyn T1	CCU CUU ACC UCA GUT ACA	6176.99	6176.60
Pentyn T2	CCU CUT ACC UCA GUT ACA	6255.11	6255.70
Pentyn T3	CCU CUT ACC TCA GUT ACA	6333.22	6333.70
Glycol T1	CCU CUU ACC UCA GUT ACA	6572.44	6570.80
Glycol T2	CCU CUT ACC UCA GUT ACA	7046.01	7046.20
Glycol T3	CCU CUT ACC TCA GUT ACA	7519.58	7519.50
Gluc A1	CCU CUU ACC UCA GUU ACA	6382.16	6381.80

Gluc A2	CCU CUU ACC UCA GUU ACA	6665.45	6664.90
Gluc T3 p	CCU CUT ACC TCA GUT ACA	6948.73	6948.90
Gal T3 p	CCU CUT ACC TCA GUT ACA	6948.73	6948.90
Lac T3 p	CCU CUT ACC TCA GUT ACA	7435.15	7434.10
Lys T3 p	CCU CUT ACC TCA GUT ACA	6849.78	6849.00
Leu T3 p	CCU CUT ACC TCA GUT ACA	6804.74	6803.90
Phe T3 p	CCU CUT ACC TCA GUT ACA	6993.92	6993.20
Val T3 p	CCU CUT ACC TCA GUT ACA	6762.66	6761.90
Sper T1	CCU CUU ACC UCA GUT ACA	6405.34	6405.80
Sper T2	CCU CUT ACC UCA GUT ACA	6711.79	6711.60
TO A1	CCU CUU ACC UCA GUU ACA	6593.56	6592.40
TO A2	CCU CUU ACC UCA GUU ACA	7088.24	7086.40
TO T1	CCU CUU ACC UCA GUT ACA	6593.56	6592.30
TO T2	CCU CUT ACC UCA GUT ACA	7088.24	7086.00

2.4 Yields of solid phase synthesis and click reaction

Supplementary Table T2: yield of solid phase synthesis

Nucleotides in black have 2´-OMe ribose sugars and phosphorothioate internucleoside linkages. Nucleotides in red are locked nucleic acid phosphorothioate triesters.

ON	Sequence (5’→3’)	Product Obtained (nM)	Yield (%)
Butyl T2	CCU CUT ACC UCA GUT ACA	430	43
Butyl T3	CCU CUT ACC TCA GUT ACA	369	37
Butyl T4	CCT CUT ACC TCA GUT ACA	213	21
CF_3 T1	CCU CUU ACC UCA GUT ACA	236	24
CF_3 T2	CCU CUT ACC UCA GUT ACA	299	30
CF_3 T3	CCU CUT ACC TCA GUT ACA	562	56
CF_3 T4	CCT CUT ACC TCA GUT ACA	280	28
Hexyl T2	CCU CUT ACC UCA GUT ACA	473	47
Hexyl T3	CCU CUT ACC TCA GUT ACA	447	45
Hexyl T4	CCT CUT ACC TCA GUT ACA	307	31
Octyl T2	CCU CUT ACC UCA GUT ACA	484	48
Octyl T3	CCU CUT ACC TCA GUT ACA	247	25
Octyl T4	CCT CUT ACC TCA GUT ACA	702	70
Pentyn T1	CCU CUU ACC UCA GUT ACA	494	49
Pentyn T2	CCU CUT ACC UCA GUT ACA	336	34
Pentyn T3	CCU CUT ACC TCA GUT ACA	373	37

Supplementary Table T3: yield of CuAAC click reaction

Nucleotides in black have 2´-OMe ribose sugars and phosphorothioate internucleoside linkages. Nucleotides in red are LNA T-alkyl phosphothiotriesters, and in blue are LNA A-alkyl phosphothiotriesters. All reactions were performed in 20-150 nmole scale.

ON	Sequence (5’→3’)	Starting material (nM)	Product Obtained (nM)	Yield (%)
Glycol T1	CCU CUU ACC UCA GUT ACA	150	141	94
Glycol T2	CCU CUT ACC UCA GUT ACA	150	100	62
Glycol T3	CCU CUT ACC TCA GUT ACA	150	100	66
Gluc A1	CCU CUU ACC UCA GUU ACA	150	141	94
Gluc A2	CCU CUU ACC UCA GUU ACA	150	135	90
Gluc T3	CCU CUT ACC TCA GUT ACA	150	142	95
Gal T3	CCU CUT ACC TCA GUT ACA	150	145	97
Lac T3	CCU CUT ACC TCA GUT ACA	150	146	97
Lys T3	CCU CUT ACC TCA GUT ACA	150	142	95
Leu T3	CCU CUT ACC TCA GUT ACA	150	148	98
Phe T3	CCU CUT ACC TCA GUT ACA	150	147	98
Val T3	CCU CUT ACC TCA GUT ACA	150	132	88
Sper T1	CCU CUU ACC UCA GUT ACA	150	135	90
Sper T2	CCU CUT ACC UCA GUT ACA	50	21	42

Sper T3	CCU CUT ACC TCA GUT ACA	50	00	00
TO A1	CCU CUU ACC UCA GUU ACA	50	32	64
TO A2	CCU CUU ACC UCA GUU ACA	20	9	45
TO T1	CCU CUU ACC UCA GUT ACA	100	71	71
TO T2	CCU CUT ACC UCA GUT ACA	100	62	62

2.5 UPLC MS of oligonucleotides

All oligonucleotides were characterised by negative-mode ultra-performance liquid chromatography (UPLC) mass spectrometry using a Waters Xevo G2-XS QT of mass spectrometer with an Acquity UPLC system. The system is equipped with an Acquity UPLC oligonucleotide BEH C18 column (particle size: 1.7 µm; pore size: 130 Å; column dimensions: 2.1 mm x 50 mm). Data were analysed using Waters MassLynx software v 4.1 or Waters UNIFI Scientific Information System software.

2.5.1 Butyl T2, Butyl T3 and Butyl T4

Butyl T2	CCU CUT ACC UCA GUT ACA	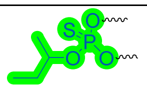

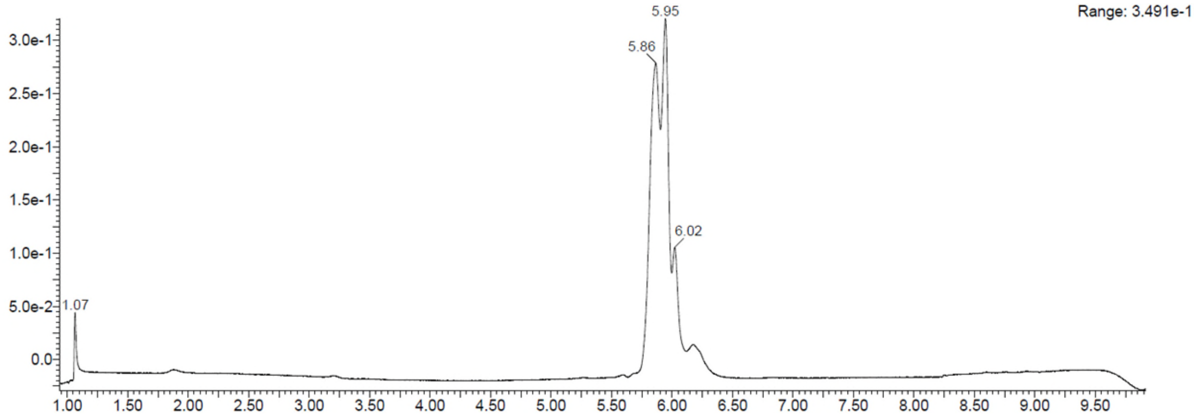

Supplementary Figure S3: Reverse-phase UPLC of **Butyl T2** (UV absorbance at 260 nm vs time in min).

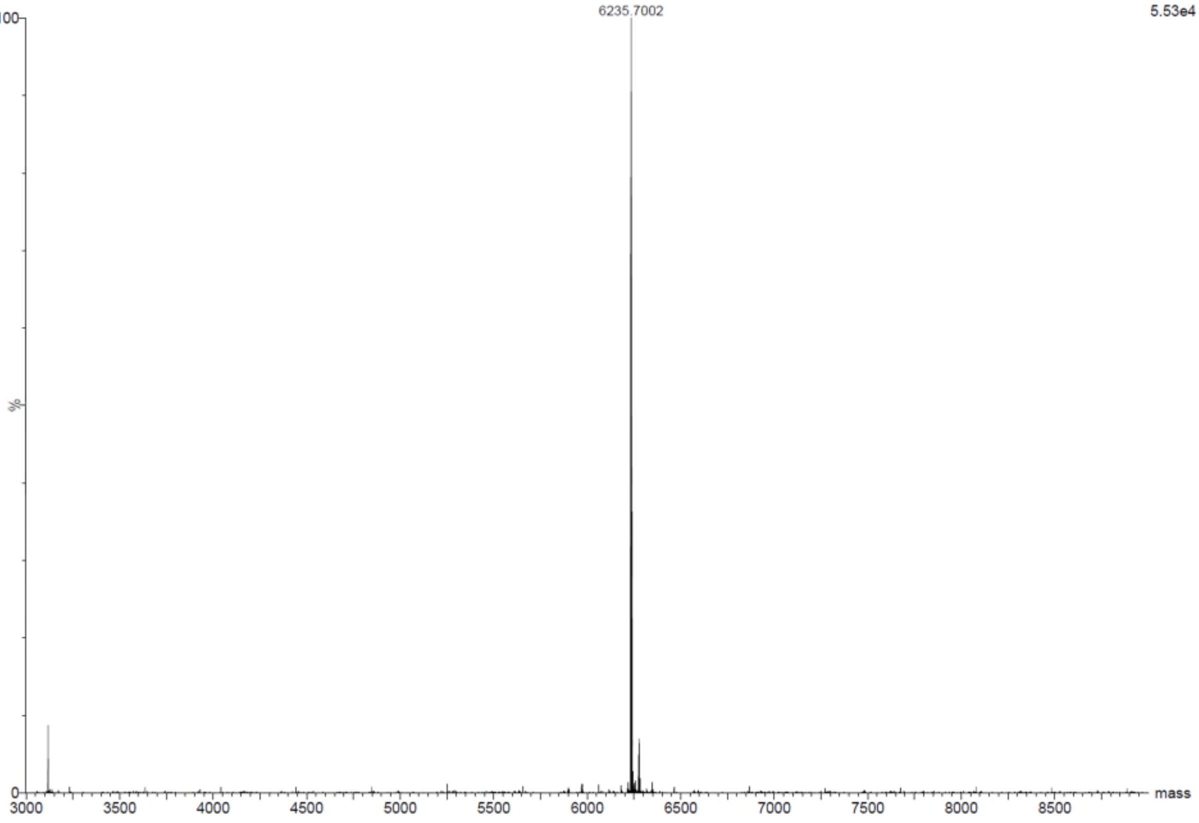

Supplementary Figure S4: Mass spectrum (ES-) of **Butyl T2**. Required **6235.12** Da, found **6235.70** Da. y-axis = relative intensity (%), x-axis = mass in Da.

Butyl T3	CCU CUT ACC TCA GUT ACA	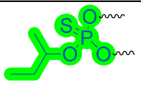

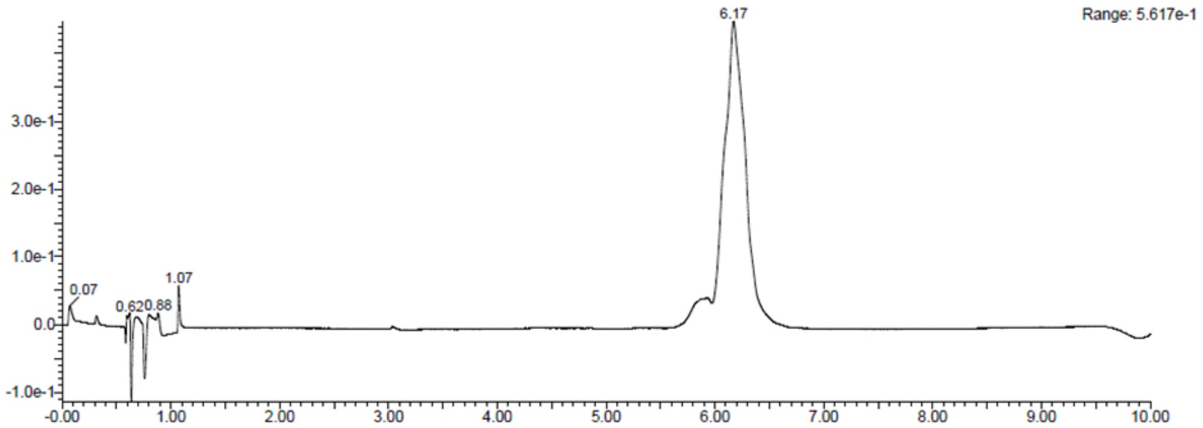

Supplementary Figure S5: Reverse-phase UPLC of **Butyl T3** (UV absorbance at 260 nm vs time in min).

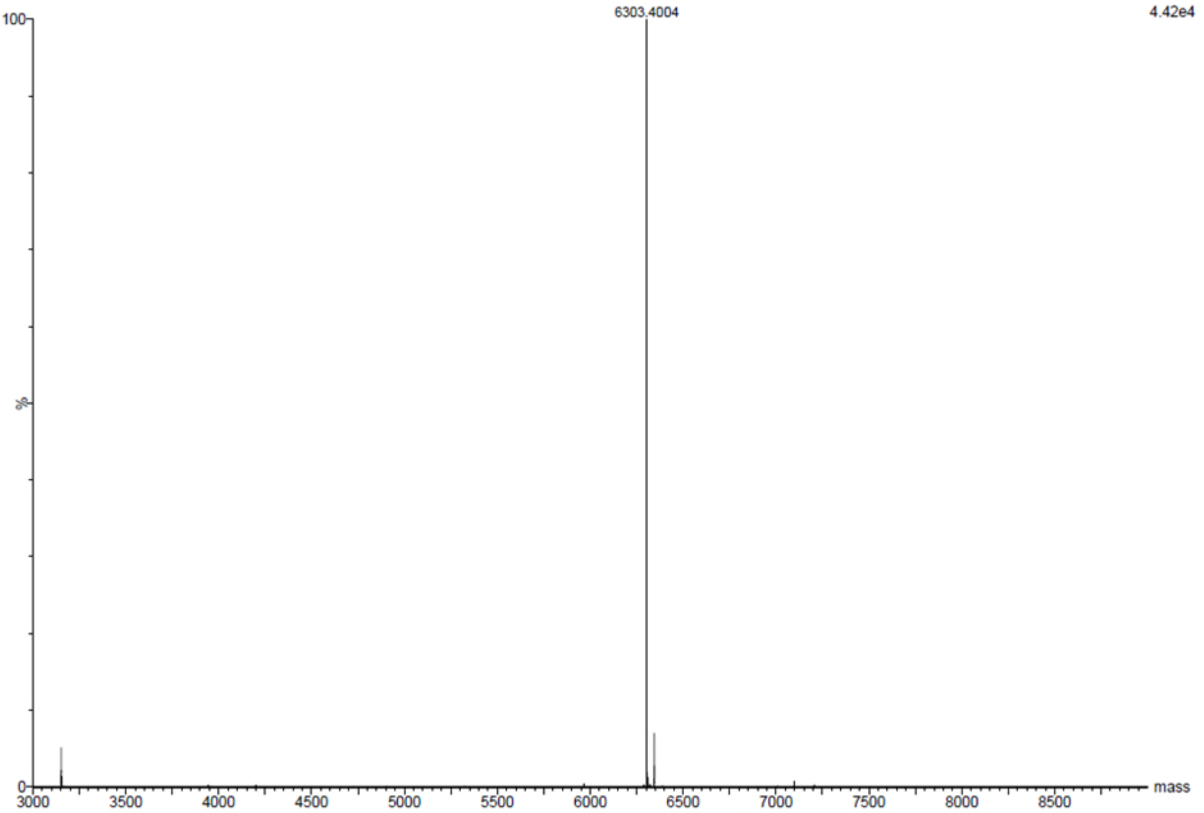

Supplementary Figure S6: Mass spectrum (ES-) of **Butyl T3**. Required **6303.23** Da, found **6303.40** Da. y-axis = relative intensity (%), x-axis = mass in Da.

Butyl T4	CCT CUT ACC TCA GUT ACA	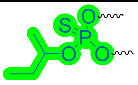

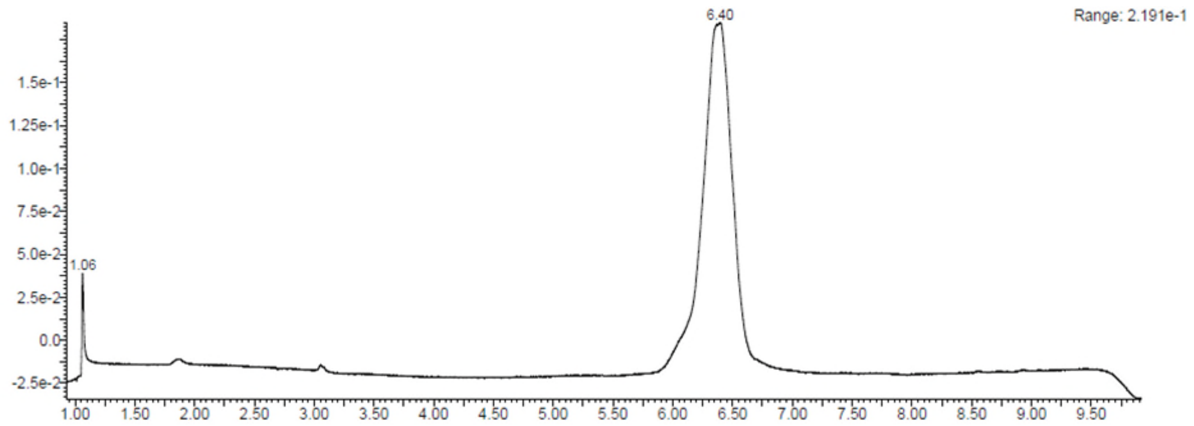

Supplementary Figure S7: Reverse-phase UPLC of **Butyl T4** (UV absorbance at 260 nm vs time in min).

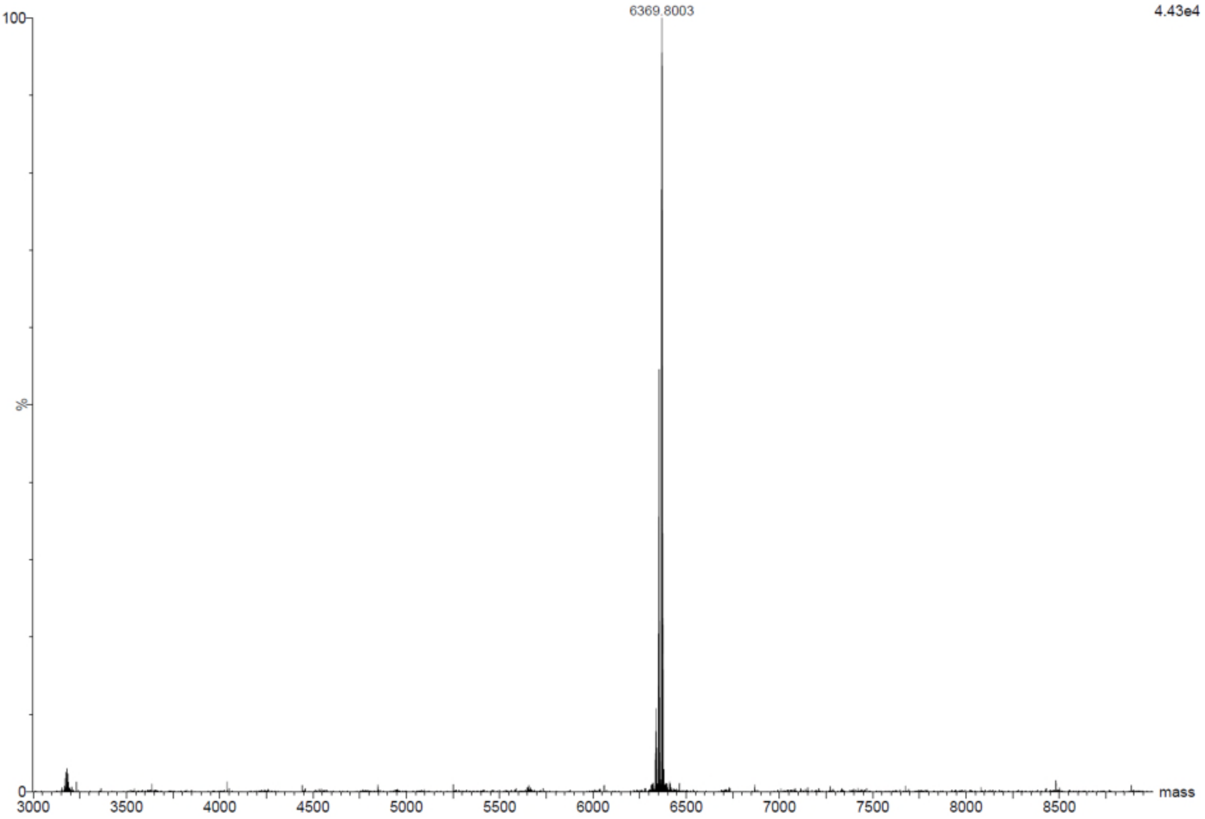

Supplementary Figure S8: Mass spectrum (ES-) of **Butyl T4**. Required **6371.35** Da, found **6369.80** Da. y-axis = relative intensity (%), x-axis = mass in Da.

2.5.2 C16 A1, C16 A2, C16 A2b and C16 A3

C16 A1	CCU CUU ACC UCA GUU ACA	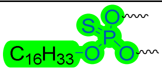

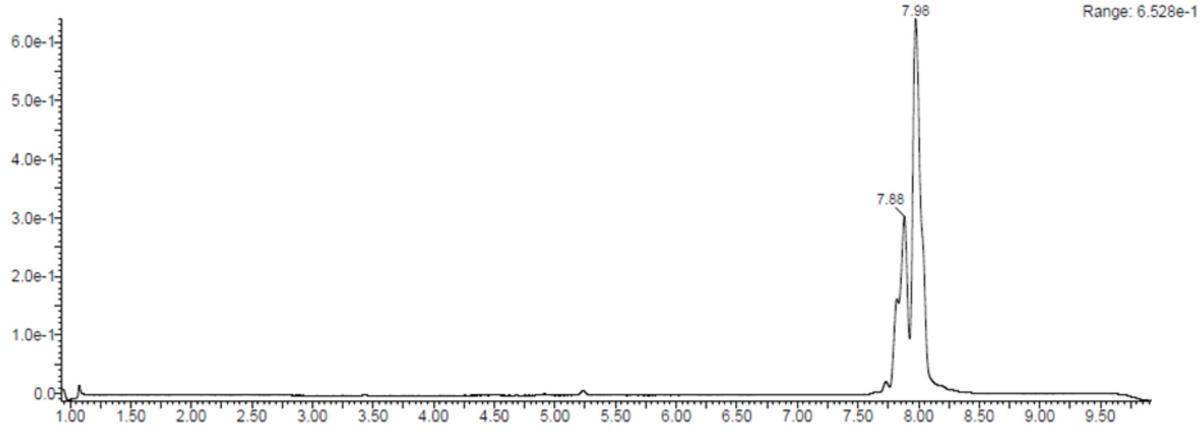

Supplementary Figure S9: Reverse-phase UPLC of **C16 A1** (UV absorbance at 260 nm vs time in min).

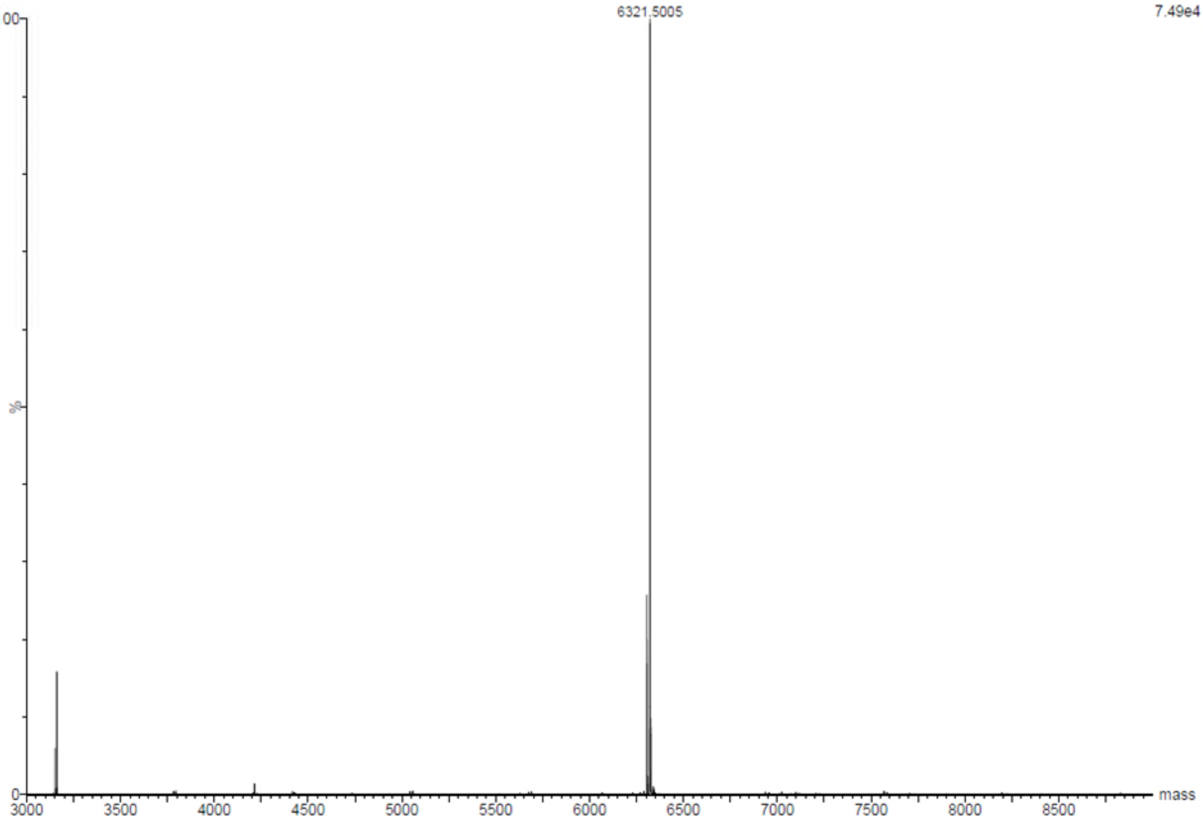

Supplementary Figure S10: Mass spectrum (ES-) of **C16 A1**. Required **6321.29** Da, found **6321.50** Da. y-axis = relative intensity (%), x-axis = mass in Da.

C16 A2	CCU CUU ACC UCA GUU ACA	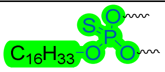

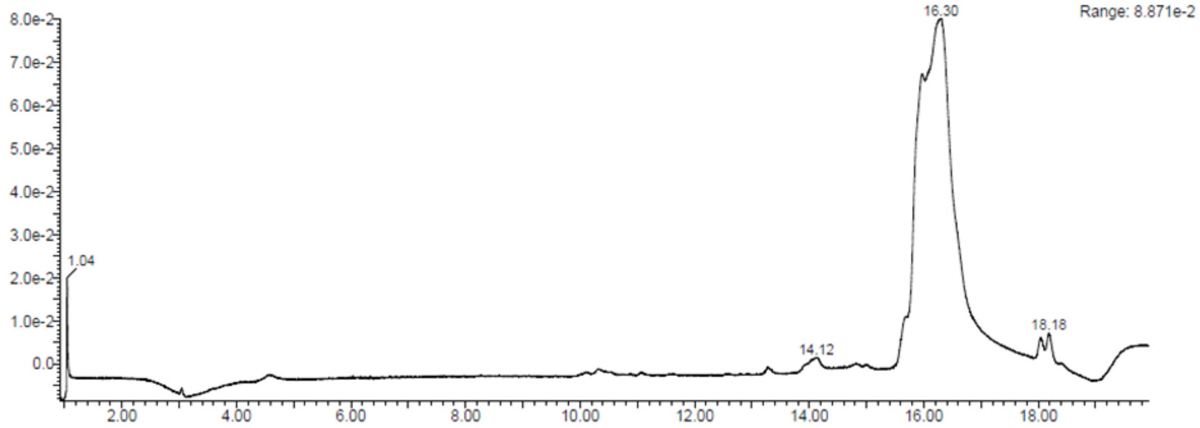

Supplementary Figure S11: Reverse-phase UPLC of **C16 A2** (UV absorbance at 260 nm vs time in min).

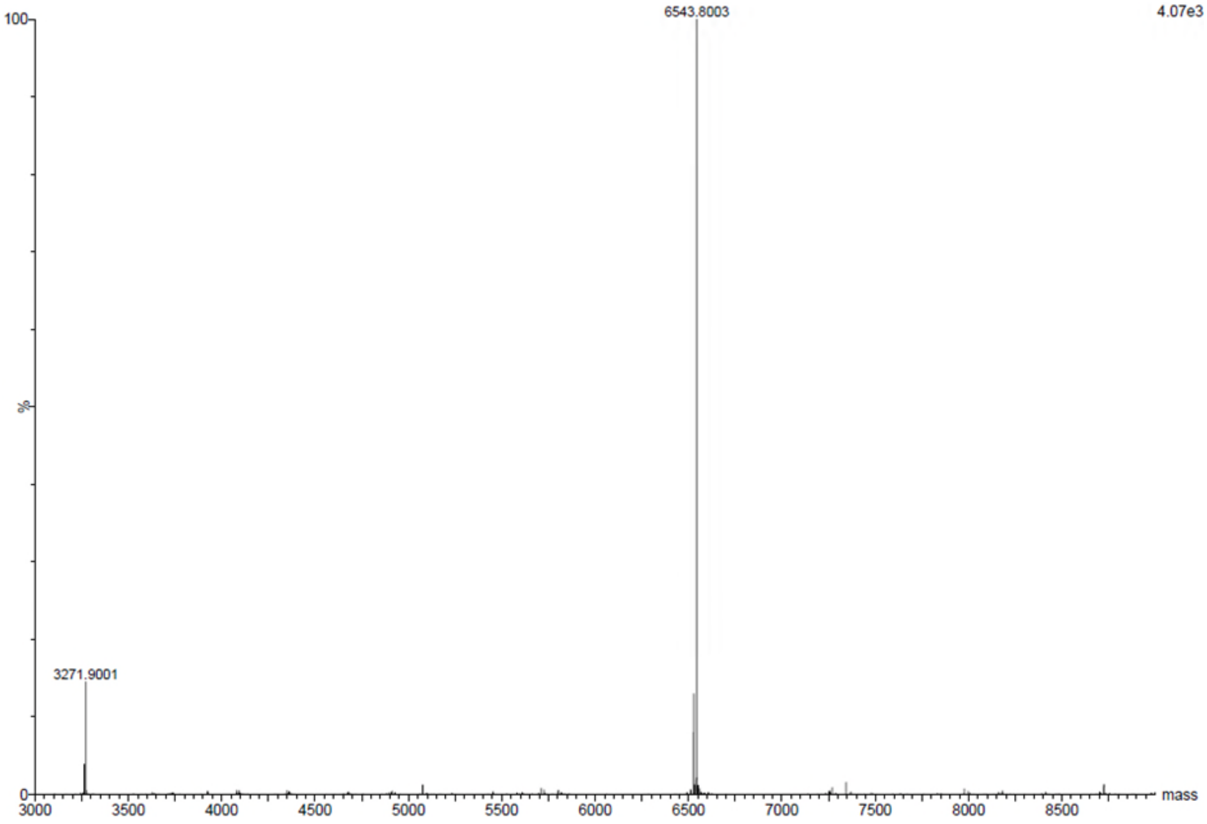

Supplementary Figure S12: Mass spectrum (ES-) of **C16 A2**. Required **6543.71** Da, found **6543.80** Da. y-axis = relative intensity (%), x-axis = mass in Da.

C16 A2b	CCU CUU ACC UCA GUU ACA	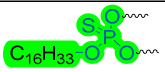

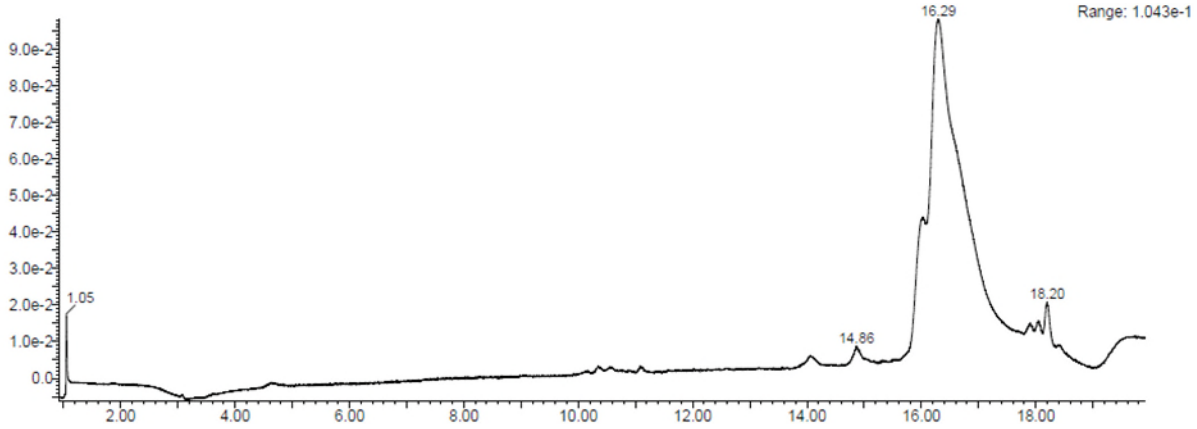

Supplementary Figure S13: Reverse-phase UPLC of **C16 A2b** (UV absorbance at 260 nm vs time in min).

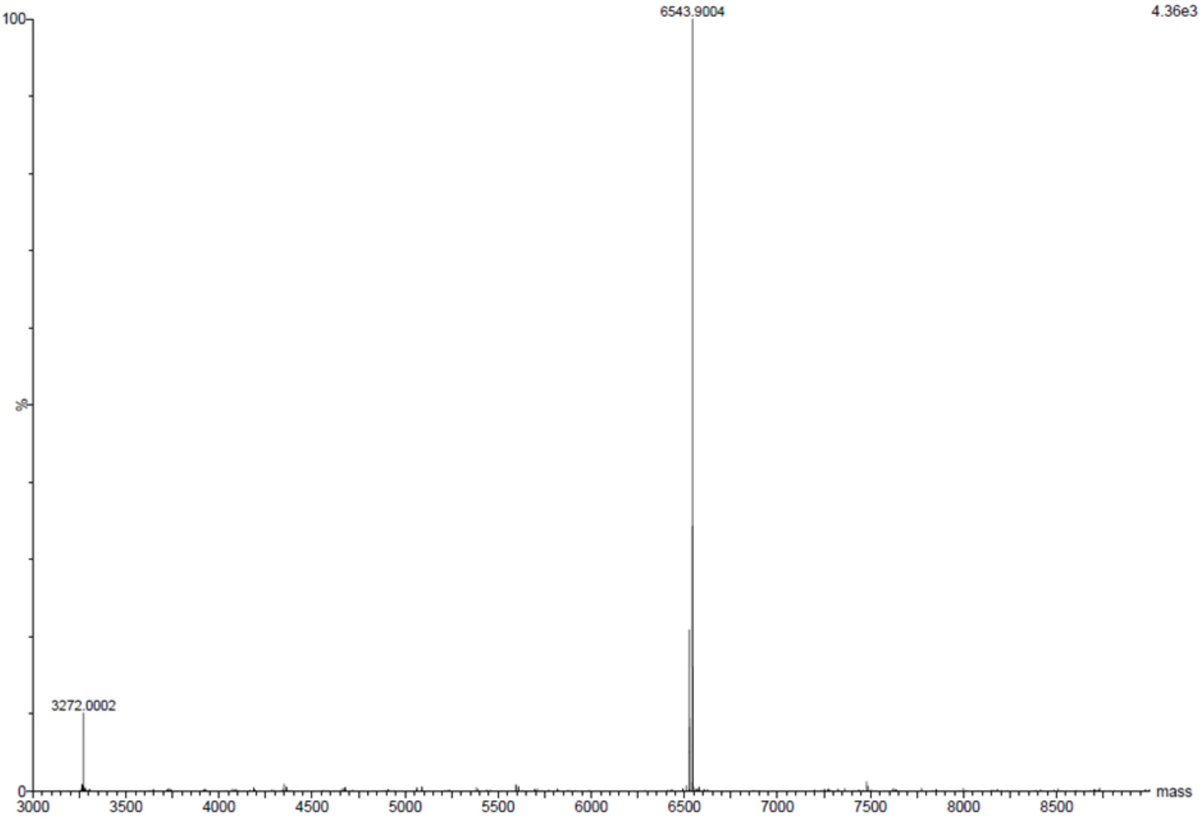

Supplementary Figure S14: Mass spectrum (ES-) of **C16 A2b**. Required **6543.71** Da, found **6543.90** Da. y-axis = relative intensity (%), x-axis = mass in Da.

C16 A3	CCU CUU ACC UCA GUU ACA	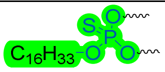

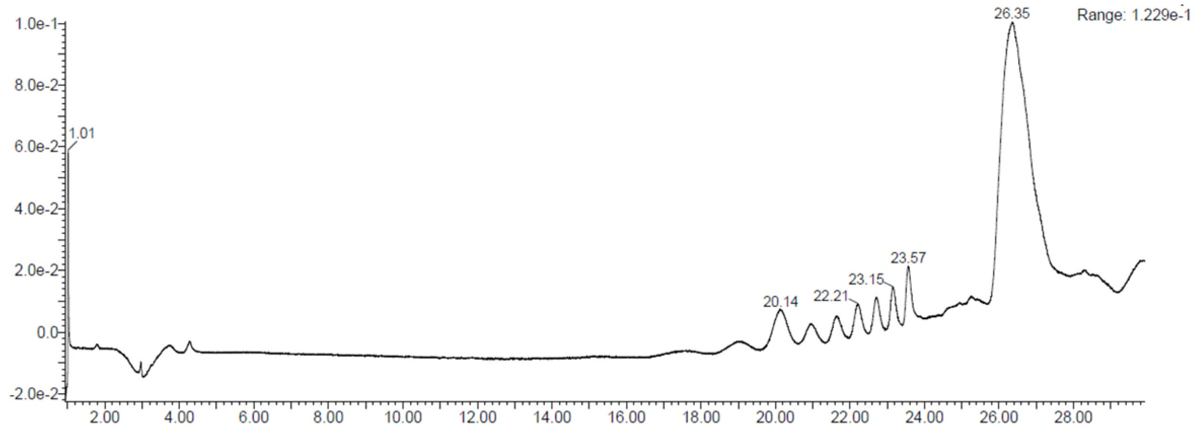

Supplementary Figure S15: Reverse-phase UPLC of **C16 A3** (UV absorbance at 260 nm vs time in min).

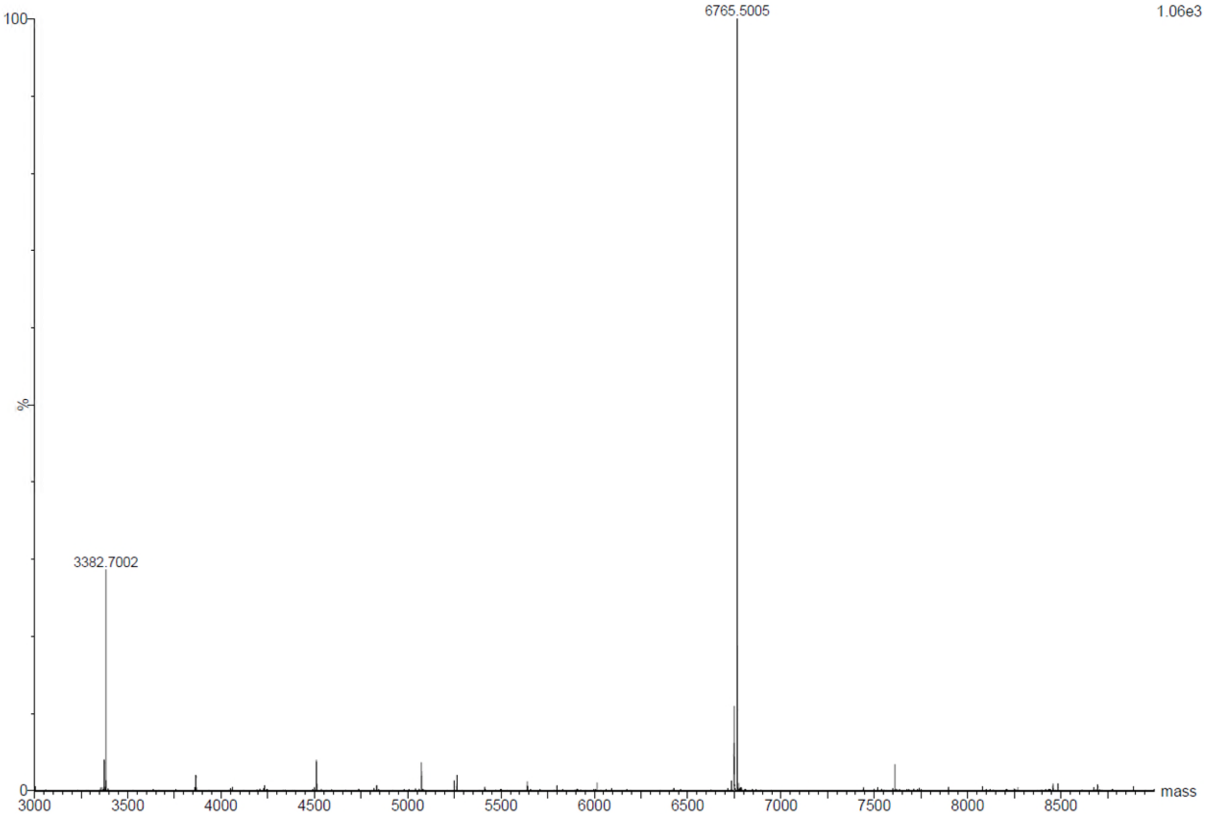

Supplementary Figure S16: Mass spectrum (ES-) of **C16 A3**. Required **6766.13** Da, found **6765.50** Da. y-axis = relative intensity (%), x-axis = mass in Da.

2.5.3 CF_3 T1, CF_3 T2, CF_3 T3 and CF_3 T4

CF_3 T1	CCU CUU ACC UCA GUT ACA	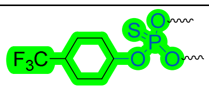

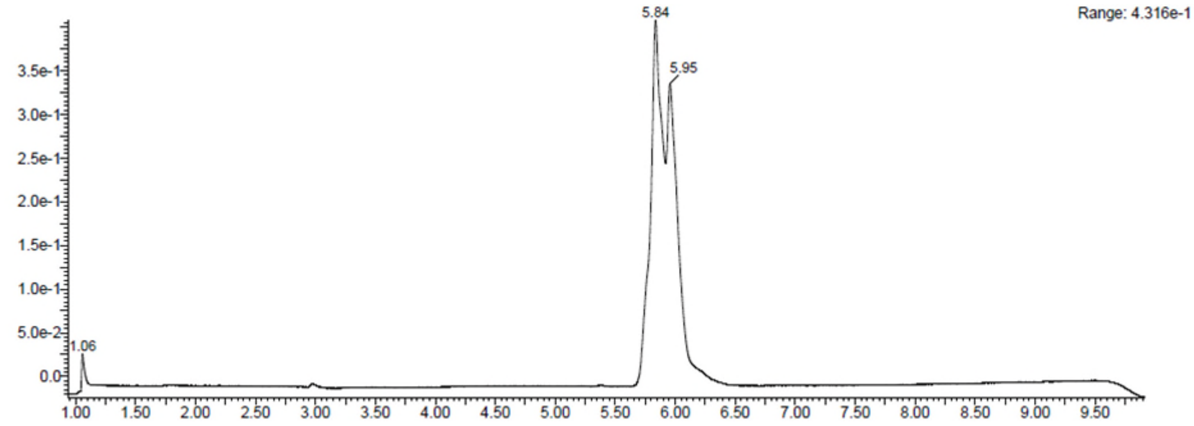

Supplementary Figure S17: Reverse-phase UPLC of **CF3 T1** (UV absorbance at 260 nm vs time in min).

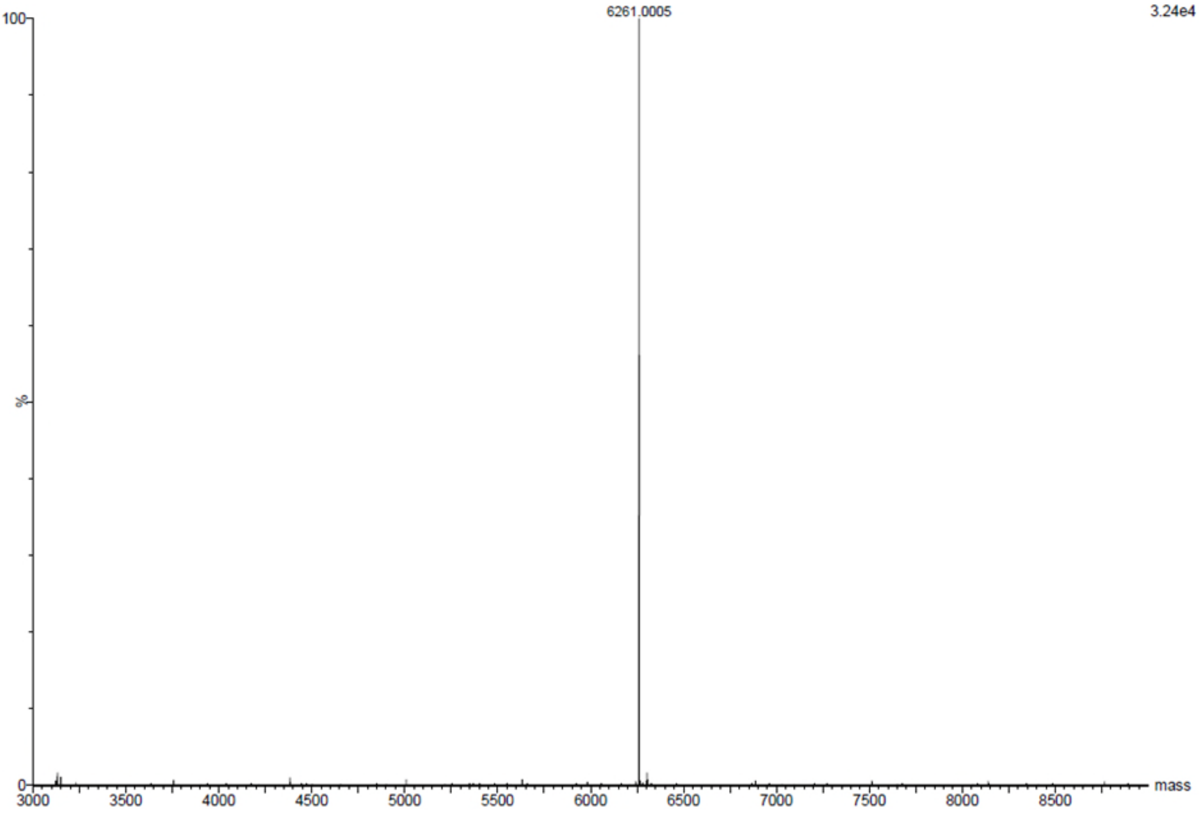

Supplementary Figure S18: Mass spectrum (ES-) of **CF3 T1**. Required **6261.03** Da, found **6261.00** Da. y-axis = relative intensity (%), x-axis = mass in Da.

CF_3 T2	CCU CUT ACC UCA GUT ACA	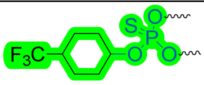

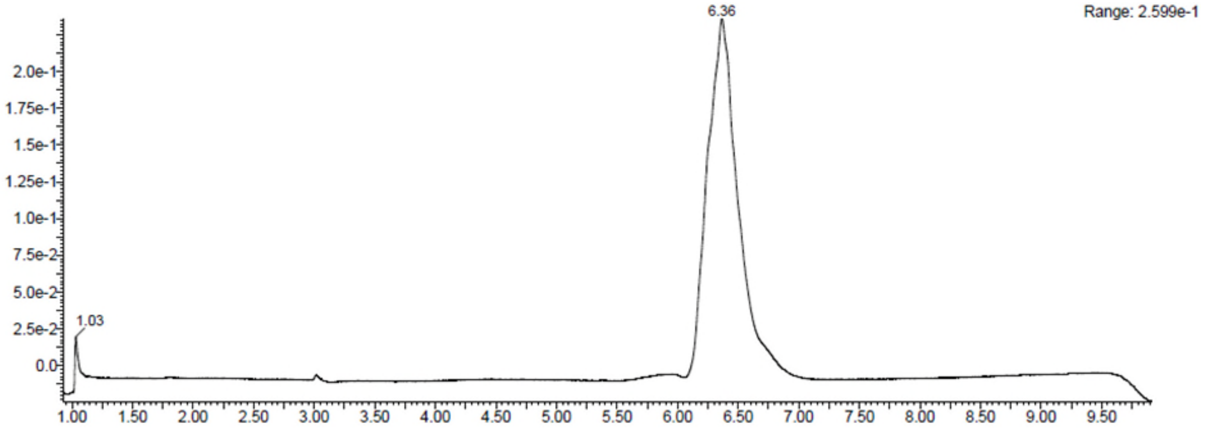

Supplementary Figure S19: Reverse-phase UPLC of **CF3 T2** (UV absorbance at 260 nm vs time in min).

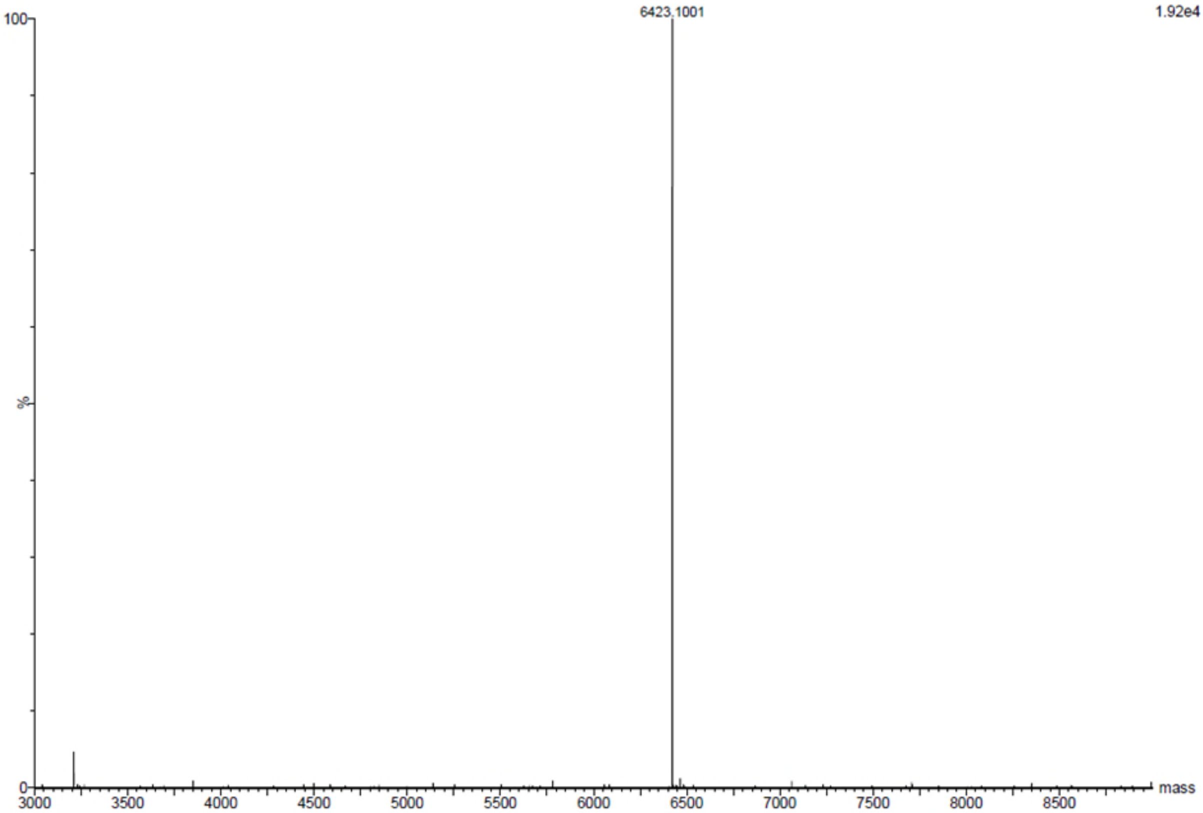

Supplementary Figure S20: Mass spectrum (ES-) of **CF3 T2**. Required **6423.19** Da, found **6423.10** Da. y-axis = relative intensity (%), x-axis = mass in Da.

CF_3 T3	CCU CUT ACC TCA GUT ACA	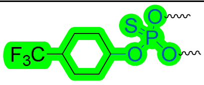

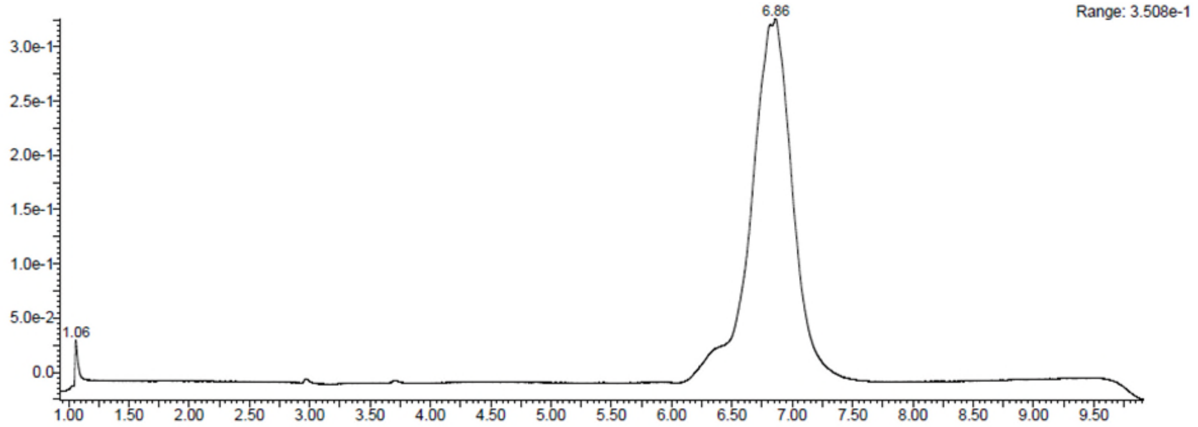

Supplementary Figure S21: Reverse-phase UPLC of **CF3 T3** (UV absorbance at 260 nm vs time in min).

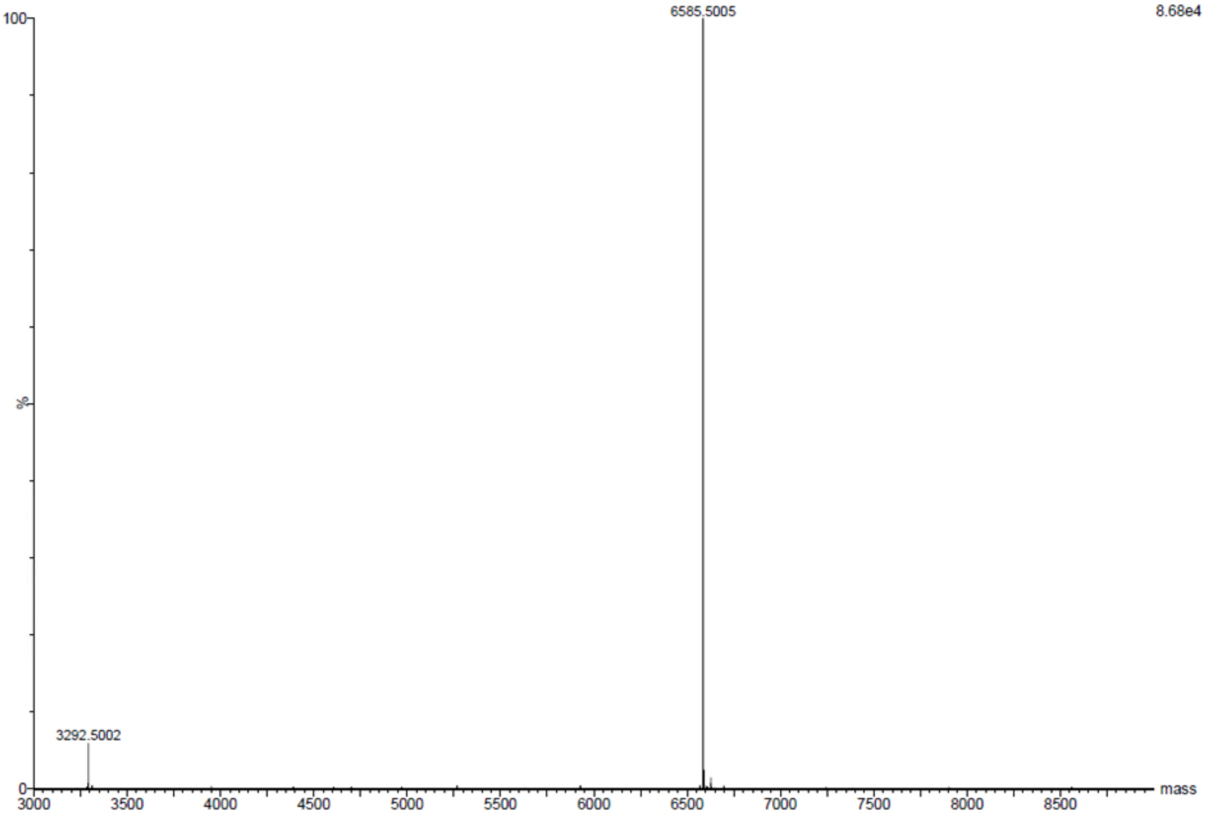

Supplementary Figure S22: Mass spectrum (ES-) of **CF3 T3**. Required **6585.34** Da, found **6585.50** Da. y-axis = relative intensity (%), x-axis = mass in Da.

CF_3 T4	CCT CUT ACC TCA GUT ACA	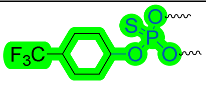

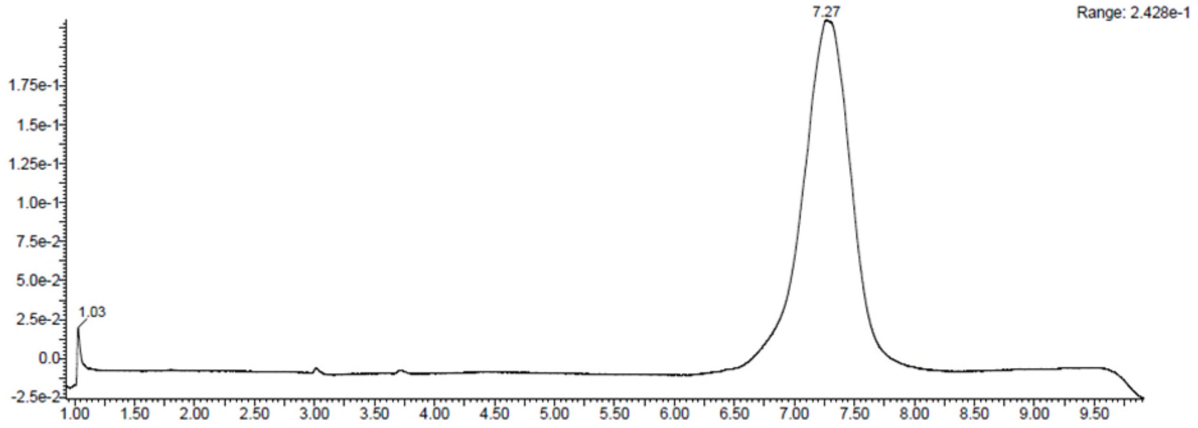

Supplementary Figure S23: Reverse-phase UPLC of **CF3 T4** (UV absorbance at 260 nm vs time in min).

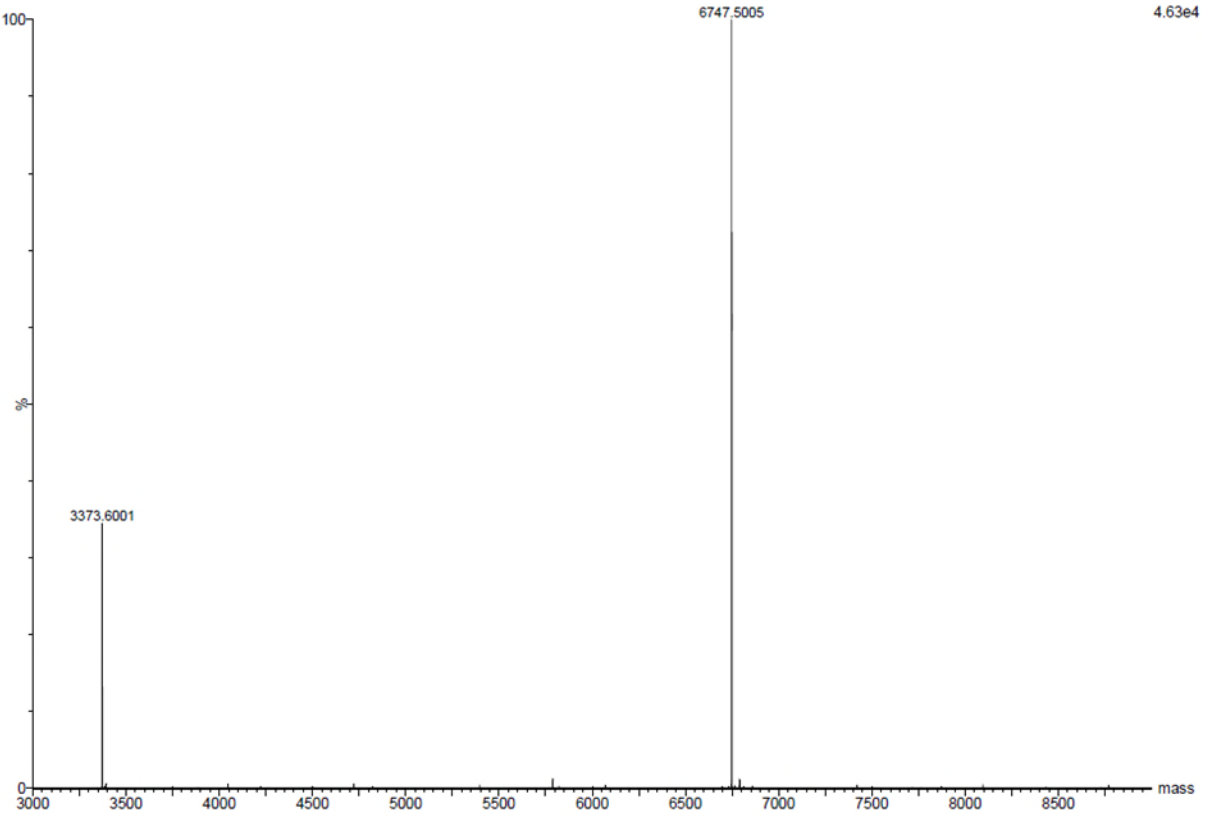

Supplementary Figure S24: Mass spectrum (ES-) of **CF3 T4**. Required **6747.50** Da, found **6747.50** Da. y-axis = relative intensity (%), x-axis = mass in Da.

2.5.4 Hexyl T2, Hexyl T3 and Hexyl T4

Hexyl T2	CCU CUT ACC UCA GUT ACA	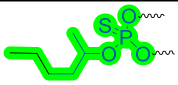

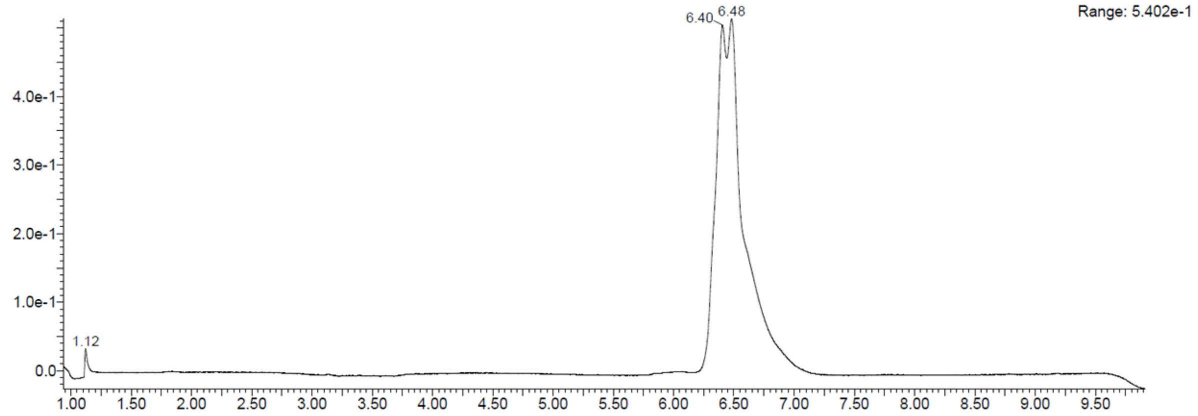

Supplementary Figure S25: Reverse-phase UPLC of **Hexyl T2** (UV absorbance at 260 nm vs time in min).

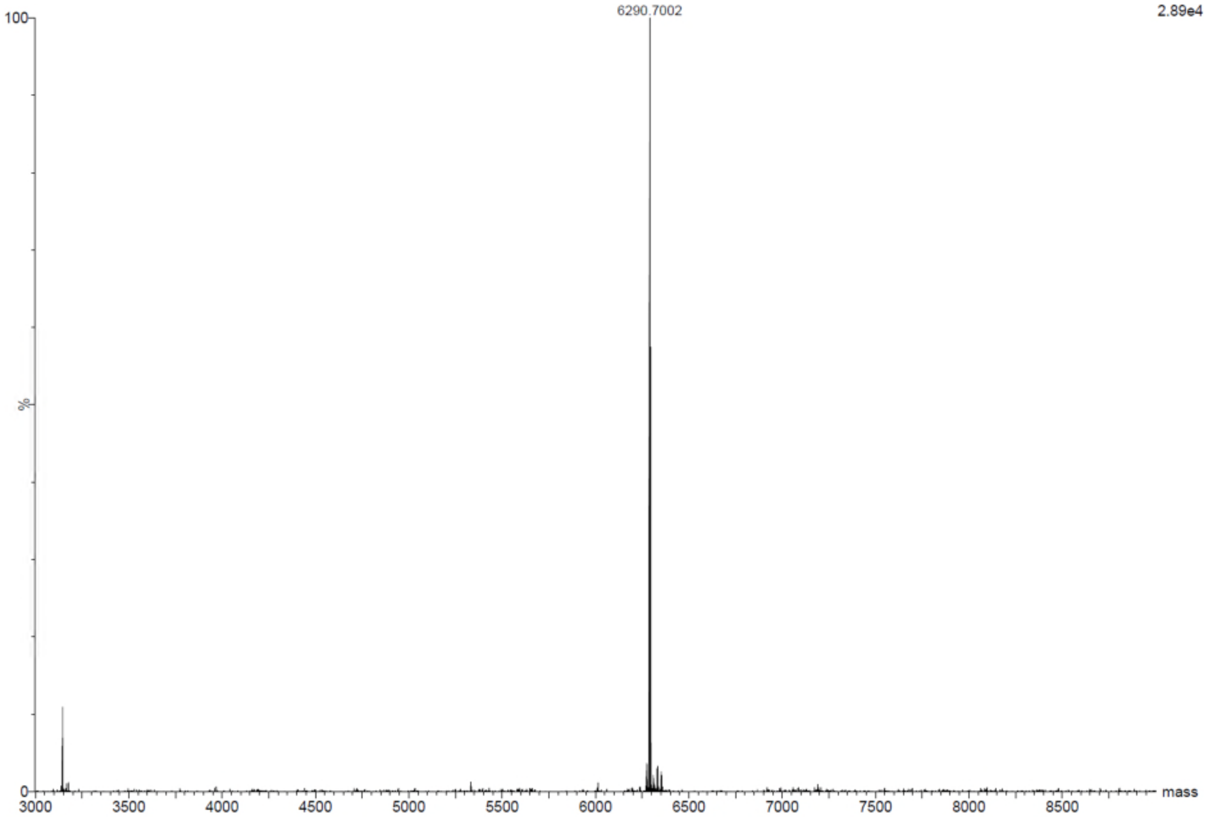

Supplementary Figure S26: Mass spectrum (ES-) of **Hexyl T2**. Required **6291.22** Da, found **6290.70** Da. y-axis = relative intensity (%), x-axis = mass in Da.

Hexyl T3	CCU CUT ACC TCA GUT ACA	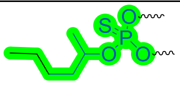

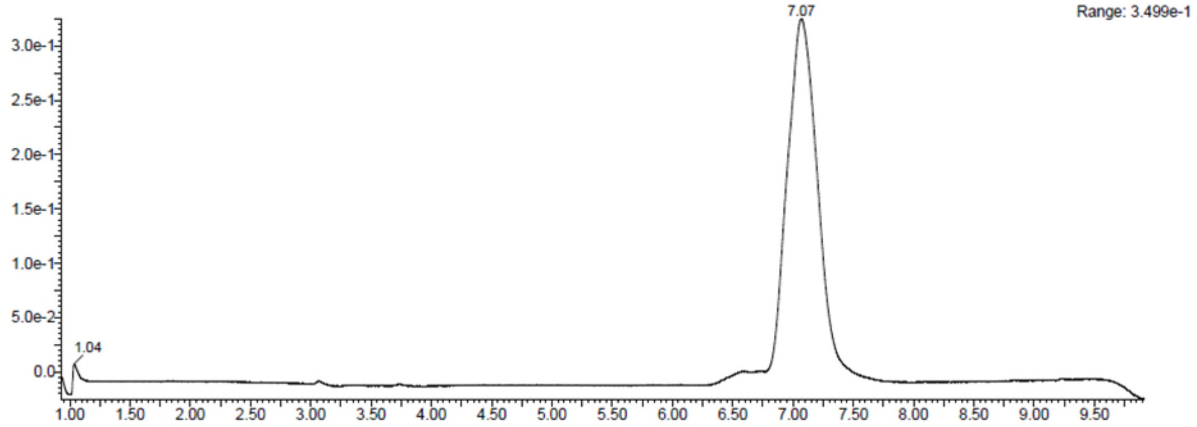

Supplementary Figure S27: Reverse-phase UPLC of **Hexyl T3** (UV absorbance at 260 nm vs time in min).

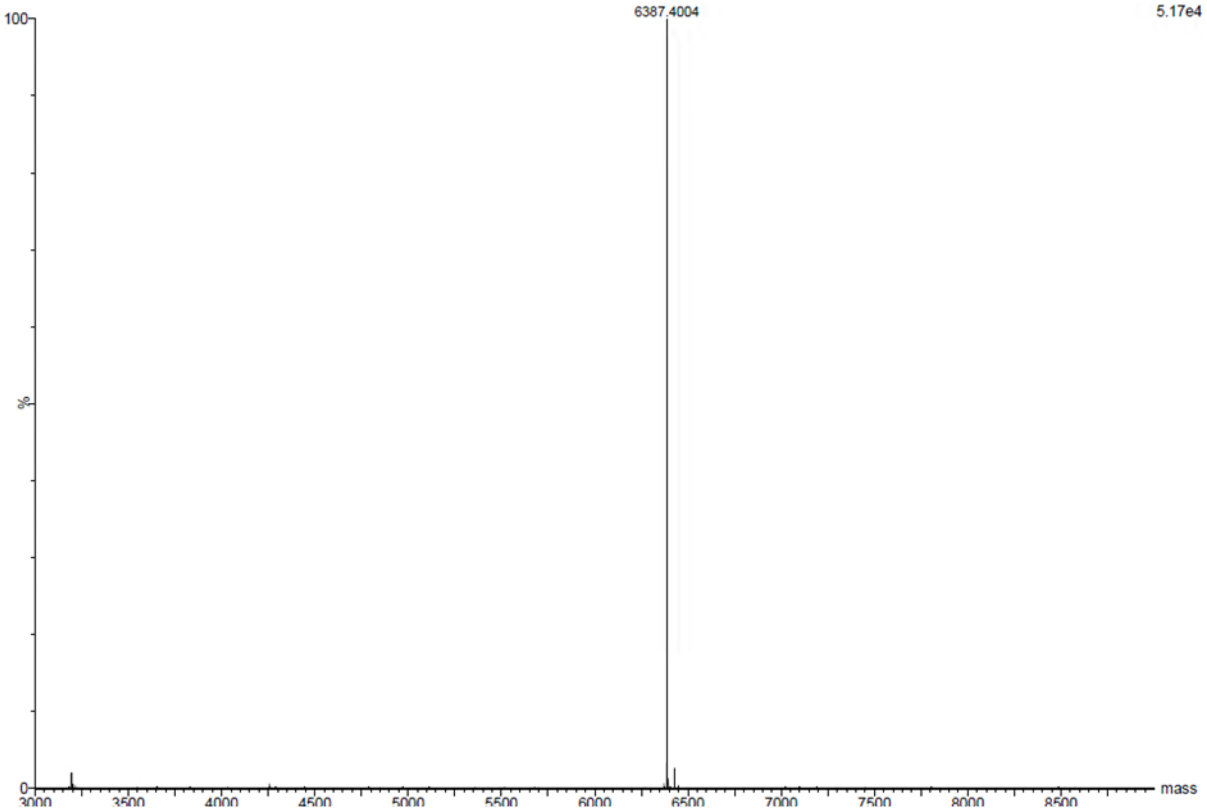

Supplementary Figure S28: Mass spectrum (ES-) of **Hexyl T3**. Required **6387.40** Da, found **6387.40** Da. y-axis = relative intensity (%), x-axis = mass in Da.

Hexyl T4	CCT CUT ACC TCA GUT ACA	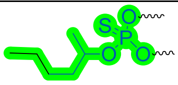

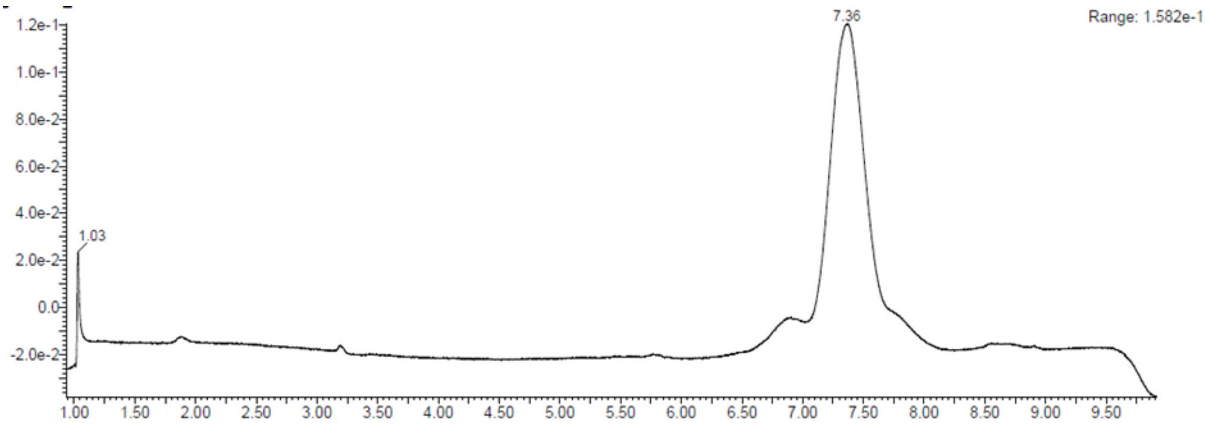

Supplementary Figure S29: Reverse-phase UPLC of **Hexyl T4** (UV absorbance at 260 nm vs time in min).

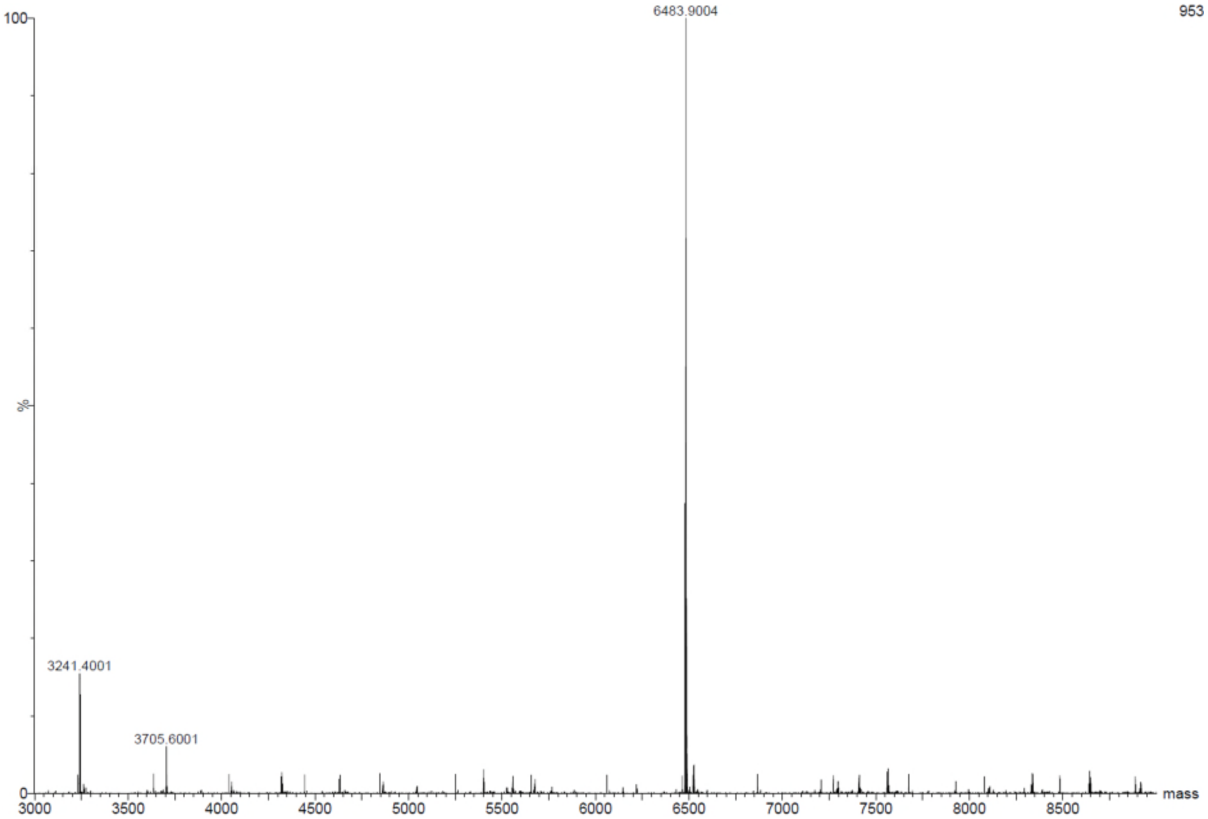

Supplementary Figure S30: Mass spectrum (ES-) of **Hexyl T4**. Required **6483.57** Da, found **6483.90** Da. y-axis = relative intensity (%), x-axis = mass in Da.

2.5.5 iPr A1, iPr A2, iPr A2b and iPr A3

iPr A1	CCU CUU ACC UCA GUU ACA	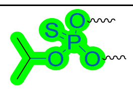

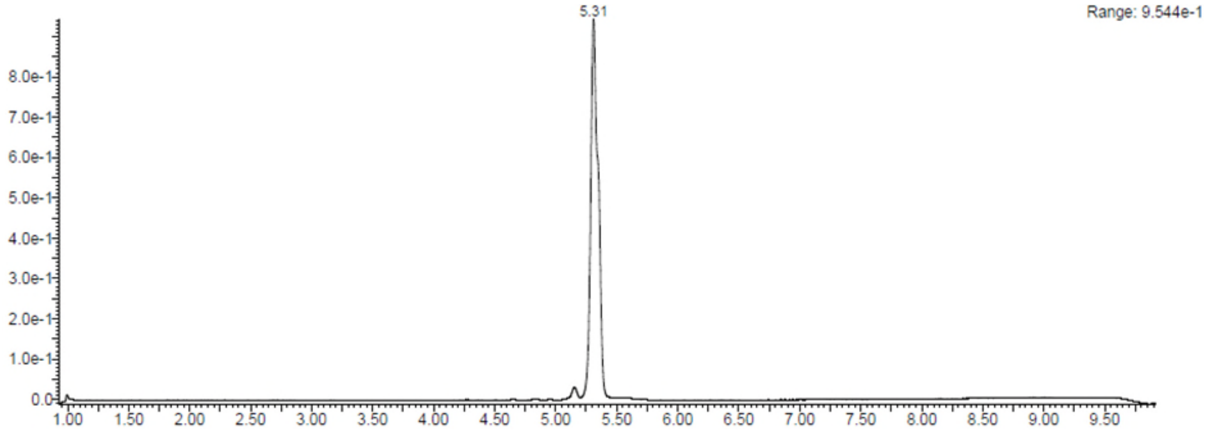

Supplementary Figure S31: Reverse-phase UPLC of **iPr A1** (UV absorbance at 260 nm vs time in min).

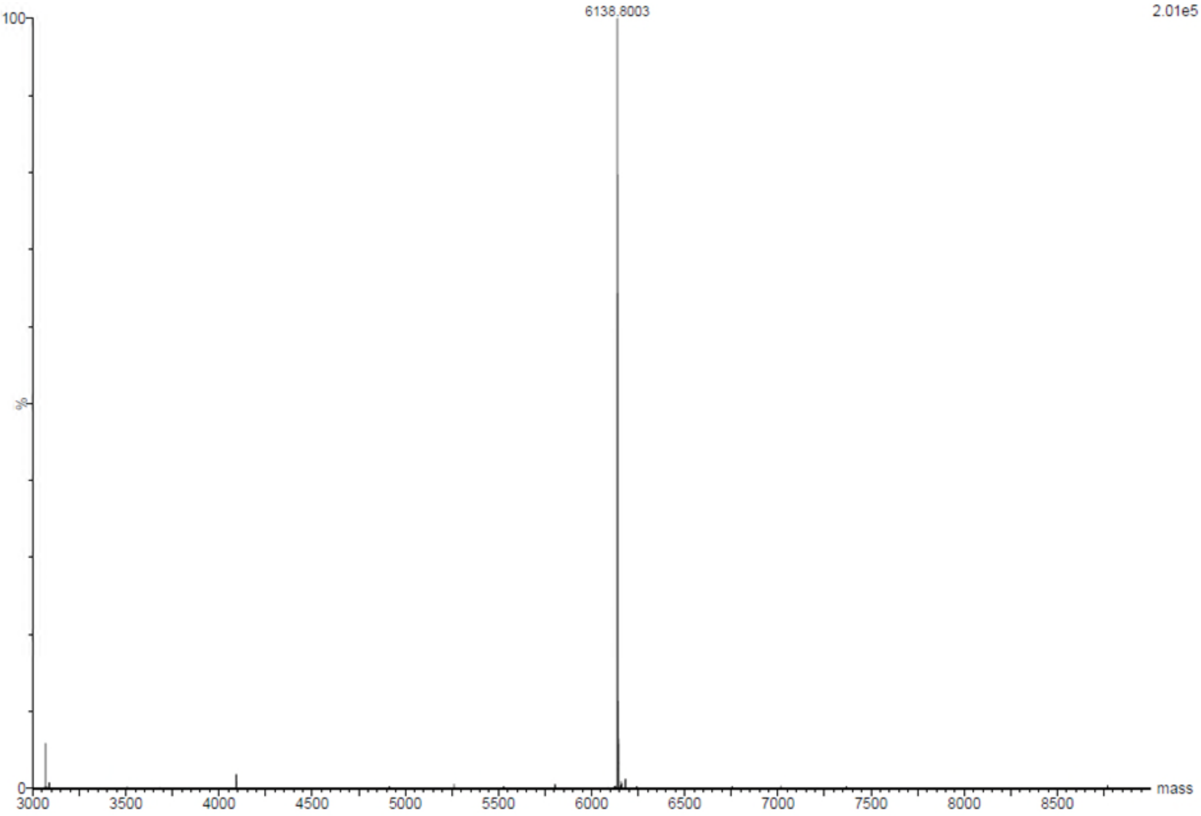

Supplementary Figure S32: Mass spectrum (ES-) of **iPr A1**. Required **6138.94** Da, found **6138.80** Da. y-axis = relative intensity (%), x-axis = mass in Da.

iPr A2	CCU CUU ACC UCA GUU ACA	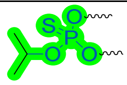

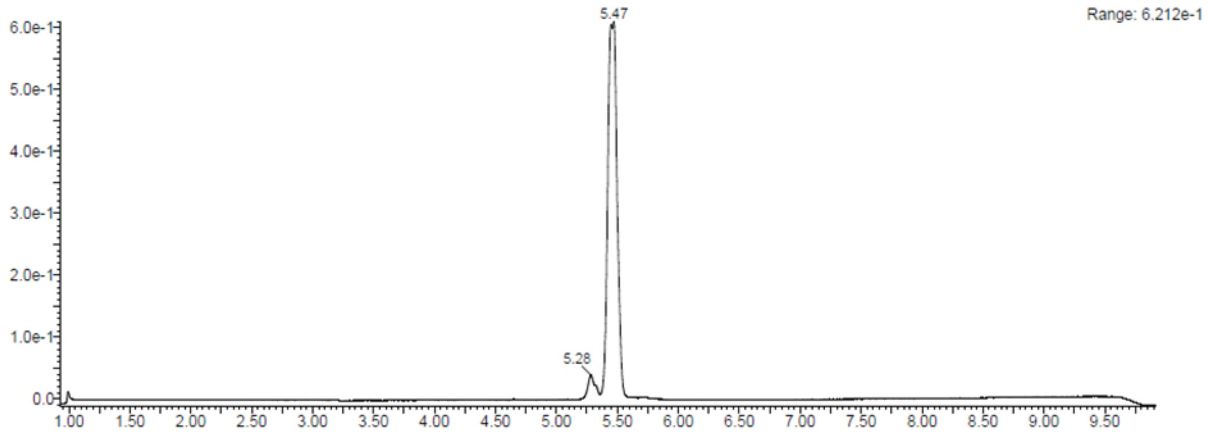

Supplementary Figure S33: Reverse-phase UPLC of **iPr A2** (UV absorbance at 260 nm vs time in min).

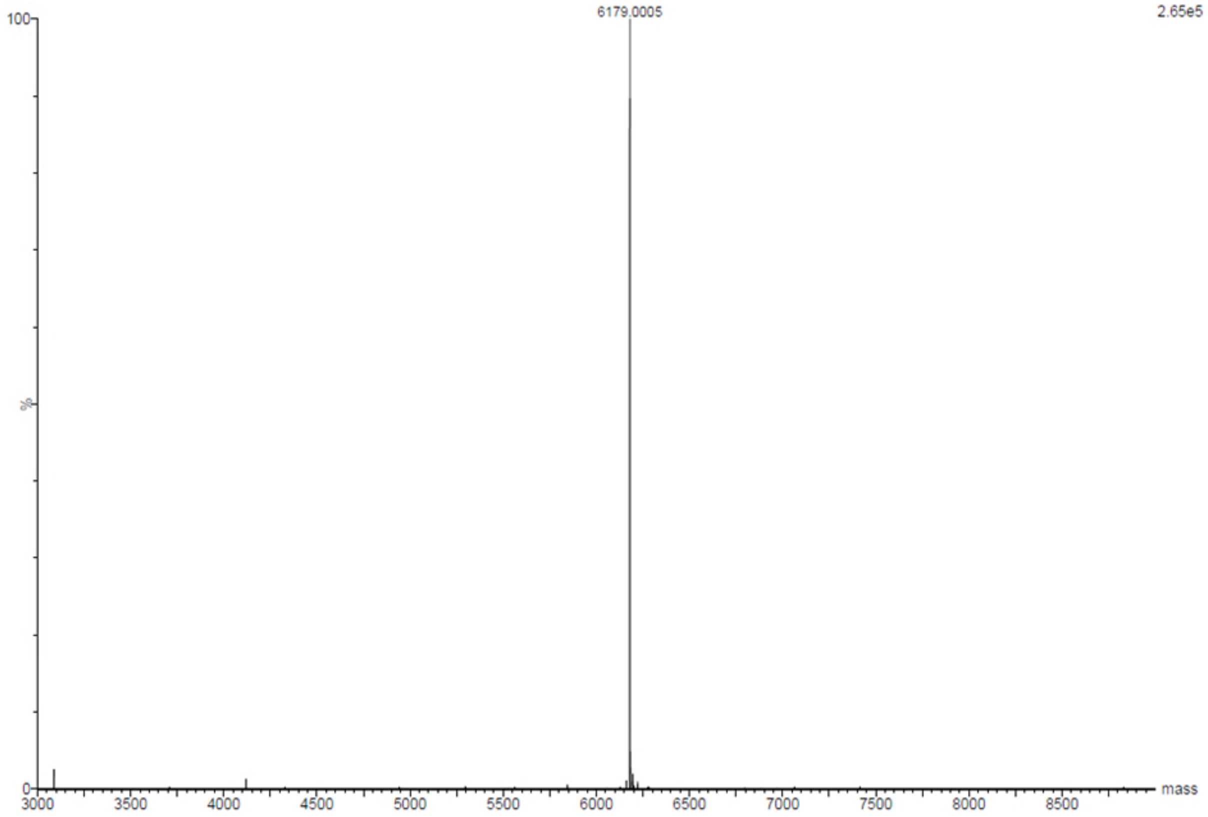

Supplementary Figure S34: Mass spectrum (ES-) of **iPr A2**. Required **6179.01** Da, found **6179.00** Da. y-axis = relative intensity (%), x-axis = mass in Da.

iPr A2b	CCU CUU ACC UCA GUU ACA	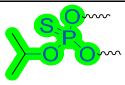

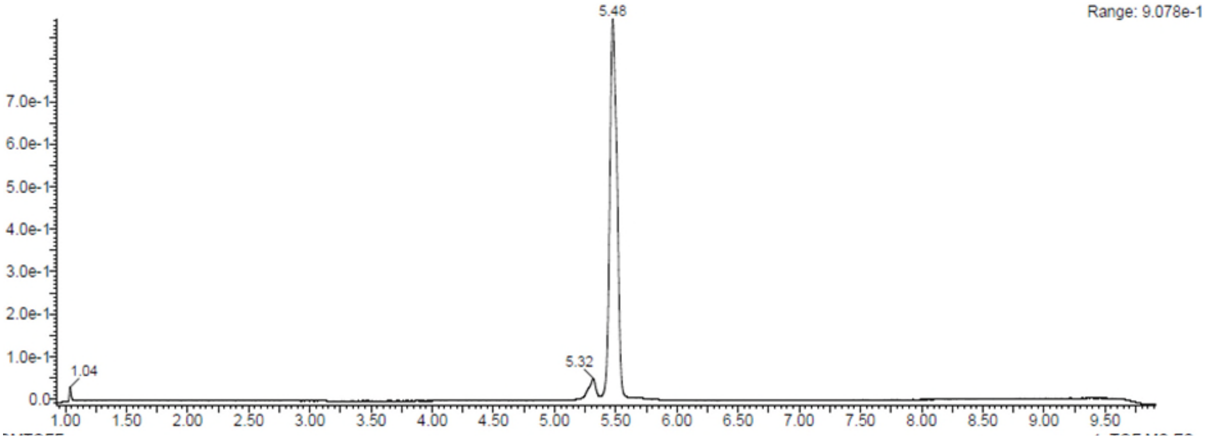

Supplementary Figure S35: Reverse-phase UPLC of **iPr A2b** (UV absorbance at 260 nm vs time in min).

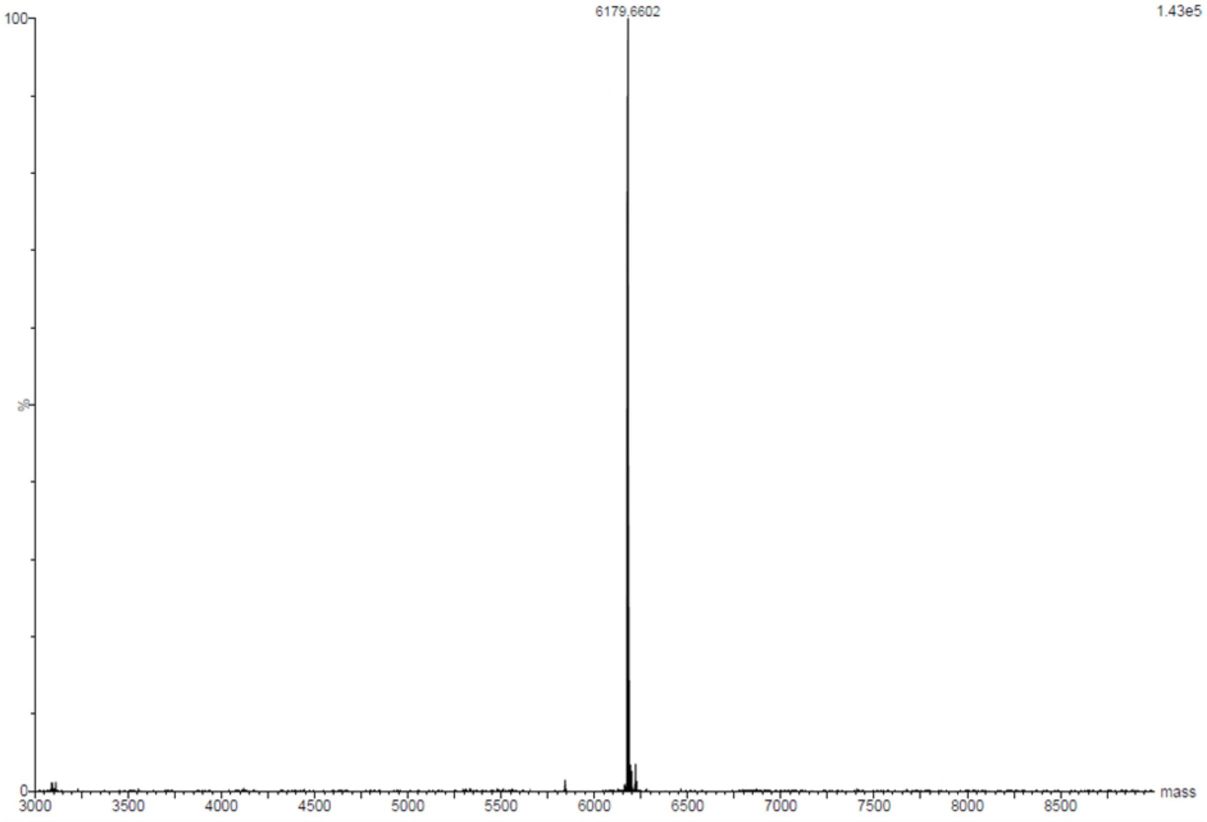

Supplementary Figure S36: Mass spectrum (ES-) of **iPr A2b**. Required **6179.01** Da, found **6179.66** Da. y-axis = relative intensity (%), x-axis = mass in Da.

iPr A3	CCU CUU ACC UCA GUU ACA	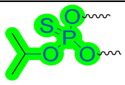

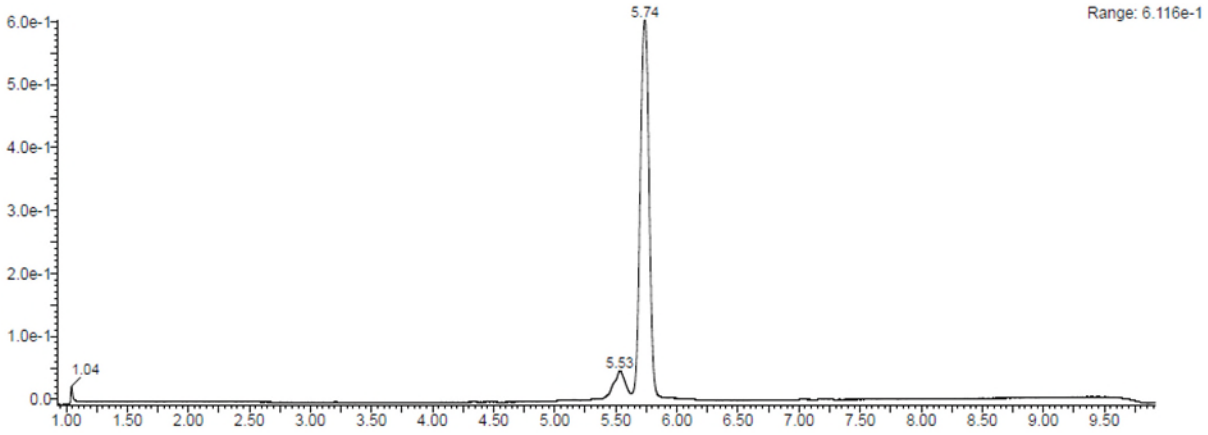

Supplementary Figure S37: Reverse-phase UPLC of **iPr A3** (UV absorbance at 260 nm vs time in min).

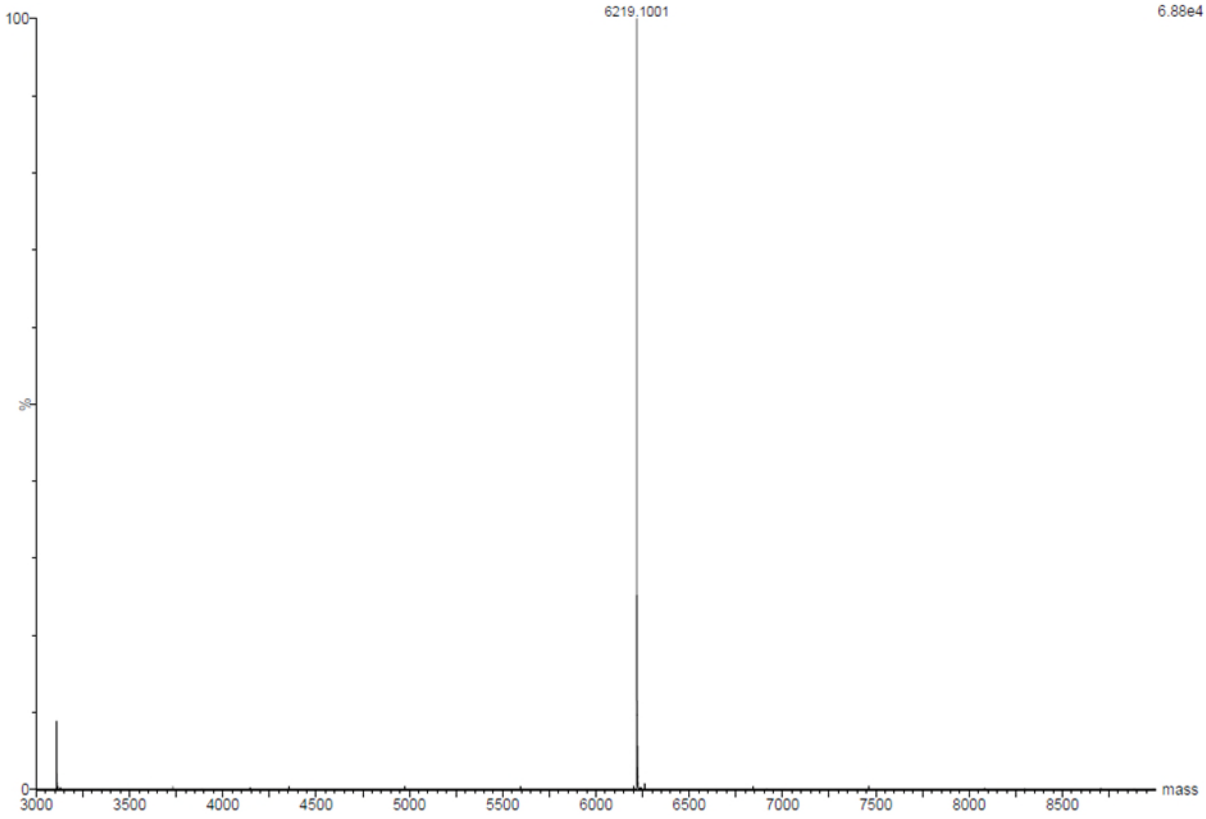

Supplementary Figure S38: Mass spectrum (ES-) of **iPr A3**. Required **6219.07** Da, found **6219.10** Da. y-axis = relative intensity (%), x-axis = mass in Da.

2.5.6 MOP A1, MOP A2, MOP A2b, MOP A3, MOP T1, MOP T2, MOP T3, MOP T4 and MOP T6

MOP A1	CCU CUU ACC UCA GUU ACA	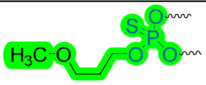

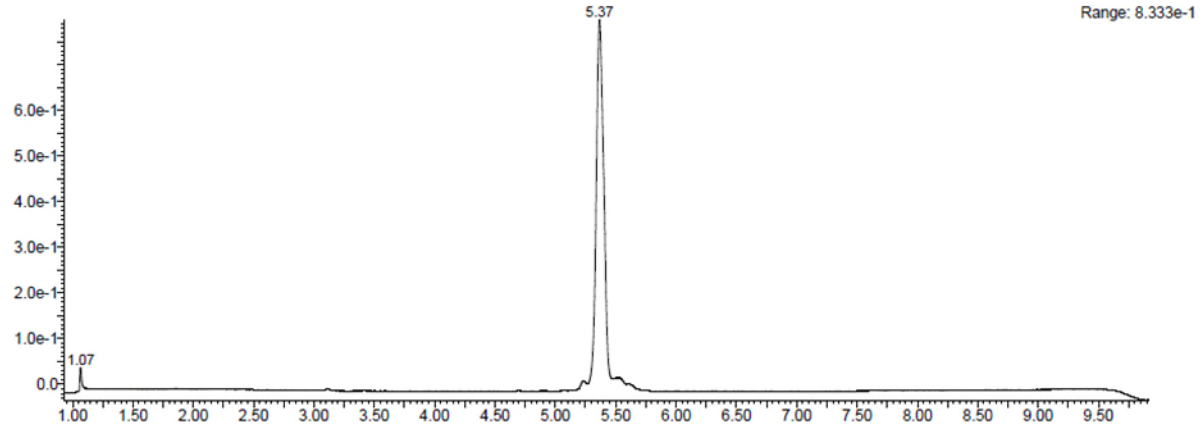

Supplementary Figure S39: Reverse-phase UPLC of **MOP A1** (UV absorbance at 260 nm vs time in min).

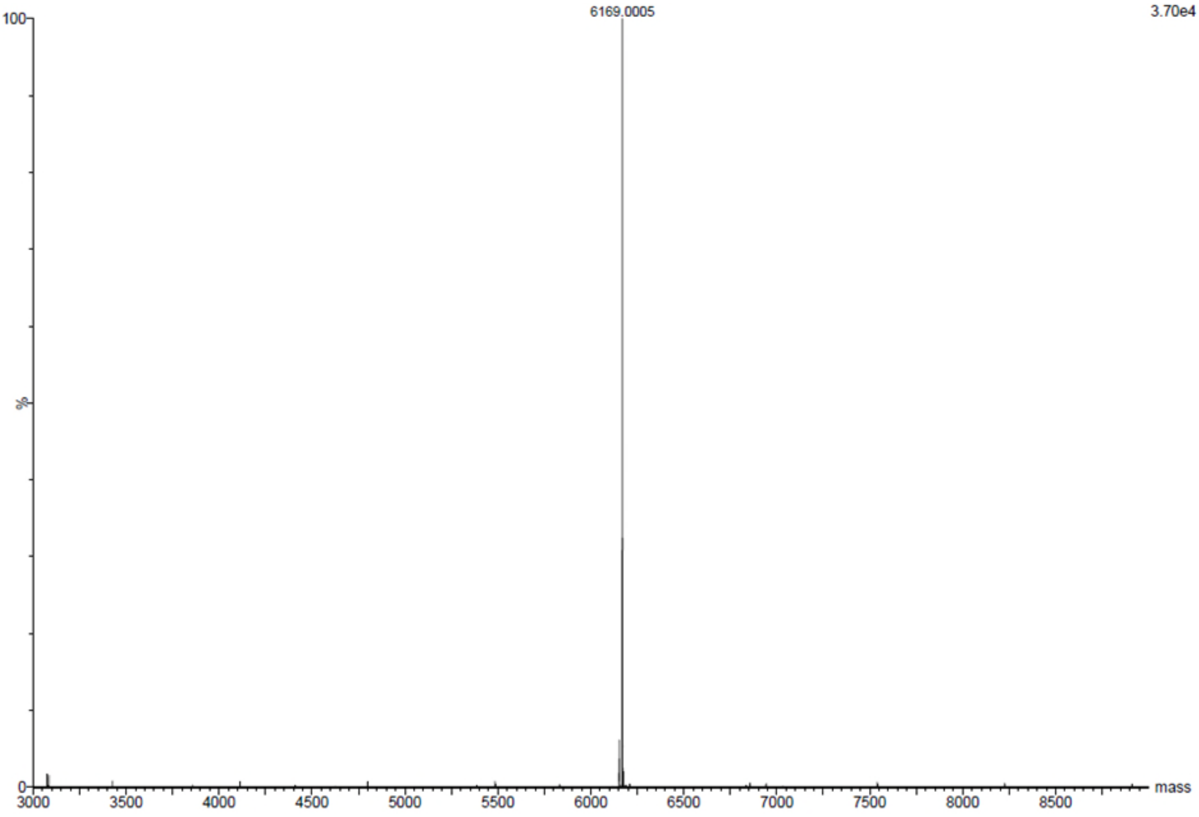

Supplementary Figure S40: Mass spectrum (ES-) of **MOP A1**. Required **6168.97** Da, found **6169.00** Da. y-axis = relative intensity (%), x-axis = mass in Da.

MOP A2	CCU CUU ACC UCA GUU ACA	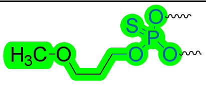

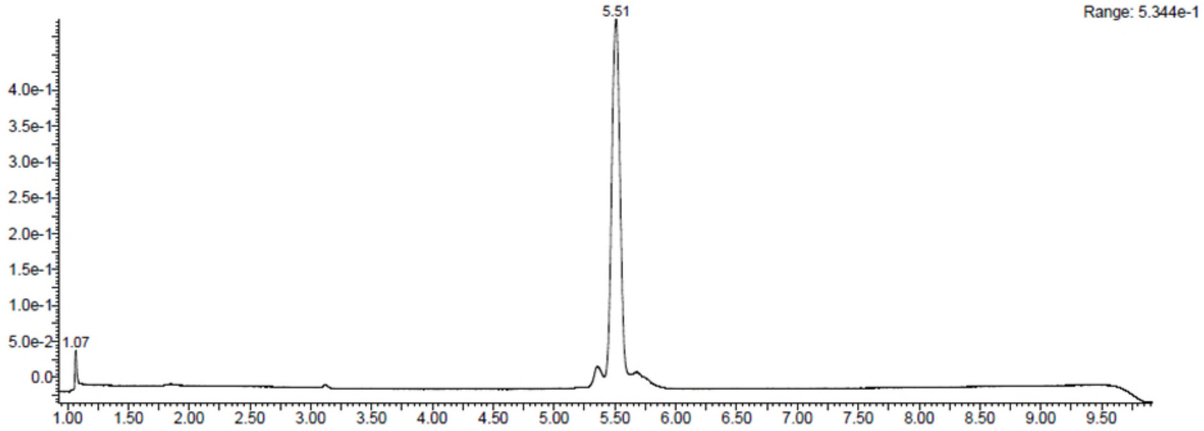

Supplementary Figure S41: Reverse-phase UPLC of **MOP A2** (UV absorbance at 260 nm vs time in min).

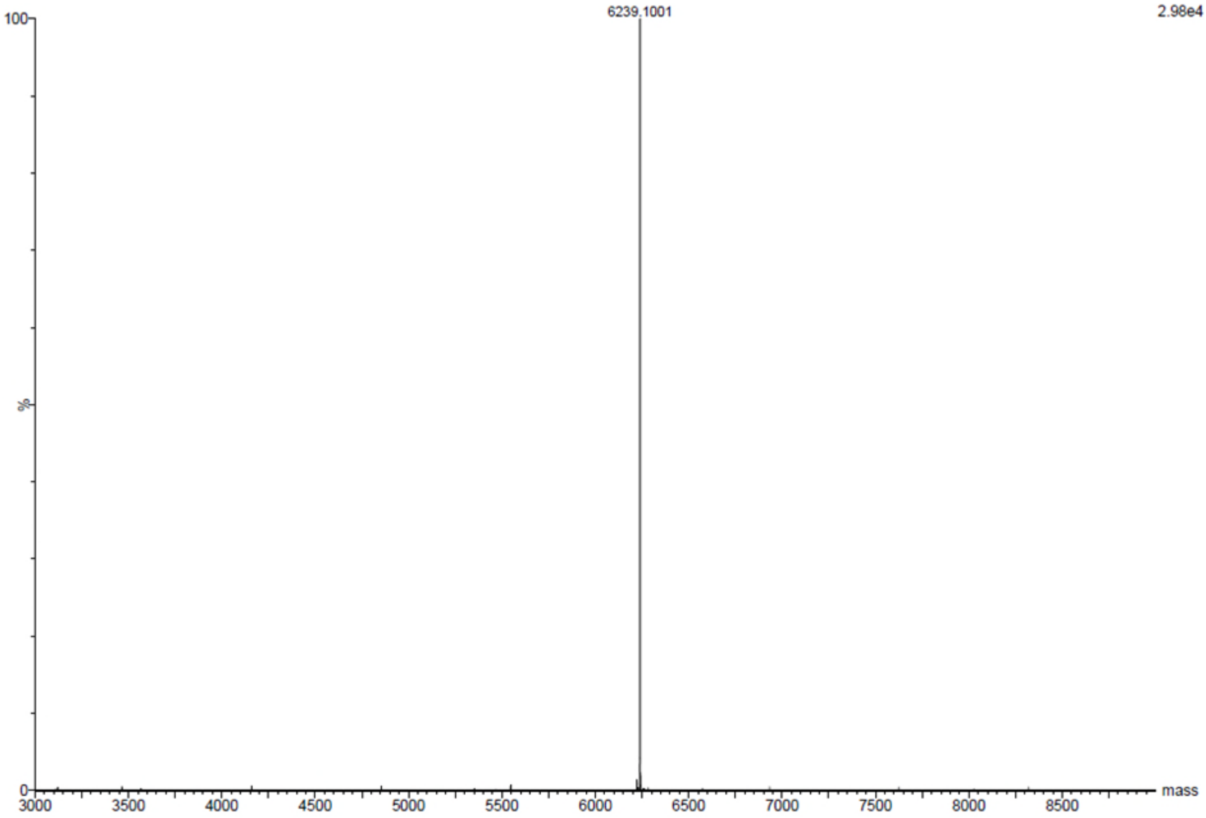

Supplementary Figure S42: Mass spectrum (ES-) of **MOP A2**. Required **6239.06** Da, found **6239.10** Da. y-axis = relative intensity (%), x-axis = mass in Da.

MOP A2b	CCU CUU ACC UCA GUU ACA	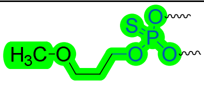

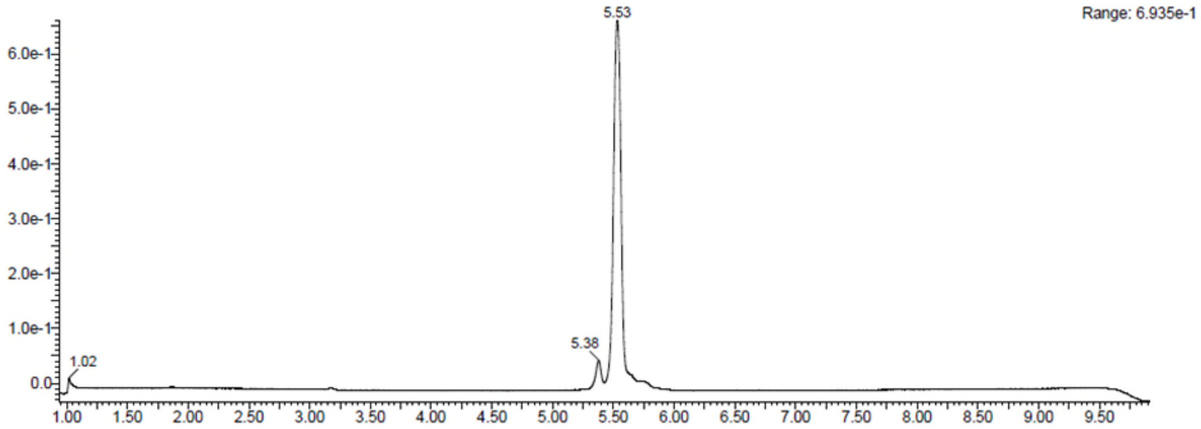

Supplementary Figure S43: Reverse-phase UPLC of **MOP A2** (UV absorbance at 260 nm vs time in min).

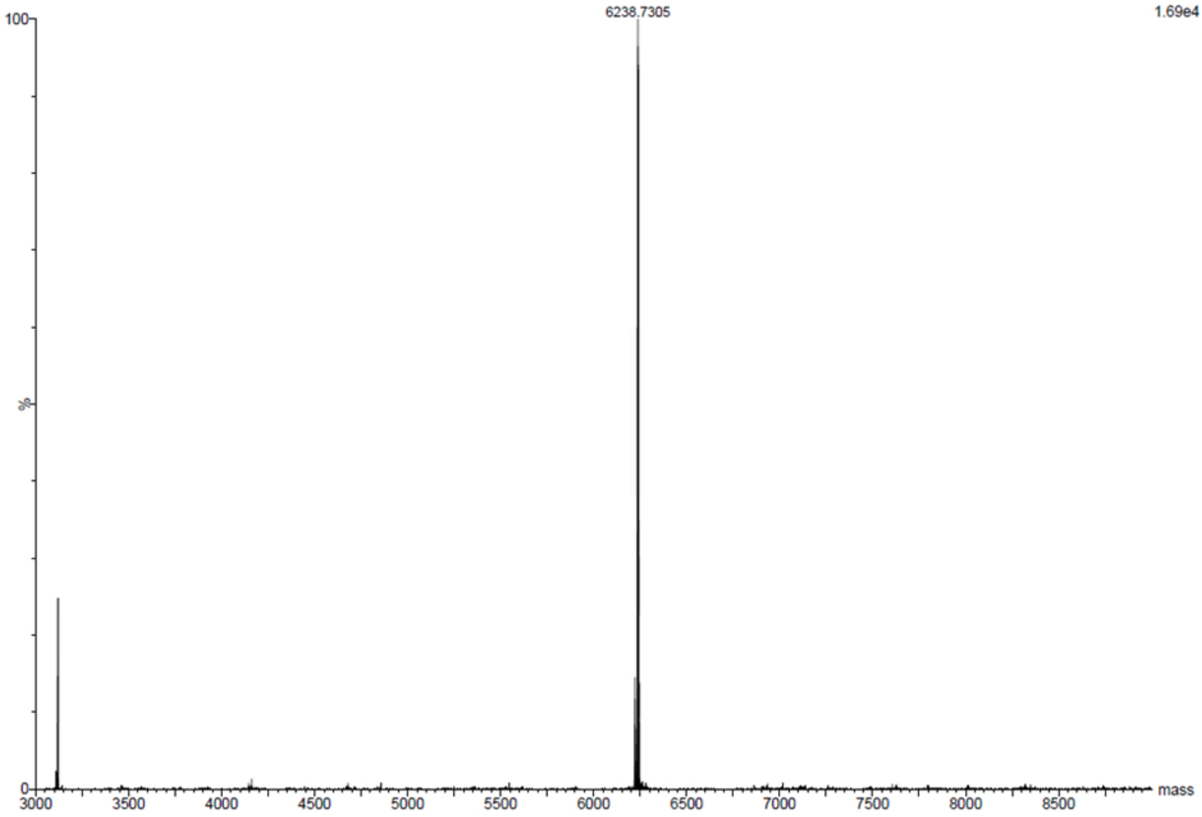

Supplementary Figure S44: Mass spectrum (ES-) of **MOP A2**. Required **6239.06** Da, found **6238.73** Da. y-axis = relative intensity (%), x-axis = mass in Da.

MOP A3	CCU CUU ACC UCA GUU ACA	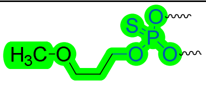

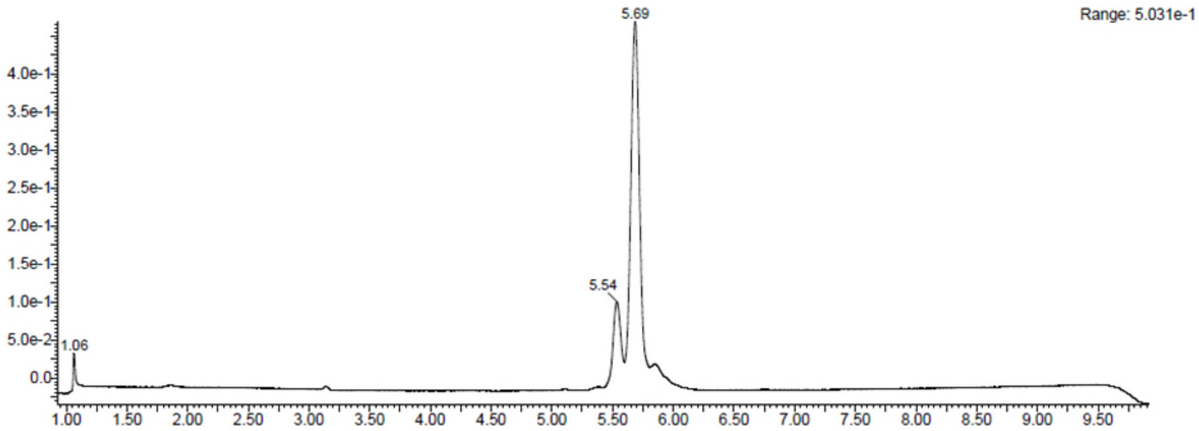

Supplementary Figure S45: Reverse-phase UPLC of **MOP A3** (UV absorbance at 260 nm vs time in min).

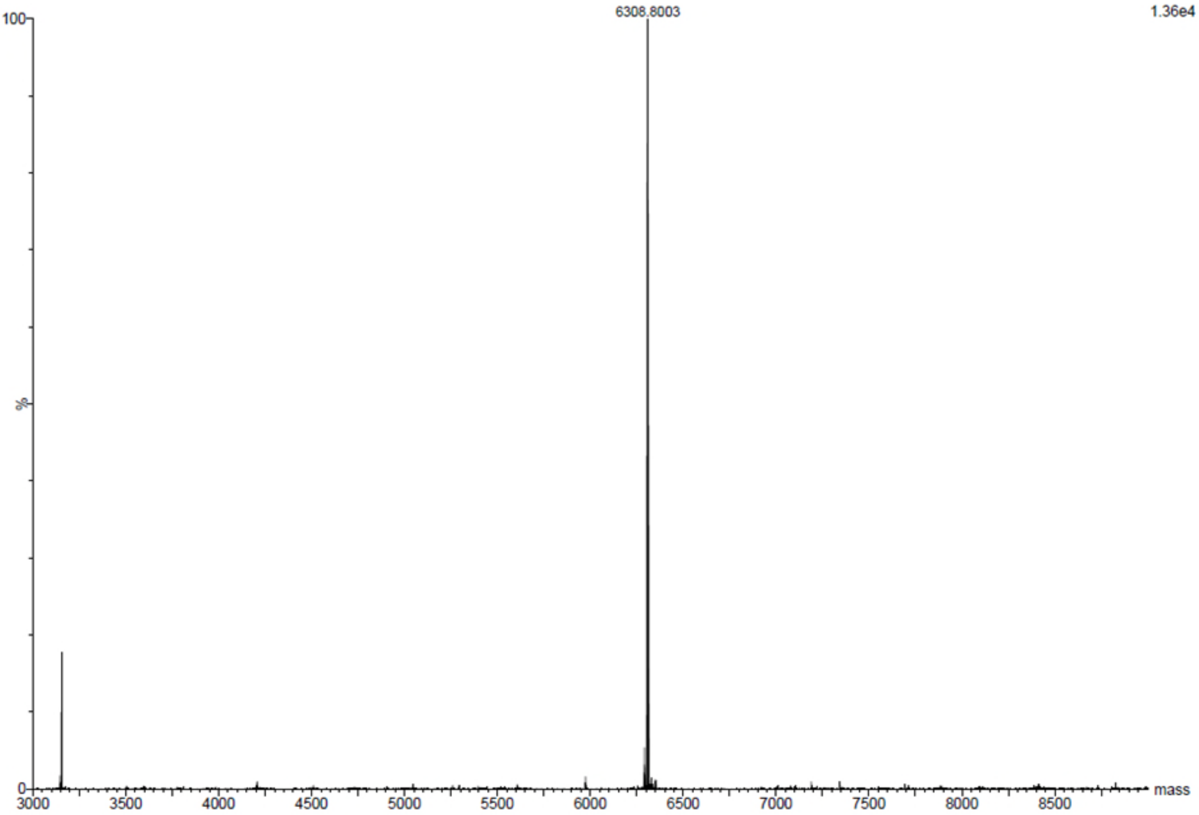

Supplementary Figure S46: Mass spectrum (ES-) of **MOP A3**. Required **6309.15** Da, found **6308.80** Da. y-axis = relative intensity (%), x-axis = mass in Da.

MOP T1	CCU CUU ACC UCA GUT ACA	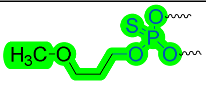

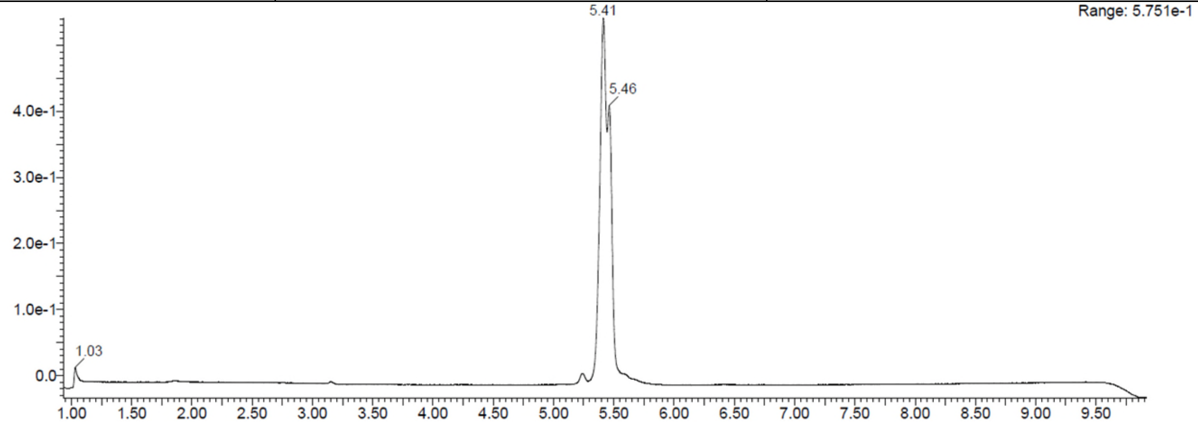

Supplementary Figure S47: Reverse-phase UPLC of **MOP T1** (UV absorbance at 260 nm vs time in min).

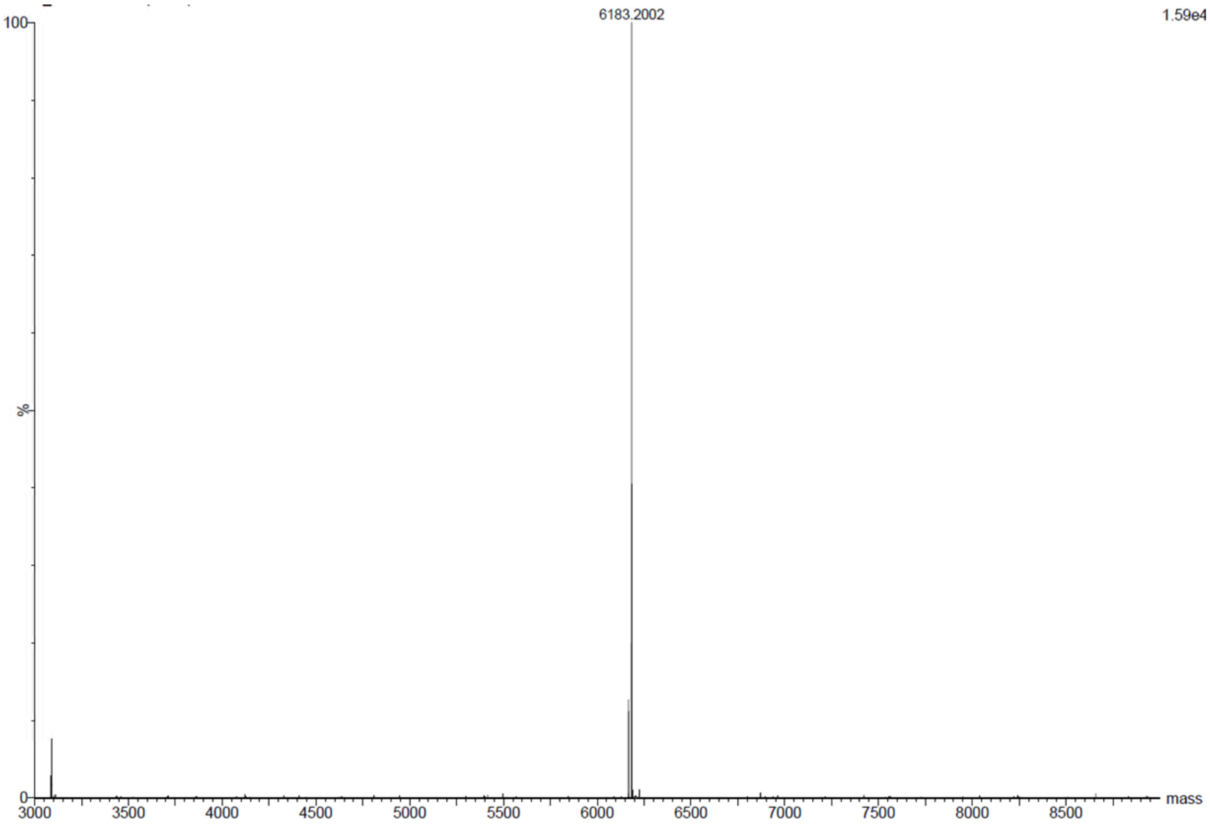

Supplementary Figure S48: Mass spectrum (ES-) of **MOP T1**. Required **6183.00** Da, found **6183.20** Da. y-axis = relative intensity (%), x-axis = mass in Da.

MOP T2	CCU CUT ACC UCA GUT ACA	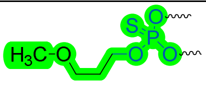

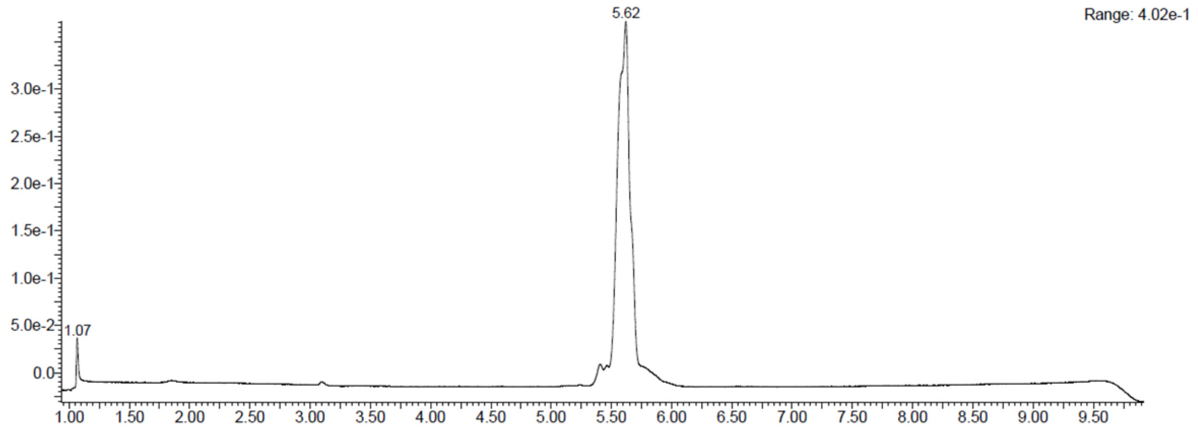

Supplementary Figure 49: Reverse-phase UPLC of **MOP T2** (UV absorbance at 260 nm vs time in min).

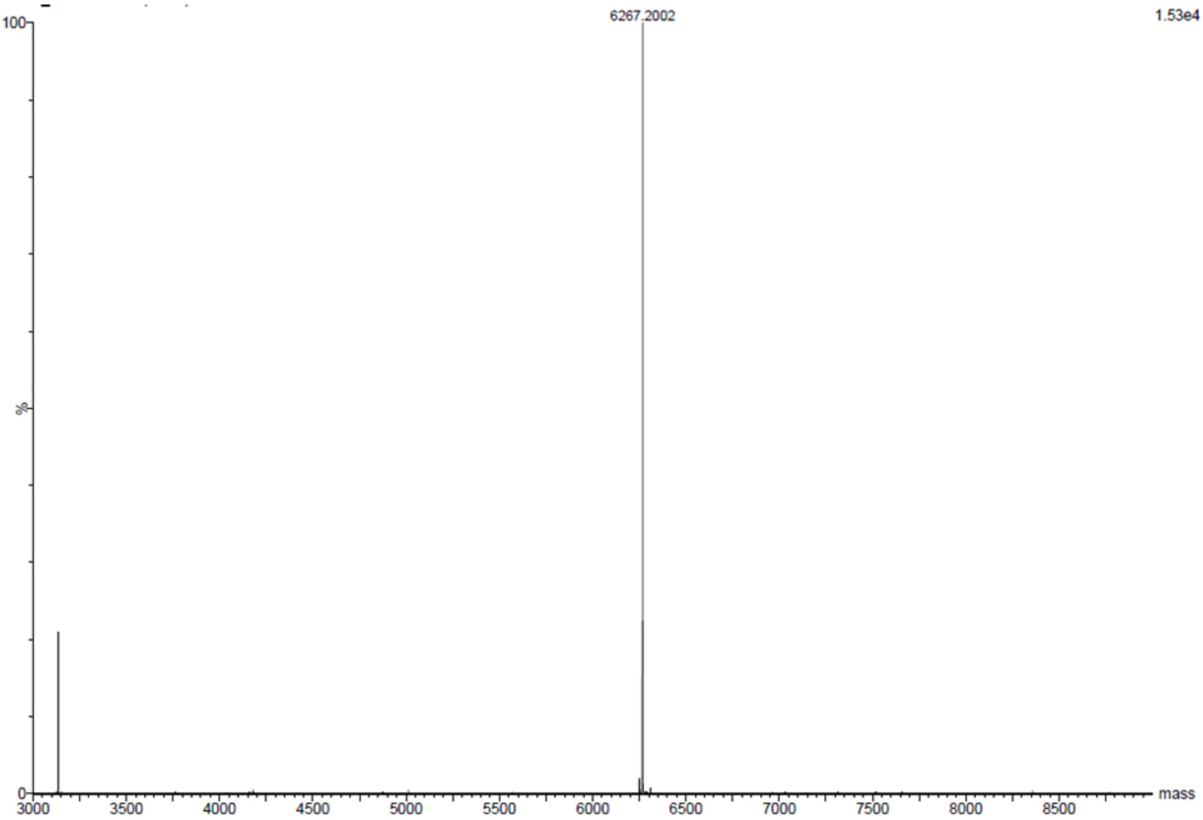

Supplementary Figure S50: Mass spectrum (ES-) of **MOP T2**. Required **6267.11** Da, found **6267.20** Da. y-axis = relative intensity (%), x-axis = mass in Da.

MOP T3	CCU CUT ACC TCA GUT ACA	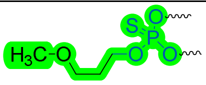

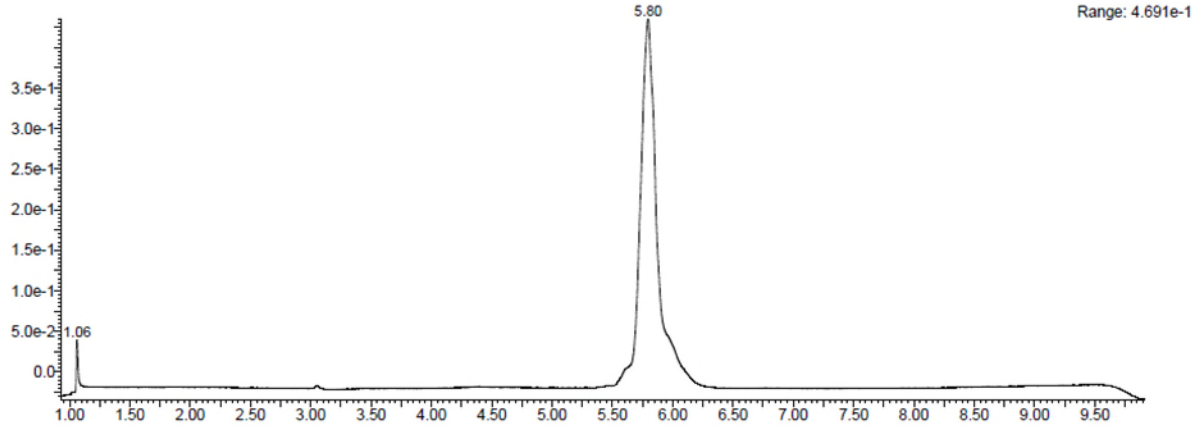

Supplementary Figure S51: Reverse-phase UPLC of **MOP T3** (UV absorbance at 260 nm vs time in min).

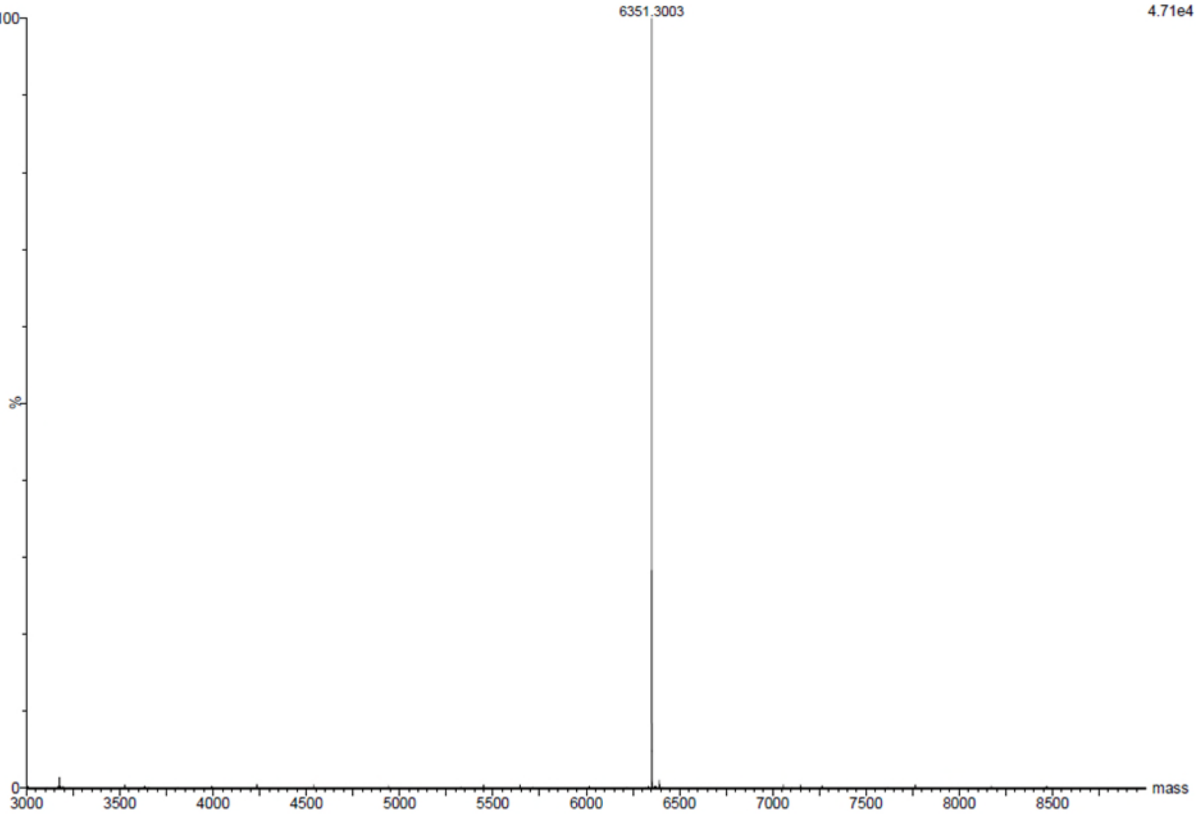

Supplementary Figure S52: Mass spectrum (ES-) of **MOP T3**. Required **6351.23** Da, found **6351.30** Da. y-axis = relative intensity (%), x-axis = mass in Da.

MOP T4	CCT CUT ACC TCA GUT ACA	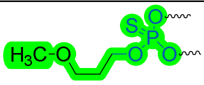

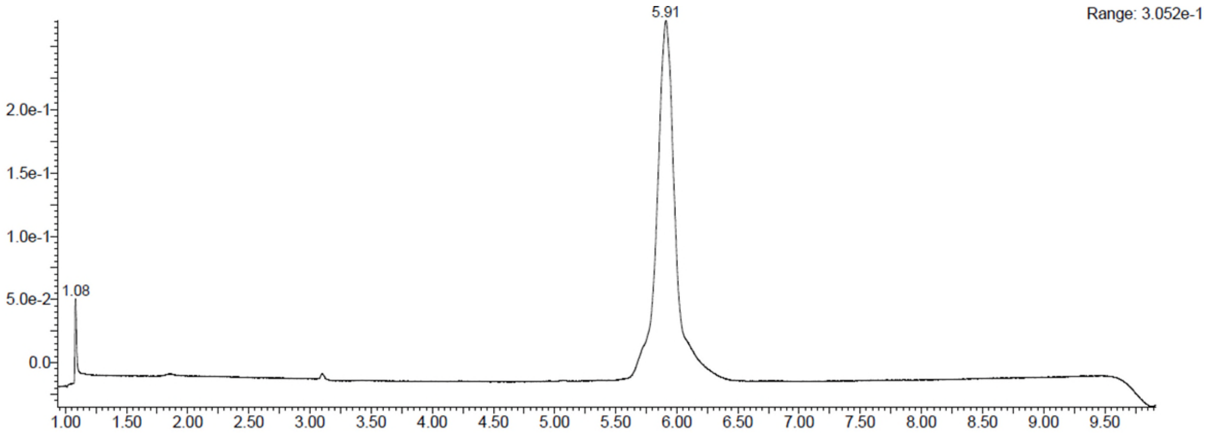

Supplementary Figure S53: Reverse-phase UPLC of **MOP T4** (UV absorbance at 260 nm vs time in min).

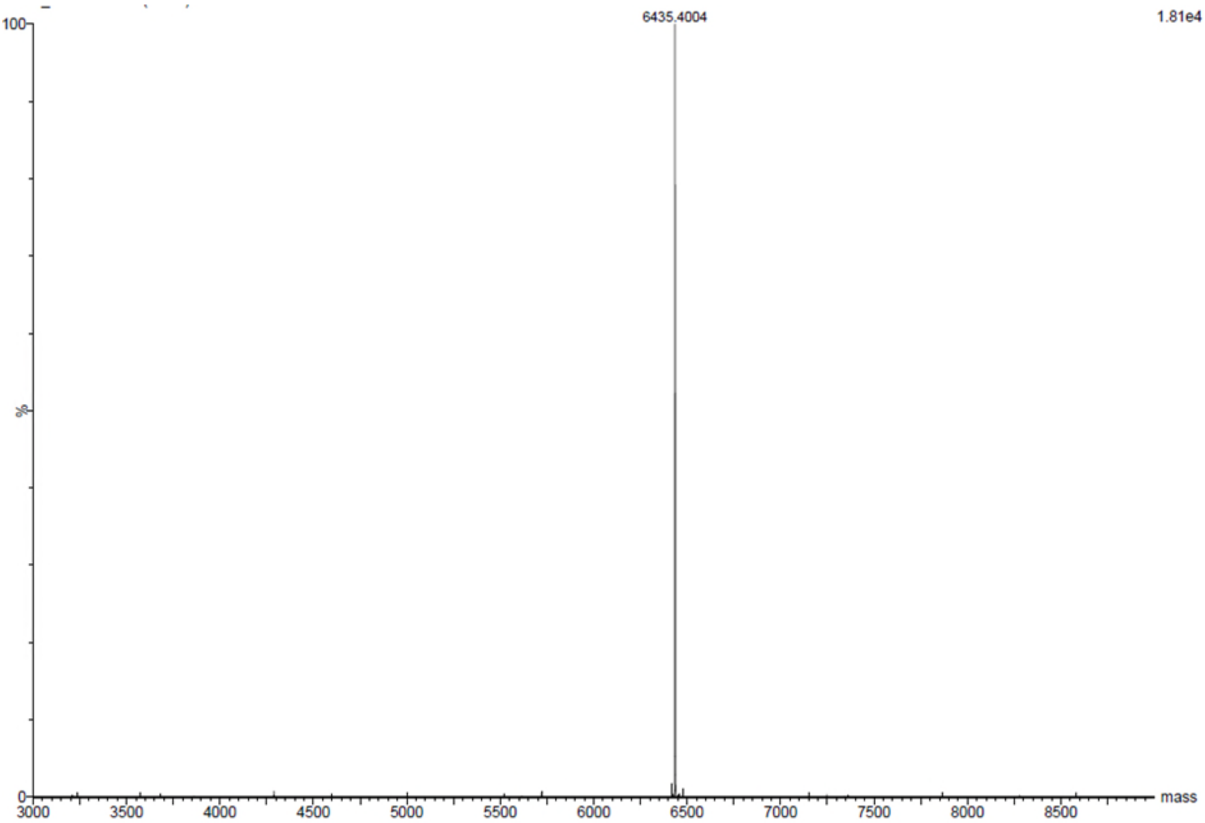

Supplementary Figure S54: Mass spectrum (ES-) of **MOP T4**. Required **6435.35** Da, found **6435.40** Da. y-axis = relative intensity (%), x-axis = mass in Da.

MOP T6	CCT CTT ACC TCA GTT ACA	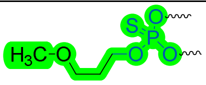

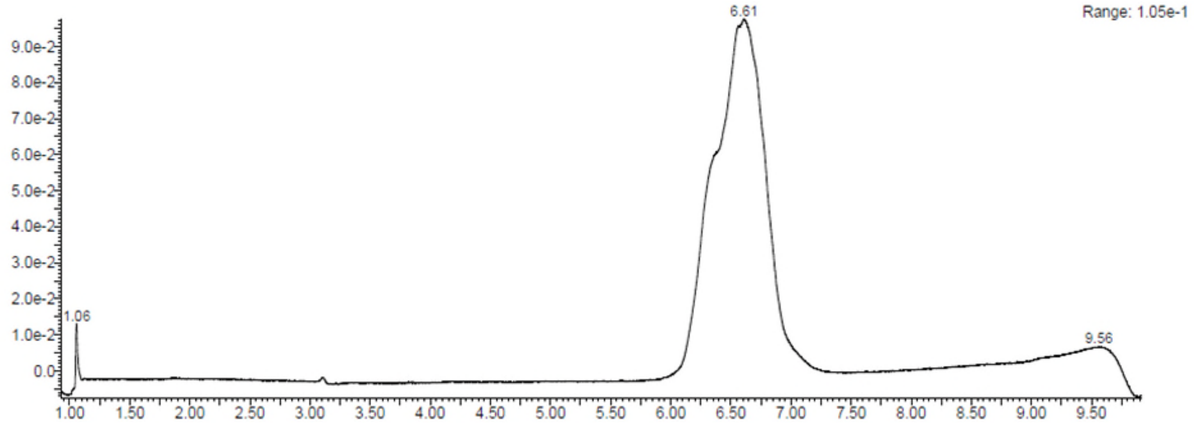

Supplementary Figure S55: Reverse-phase UPLC of **MOP T6** (UV absorbance at 260 nm vs time in min).

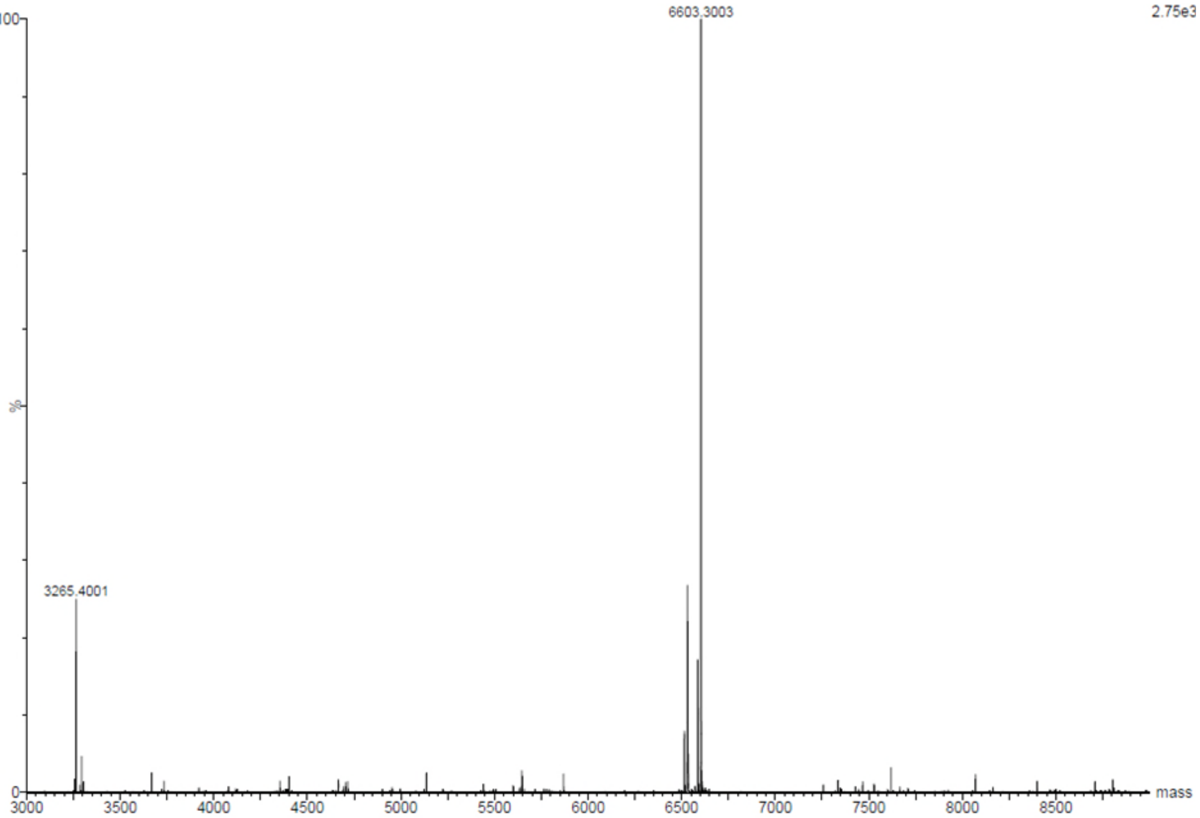

Supplementary Figure S56: Mass spectrum (ES-) of **MOP T6**. Required **6603.59** Da, found **6603.30** Da. y-axis = relative intensity (%), x-axis = mass in Da.

2.5.7 Octyl T2, Octyl T3 and Octyl T4

Octyl T2	CCU CUT ACC UCA GUT ACA	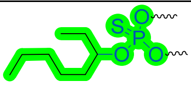

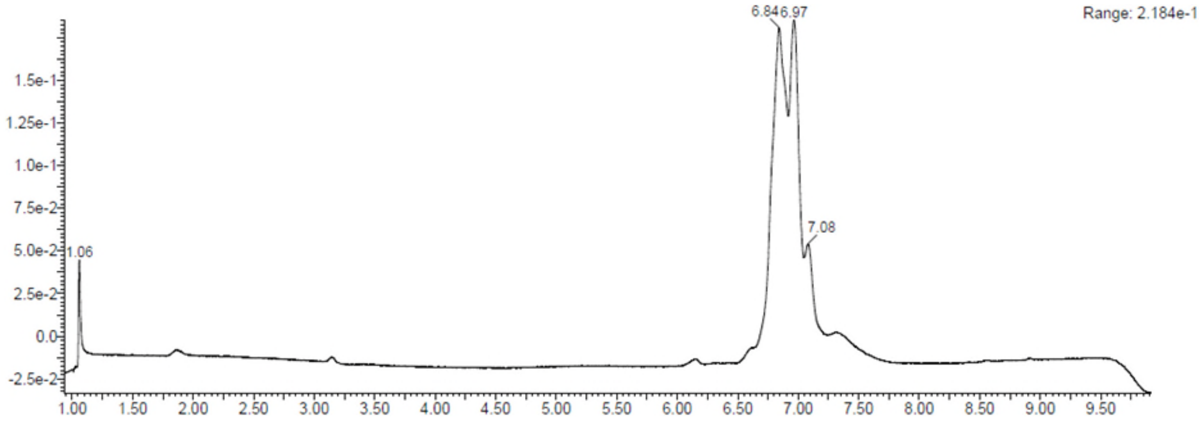

Supplementary Figure S57: Reverse-phase UPLC of **Octyl T2** (UV absorbance at 260 nm vs time in min).

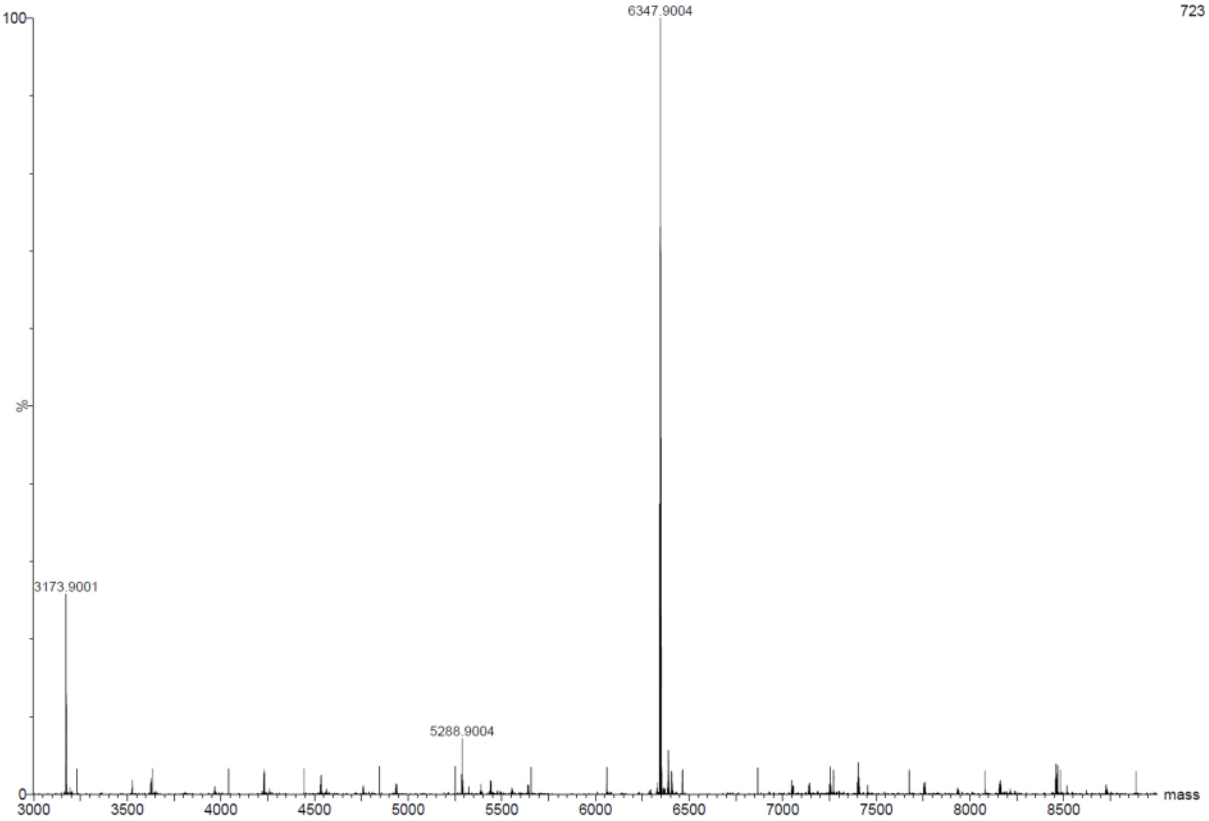

Supplementary Figure S58: Mass spectrum (ES-) of **Octyl T2**. Required **6347.33** Da, found **6347.90** Da. y-axis = relative intensity (%), x-axis = mass in Da.

Octyl T3	CCU CUT ACC TCA GUT ACA	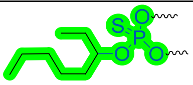

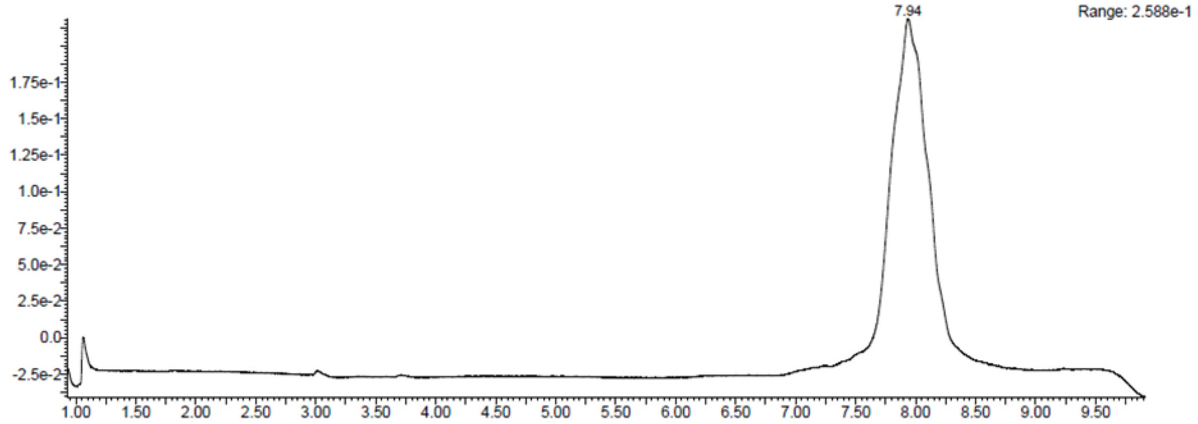

Supplementary Figure S59: Reverse-phase UPLC of **Octyl T3** (UV absorbance at 260 nm vs time in min).

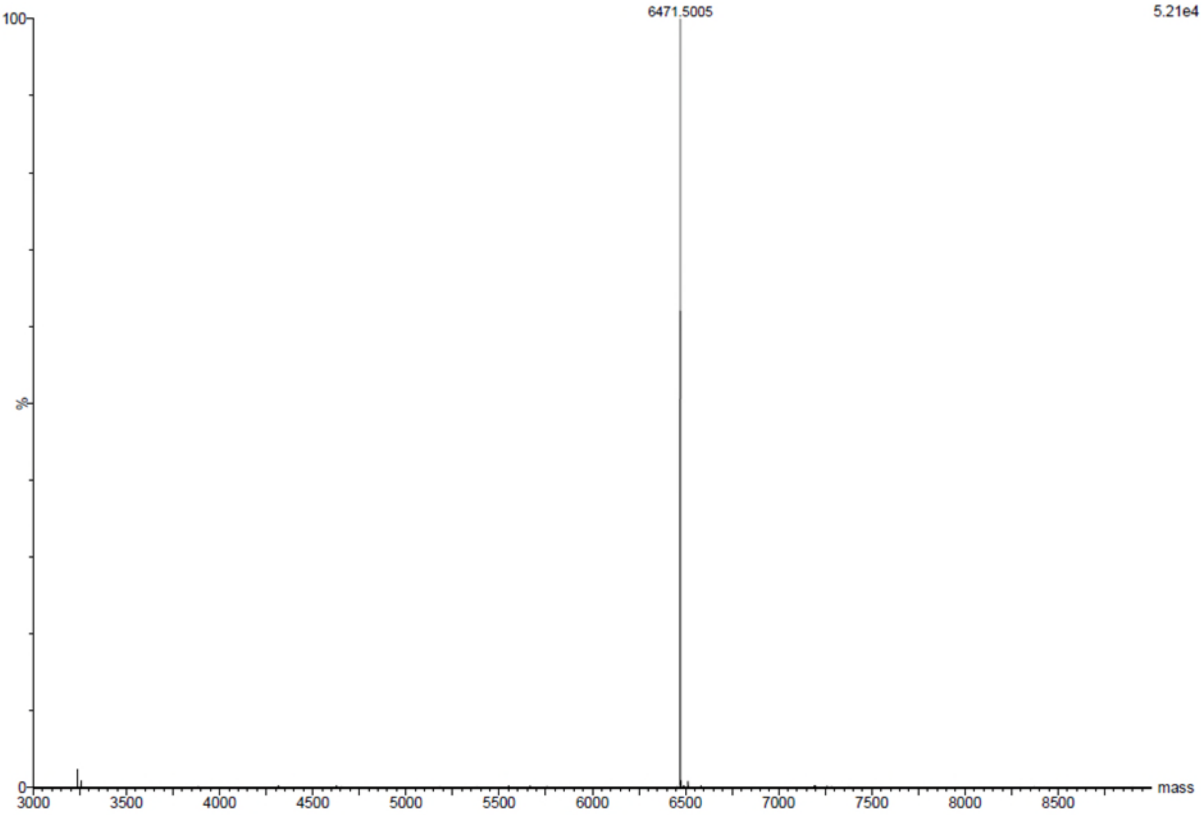

Supplementary Figure S60: Mass spectrum (ES-) of **Octyl T3**. Required **6471.56** Da, found **6471.50** Da. y-axis = relative intensity (%), x-axis = mass in Da.

Octyl T4	CCT CUT ACC TCA GUT ACA	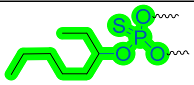

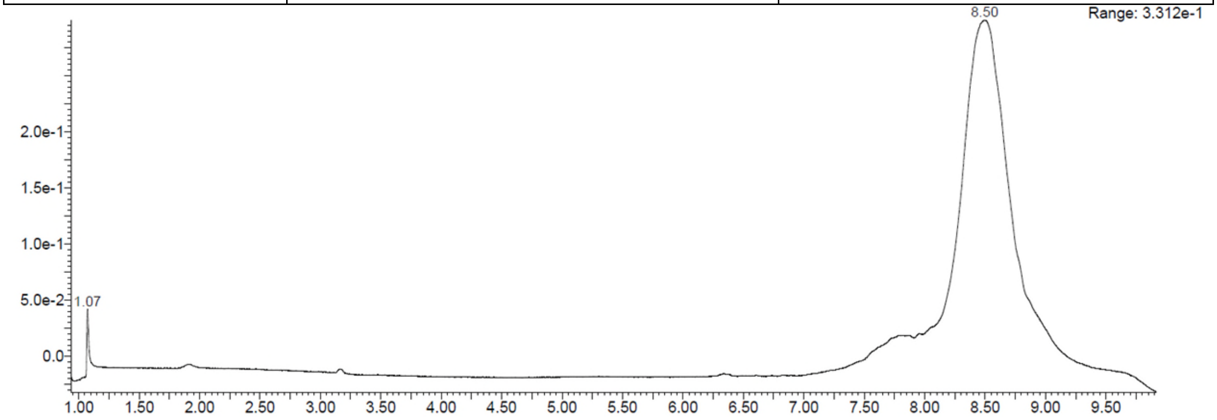

Supplementary Figure S61: Reverse-phase UPLC of **Octyl T4** (UV absorbance at 260 nm vs time in min).

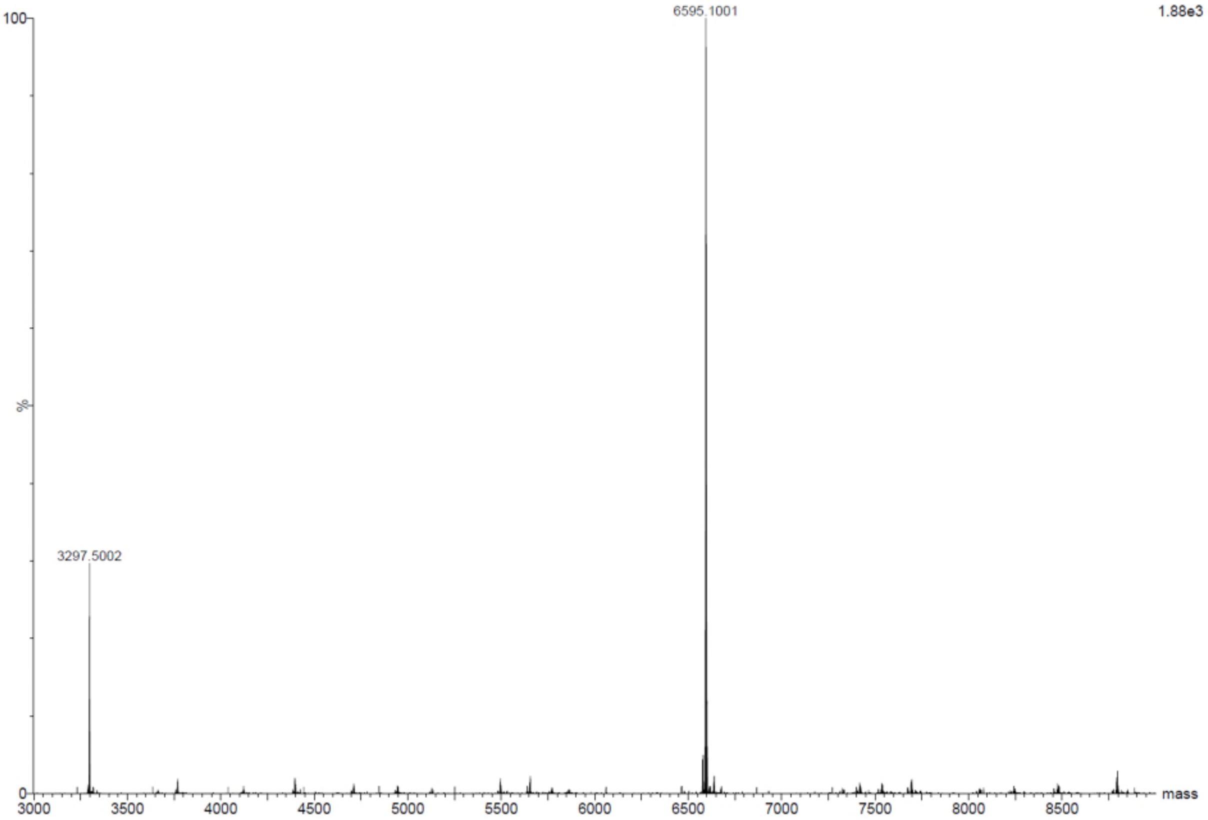

Supplementary Figure S62: Mass spectrum (ES-) of **Octyl T4**. Required **6595.79** Da, found **6595.10** Da. y-axis = relative intensity (%), x-axis = mass in Da.

2.5.8 THP T4 and THP T6 15

THP T4	CCT CUT ACC TCA GUT ACA	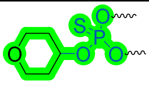

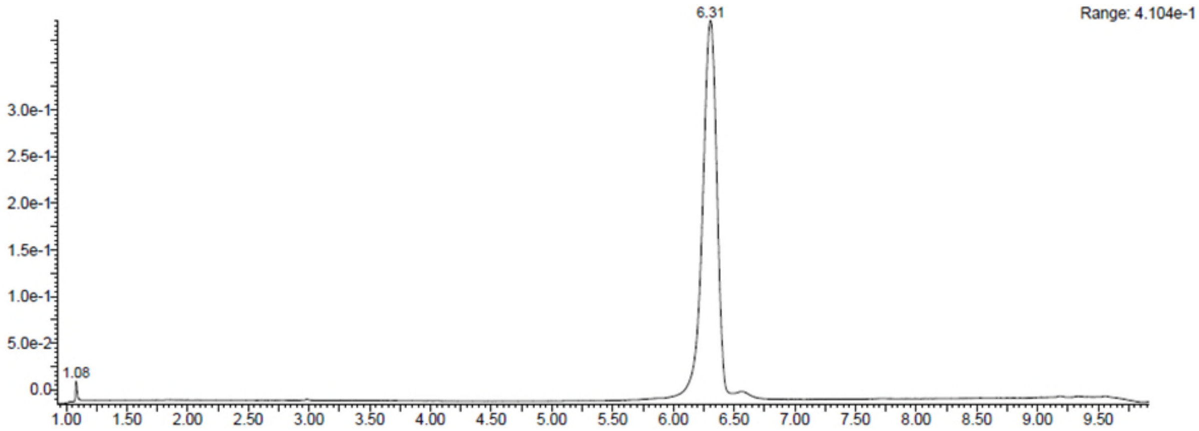

Supplementary Figure S63: Reverse-phase UPLC of **THP T4** (UV absorbance at 260 nm vs time in min).

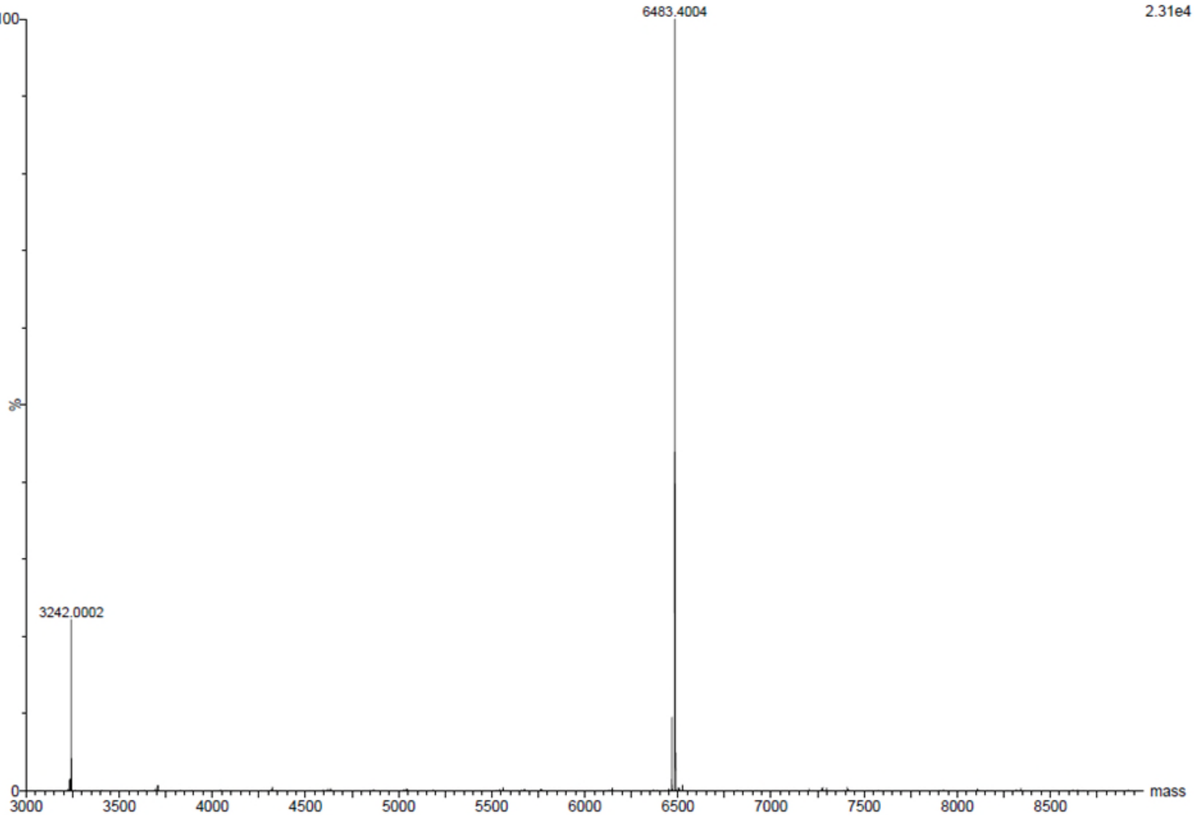

Supplementary Figure S64: Mass spectrum (ES-) of **THP T4**. Required **6483.39** Da, found **6483.40** Da. y-axis = relative intensity (%), x-axis = mass in Da.

THP T6 15	T CTT ACC TCA GTT AC	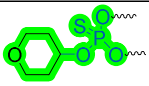

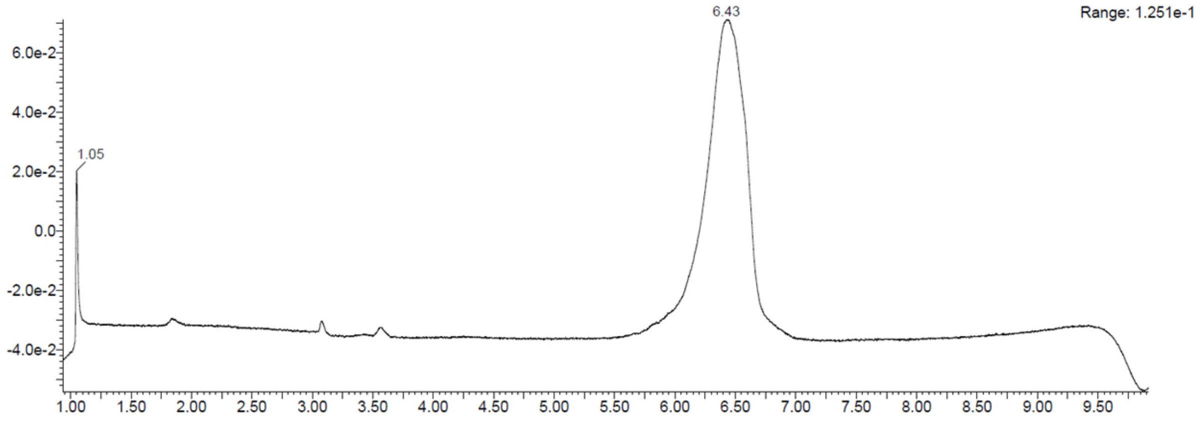

Supplementary Figure S65: Reverse-phase UPLC of **THP T6 15** (UV absorbance at 260 nm vs time in min).

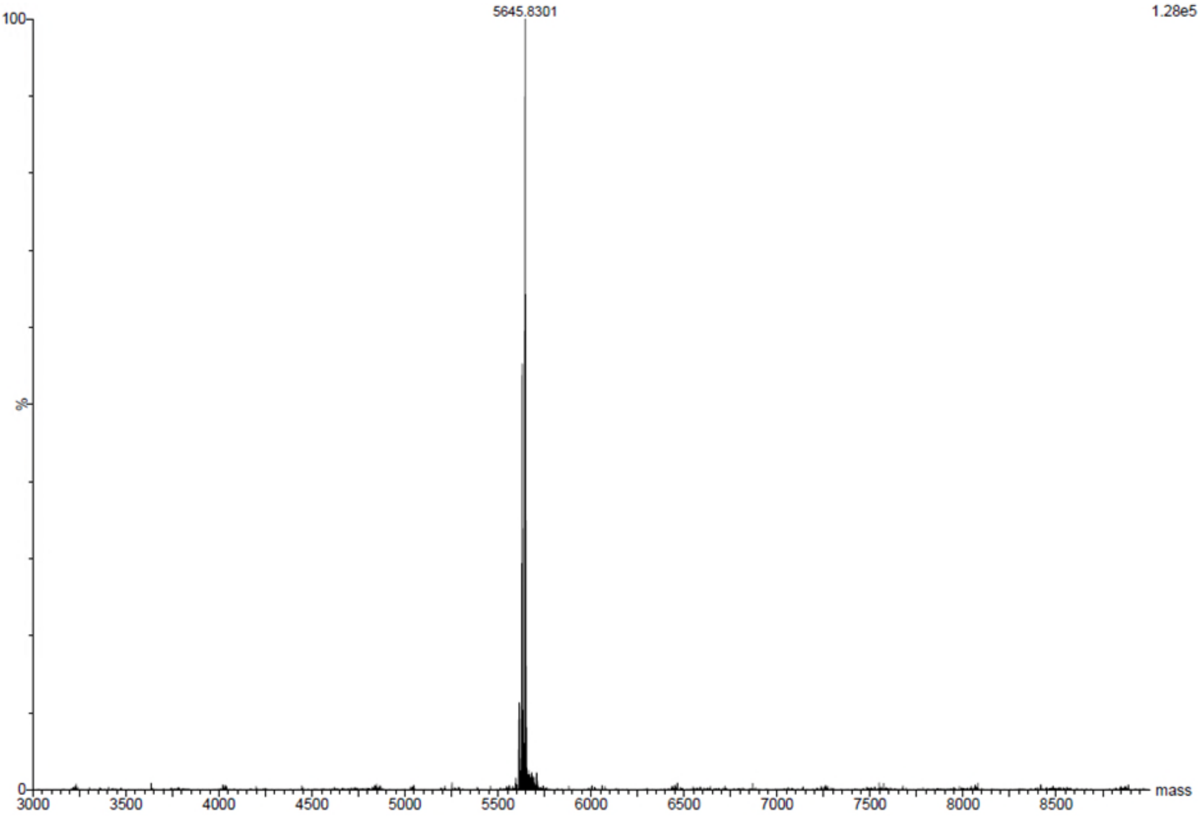

Supplementary Figure S66: Mass spectrum (ES-) of **THP T6 15**. Required **5645.81** Da, found **5645.83** Da. y-axis = relative intensity (%), x-axis = mass in Da.

2.5.9 Hexyn A1 and Hexyn A2

Hexyn A1	CCU CUU ACC UCA GUU ACA	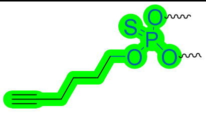

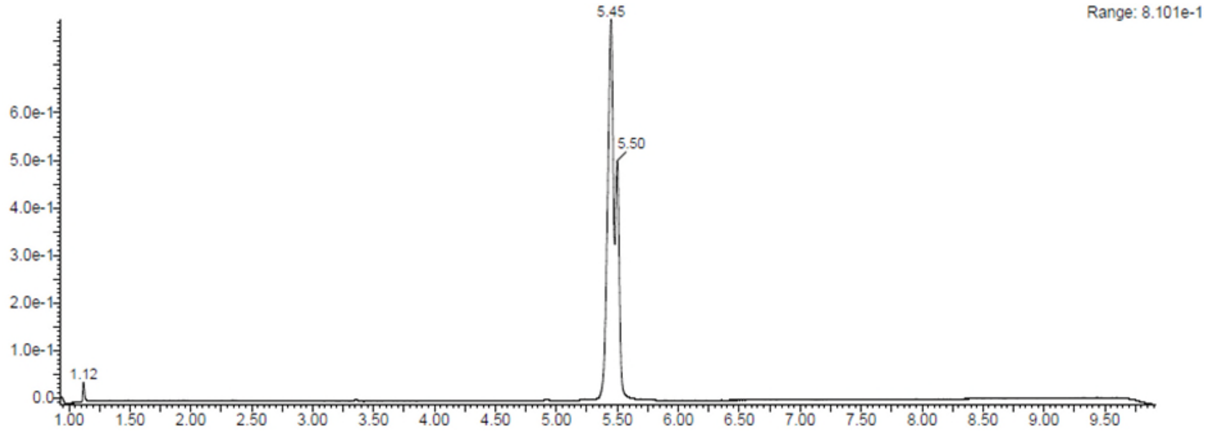

Supplementary Figure S67: Reverse-phase UPLC of **Hexyn A1** (UV absorbance at 260 nm vs time in min).

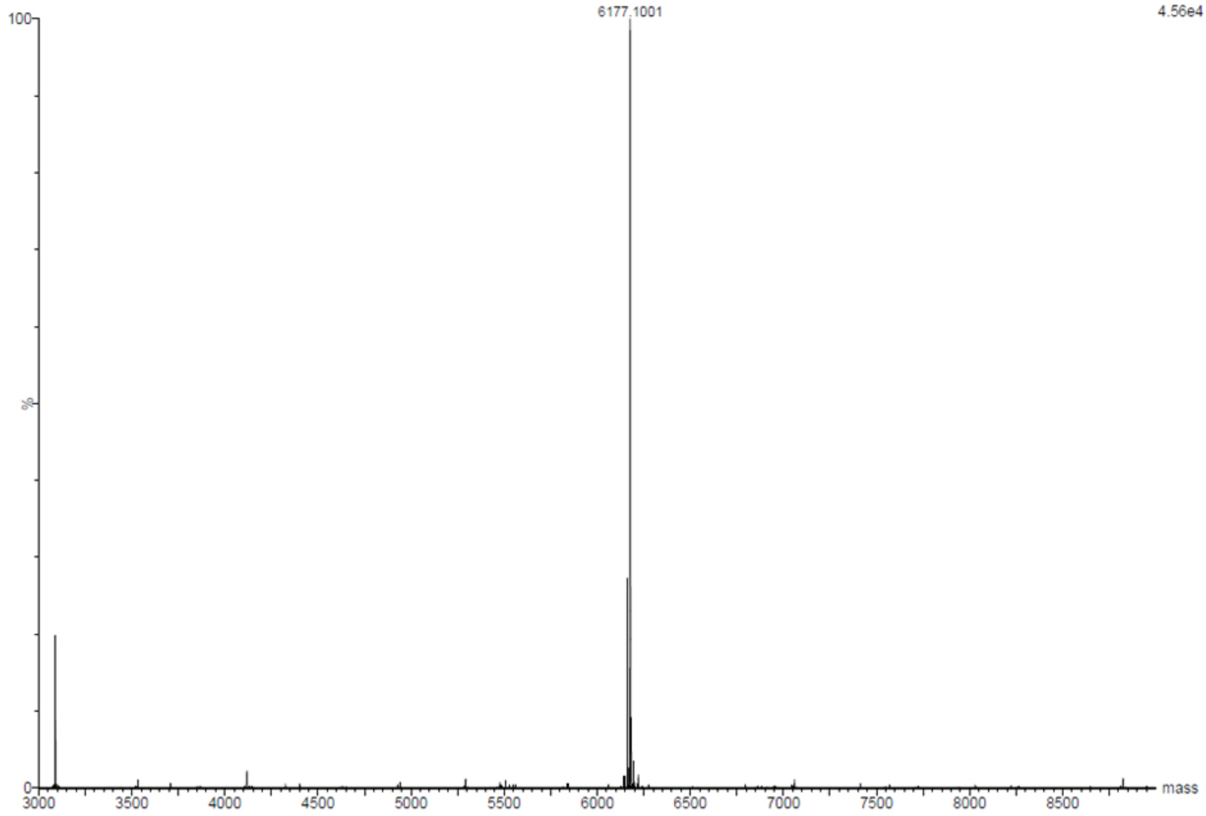

Supplementary Figure S68: Mass spectrum (ES-) of **Hexyn A1**. Required **6176.99** Da, found **6177.10** Da. y-axis = relative intensity (%), x-axis = mass in Da.

Hexyn A2	CCU CUU ACC UCA GUU ACA	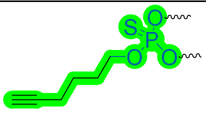

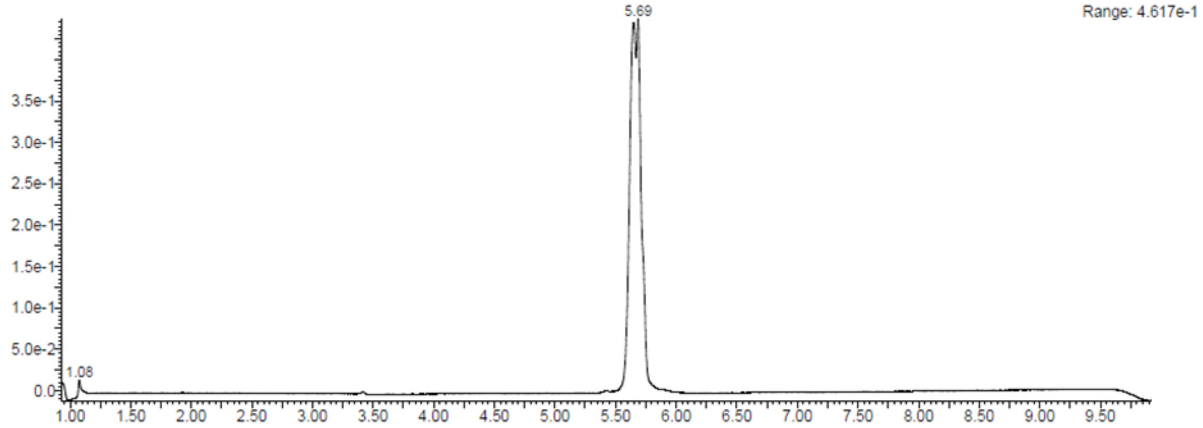

Supplementary Figure S69: Reverse-phase UPLC of **Hexyn A2** (UV absorbance at 260 nm vs time in min).

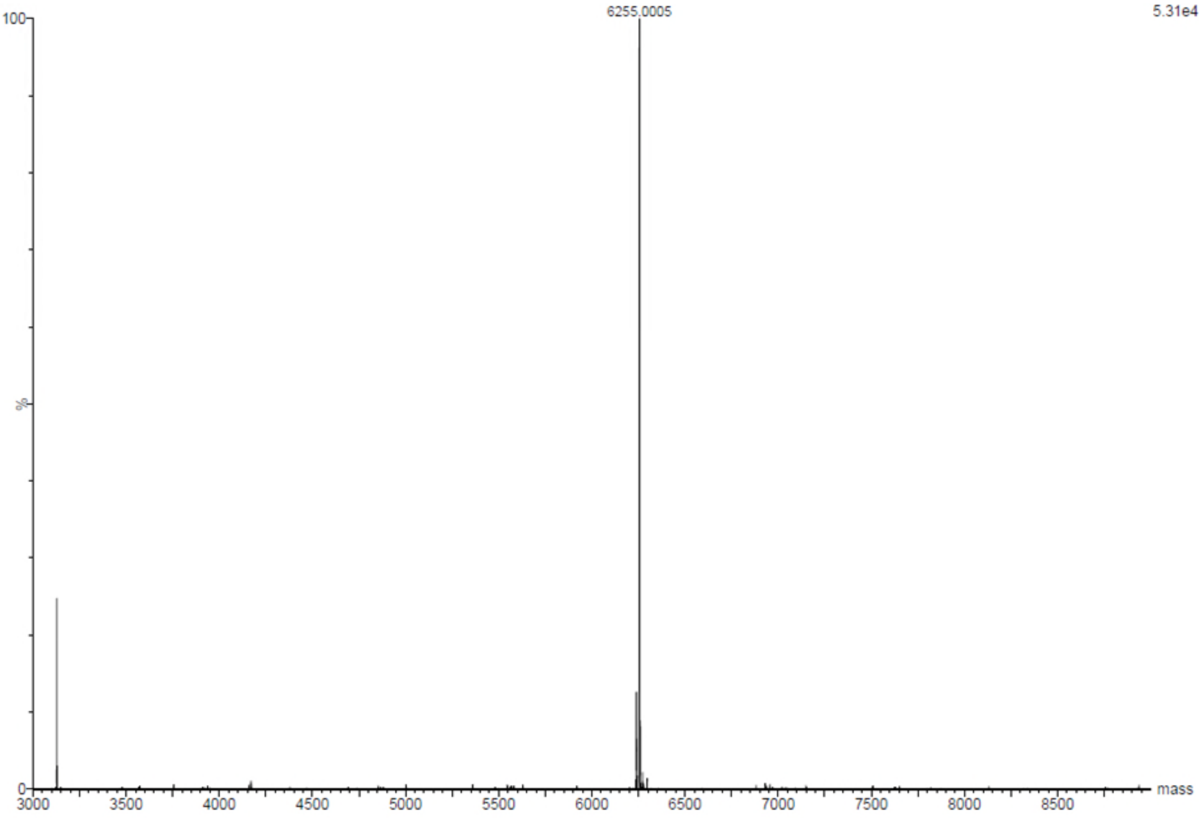

Supplementary Figure S70: Mass spectrum (ES-) of **Hexyn A2**. Required **6255.10** Da, found **6255.00** Da. y-axis = relative intensity (%), x-axis = mass in Da.

2.5.10 Pentyn T1, Pentyn T2 and Pentyn T3

Pentyn T1	CCU CUU ACC UCA GUT ACA	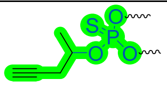

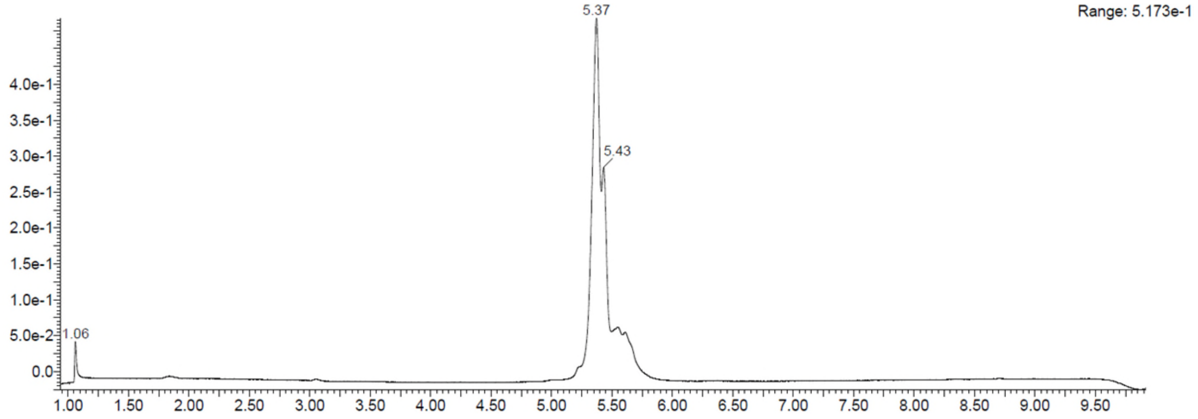

Supplementary Figure S71: Reverse-phase UPLC of **Pentyn T1** (UV absorbance at 260 nm vs time in min).

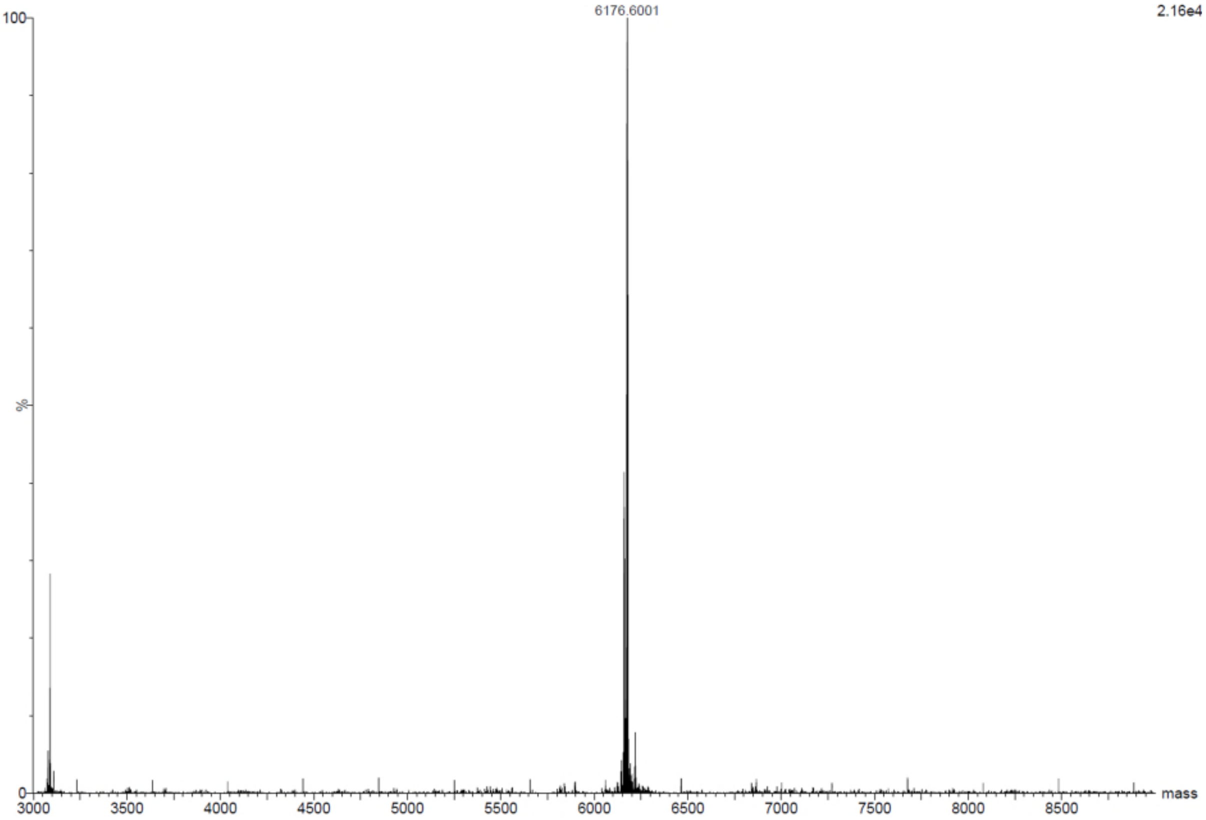

Supplementary Figure S72: Mass spectrum (ES-) of **Pentyn T1**. Required **6176.99** Da, found **6176.60** Da. y-axis = relative intensity (%), x-axis = mass in Da.

Pentyn T2	CCU CUT ACC UCA GUT ACA	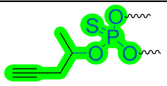

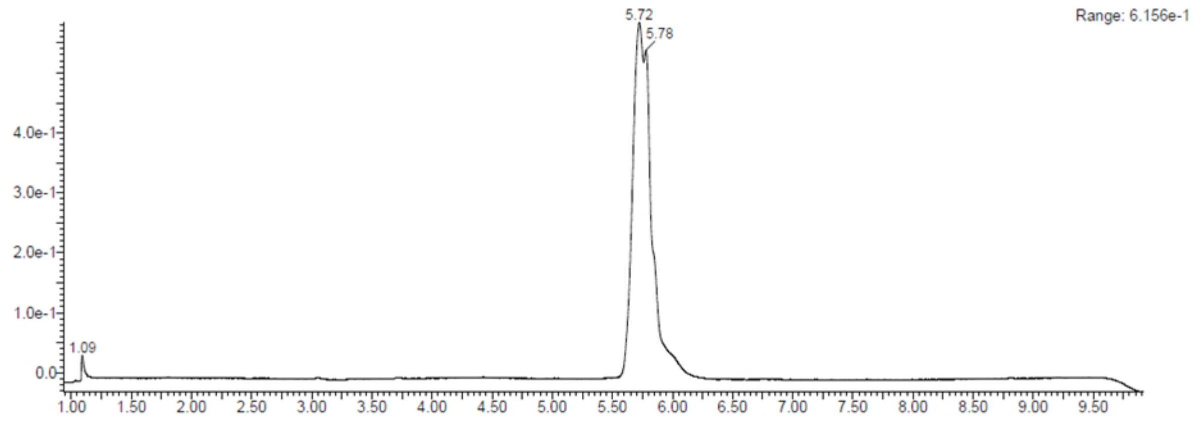

Supplementary Figure S73: Reverse-phase UPLC of **Pentyn T2** (UV absorbance at 260 nm vs time in min).

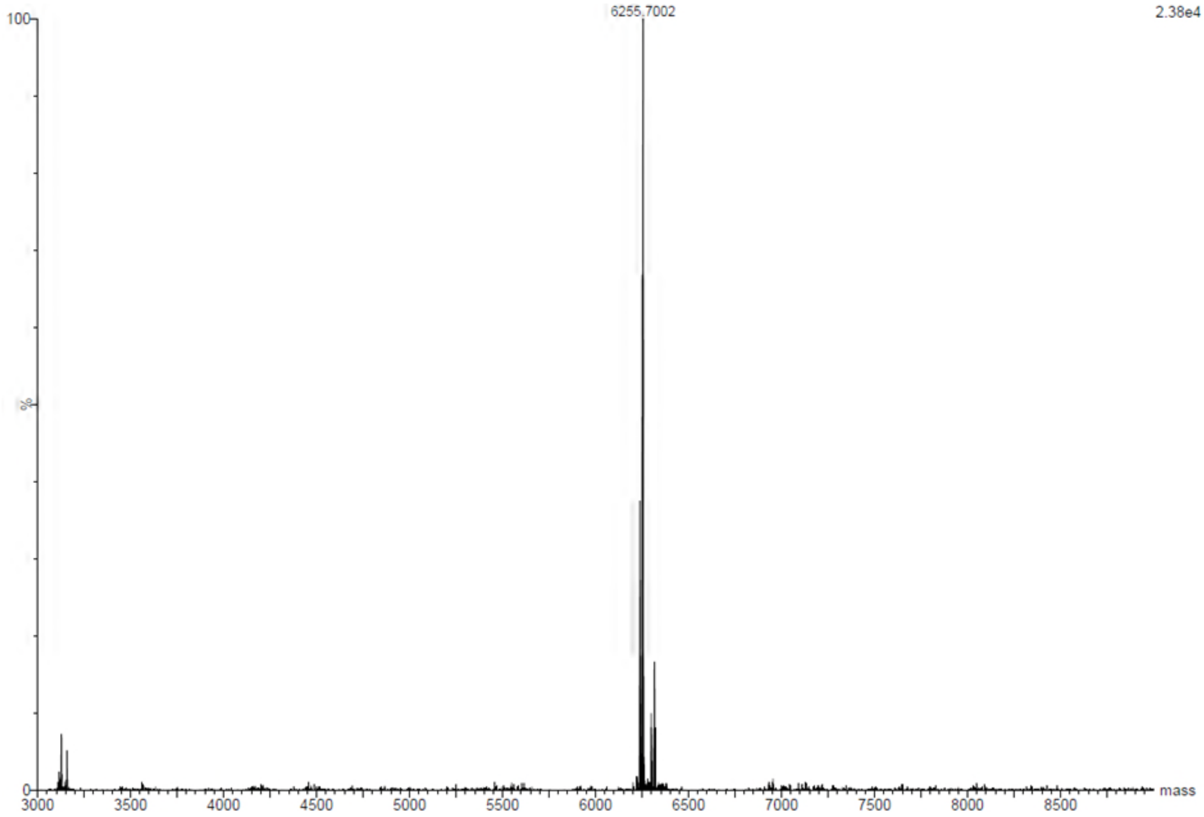

Supplementary Figure S74: Mass spectrum (ES-) of **Pentyn T2**. Required **6255.11** Da, found **6255.70** Da. y-axis = relative intensity (%), x-axis = mass in Da.

Pentyn T3	CCU CUT ACC TCA GUT ACA	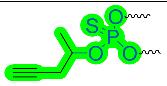

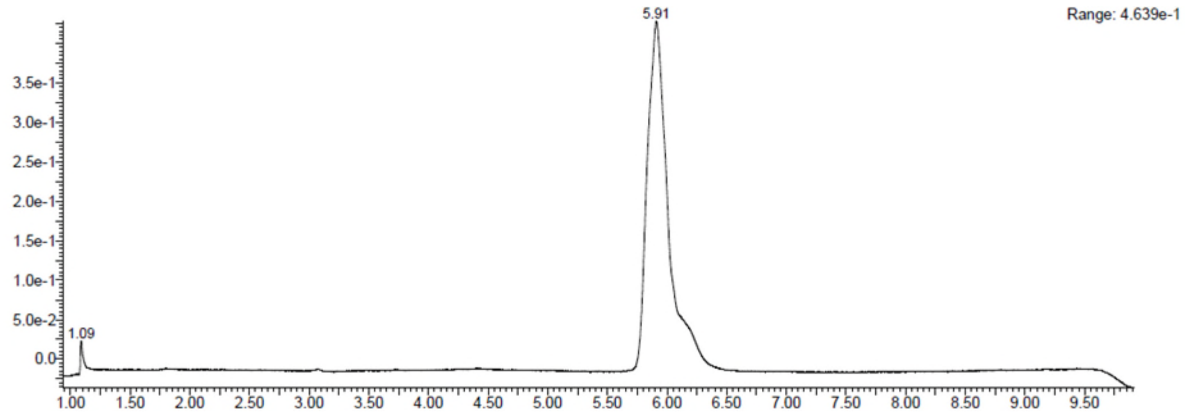

Supplementary Figure S75: Reverse-phase UPLC of **Pentyn T3** (UV absorbance at 260 nm vs time in min).

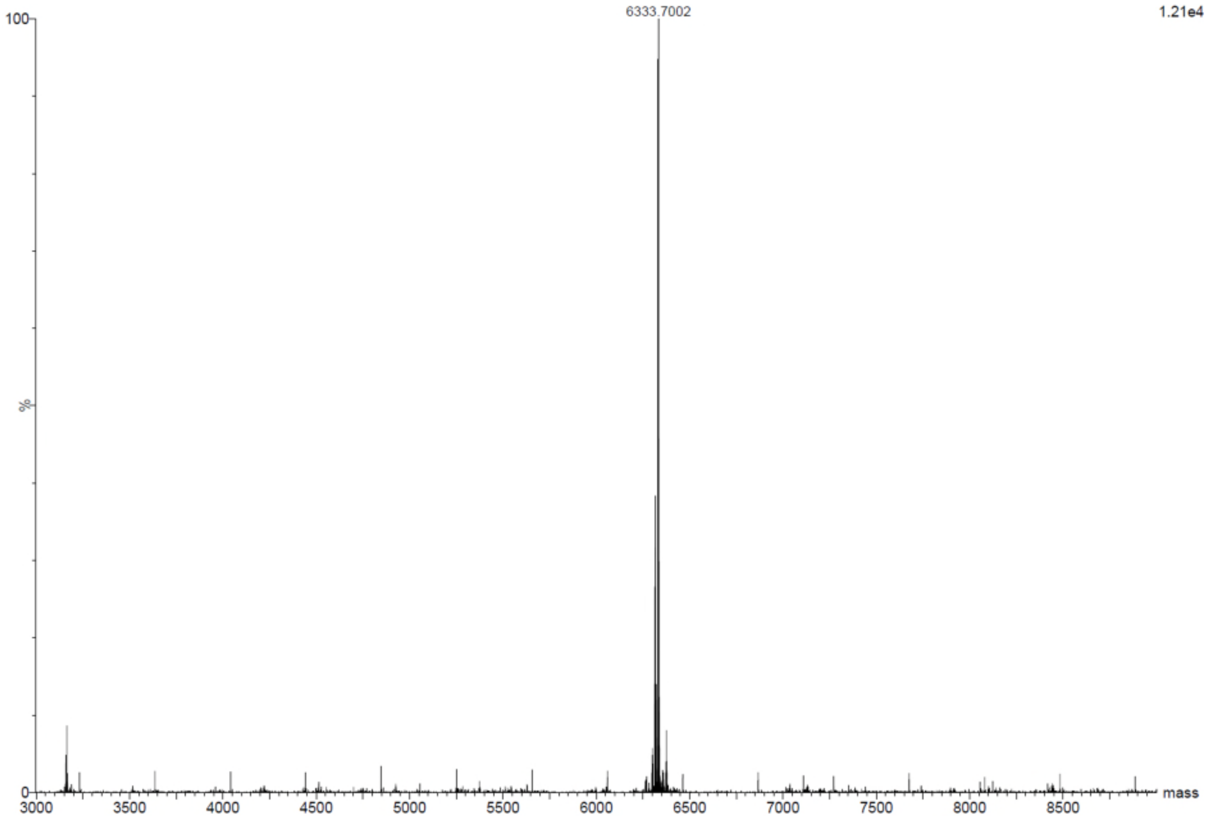

Supplementary Figure S76: Mass spectrum (ES-) of **Pentyn T3**. Required **6333.22** Da, found **6333.70** Da. y-axis = relative intensity (%), x-axis = mass in Da.

2.5.11 Glycol T1, Glycol T2 and Glycol T3

Glycol T1	CCU CUU ACC UCA GUT ACA	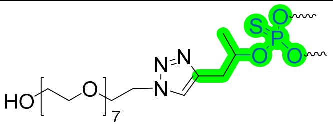

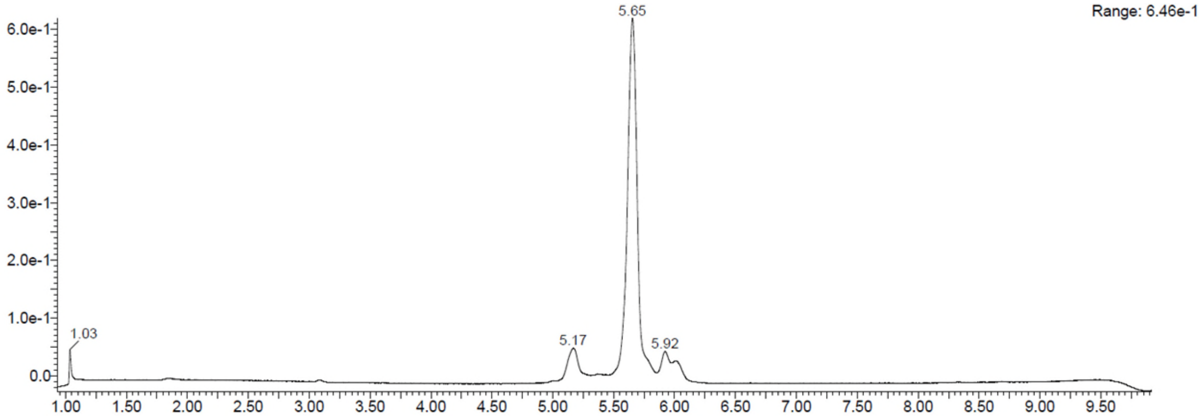

Supplementary Figure S77: Reverse-phase UPLC of **Glycol T1** (UV absorbance at 260 nm vs time in min).

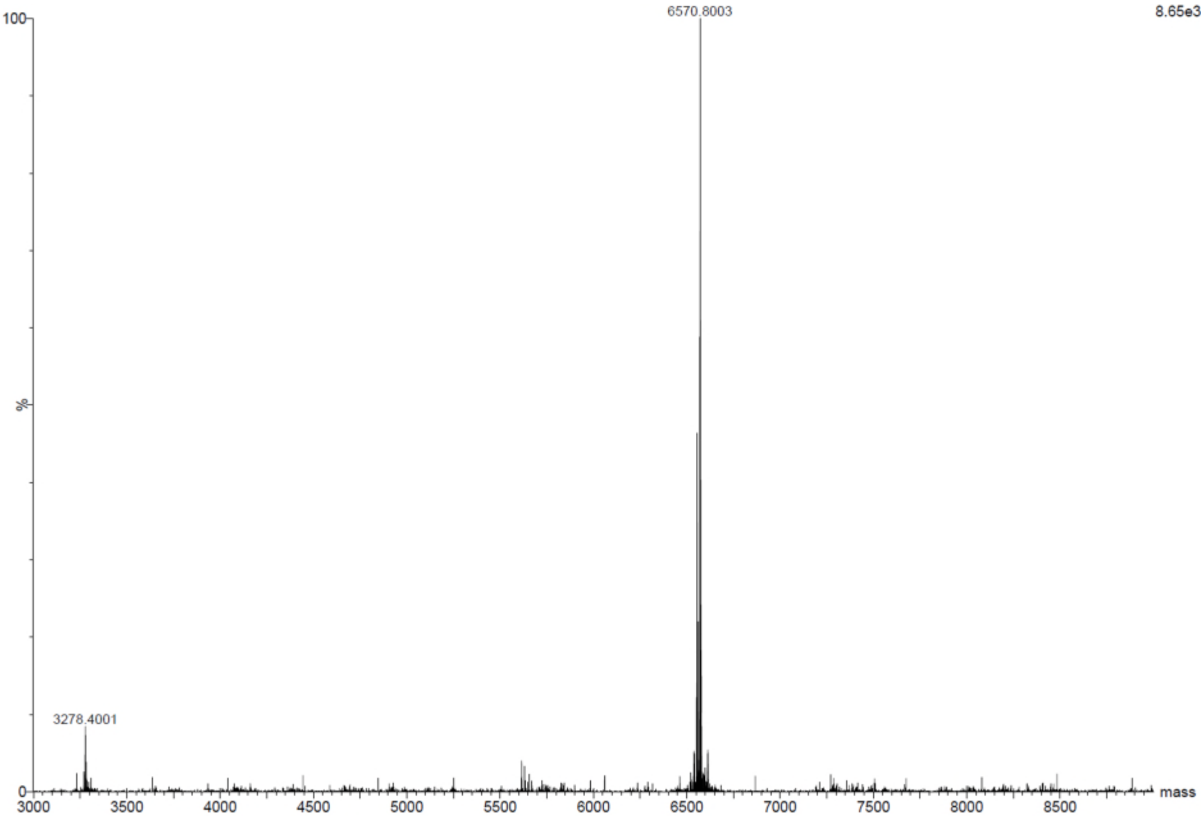

Supplementary Figure S78: Mass spectrum (ES-) of **Glycol T1**. Required **6572.44** Da, found **6570.80** Da. y-axis = relative intensity (%), x-axis = mass in Da.

Glycol T2	CCU CUT ACC UCA GUT ACA	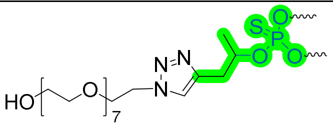

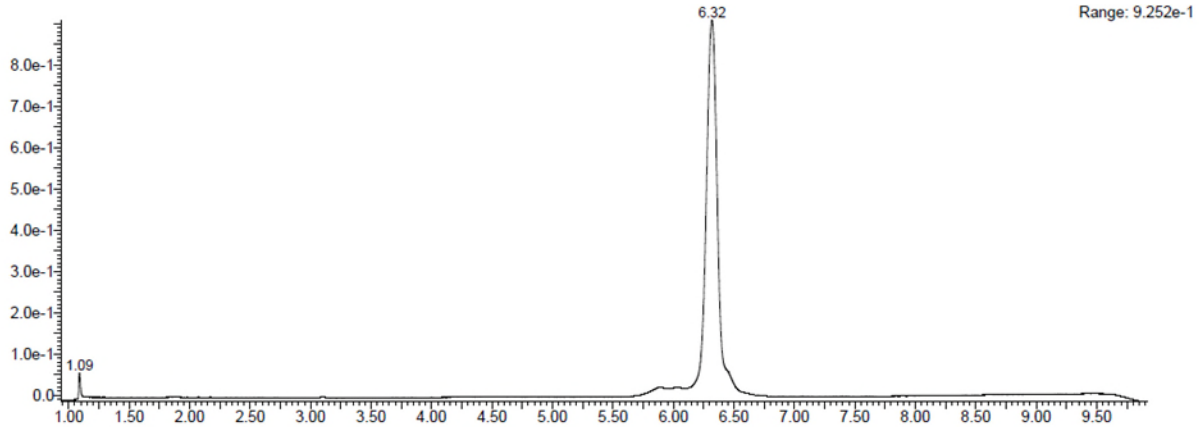

Supplementary Figure S79: Reverse-phase UPLC of **Glycol T2** (UV absorbance at 260 nm vs time in min).

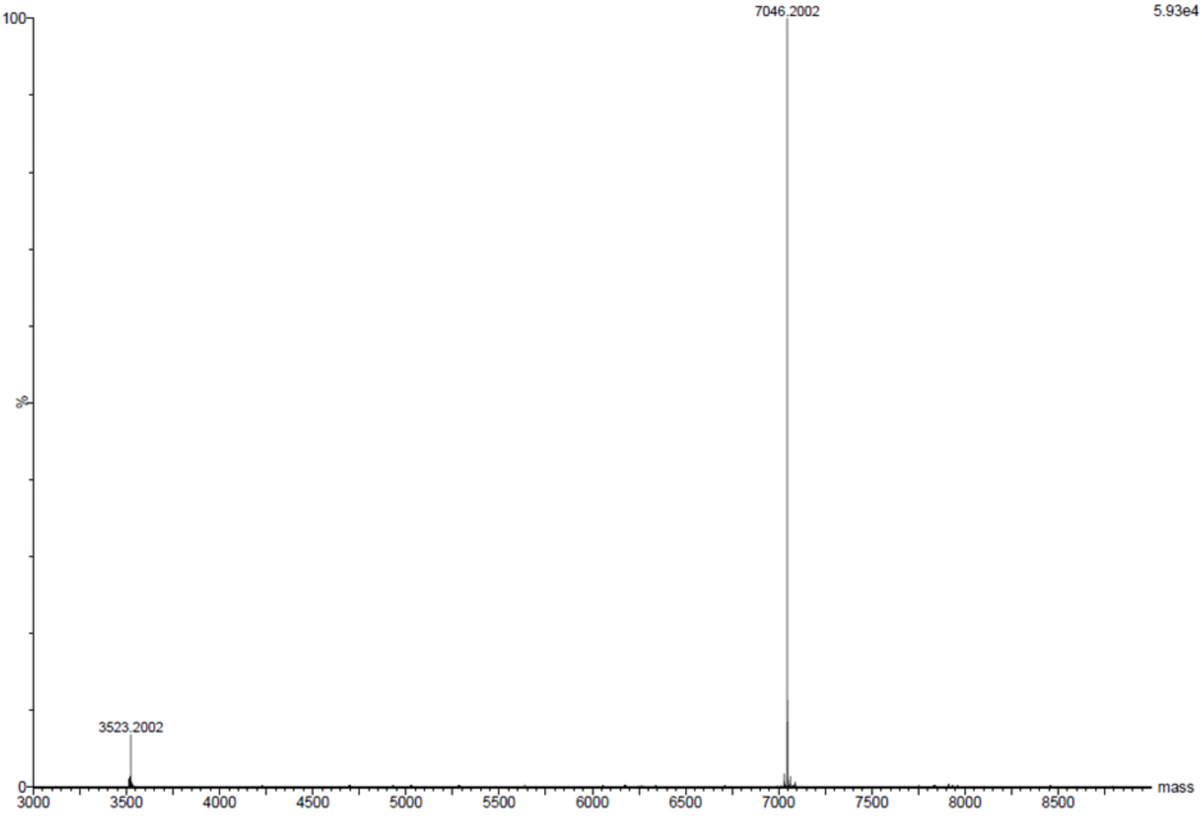

Supplementary Figure S80: Mass spectrum (ES-) of **Glycol T2**. Required **7046.01** Da, found **7046.20** Da. y-axis = relative intensity (%), x-axis = mass in Da.

Glycol T3	CCU CUT ACC TCA GUT ACA	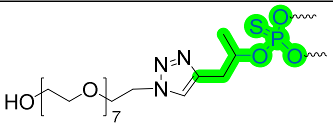

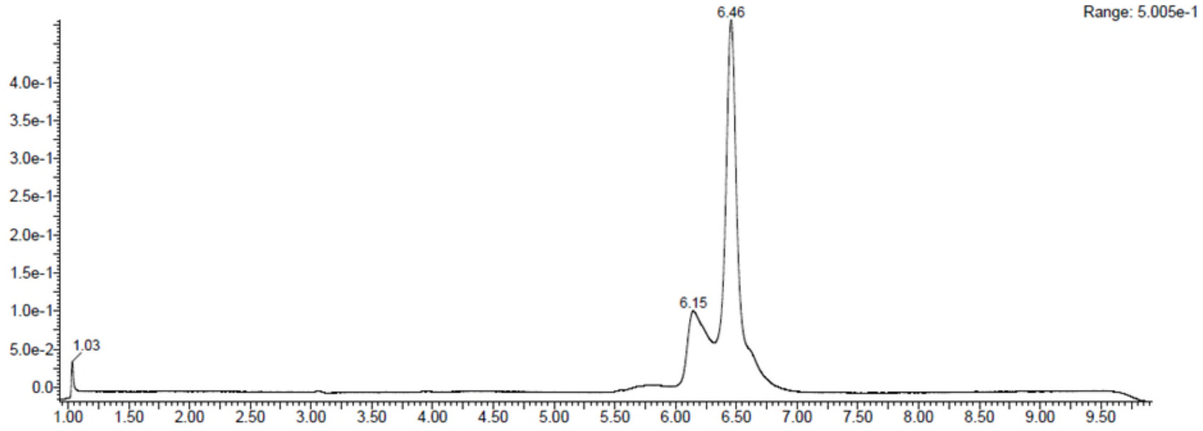

Supplementary Figure S81: Reverse-phase UPLC of **Glycol T3** (UV absorbance at 260 nm vs time in min).

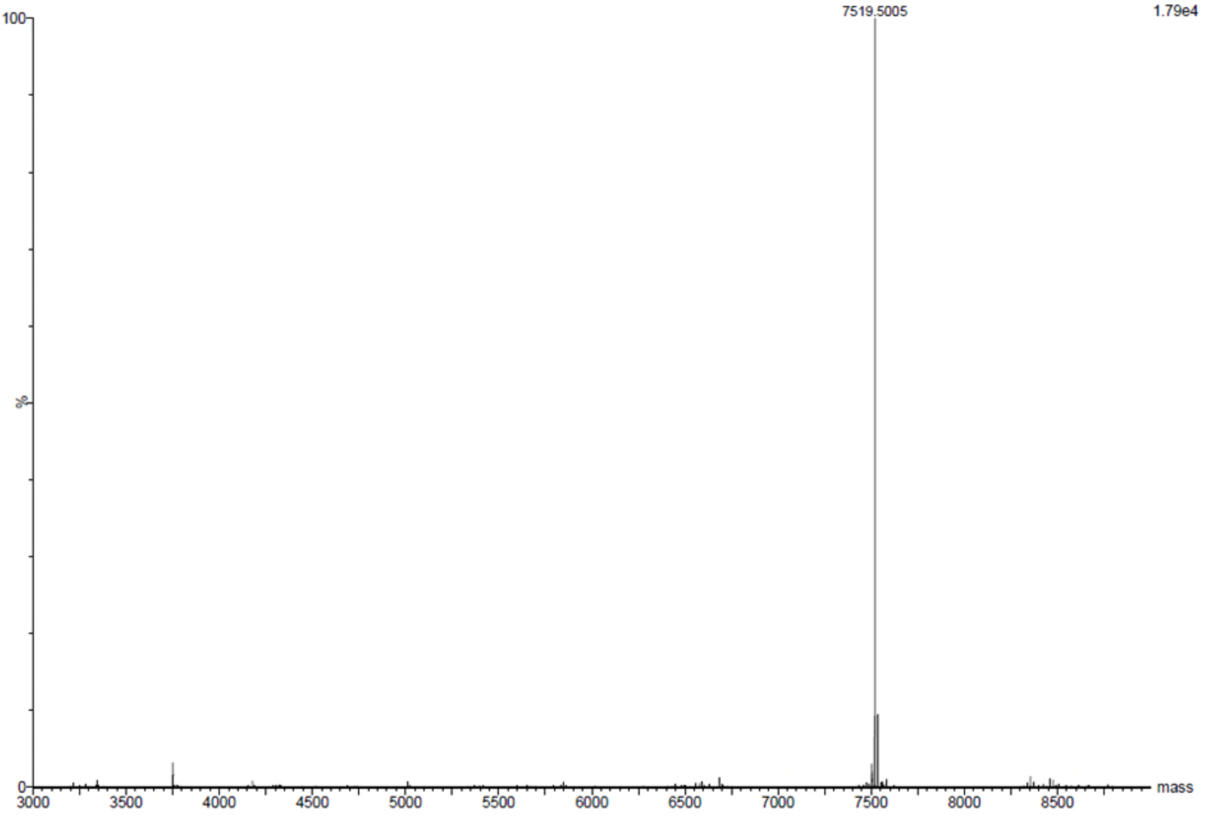

Supplementary Figure S82: Mass spectrum (ES-) of **Glycol T3**. Required **6333.22** Da, found **6333.70** Da. y-axis = relative intensity (%), x-axis = mass in Da.

2.5.12 Gluc A1 and Gluc A2

Glu A1	CCU CUU ACC UCA GUU ACA	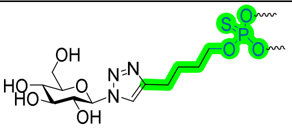

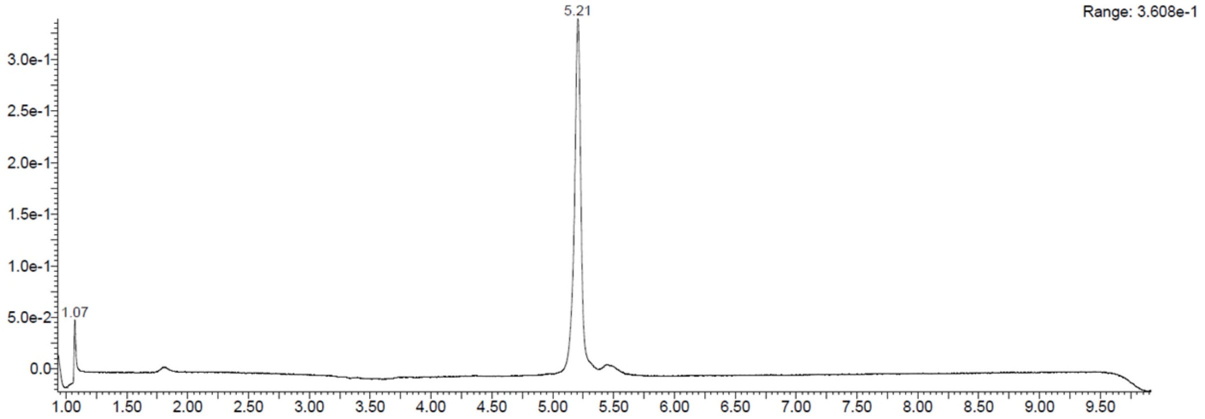

Supplementary Figure S83: Reverse-phase UPLC of **Glu A1** (UV absorbance at 260 nm vs time in min).

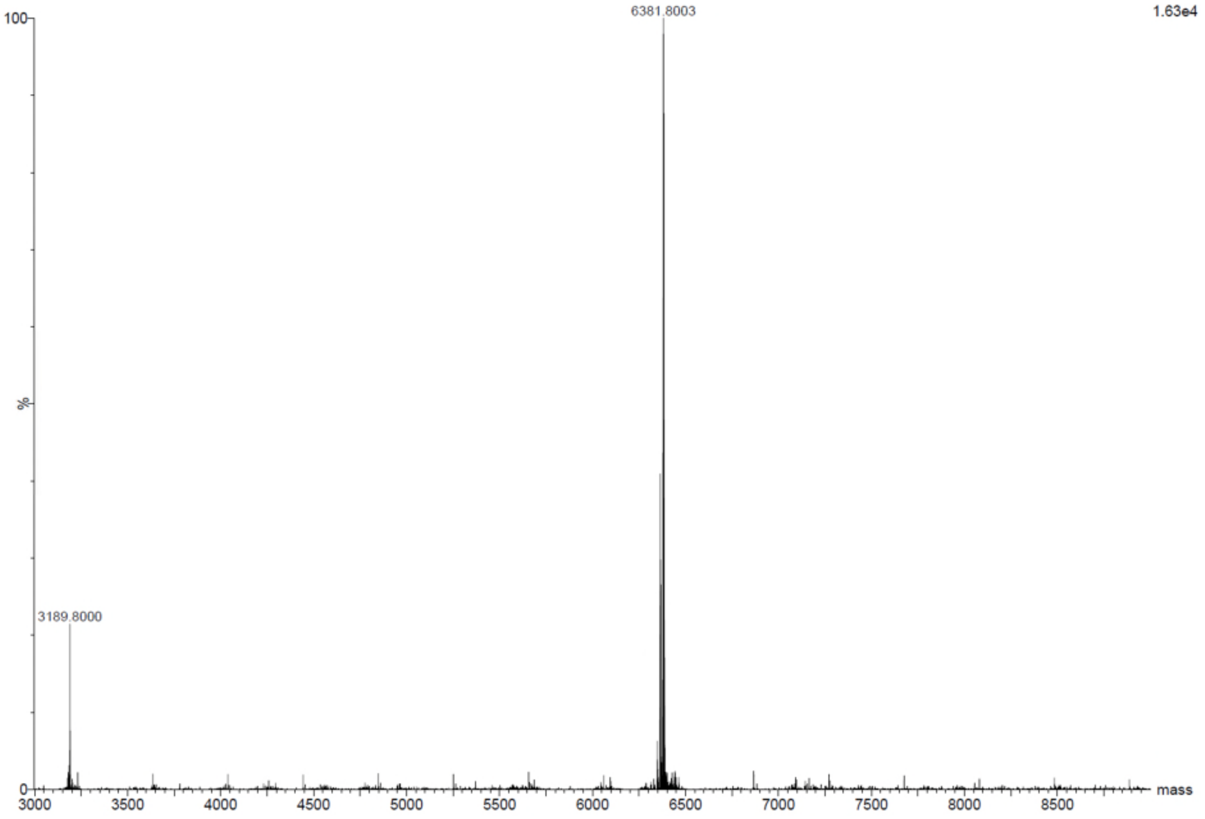

Supplementary Figure S84: Mass spectrum (ES-) of **Glu A1**. Required **6382.16** Da, found **6381.80** Da. y-axis = relative intensity (%), x-axis = mass in Da.

Glu A2	CCU CUU ACC UCA GUU ACA	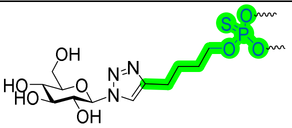

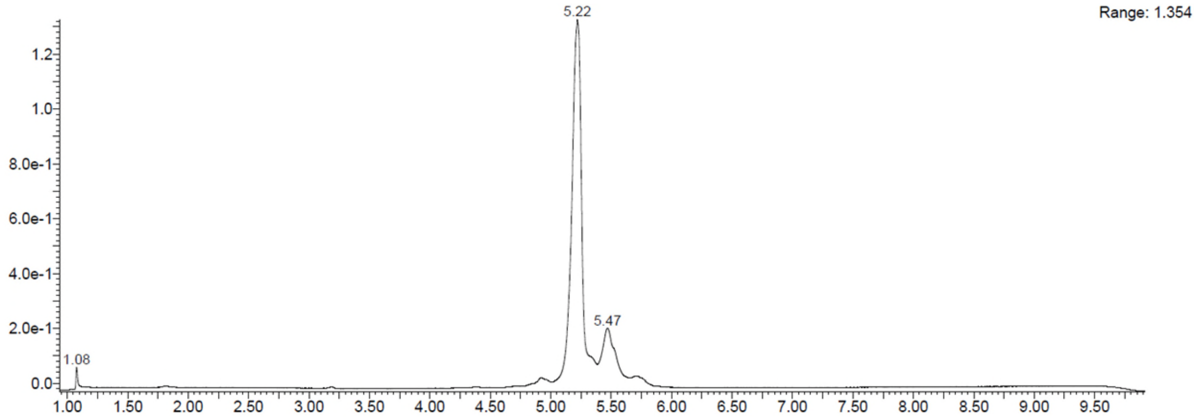

Supplementary Figure S85: Reverse-phase UPLC of **Glu A2** (UV absorbance at 260 nm vs time in min).

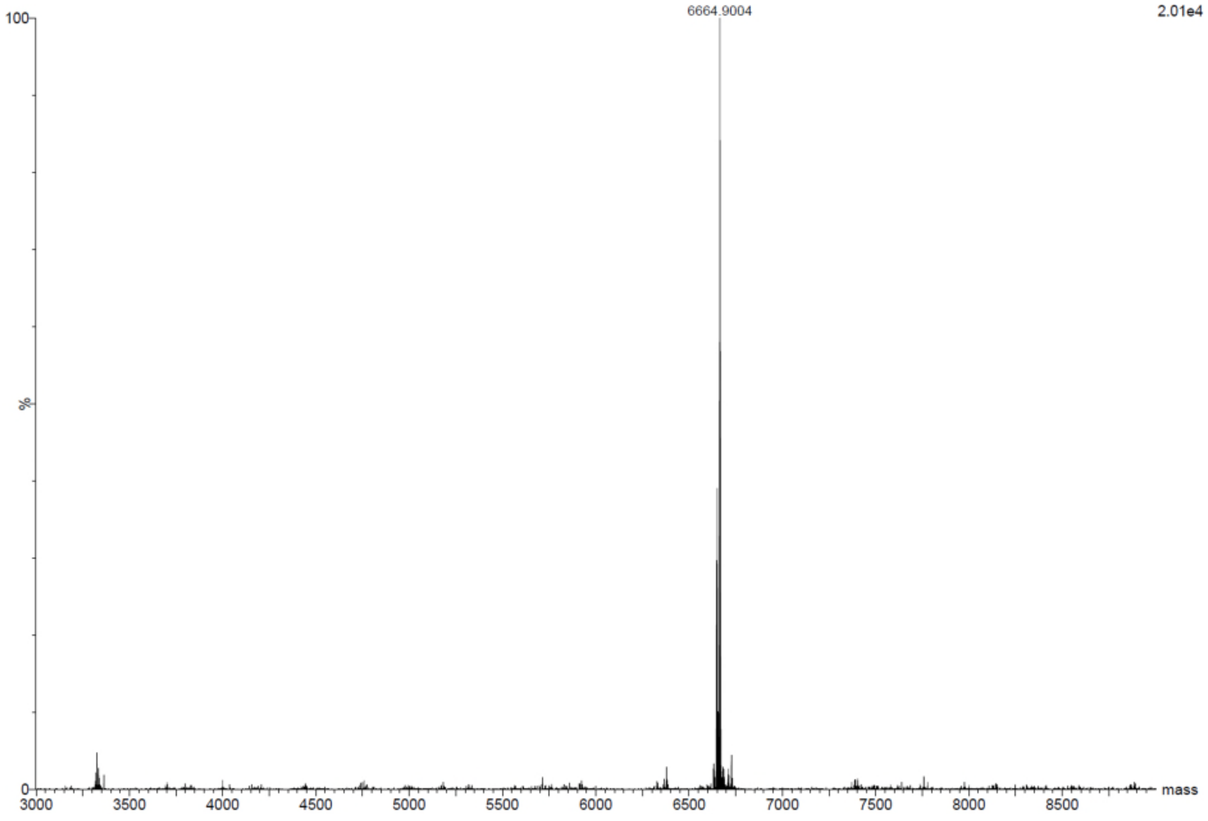

Supplementary Figure S86: Mass spectrum (ES-) of **Glu A2**. Required **6665.45** Da, found **6664.90** Da. y-axis = relative intensity (%), x-axis = mass in Da.

2.5.13 Gluc T3, Gal T3 and Lac T3

Gluc T3	CCU CUT ACC TCA GUT ACA	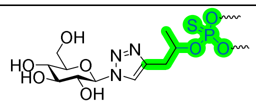

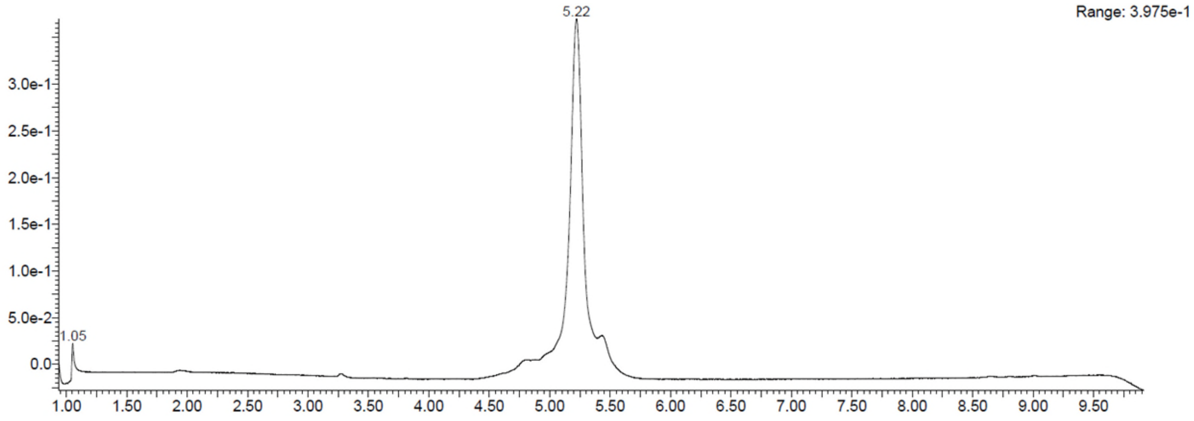

Supplementary Figure S87: Reverse-phase UPLC of **Gluc T3** (UV absorbance at 260 nm vs time in min).

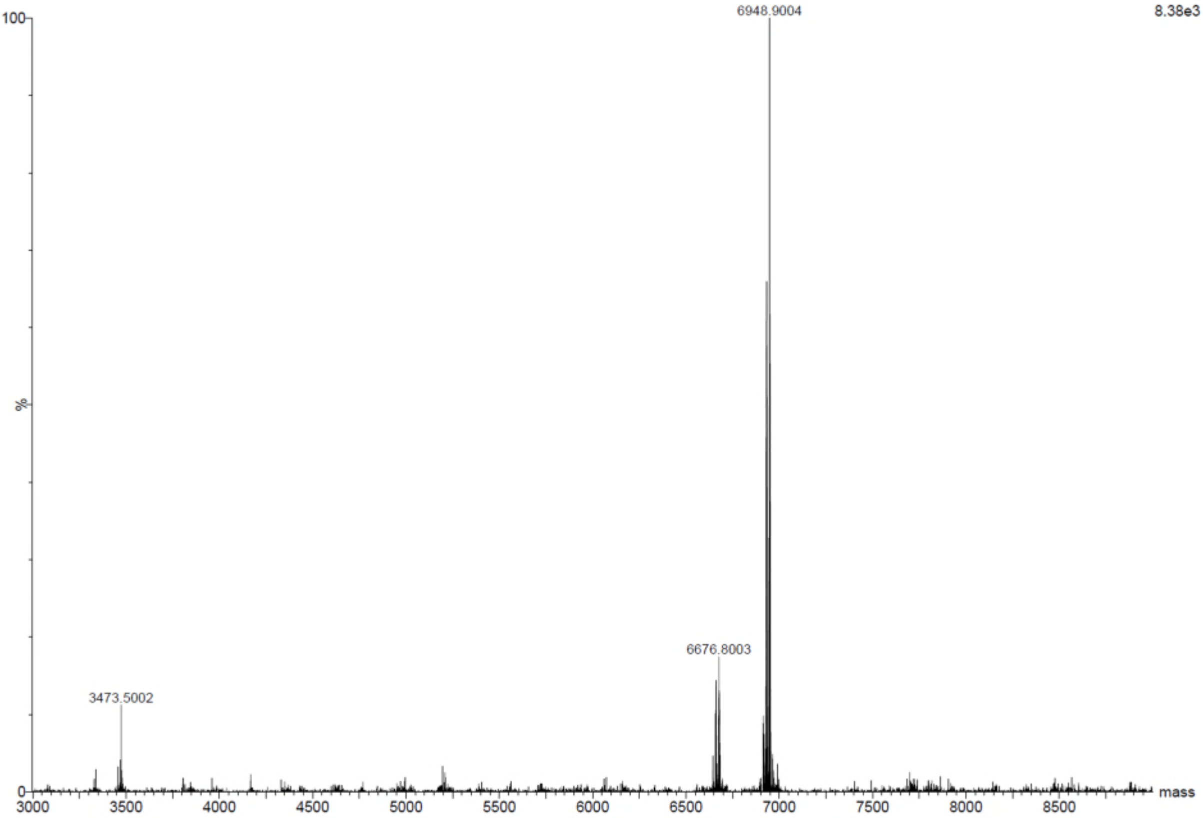

Supplementary Figure S88: Mass spectrum (ES-) of **Gluc T3**. Required **6948.73** Da, found **6948.90** Da. y-axis = relative intensity (%), x-axis = mass in Da.

Gal T3	CCU CUT ACC TCA GUT ACA	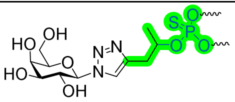

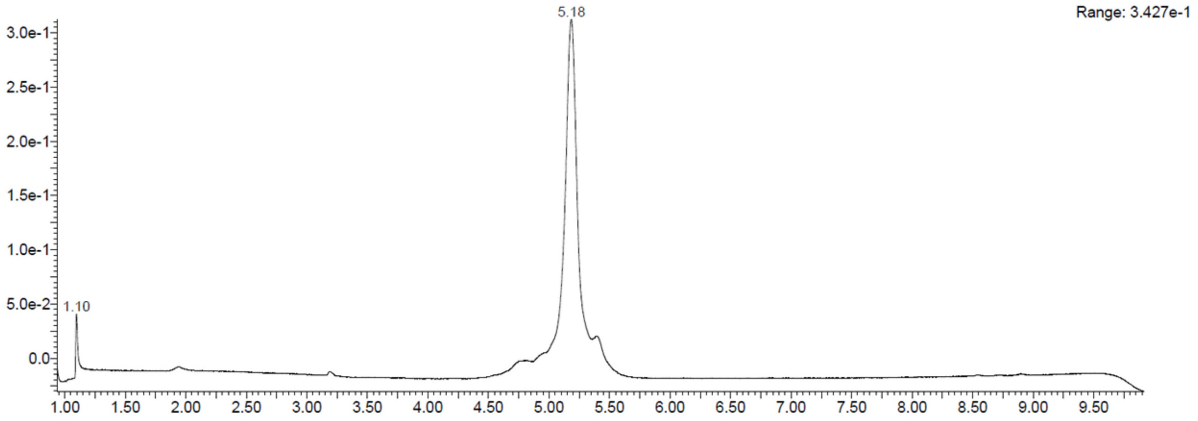

Supplementary Figure S89: Reverse-phase UPLC of **Gal T3** (UV absorbance at 260 nm vs time in min).

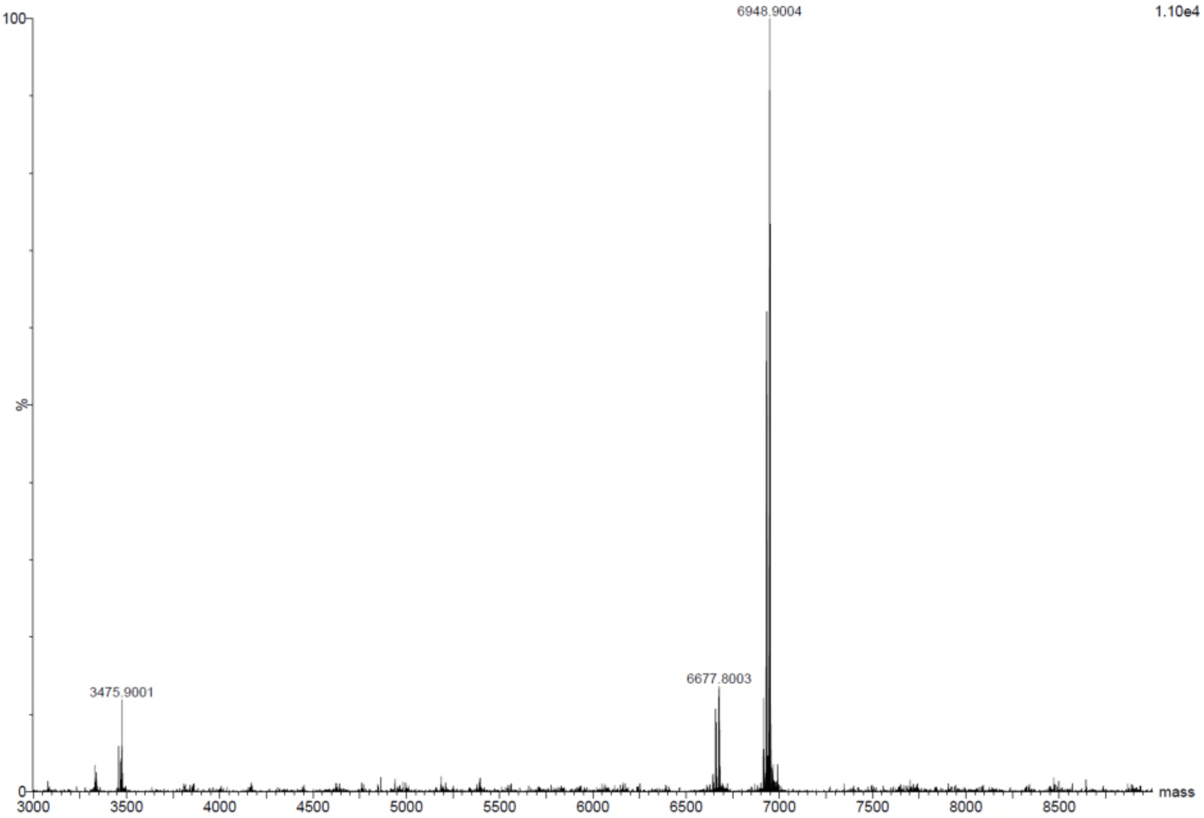

Supplementary Figure S90: Mass spectrum (ES-) of **Gal T3**. Required **6948.73** Da, found **6948.90** Da. y-axis = relative intensity (%), x-axis = mass in Da.

Lac T3	CCU CUT ACC TCA GUT ACA	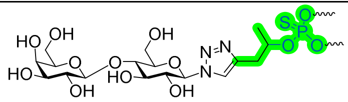

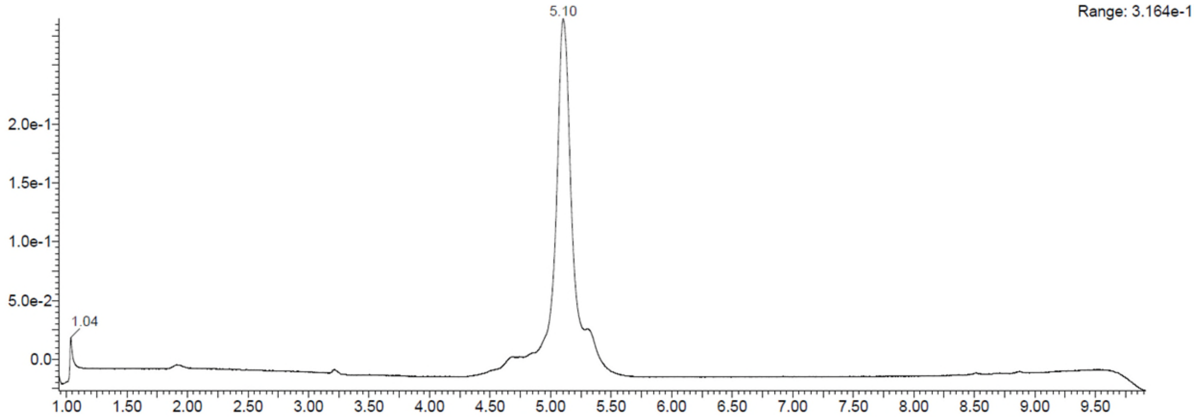

Supplementary Figure S91: Reverse-phase UPLC of **Lac T3** (UV absorbance at 260 nm vs time in min).

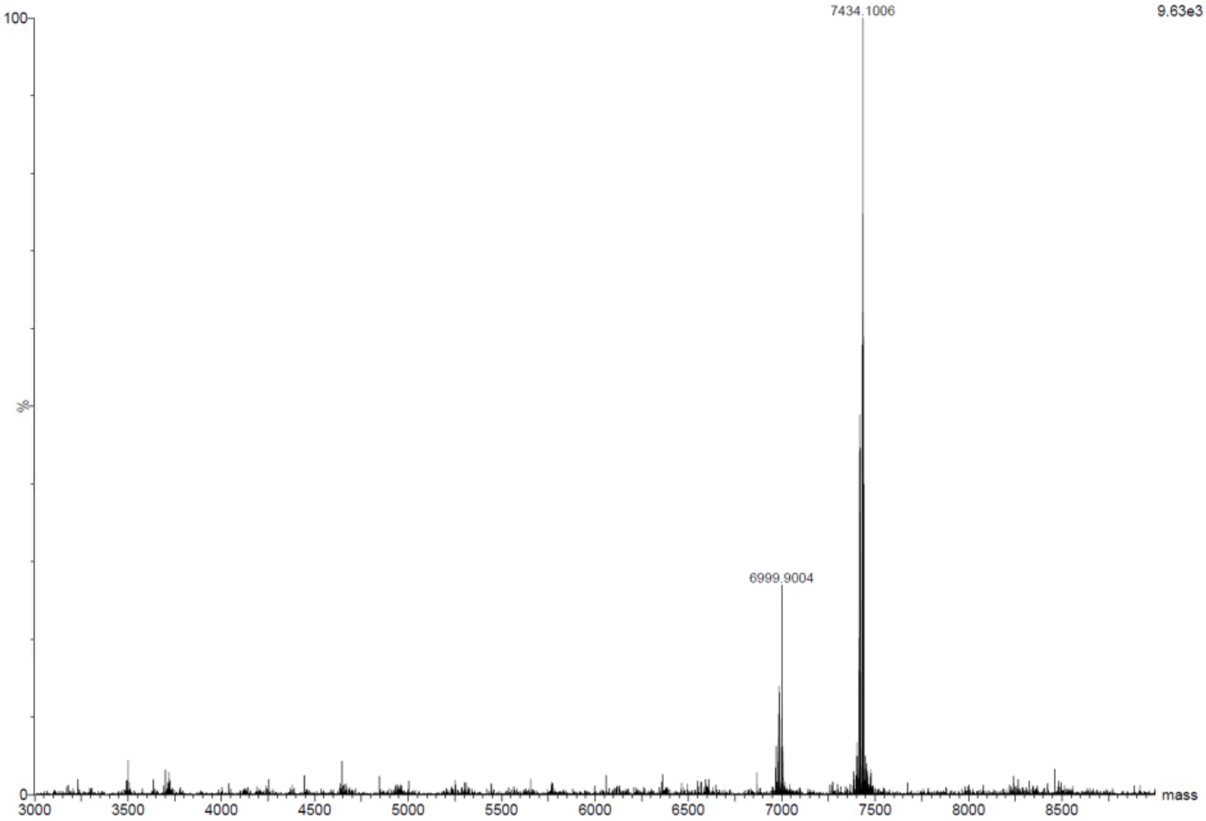

Supplementary Figure S92: Mass spectrum (ES-) of **Lac T3**. Required **7435.15** Da, found **7434.10** Da. y-axis = relative intensity (%), x-axis = mass in Da.

2.5.14 Lys T3, Leu T3, Phe T3 and Val T3

Lys T3	CCU CUT ACC TCA GUT ACA	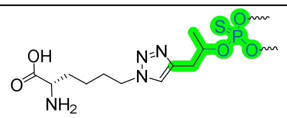

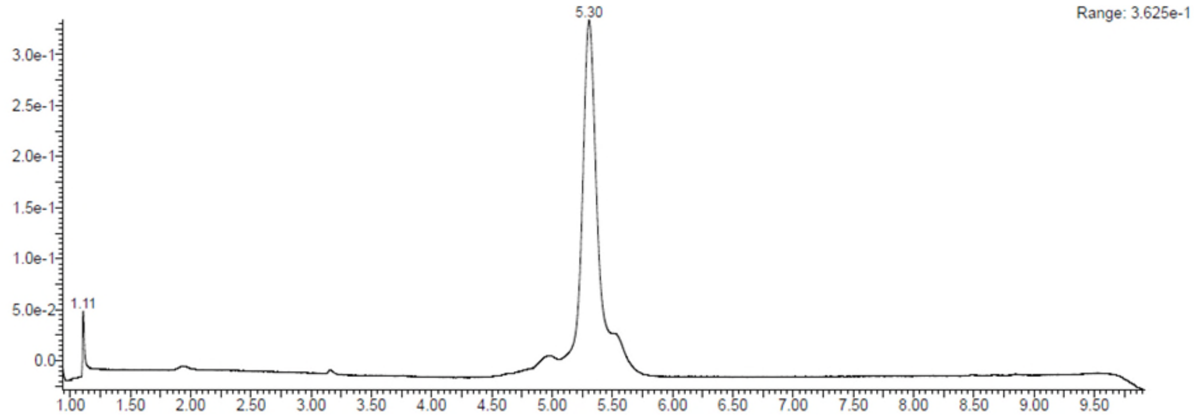

Supplementary Figure S93: Reverse-phase UPLC of **Lys T3** (UV absorbance at 260 nm vs time in min).

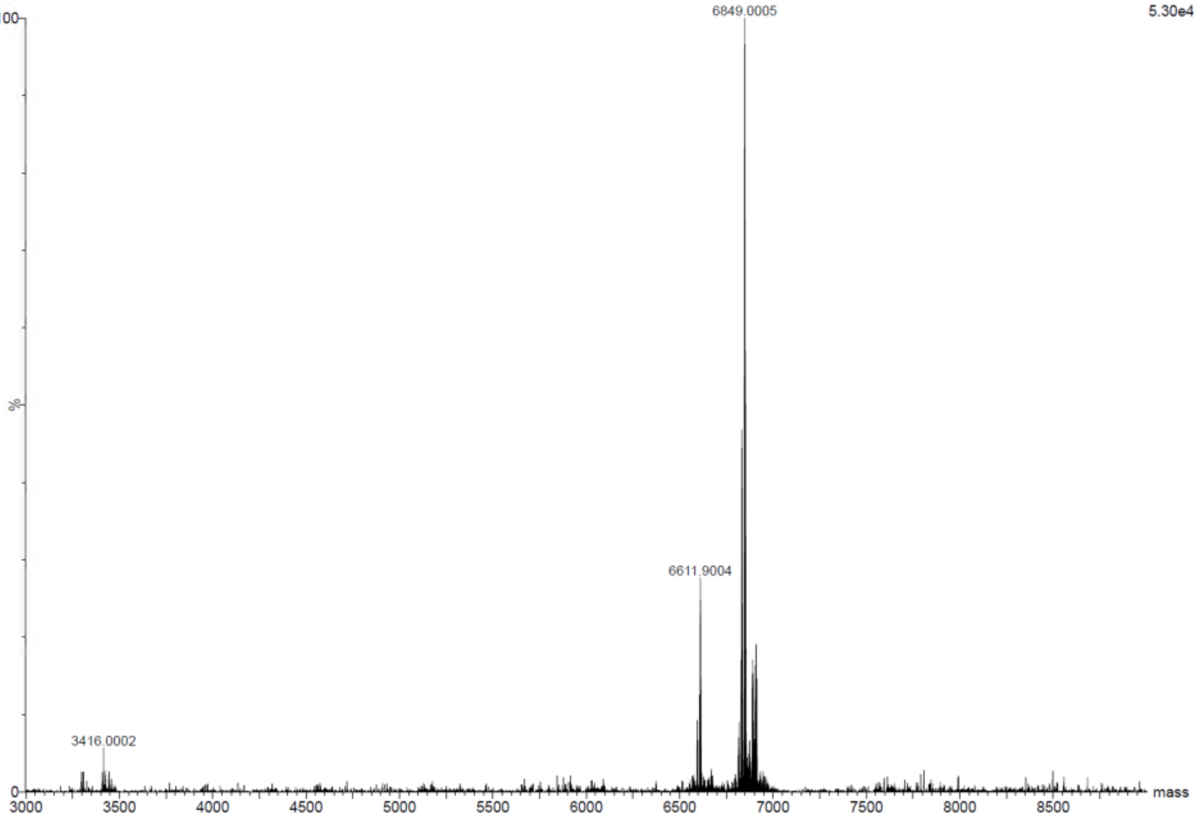

Supplementary Figure S94: Mass spectrum (ES-) of **Lys T3**. Required **6849.78** Da, found **6149.00** Da. y-axis = relative intensity (%), x-axis = mass in Da.

Leu T3	CCU CUT ACC TCA GUT ACA	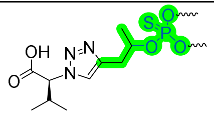

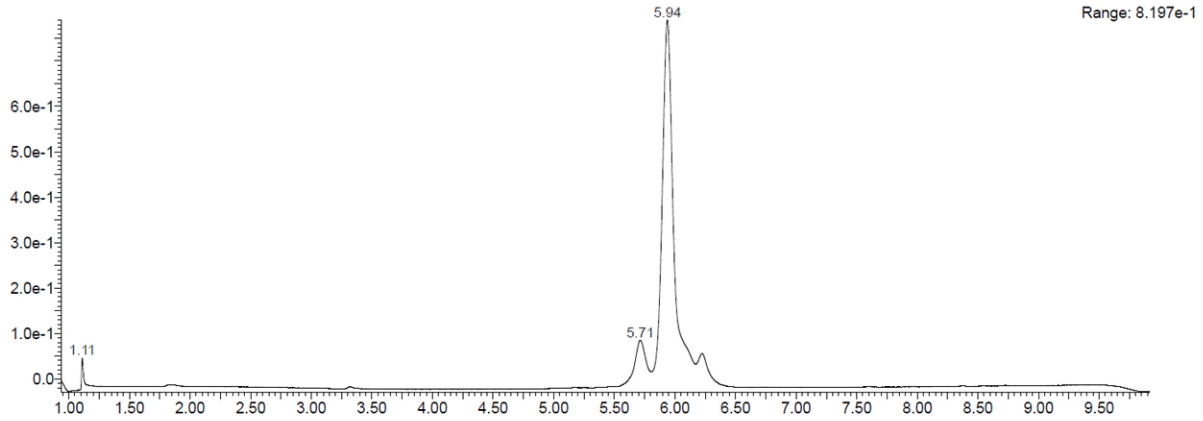

Supplementary Figure S95: Reverse-phase UPLC of **Leu T3** (UV absorbance at 260 nm vs time in min).

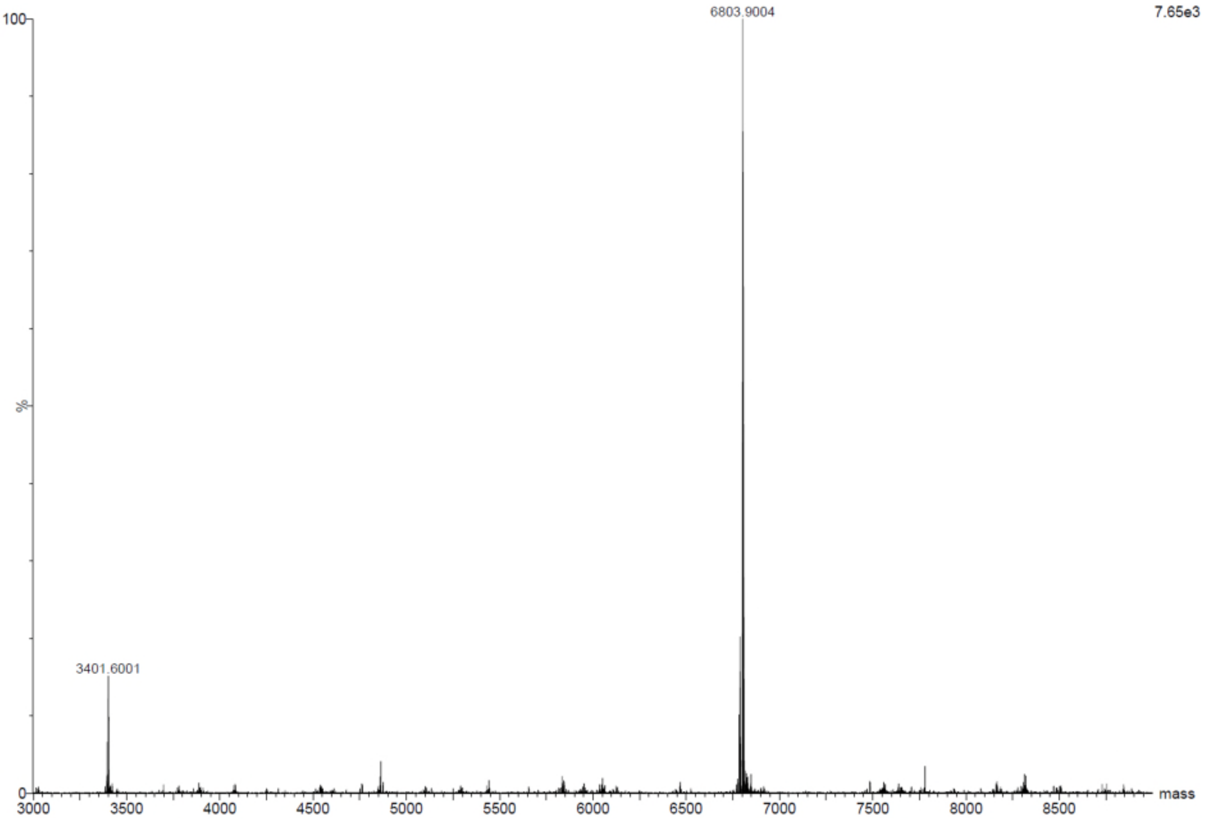

Supplementary Figure S96: Mass spectrum (ES-) of **Leu T3**. Required **6804.74** Da, found **6803.90** Da. y-axis = relative intensity (%), x-axis = mass in Da.

Phe T3	CCU CUT ACC TCA GUT ACA	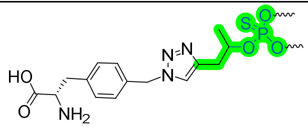

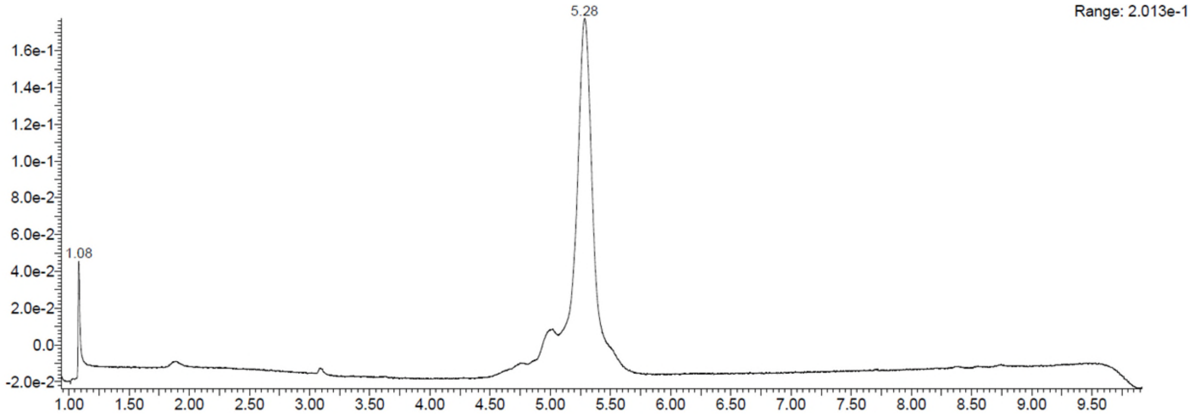

Supplementary Figure S97: Reverse-phase UPLC of **Phe T3** (UV absorbance at 260 nm vs time in min).

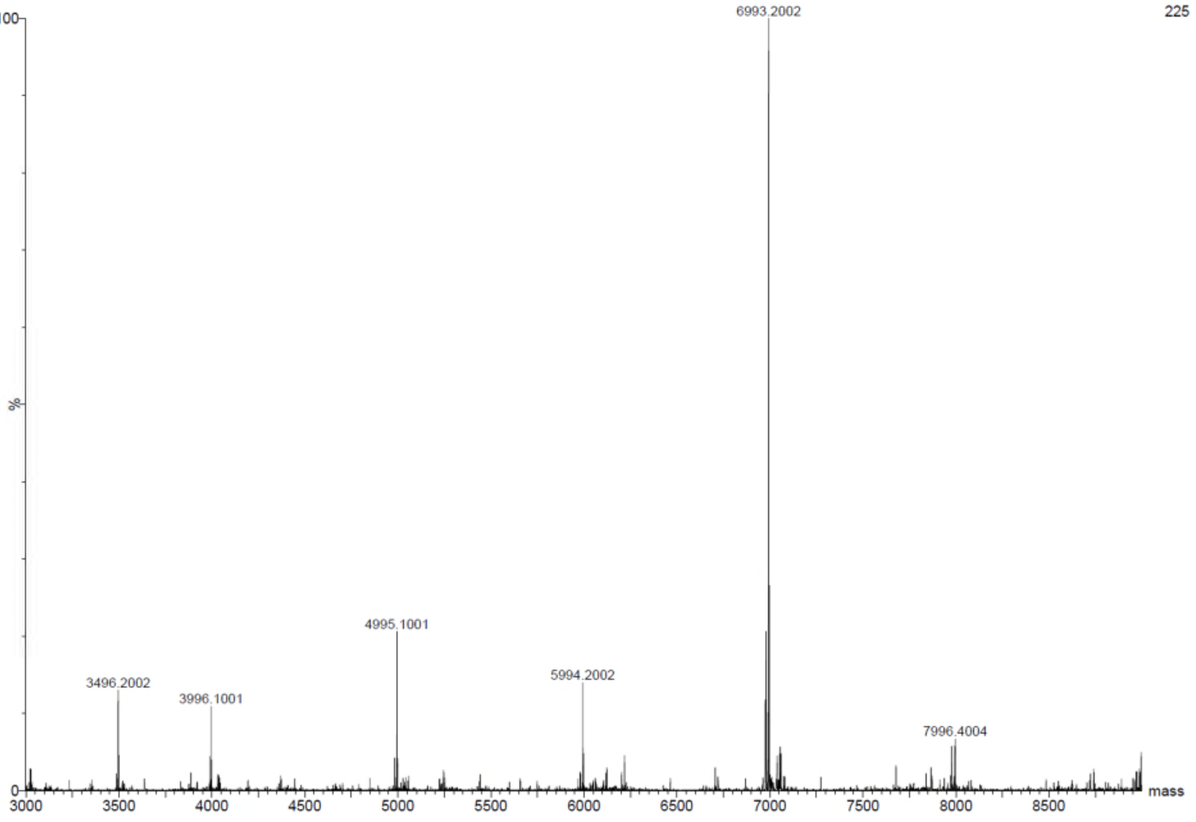

Supplementary Figure S98: Mass spectrum (ES-) of **Phe T3**. Required **6993.92** Da, found **6993.20** Da. y-axis = relative intensity (%), x-axis = mass in Da.

Val T3	CCU CUT ACC TCA GUT ACA	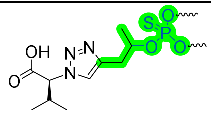

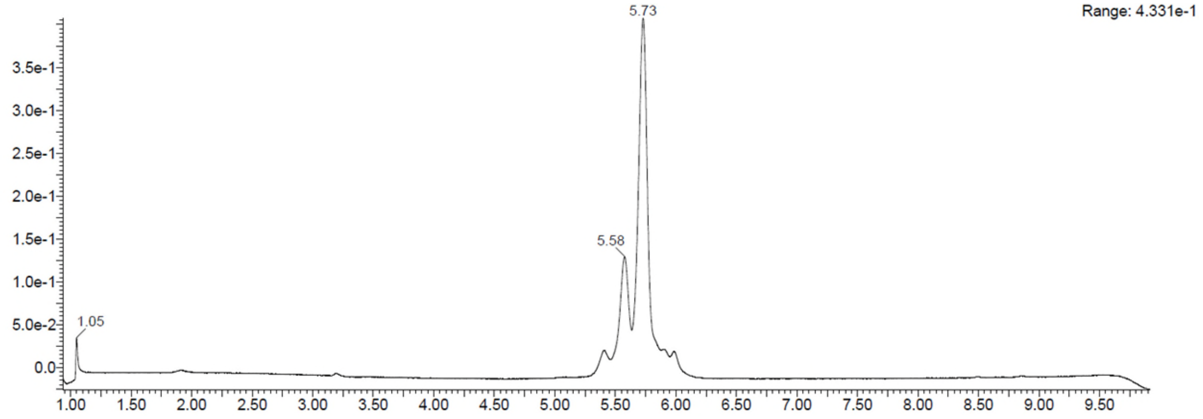

Supplementary Figure S99: Reverse-phase UPLC of **Val T3** (UV absorbance at 260 nm vs time in min).

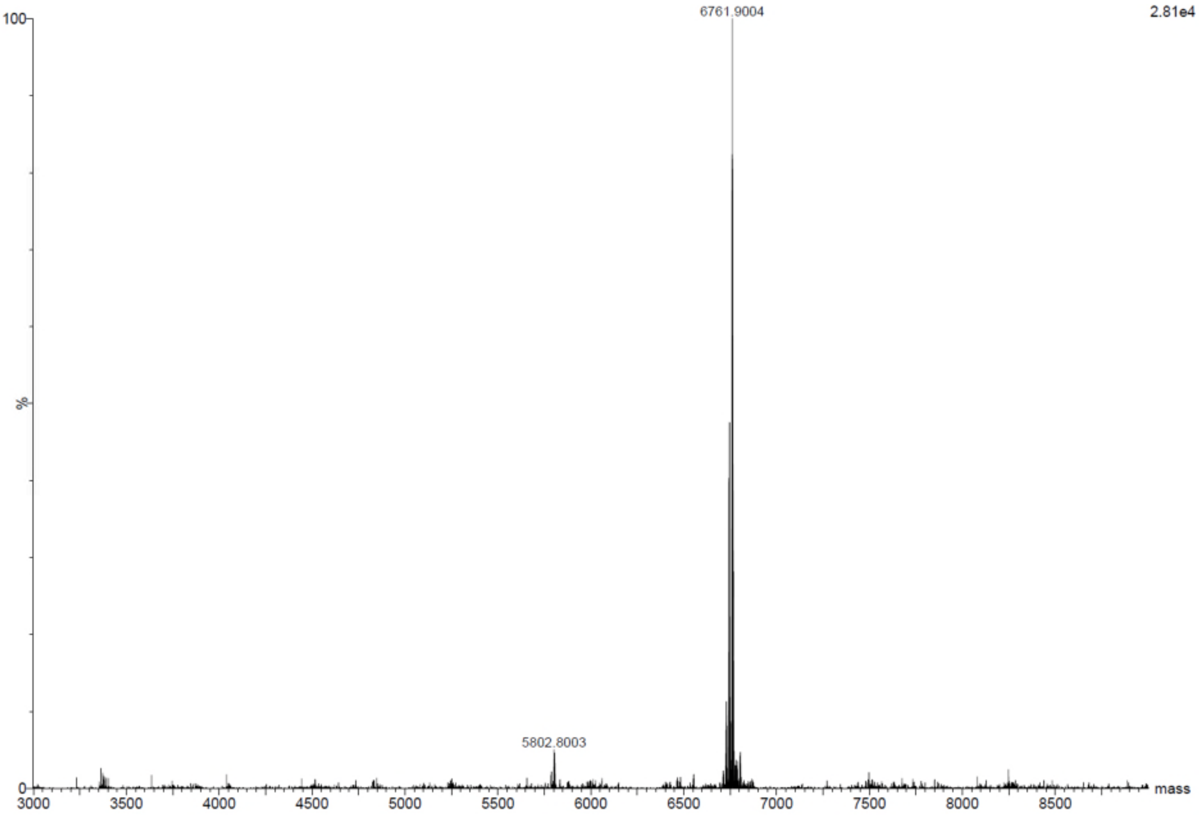

Supplementary Figure S100: Mass spectrum (ES-) of **Val T3**. Required **6168.97** Da, found **6168.50** Da. y-axis = relative intensity (%), x-axis = mass in Da.

2.5.15 Sper T1 and Sper T2

Sper T1	CCU CUU ACC UCA GUT ACA	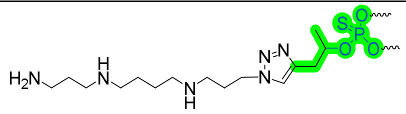

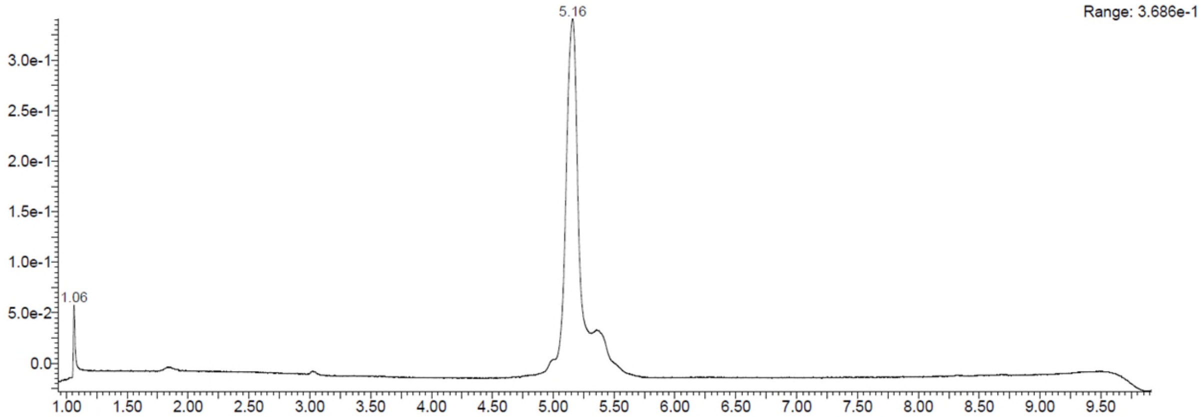

Supplementary Figure S101: Reverse-phase UPLC of **Sper T1** (UV absorbance at 260 nm vs time in min).

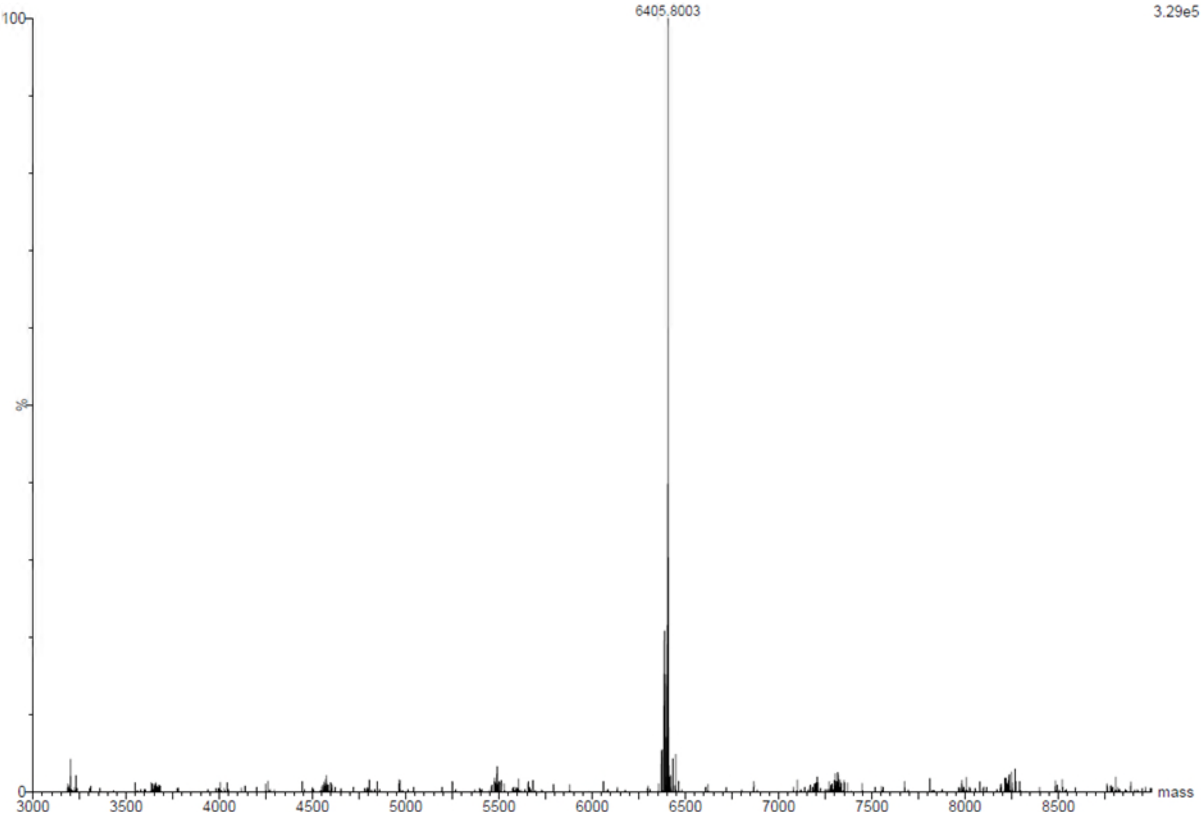

Supplementary Figure S102: Mass spectrum (ES-) of **Sper T1**. Required **6433.39** Da, found **6405.82** Da. y-axis = relative intensity (%), x-axis = mass in Da.

Sper T2	CCU CUT ACC UCA GUT ACA	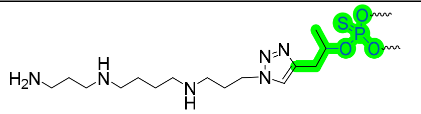

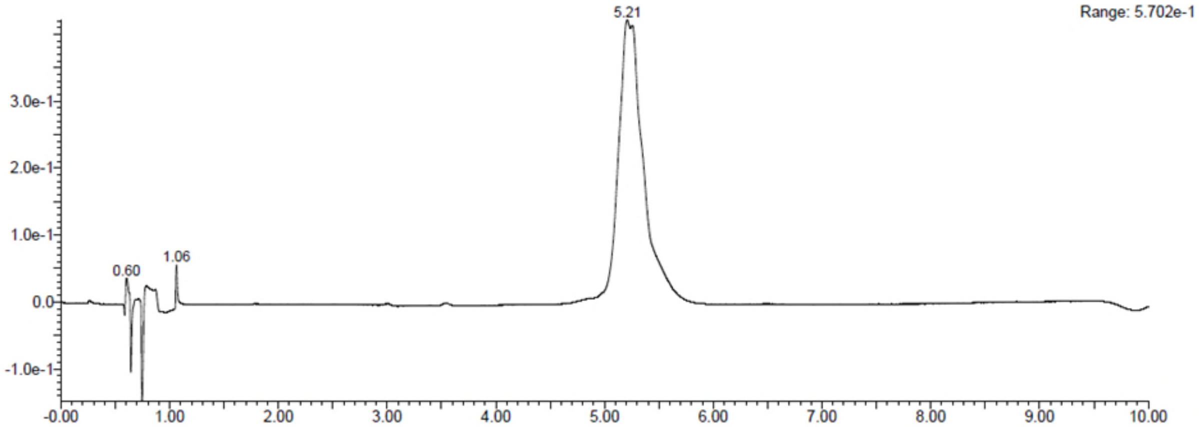

Supplementary Figure S103: Reverse-phase UPLC of **Sper T2** (UV absorbance at 260 nm vs time in min).

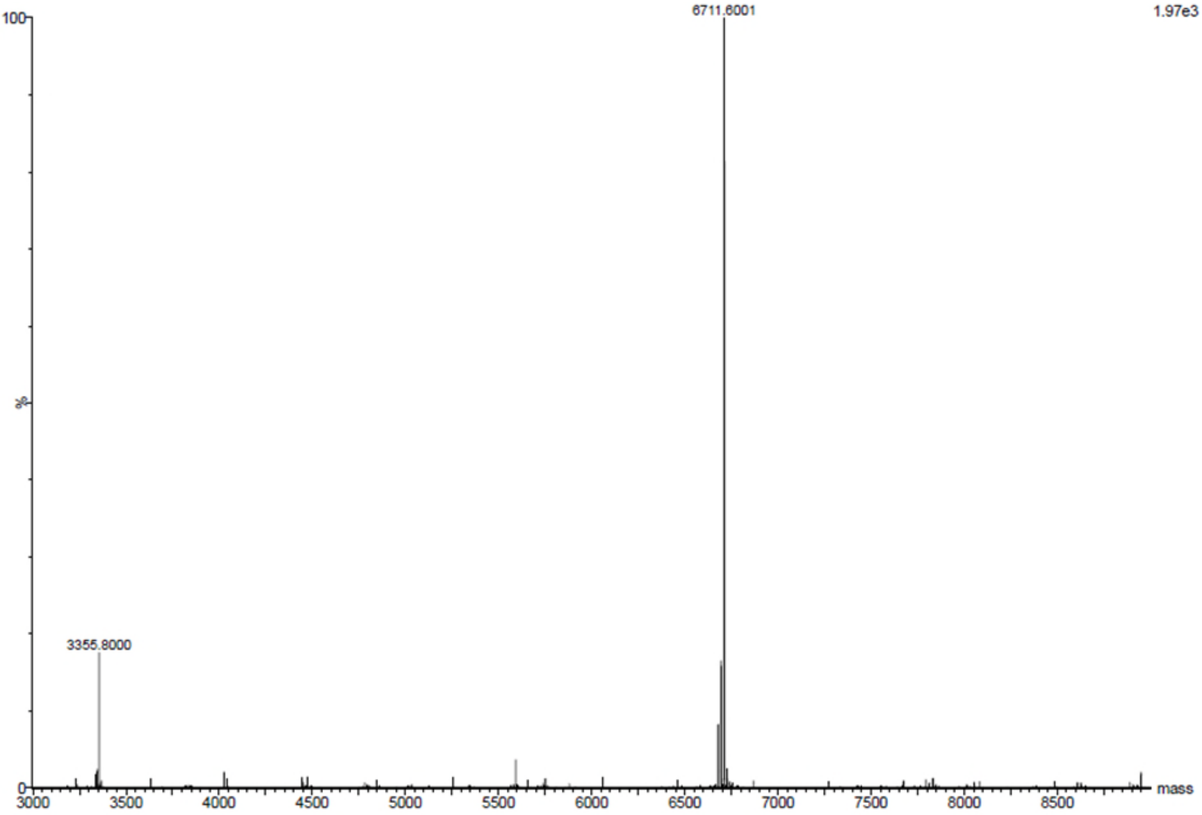

Supplementary Figure S104: Mass spectrum (ES-) of **Sper T2**. Required **6711.79** Da, found **6711.60** Da. y-axis = relative intensity (%), x-axis = mass in Da.

2.5.16 TO A1, TO A2, TO T1 and TO T2

TO A1	CCU CUU ACC UCA GUU ACA	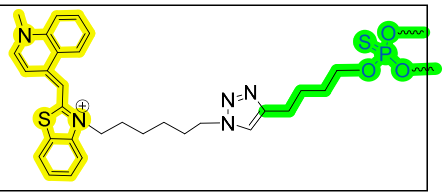

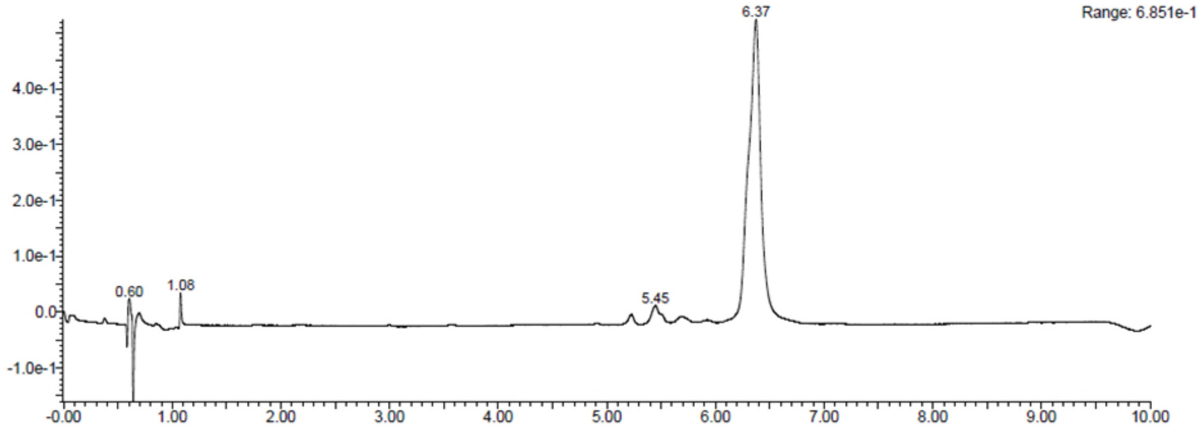

Supplementary Figure S105: Reverse-phase UPLC of **TO A1** (UV absorbance at 260 nm vs time in min).

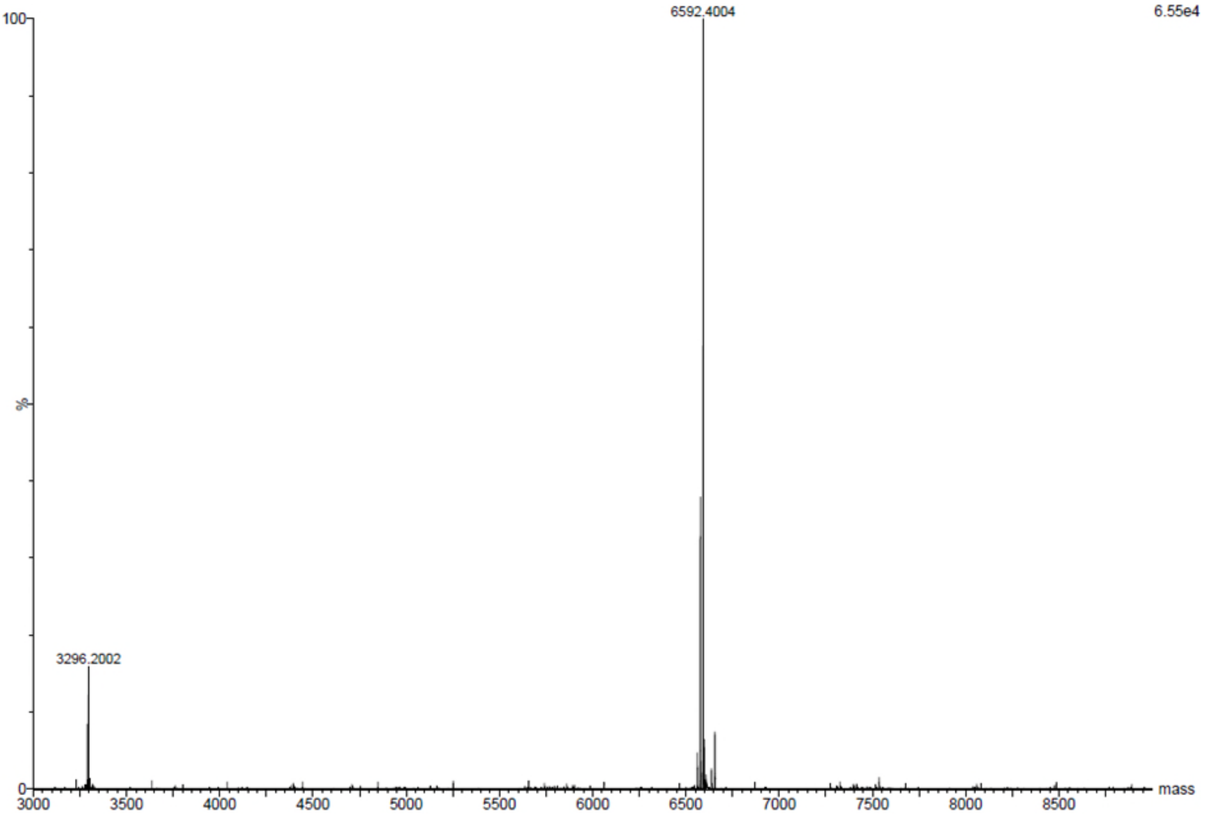

Supplementary Figure S106: Mass spectrum (ES-) of **TO A1**. Required **6593.56** Da, found **6592.40** Da. y-axis = relative intensity (%), x-axis = mass in Da.

TO A2	CCU CUU ACC UCA GUU ACA	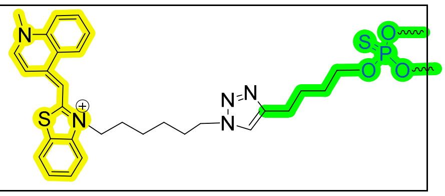

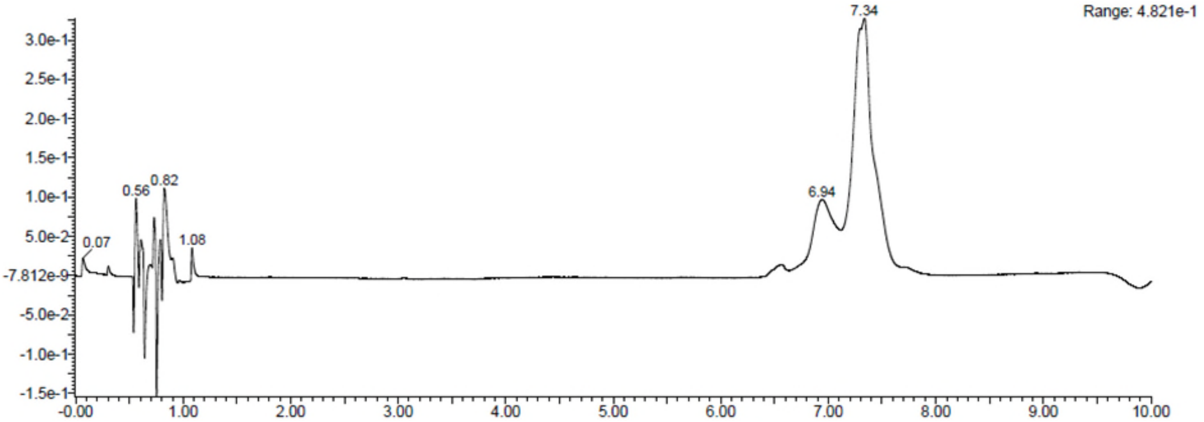

Supplementary Figure S107: Reverse-phase UPLC of **TO A2** (UV absorbance at 260 nm vs time in min).

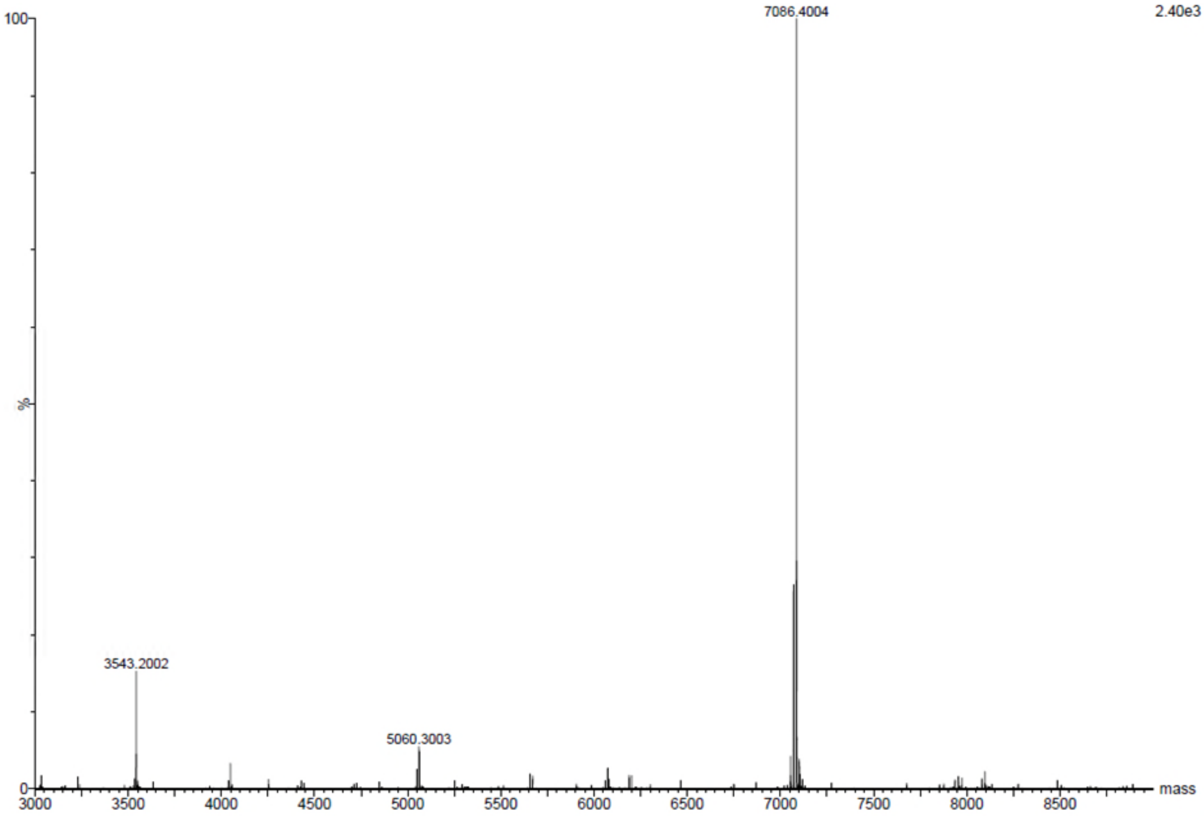

Supplementary Figure S108: Mass spectrum (ES-) of **TO A2**. Required **7088.24** Da, found **7086.40** Da. y-axis = relative intensity (%), x-axis = mass in Da.

TO T1	CCU CUU ACC UCA GUT ACA	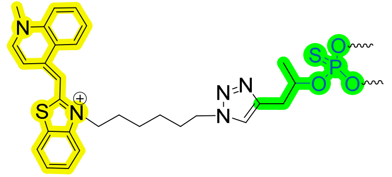

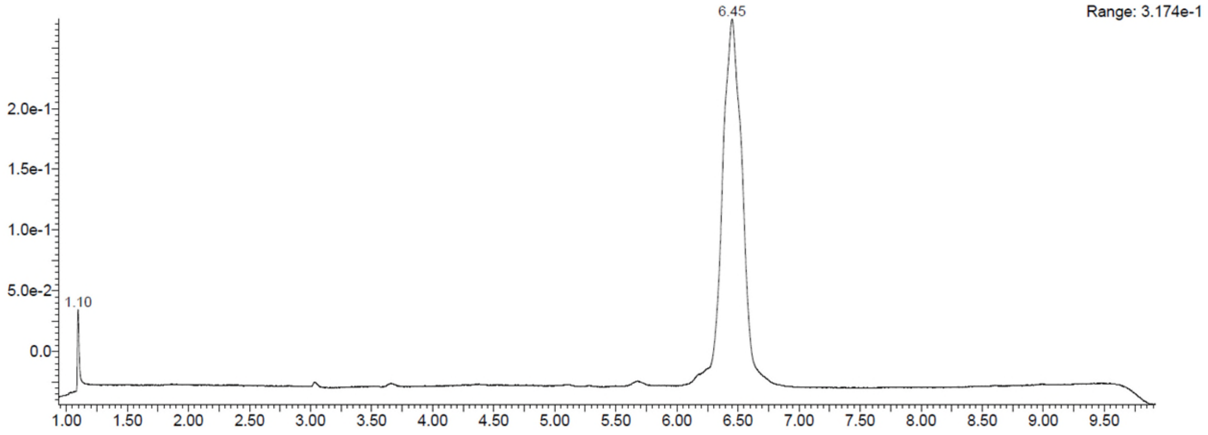

Supplementary Figure S109: Reverse-phase UPLC of **TO T1** (UV absorbance at 260 nm vs time in min).

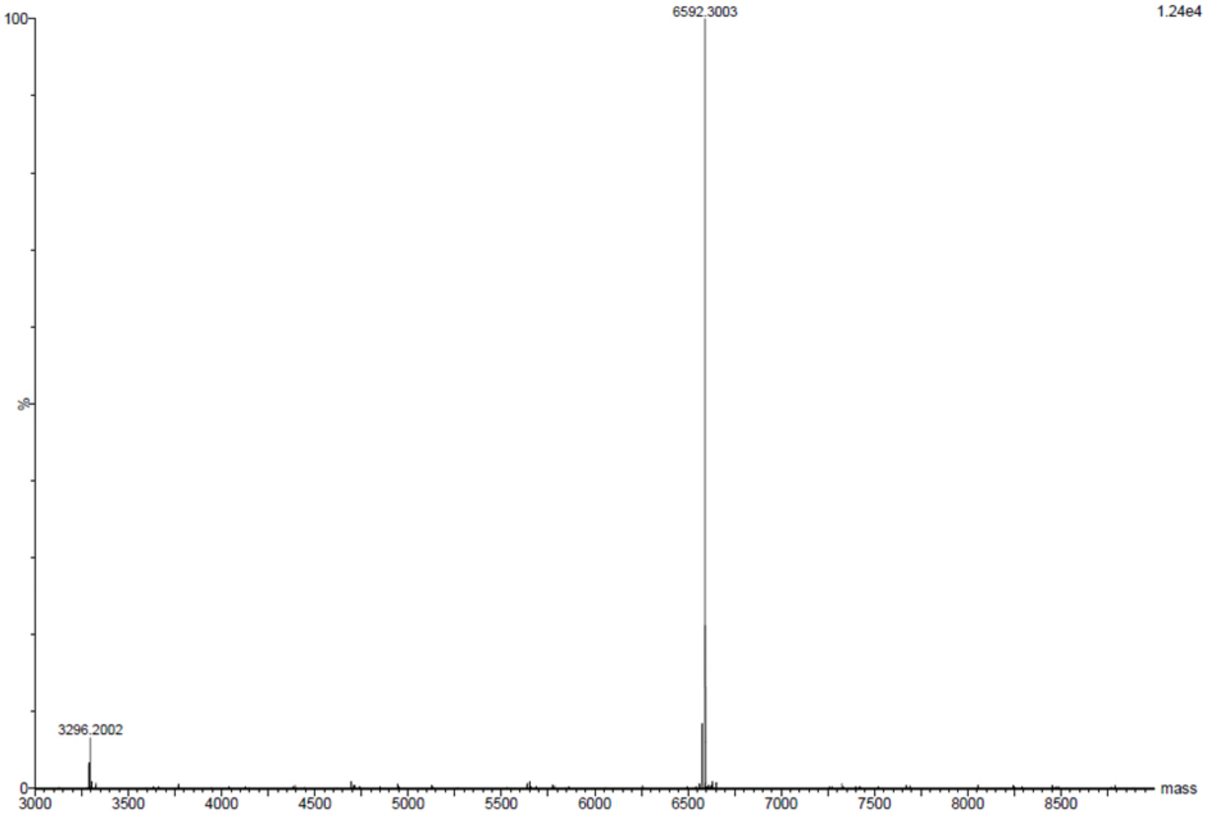

Supplementary Figure S110: Mass spectrum (ES-) of **TO T1**. Required **6593.56** Da, found **6592.30** Da. y-axis = relative intensity (%), x-axis = mass in Da.

TO T2	CCU CUT ACC UCA GUT ACA	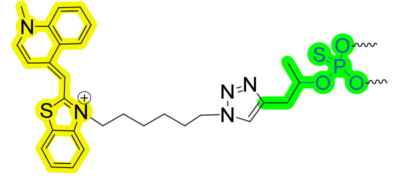

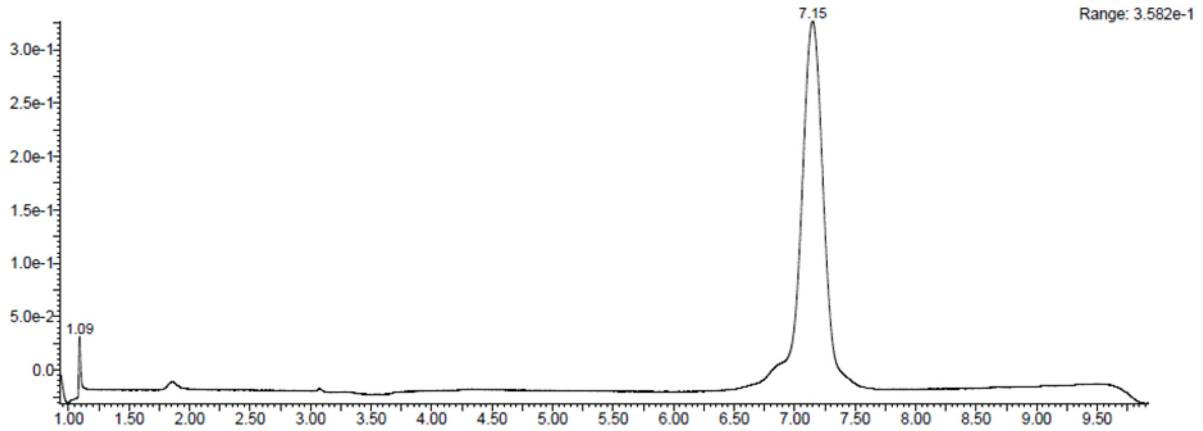

Supplementary Figure S111: Reverse-phase UPLC of **TO T2** (UV absorbance at 260 nm vs time in min).

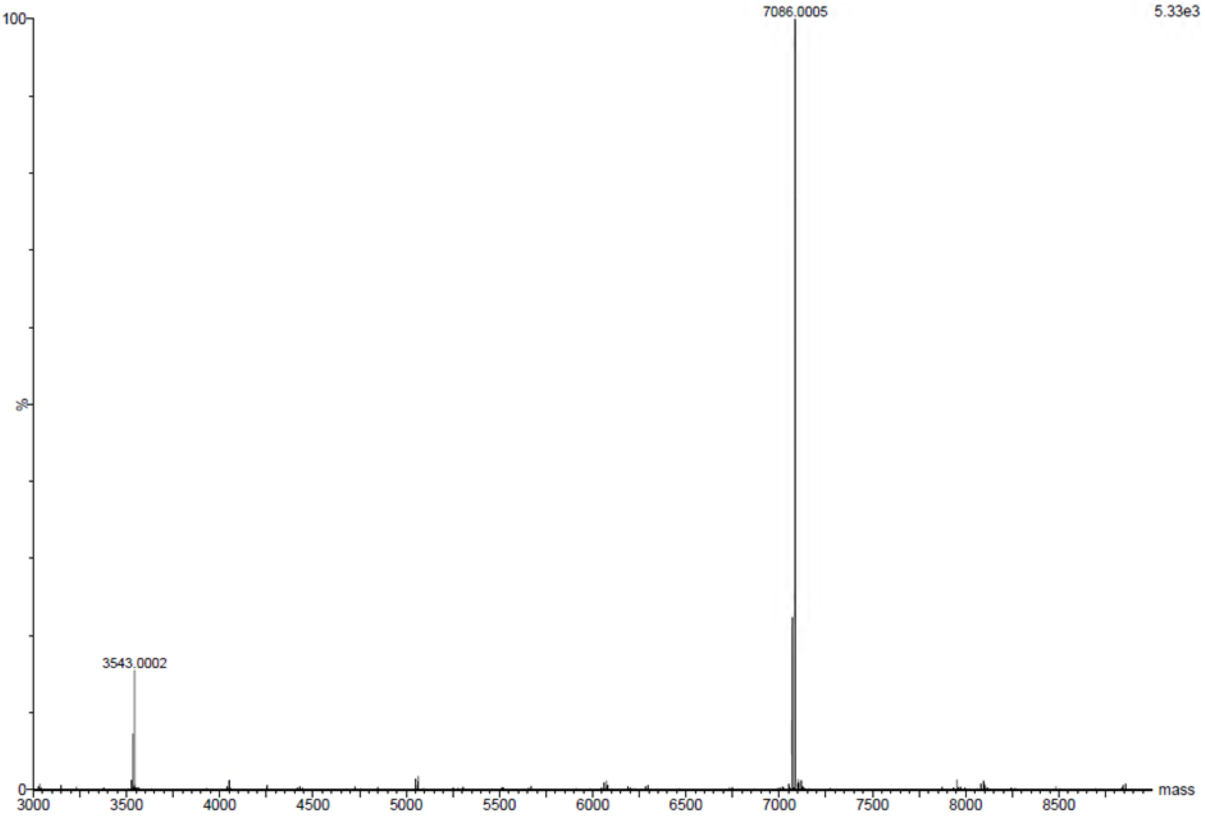

Supplementary Figure S112: Mass spectrum (ES-) of **TO T2**. Required **7088.24** Da, found **7086.00** Da. y-axis = relative intensity (%), x-axis = mass in Da.

3.0 UV meting experiments

UV melting experiments were performed using a Cary 4000 scan UV-vis spectrophotometer. Each oligonucleotide and its complementary DNA or RNA (2 µM final concentration) was dissolved in 1 mL of 10 mM phosphate buffer containing 100 mM NaCl (for DNA Target) and 25 mM NaCl (for RNA target) at pH 7.0. The samples were first denatured by heating to 85 °C (10 °C/min) and then annealed by slowly cooling to 20 °C. (1 °C/min). Six successive cycles of heating and cooling were performed at a gradient of 1 °C/min whilst recording the change in UV absorbance at 260 nm. The built-in Cary WinUV 3.0 software was then used to calculate the melting temperature from the first derivative of the melting curves. The curves shown are representative of three independent repeats, each consisting of at least two technical repeats. Tm values are an average of three experiments with an error of ±0.50 ^{o}C.

3.1 Butyl T2, Butyl T3 and Butyl T4

Supplementary Table T4: Duplex melting temperatures (Tm) in °C of **Butyl T2, Butyl T3 and Butyl T4** against complementary DNA and RNA. Nucleotides in black have 2´-OMe ribose sugars and phosphorothioate internucleoside linkages. Nucleotides in red are locked nucleic acid alkyl phosphothiotriesters.

**Oligonucleotide**	**Sequence (5´→3´)**	**DNA target Tm (ΔTm)**	**RNA target Tm (ΔTm)**
Butyl T2	CCU CUT ACC UCA GUT ACA	54.9 (+6.4)	67.0 (+5.7)
Butyl T3	CCU CUT ACC TCA GUT ACA	57.8 (+9.2)	69.8 (+8.5)
Butyl T4	CCT CUT ACC TCA GUT ACA	61.9 (+13.4)	74.3 (+13.0)

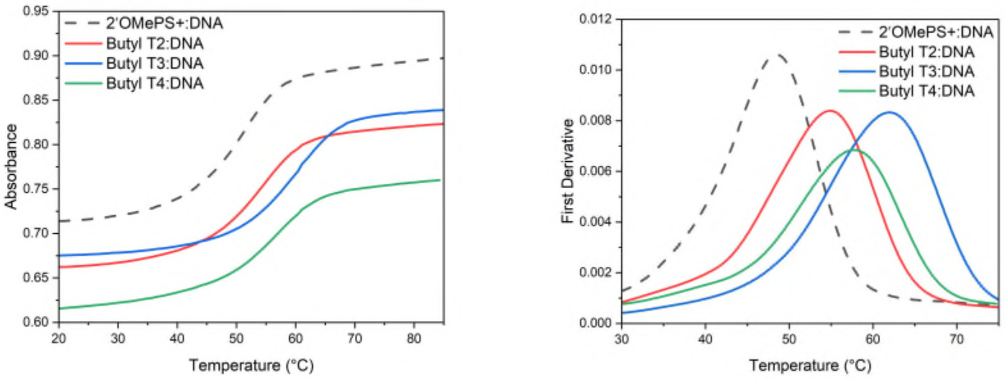

Supplementary Figure S113: UV melting studies of **Butyl T2, Butyl T3 and Butyl T4** against complementary DNA. *Left:* Representative UV melting curves measured using 2 µM of each oligonucleotide in 10 mM Na-phosphate buffer, 100 mM NaCl, pH = 7.0; *Right:*1st derivative of melting curves.

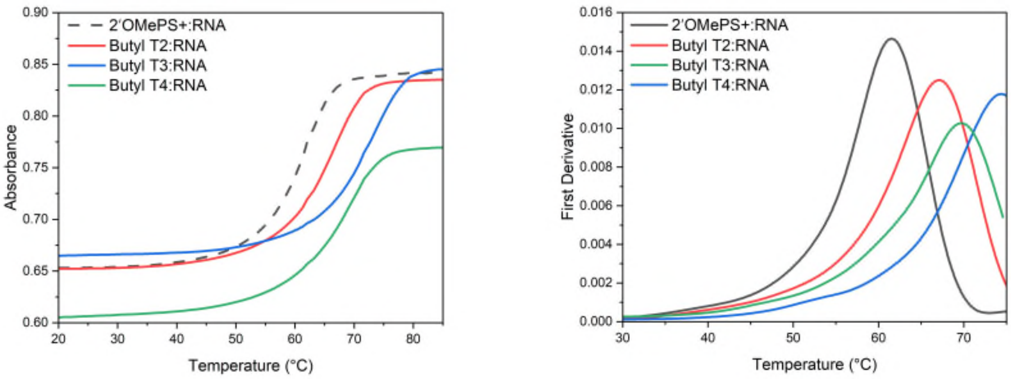

Supplementary Figure S114: UV melting studies of **Butyl T2, Butyl T3 and Butyl T4** against complementary RNA. *Left:* Representative UV melting curves measured using 2 µM of each oligonucleotide in 10 mM Na-phosphate buffer, 25 mM NaCl, pH = 7.0; *Right:* 1st derivative of melting curves.

3.2 CF_3 T1, CF_3 T2, CF_3 T3 and CF_3 T4

Supplementary Table T5: Duplex melting temperatures (Tm) in °C of **CF_3 T1, CF_3 T2, CF_3 T3 and CF_3 T4** against complementary DNA and RNA. Nucleotides in black have 2´-OMe ribose sugars and phosphorothioate internucleoside linkages. Nucleotides in red are locked nucleic acid alkyl phosphothiotriesters.

Oligonucleotide	Sequence (5´→3´)	DNA target Tm (ΔTm)	RNA target Tm (ΔTm)
CF_3 T1	CCU CUU ACC UCA GUT ACA	50.2 (+1.7)	62.7 (+1.4)
CF_3 T2	CCU CUT ACC UCA GUT ACA	52.5 (+4.0)	64.8 (+3.5)
CF_3 T3	CCU CUT ACC TCA GUT ACA	53.8 (+5.3)	66.8 (+5.5)
CF_3 T4	CCT CUT ACC TCA GUT ACA	54.7 (+6.2)	67.6 (+6.3)

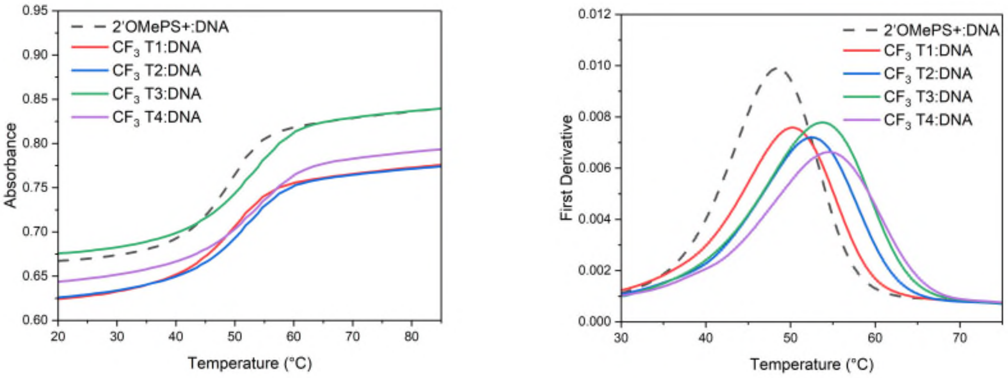

Supplementary Figure S115: UV melting studies of **CF_3 T1, CF_3 T2, CF_3 T3 and CF_3 T4** against complementary DNA. *Left:* Representative UV melting curves measured using 2 µM of each oligonucleotide in 10 mM Na-phosphate buffer, 100 mM NaCl, pH = 7.0; *Right:* 1st derivative of melting curves.

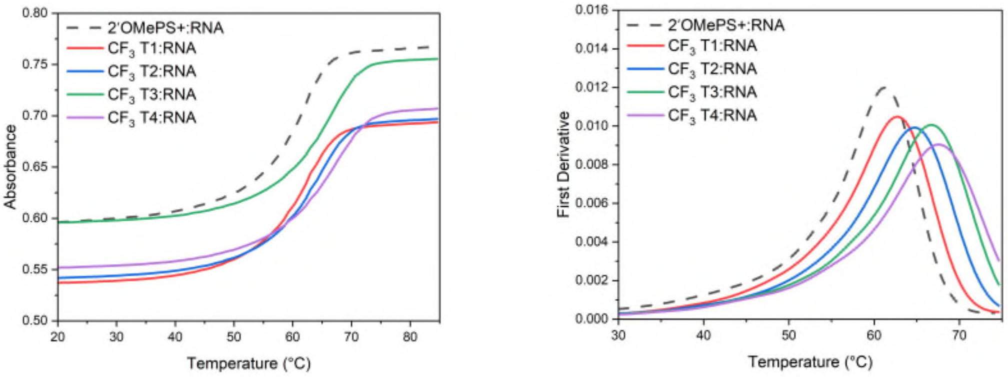

Supplementary Figure S116: UV melting studies of **CF_3 T1, CF_3 T2, CF_3 T3 and CF_3 T4** against complementary RNA. *Left:* Representative UV melting curves measured using 2 µM of each oligonucleotide in 10 mM Na-phosphate buffer, 25 mM NaCl, pH = 7.0; *Right:* 1st derivative of melting curves.

3.3 Hexyl T2, Hexyl T3 and Hexyl T4

Supplementary Table T6: Duplex melting temperatures (Tm) in °C of **Hexyl T2, Hexyl T3 and Hexyl T4** against complementary DNA and RNA. Nucleotides in black have 2´-OMe ribose sugars and phosphorothioate internucleoside linkages. Nucleotides in red are locked nucleic acid alkyl phosphothiotriesters.

**Oligonucleotide**	**Sequence (5´→3´)**	**DNA target Tm (ΔTm)**	**RNA target Tm (ΔTm)**
Hexyl T2	CCU CUT ACC UCA GUT ACA	51.5 (+3.0)	64.7 (+3.4)
Hexyl T3	CCU CUT ACC TCA GUT ACA	54.8 (+6.3)	67.7 (+6.4)
Hexyl T4	CCT CUT ACC TCA GUT ACA	58.0 (+9.5)	71.6 (+10.3)

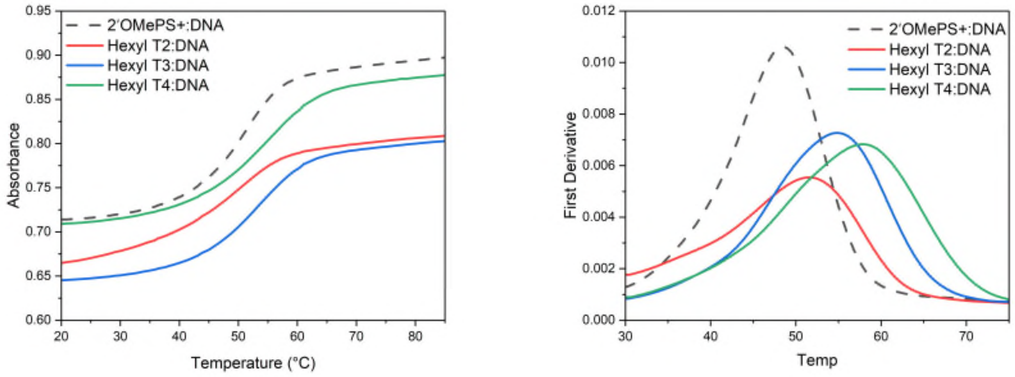

Supplementary Figure S117: UV melting studies of **Hexyl T2, Hexyl T3 and Hexyl T4** against complementary DNA. *Left:* Representative UV melting curves measured using 2 µM of each oligonucleotide in 10 mM Na-phosphate buffer, 100 mM NaCl, pH = 7.0; *Right:*1st derivative of melting curves.

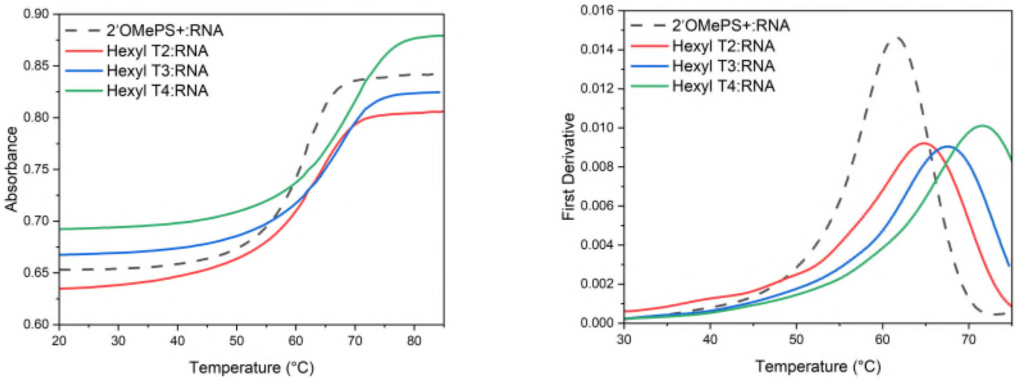

Supplementary Figure S118: UV melting studies of **Hexyl T2, Hexyl T3 and Hexyl T4** against complementary RNA. *Left:* Representative UV melting curves measured using 2 µM of each oligonucleotide in 10 mM Na-phosphate buffer, 25 mM NaCl, pH = 7.0; *Right:* 1st derivative of melting curves.

3.4 Octyl T2, Octyl T3 and Octyl T4

Supplementary Table T7: Duplex melting temperatures (Tm) in °C of **Octyl T2, Octyl T3 and Octyl T4** against complementary DNA and RNA. Nucleotides in black have 2´-OMe ribose sugars and phosphorothioate internucleoside linkages. Nucleotides in red are locked nucleic acid alkyl phosphothiotriesters.

Oligonucleotide	Sequence (5´→3´)	DNA target Tm (ΔTm)	RNA target Tm (ΔTm)
Octyl T2	CCU CUT ACC UCA GUT ACA	51.5 (+3.0)	65.5 (+4.2)
Octyl T3	CCU CUT ACC TCA GUT ACA	51.4 (+2.9)	69.2 (+7.9)
Octyl T4	CCT CUT ACC TCA GUT ACA	50.5 (+2.0)	66.1 (+5.8)

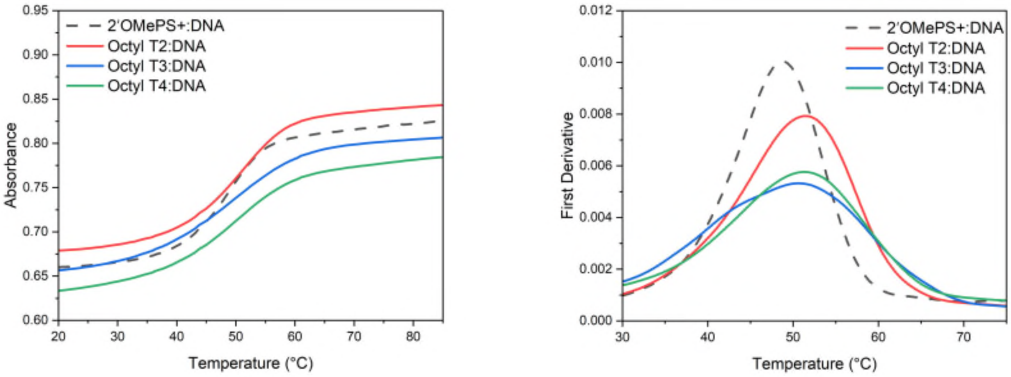

Supplementary Figure S119: UV melting studies of **Octyl T2, Octyl T3 and Octyl T4** against complementary DNA. *Left:* Representative UV melting curves measured using 2 µM of each oligonucleotide in 10 mM Na-phosphate buffer, 100 mM NaCl, pH = 7.0; *Right:* 1st derivative of melting curves.

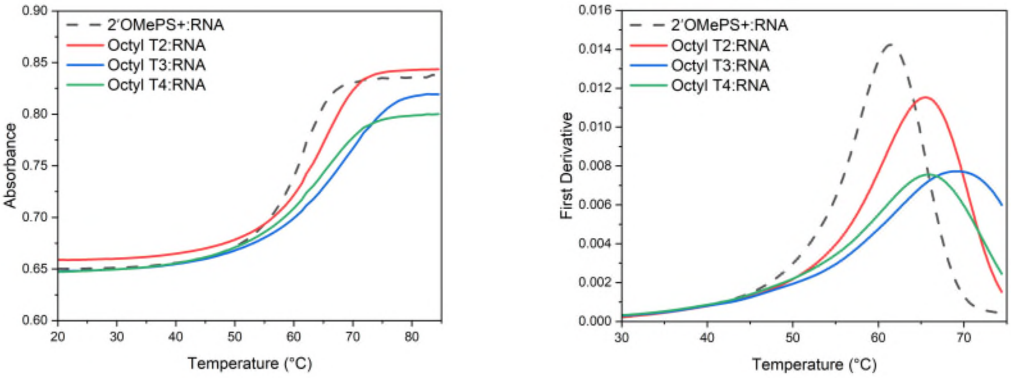

Supplementary Figure S120: UV melting studies of **Octyl T2, Octyl T3 and Octyl T4** against complementary RNA. *Left:* Representative UV melting curves measured using 2 µM of each oligonucleotide in 10 mM Na-phosphate buffer, 25 mM NaCl, pH = 7.0; *Right:* 1st derivative of melting curves.

3.5 Pentyn T1, Pentyn T2 and Pentyn T3

Supplementary Table T8: Duplex melting temperatures (Tm) in °C of **Pentyn T1, Pentyn T2 and Pentyn T3** against complementary DNA and RNA. Nucleotides in black have 2´-OMe ribose sugars and phosphorothioate internucleoside linkages. Nucleotides in red are locked nucleic acid alkyl phosphothiotriesters

Pentyn T1	CCU CUU ACC UCA GUT ACA	51.1 (+2.6)	63.4 (+2.1)
Pentyn T2	CCU CUT ACC UCA GUT ACA	54.3 (+5.8)	65.9 (+4.6)
Pentyn T3	CCU CUT ACC TCA GUT ACA	56.6 (+8.1)	67.6 (+6.3)

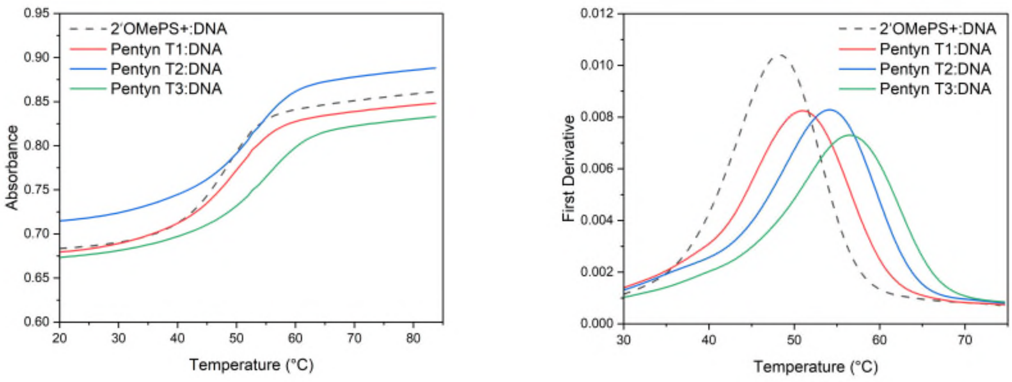

Supplementary Figure S121: UV melting studies of **Pentyn T1, Pentyn T2 and Pentyn T3** against complementary DNA. *Left:* Representative UV melting curves measured using 2 µM of each oligonucleotide in 10 mM Na-phosphate buffer, 100 mM NaCl, pH = 7.0; *Right:* 1st derivative of melting curves.

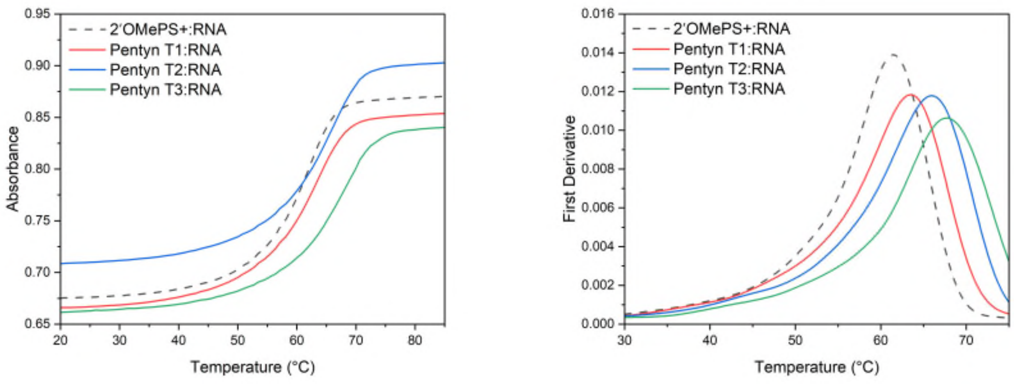

Supplementary Figure S122: UV melting studies of **Pentyn T1, Pentyn T2 and Pentyn T3** against complementary RNA. *Left:* Representative UV melting curves measured using 2 µM of each oligonucleotide in 10 mM Na-phosphate buffer, 25 mM NaCl, pH = 7.0; *Right:* 1st derivative of melting curves.

3.6 Glycol T1, Glycol T2 and Glycol T3

Supplementary Table T9: Duplex melting temperatures (Tm) in °C of **Glycol T1, Glycol T2 and Glycol T3** against complementary DNA and RNA. Nucleotides in black have 2´-OMe ribose sugars and phosphorothioate internucleoside linkages. Nucleotides in red are locked nucleic acid phosphothiotriesters.

Glycol T1	CCU CUU ACC UCA GUT ACA	50.7 (+2.2)	62.9 (+1.6)
Glycol T2	CCU CUT ACC UCA GUT ACA	52.4 (+3.9)	65.3 (4.0)
Glycol T3	CCU CUT ACC TCA GUT ACA	54.3 (+5.8)	66.0 (+4.7)

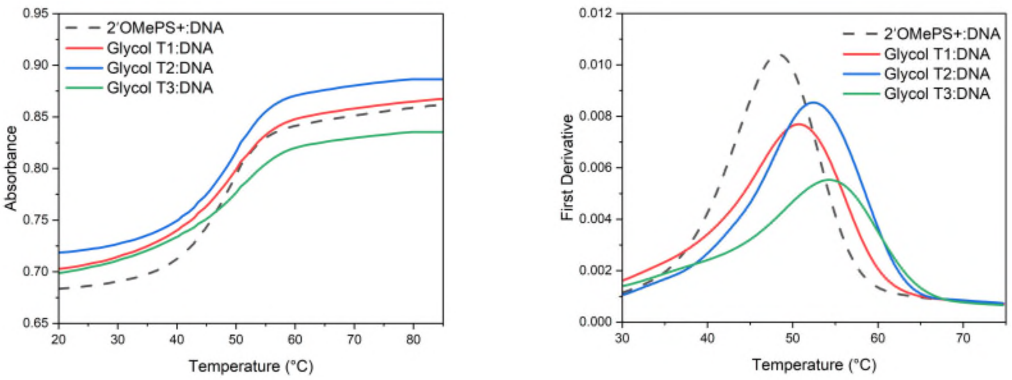

Supplementary Figure S123: UV melting studies of **Glycol T1, Glycol T2 and Glycol T3** against complementary DNA. *Left:* Representative UV melting curves measured using 2 µM of each oligonucleotide in 10 mM Na-phosphate buffer, 100 mM NaCl, pH = 7.0; *Right:* 1st derivative of melting curves.

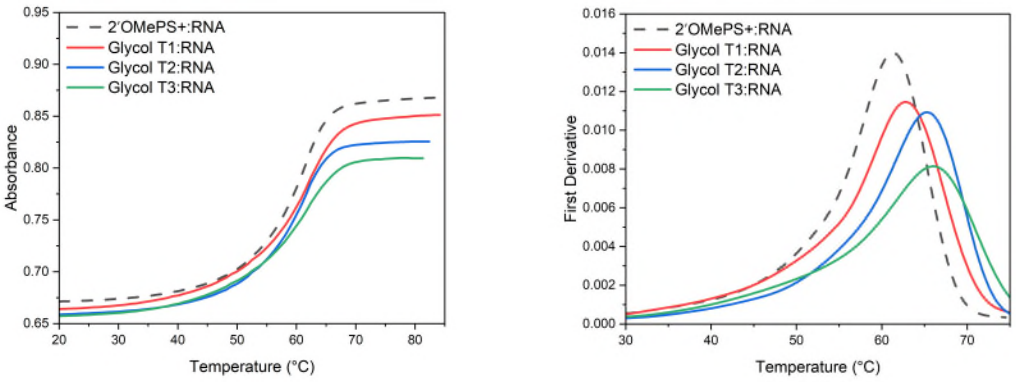

Supplementary Figure S124: UV melting studies of **Glycol T1, Glycol T2 and Glycol T3** against complementary RNA. *Left:* Representative UV melting curves measured using 2 µM of each oligonucleotide in 10 mM Na-phosphate buffer, 25 mM NaCl, pH = 7.0; *Right:* 1st derivative of melting curves.

3.7 Gluc A1 and Gluc A2

Supplementary Table T10: Duplex melting temperatures (Tm) in °C of **Gluc A1 and Gluc A2** against complementary DNA and RNA. Nucleotides in black have 2´-OMe ribose sugars and phosphorothioate internucleoside linkages. Nucleotides in in blue are locked nucleic acid phosphothiotriesters.

Gluc A1	CCU CUU ACC UCA GUU ACA	51.5 (+3.0)	63.5 (+2.2)
Gluc A2	CCU CUU ACC UCA GUU ACA	53.3 (+4.8)	64.5 (+3.2)

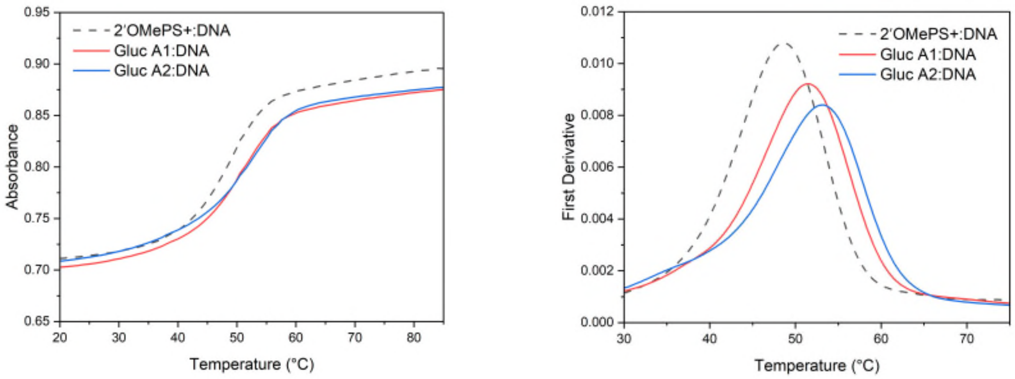

Supplementary Figure S125: UV melting studies of **Gluc A1 and Gluc A2** against complementary DNA. *Left:* Representative UV melting curves measured using 2 µM of each oligonucleotide in 10 mM Na-phosphate buffer, 100 mM NaCl, pH = 7.0; *Right:* 1st derivative of melting curves.

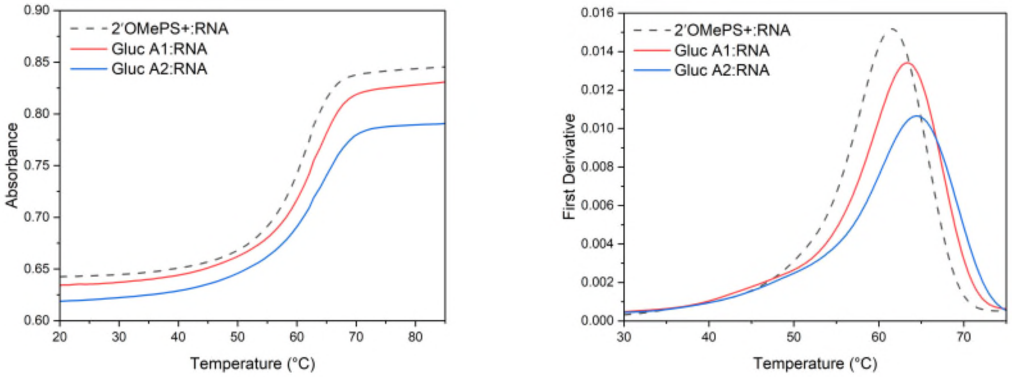

Supplementary Figure S126: UV melting studies of **Gluc A1 and Gluc A2** against complementary RNA (**ON43**). *Left:* Representative UV melting curves measured using 2 µM of each oligonucleotide in 10 mM Na-phosphate buffer, 25 mM NaCl, pH = 7.0; *Right:* 1st derivative of melting curves.

3.8 Gluc T3, Gal T3 and Lac T3

Supplementary Table T11: Duplex melting temperatures (Tm) in °C of **Gluc T3, Gal T3 and Lac T3** against complementary DNA and RNA. Nucleotides in black have 2´-OMe ribose sugars and phosphorothioate internucleoside linkages. Nucleotides in red are locked nucleic acid phosphothiotriesters

Gluc T3	CCU CUT ACC TCA GUT ACA	57.1 (+8.6)	68.6 (+7.3)
Gal T3	CCU CUT ACC TCA GUT ACA	57.5 (+9.0)	68.8 (+7.5)
Lac T3	CCU CUT ACC TCA GUT ACA	57.0 (+8.5)	68.2 (+6.9)

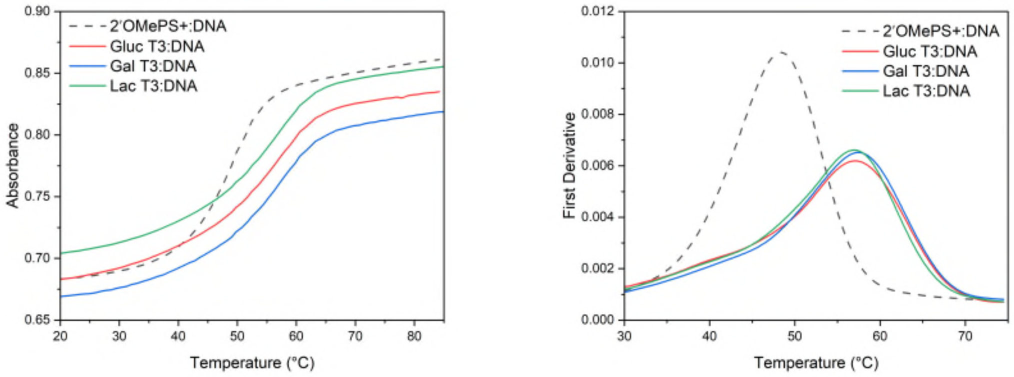

Supplementary Figure S127: UV melting studies of **Gluc T3, Gal T3 and Lac T3** against complementary DNA. *Left:* Representative UV melting curves measured using 2 µM of each oligonucleotide in 10 mM Na-phosphate buffer, 100 mM NaCl, pH = 7.0; *Right:* 1st derivative of melting curves.

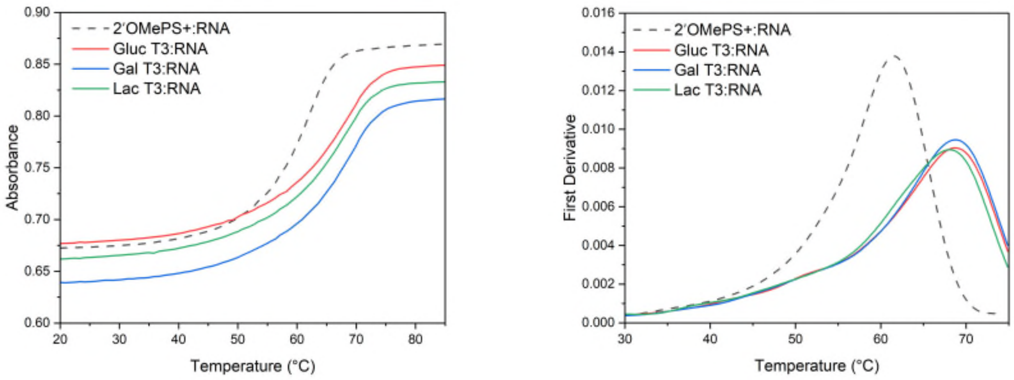

Supplementary Figure S128: UV melting studies of **Gluc T3, Gal T3 and Lac T3** against complementary RNA. *Left:* Representative UV melting curves measured using 2 µM of each oligonucleotide in 10 mM Na-phosphate buffer, 25 mM NaCl, pH = 7.0; *Right:* 1st derivative of melting curves.

3.9 Lys T3, Leu T3, Phe T3 and Val T3

Supplementary Table T12: Duplex melting temperatures (Tm) in °C of **Lys T3, Leu T3, Phe T3 and Val T3** against complementary DNA and RNA. Nucleotides in black have 2´-OMe ribose sugars and phosphorothioate internucleoside linkages. Nucleotides in red are locked nucleic acid phosphothiotriesters

Lys T3	CCU CUT ACC TCA GUT ACA	56.8 (+8.3)	68.5 (+7.2
Leu T3	CCU CUT ACC TCA GUT ACA	54.4 (+5.9)	66.0 (+4.7)
Phe T3	CCU CUT ACC TCA GUT ACA	54.8 (+6.3)	66.9 (+5.6)
Val T3	CCU CUT ACC TCA GUT ACA	54.7 (+6.2)	66.3 (+5.0)

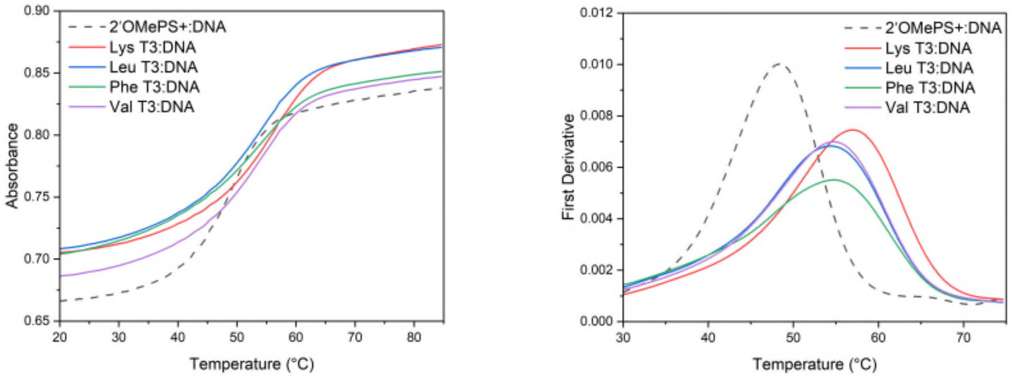

Supplementary Figure S129: UV melting studies of **Lys T3, Leu T3, Phe T3 and Val T3** against complementary DNA (**ON42**). *Left:* Representative UV melting curves measured using 2 µM of each oligonucleotide in 10 mM Na-phosphate buffer, 100 mM NaCl, pH = 7.0; *Right:* 1st derivative of melting curves.

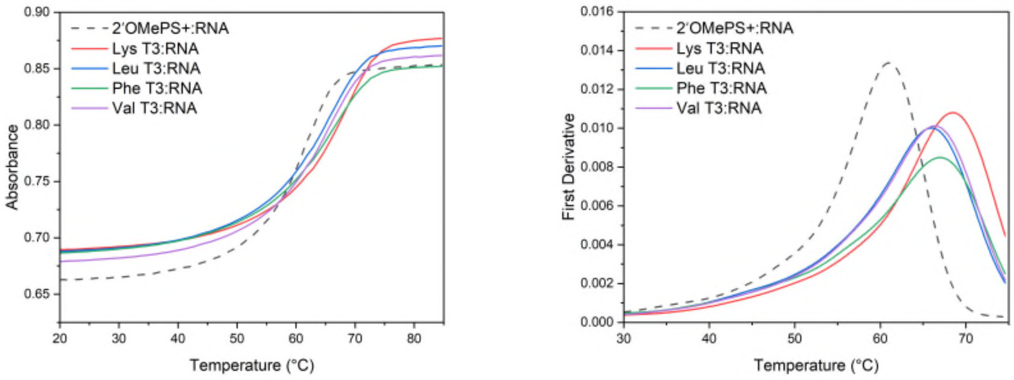

Supplementary Figure S130: UV melting studies of **Lys T3, Leu T3, Phe T3 and Val T3** against complementary RNA (**ON43**). *Left:* Representative UV melting curves measured using 2 µM of each oligonucleotide in 10 mM Na-phosphate buffer, 25 mM NaCl, pH = 7.0; *Right:* 1st derivative of melting curves.

3.10 Sper T1 and Sper T2

Supplementary Table T13: Duplex melting temperatures (Tm) in °C of **Sper T1 and Sper T2** against complementary DNA and RNA. Nucleotides in black have 2´-OMe ribose sugars and phosphorothioate internucleoside linkages. Nucleotides in red are locked nucleic acid phosphothiotriesters

Sper T1	CCU CUU ACC UCA GUT ACA	51.0 (+2.5)	64.5 (+3.2)
Sper T2	CCU CUT ACC UCA GUT ACA	59.0 (+10.5)	74.7 (+13.4)

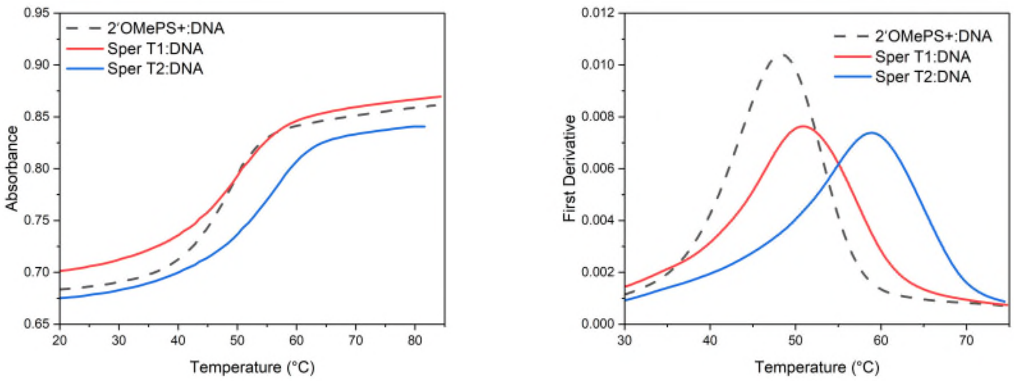

Supplementary Figure S131: UV melting studies of **Sper T1 and Sper T2** against complementary DNA. *Left:* Representative UV melting curves measured using 2 µM of each oligonucleotide in 10 mM Na-phosphate buffer, 100 mM NaCl, pH = 7.0; *Right:* 1st derivative of melting curves.

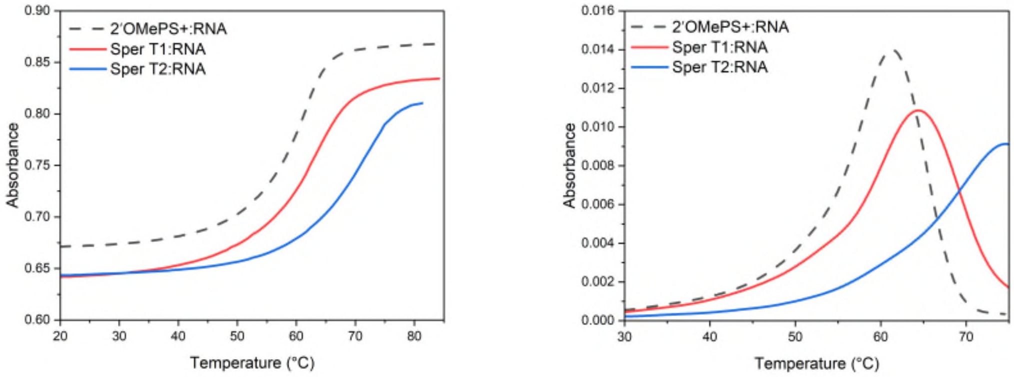

Supplementary Figure S132: UV melting studies of **Sper T1 and Sper T2** against complementary RNA. *Left:* Representative UV melting curves measured using 2 µM of each oligonucleotide in 10 mM Na-phosphate buffer, 25 mM NaCl, pH = 7.0; *Right:* 1st derivative of melting curves.

3.11 TO A1, TO A2, TO T1 and TO T2

Supplementary Table T14: Duplex melting temperatures (Tm) in °C of **TO A1, TO A2, TO T1 and TO T2** against complementary DNA and RNA. Nucleotides in black have 2´-OMe ribose sugars and phosphorothioate internucleoside linkages. Nucleotides in red are LNA T-alkyl phosphothiotriesters, and in blue are LNA A-alkyl phosphothiotriesters .

TO A1	CCU CUU ACC UCA GUU ACA	53.7 (+5.2)	64.8 (+3.5)
TO A2	CCU CUU ACC UCA GUU ACA	52.0 (+3.5)	64.2 (+2.9)
TO T1	CCU CUU ACC UCA GUT ACA	50.0 (+1.5)	63.8 (+2.5)
TO T2	CCU CUT ACC UCA GUT ACA	48.0 (-0.5)	60.7 (-0.6)

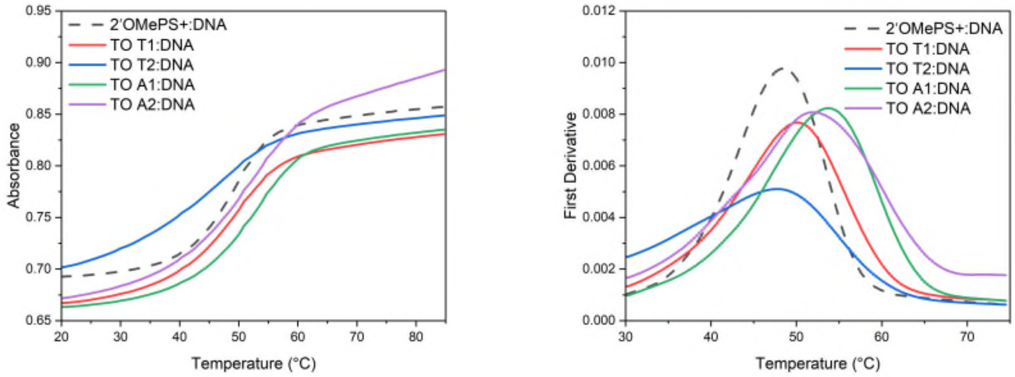

Supplementary Figure S133: UV melting studies of **TO A1, TO A2, TO T1 and TO T2** against complementary DNA. *Left:* Representative UV melting curves measured using 2 µM of each oligonucleotide in 10 mM Na-phosphate buffer, 100 mM NaCl, pH = 7.0; *Right:* 1st derivative of melting curves.

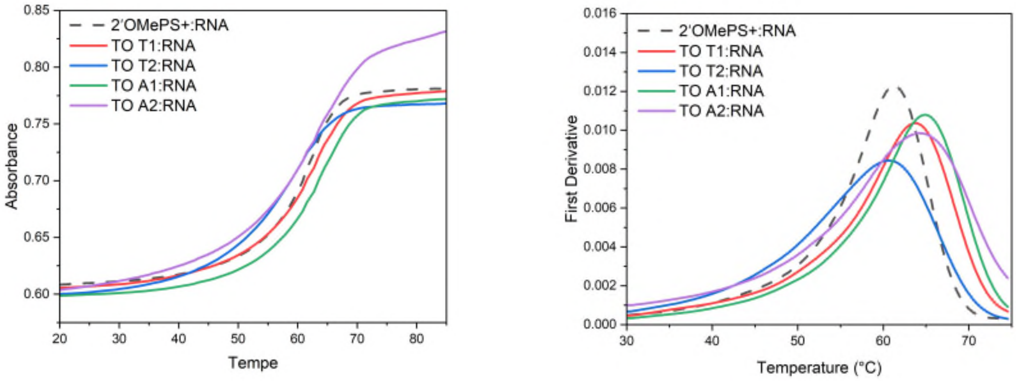

Supplementary Figure S134: UV melting studies for modified ONs **TO A1, TO A2, TO T1 and TO T2** against complementary RNA. *Left:* Representative UV melting curves measured using 2 µM of each oligonucleotide in 10 mM Na-phosphate buffer, 25 mM NaCl, pH = 7.0; *Right:* 1st derivative of melting curves.

4.0 Circular dichroism (CD)

CD spectra were acquired with a Chirascan CD spectrometer (Applied Photophysics Ltd). Duplexes was prepared from 2 nM of modified oligonucleotide with 2 nM of complementary DNA or RNA in 100 mM Na-Phosphate buffer with 100 mM NaCl (for DNA) or 25 mM NaCl (for RNA) at pH 7.0. CD was recorded for duplex sequences were measured in 2 mm quartz cuvettes at 25 °C. Spectra were obtained by the accumulation of four scans at a speed 20nm/min over a range of 200–330 nm, a bandwidth of 1 nm.

4.1 Butyl T2, Butyl T3 and Butyl T4

Supplementary Table 14: Nucleotides in black have 2´-OMe ribose sugars and phosphorothioate internucleoside linkages. Nucleotides in red are locked nucleic acid phosphothiotriesters.

Oligonucleotide	Sequence (5´→3´)	Modification
Butyl T2	CCU CUT ACC UCA GUT ACA	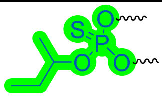
Butyl T3	CCU CUT ACC TCA GUT ACA	
Butyl T4	CCT CUT ACC TCA GUT ACA	

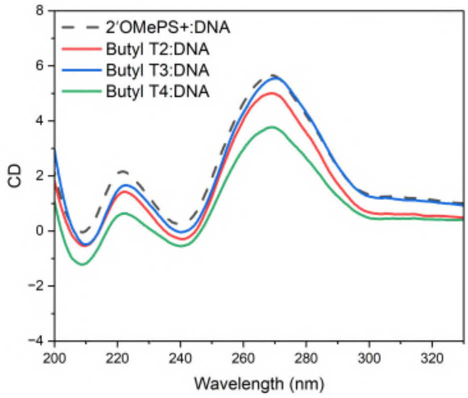

Supplementary Figure S135: CD spectra of modified oligonucleotides (**Butyl T2, Butyl T3 and Butyl T4**) complementary DNA duplex in 10 mM Na-Phosphate buffer at 100 mM NaCl, pH 7.0. Y-axis is ellipticity θ, (10^{-3} deg.cm^2/dmol).

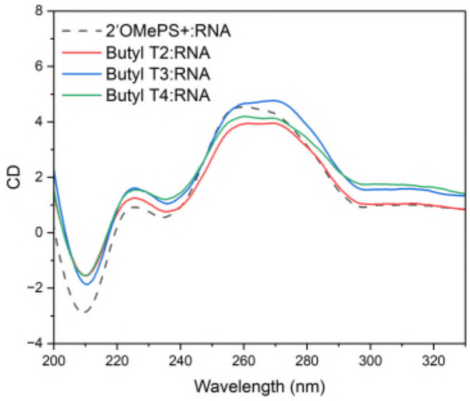

Supplementary Figure S136: CD spectra of modified oligonucleotides (**Butyl T2, Butyl T3 and Butyl T4**) complementary RNA duplex in 10 mM Na-Phosphate buffer at 25 mM NaCl, pH 7.0. Y-axis is ellipticity θ, (10^{-3} deg.cm^2/dmol).

4.2 CF_3 T1, CF_3 T2, CF_3 T3 and CF_3 T4

Supplementary Table 15: Nucleotides in black have 2´-OMe ribose sugars and phosphorothioate internucleoside linkages. Nucleotides in red are locked nucleic acid phosphothiotriesters.

Oligonucleotide	Sequence (5´→3´)	Modification
CF_3 T1	CCU CUU ACC UCA GUT ACA	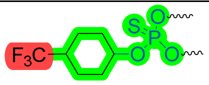
CF_3 T2	CCU CUT ACC UCA GUT ACA	
CF_3 T3	CCU CUT ACC TCA GUT ACA	
CF_3 T4	CCT CUT ACC TCA GUT ACA	

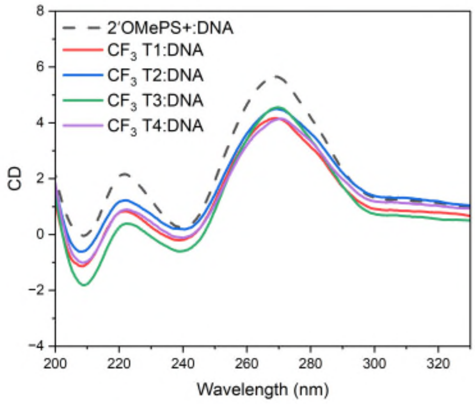

Supplementary Figure S137: CD spectra of modified oligonucleotides (**CF_3 T1, CF_3 T2, CF_3 T3 and CF_3 T4**) complementary DNA duplex in 10 mM Na-Phosphate buffer at 100 mM NaCl, pH 7.0. Y-axis is ellipticity θ, (10^{-3} deg.cm^2/dmol).

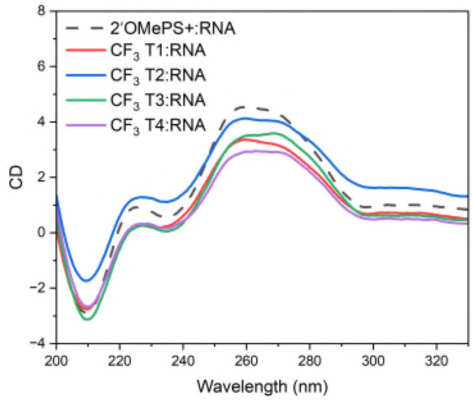

Supplementary Figure S138: CD spectra of modified oligonucleotides (**CF_3 T1, CF_3 T2, CF_3 T3 and CF_3 T4**) complementary RNA duplex in 10 mM Na-Phosphate buffer at 25 mM NaCl, pH 7.0. Y-axis is ellipticity θ, (10^{-3} deg.cm^2/dmol).

4.3 Hexyl T2, Hexyl T3 and Hexyl T4

Supplementary Table 16: Nucleotides in black have 2´-OMe ribose sugars and phosphorothioate internucleoside linkages. Nucleotides in red are locked nucleic acid phosphothiotriesters.

Oligonucleotide	Sequence (5´→3´)	Modification
Hexyl T2	CCU CUT ACC UCA GUT ACA	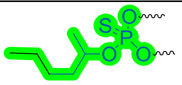
Hexyl T3	CCU CUT ACC TCA GUT ACA	
Hexyl T4	CCT CUT ACC TCA GUT ACA	

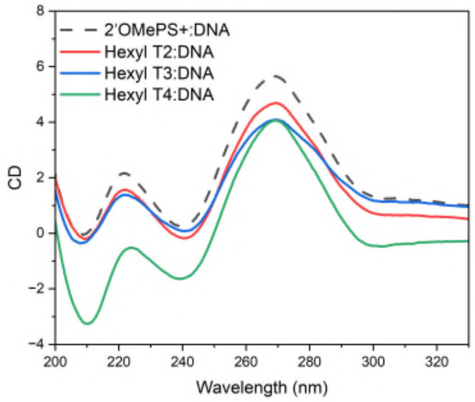

Supplementary Figure S139: CD spectra of oligonucleotide (**Hexyl T2, Hexyl T3 and Hexyl T4**) with complementary DNA duplex at in 10 mM Na-Phosphate buffer at 100 mM NaCl, pH 7.0. Y-axis is ellipticity θ, (10^{-3} deg.cm^2/dmol).

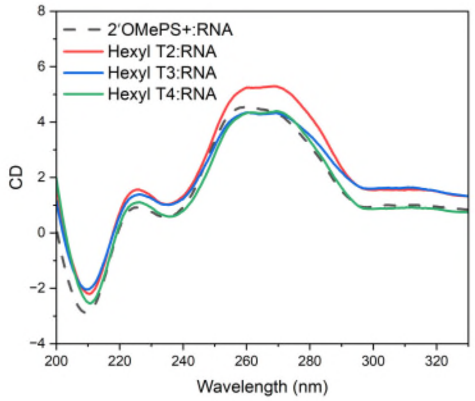

Supplementary Figure S140: CD spectra of oligonucleotides (**Hexyl T2, Hexyl T3 and Hexyl T4)** with complementary RNA duplex at in 10 mM Na-Phosphate buffer at 25 mM NaCl, pH 7.0. Y-axis is ellipticity θ, (10^{-3} deg.cm^2/dmol).

4.4 Octyl T2, Octyl T3 and Octyl T4

Supplementary Table 17: Nucleotides in black have 2´-OMe ribose sugars and phosphorothioate internucleoside linkages. Nucleotides in red are locked nucleic acid phosphothiotriesters.

Oligonucleotide	Sequence (5´→3´)	Modification
Octyl T2	CCU CUT ACC UCA GUT ACA	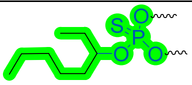
Octyl T3	CCU CUT ACC TCA GUT ACA	
Octyl T4	CCT CUT ACC TCA GUT ACA	

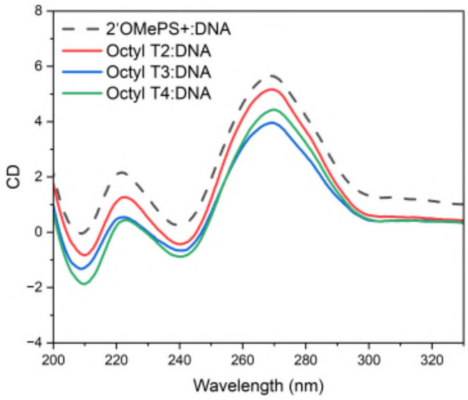

Supplementary Figure S141: CD spectra of oligonucleotides (**Octyl T2, Octyl T3 and Octyl T4**) with complementary DNA duplex at in 10 mM Na-Phosphate buffer at 100 mM NaCl, pH 7.0. Y-axis is ellipticity θ, (10^{-3} deg.cm^2/dmol).

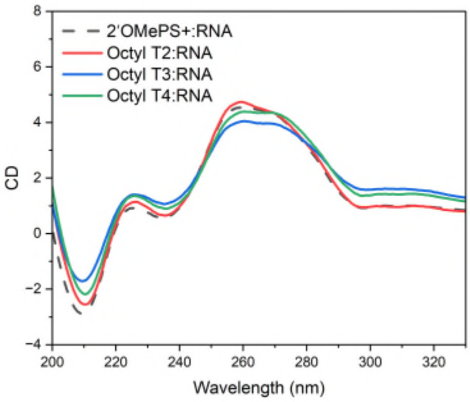

Supplementary Figure S142: CD spectra of oligonucleotides (**Octyl T2, Octyl T3 and Octyl T4**) with complementary RNA duplex at in 10 mM Na-Phosphate buffer at 25 mM NaCl, pH 7.0. Y-axis is ellipticity θ, (10^{-3} deg.cm^2/dmol).

4.5 Pentyn T1, Pentyn T2 and Pentyn T3

Supplementary Table 18: Nucleotides in black have 2´-OMe ribose sugars and phosphorothioate internucleoside linkages. Nucleotides in red are locked nucleic acid phosphothiotriesters.

Oligonucleotide	Sequence (5´→3´)	Modification
Pentyn T1	CCU CUU ACC UCA GUT ACA	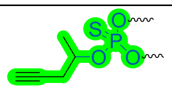
Pentyn T2	CCU CUT ACC UCA GUT ACA	
Pentyn T3	CCU CUT ACC TCA GUT ACA	

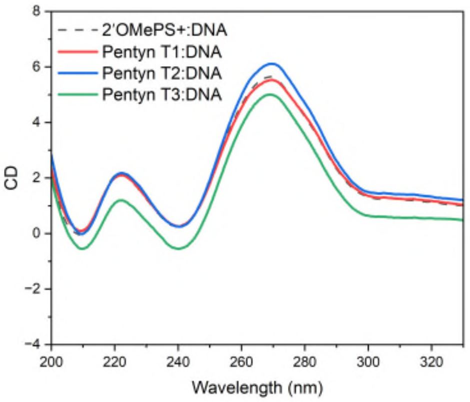

Supplementary Figure S143: CD spectra of modified oligonucleotides (**Pentyn T1-T3**) complementary DNA duplex in 10 mM Na-Phosphate buffer at 100 mM NaCl, pH 7.0. Y-axis is ellipticity θ, (10^{-3} deg.cm^2/dmol).

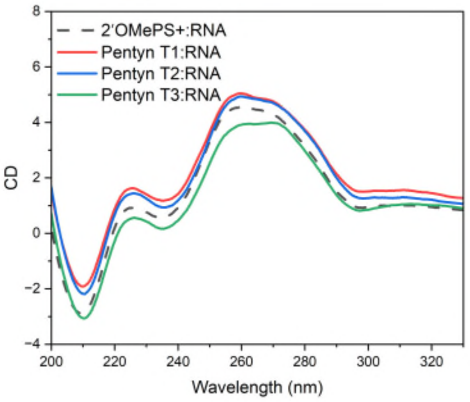

Supplementary Figure S144: CD spectra of modified oligonucleotides (**Pentyn T1-T3**) complementary RNA duplex in 10 mM Na-Phosphate buffer at 25 mM NaCl, pH 7.0. Y-axis is ellipticity θ, (10^{-3} deg.cm^2/dmol).

4.6 Glycol T1, Glycol T2 and Glycol T3

Supplementary Table 19: Nucleotides in black have 2´-OMe ribose sugars and phosphorothioate internucleoside linkages. Nucleotides in red are locked nucleic acid phosphothiotriesters.

Oligonucleotide	Sequence (5´→3´)	Modification
Glycol T1	CCU CUU ACC UCA GUT ACA	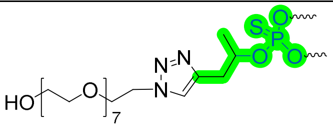
Glycol T2	CCU CUT ACC UCA GUT ACA	
Glycol T3	CCU CUT ACC TCA GUT ACA	

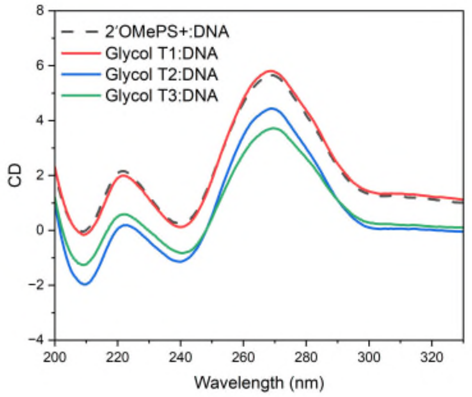

Supplementary Figure S145: CD spectra of oligonucleotides (**Glycol T1, Glycol T2 and Glycol T3**) complementary DNA duplex at in 10 mM Na-Phosphate buffer at 100 mM NaCl, pH 7.0. Y-axis is ellipticity θ, (10^{-3} deg.cm^2/dmol).

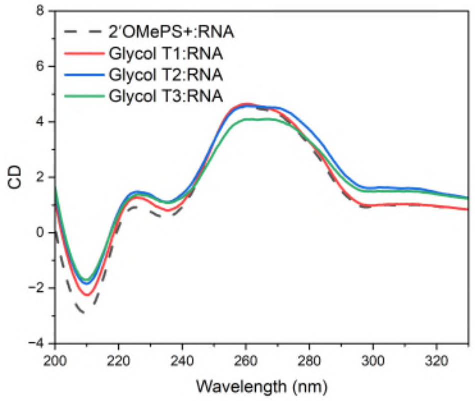

Supplementary Figure S146: CD spectra of oligonucleotides (**Glycol T1, Glycol T2 and Glycol T3**) complementary RNA duplex at in 10 mM Na-Phosphate buffer at 25 mM NaCl, pH 7.0. Y-axis is ellipticity θ, (10^{-3} deg.cm^2/dmol).

4.7 Gluc A1 and Gluc A2

Supplementary Table 20: Nucleotides in black have 2´-OMe ribose sugars and phosphorothioate internucleoside linkages. Nucleotides in in blue are locked nucleic acid phosphothiotriesters.

Oligonucleotide	Sequence (5´→3´)	Modification
Gluc A1	CCU CUU ACC UCA GUU ACA	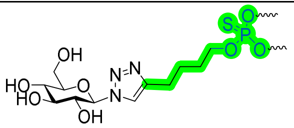
Gluc A2	CCU CUU ACC UCA GUU ACA	

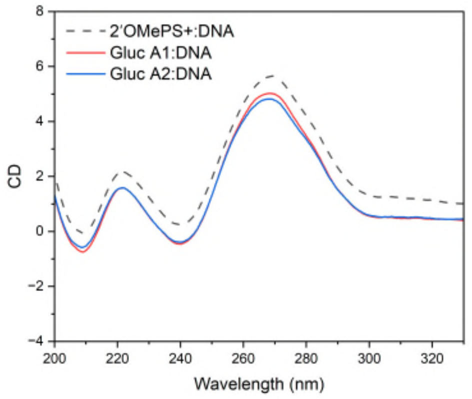

Supplementary Figure S147: CD spectra of oligonucleotides (**Gluc A1 and Gluc A2**) complementary DNA duplex at in 10 mM Na-Phosphate buffer at 100 mM NaCl, pH 7.0. Y-axis is ellipticity θ, (10^{-3} deg.cm^2/dmol).

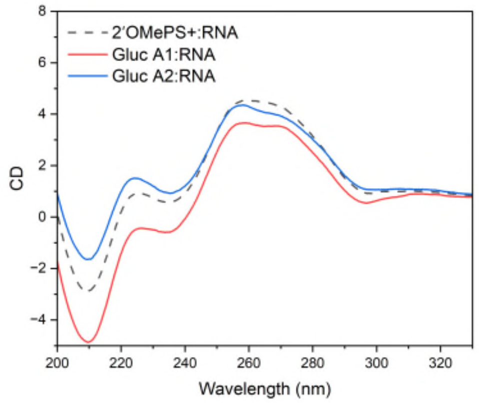

Supplementary Figure S148: CD spectra of oligonucleotide (**Gluc A1 and Gluc A2**) complementary RNA duplex at in 10 mM Na-Phosphate buffer at 25 mM NaCl, pH 7.0. Y-axis is ellipticity θ, (10^{-3} deg.cm^2/dmol).

4.8 Gluc T3, Gal T3 and Lac T3

Supplementary Table 21: Nucleotides in black have 2´-OMe ribose sugars and phosphorothioate internucleoside linkages. Nucleotides in red are locked nucleic acid phosphothiotriesters.

Oligonucleotide	Sequence (5´→3´)	Modification
Gluc T3	CCU CUT ACC TCA GUT ACA	Glucose conjugate
Gal T3	CCU CUT ACC TCA GUT ACA	Galactose conjugate
Lac T3	CCU CUT ACC TCA GUT ACA	Lactose conjugate

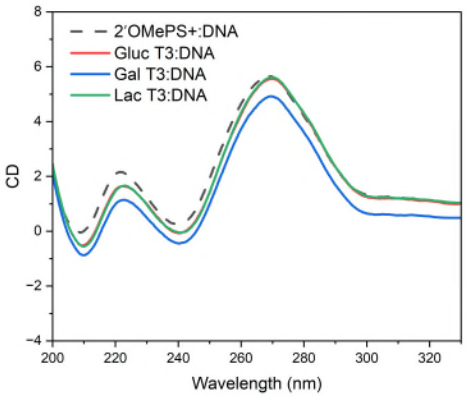

Supplementary Figure S149: CD spectra of oligonucleotides (**Gluc T3, Gal T3 and Lac T3**) complementary DNA duplex at in 10 mM Na-Phosphate buffer at 100 mM NaCl, pH 7.0. Y-axis is ellipticity θ, (10^{-3} deg.cm^2/dmol).

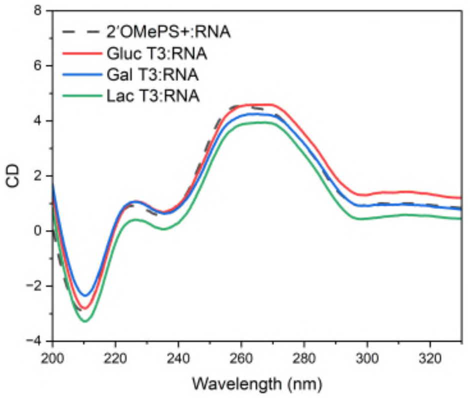

Supplementary Figure S150: CD spectra of oligonucleotide (**Gluc T3, Gal T3 and Lac T3**) complementary RNA (duplex at in 10 mM Na-Phosphate buffer at 25 mM NaCl, pH 7.0. Y-axis is ellipticity θ, (10^{-3} deg.cm^2/dmol).

4.9 Lys T3, Leu T3, Phe T3 and Val T3

Supplementary Table 22: Nucleotides in black have 2´-OMe ribose sugars and phosphorothioate internucleoside linkages. Nucleotides in red are locked nucleic acid phosphothiotriesters

Oligonucleotide	Sequence (5´→3´)	Modification
Lys T3	CCU CUT ACC TCA GUT ACA	Lysine conjugate
Leu T3	CCU CUT ACC TCA GUT ACA	Leucine conjugate
Phe T3	CCU CUT ACC TCA GUT ACA	Phenylalanine conjugate
Val T3	CCU CUT ACC TCA GUT ACA	Valine conjugate

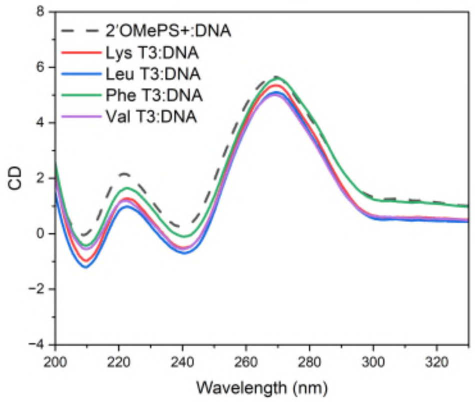

Supplementary Figure S151: CD spectra of oligonucleotides (**Lys T3, Leu T3, Phe T3 and Val T3**) complementary DNA duplex at in 10 mM Na-Phosphate buffer at 100 mM NaCl, pH 7.0. Y-axis is ellipticity θ, (10^{-3} deg.cm^2/dmol).

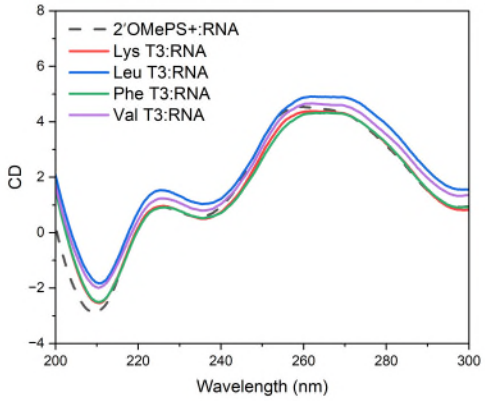

Supplementary Figure S152: CD spectra of oligonucleotides (**Lys T3, Leu T3, Phe T3 and Val T3**) complementary RNA duplex at in 10 mM Na-Phosphate buffer at 25 mM NaCl, pH 7.0. Y-axis is ellipticity θ, (10^{-3} deg.cm^2/dmol).

4.10 Sper T1 and Sper T2

Supplementary Table 23: Nucleotides in black have 2´-OMe ribose sugars and phosphorothioate internucleoside linkages. Nucleotides in red are locked nucleic acid phosphothiotriesters.

Oligonucleotide	Sequence (5´→3´)	Modification
Sper T1	CCU CUU ACC UCA GUT ACA	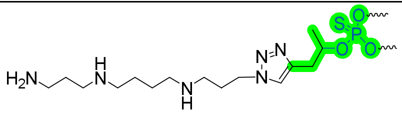
Sper T2	CCU CUT ACC UCA GUT ACA	

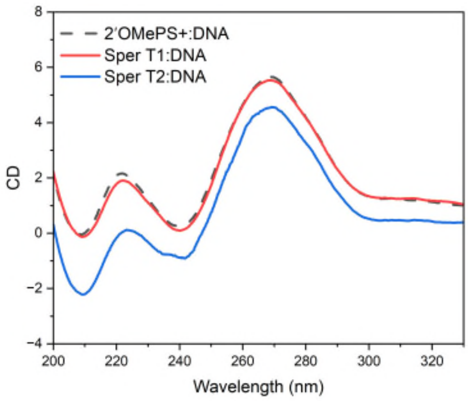

Supplementary Figure S153: CD spectra of oligonucleotides (**Sper T1-T2**) complementary DNA duplex at in 10 mM Na-Phosphate buffer at 100 mM NaCl, pH 7.0. Y-axis is ellipticity θ, (10^{-3} deg.cm^2/dmol).

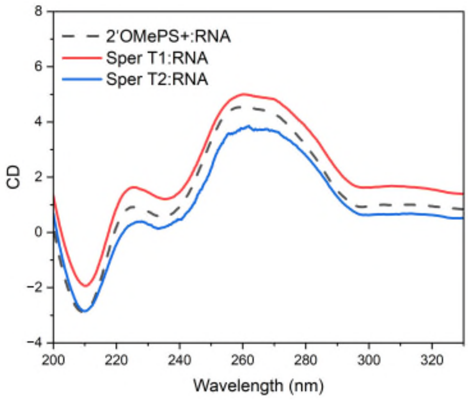

Supplementary Figure S154: CD spectra of oligonucleotides (**Sper T1-T2**) complementary RNA duplex at in 10 mM Na-Phosphate buffer at 25 mM NaCl, pH 7.0. Y-axis is ellipticity θ, (10^{-3} deg.cm^2/dmol).

4.11 TO A1, TO A2, TO T1 and TO T2

Supplementary Table 24: Nucleotides in black have 2´-OMe ribose sugars and phosphorothioate internucleoside linkages. Nucleotides in red are LNA T-alkyl phosphothiotriesters, and in blue are LNA A-alkyl phosphothiotriesters.

Oligonucleotide	Sequence (5´→3´)	Modification
TO T1	CCU CUU ACC UCA GUT ACA	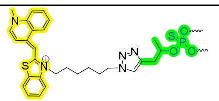
TO T2	CCU CUT ACC UCA GUT ACA	
TO A1	CCU CUU ACC UCA GUU ACA	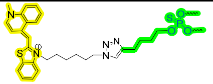
TO A2	CCU CUU ACC UCA GUU ACA	

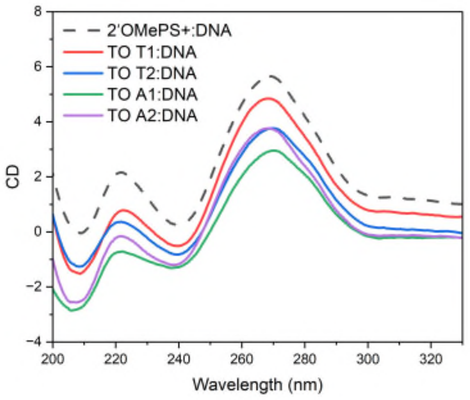

Supplementary Figure S155: CD spectra of modified oligonucleotides (**TO T1-T2 and TO A1-A2**) complementary DNA duplex in 10 mM Na-Phosphate buffer at 100 mM NaCl, pH 7.0. Y-axis is ellipticity θ, (10^{-3} deg.cm^2/dmol).

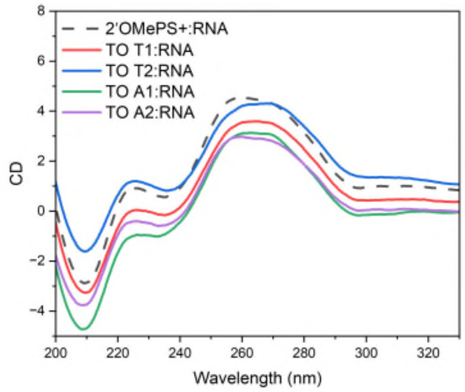

Supplementary Figure S156: CD spectra of modified oligonucleotides (**TO T1-T2 and TO A1-A2**) complementary RNA duplex in 10 mM Na-Phosphate buffer at 25 mM NaCl, pH 7.0. Y-axis is ellipticity θ, (10^{-3} deg.cm^2/dmol).

5.0 Fluorescence measurements

Fluorescence studies were performed on a Perkin Elmer LS50B luminescence spectrometer fitted with Perkin Elmer PTP-1 Peltier temperature controller. FLWinlabTempScan software was used with settings of 400 nm/s scan speed. The emission wavelength was recorded from 510 nm to 700 nm, excitation wavelength 484 nm, gain: high (900V), excitation slit width 7.0 nm, emission slit width 7.0 nm.

Experiment: Samples were prepared in 250 µL cuvettes with 0.25 µM probe containing TO, 100 mM NaCl, 10 mM Na-phosphate buffer pH = 7.0 at 20 °C. Spectra were recorded for the single stranded oligonucleotide probe, and then titrated with 1.1 eq of the desired complementary DNA or RNA and recorded again. Each probe target pair was recorded at least in triplicate.

6.0 Biological Assay

6.1 Experiments, materials and methods

Cell culture

HeLa pLuc/705 cells(2) were maintained in Dulbecco's Modified Eagle Medium (DMEM; Gibco, 31966) supplemented with 10% fetal bovine serum (FBS) and 1X Antibiotic-Antimycotic (Gibco, 15240) at 37 °C and 5% CO_2 in a humidified incubator.

Lipofection experiments

HeLa pLuc/705 cells were seeded at a density of 10,000 cells/well in 100 µL DMEM supplemented with 10% FBS and 1X Antibiotic-Antimycotic in a 96-well plate (Greiner, 655098) and incubated for 16 h at 37 °C and 5% CO_2 in a humidified incubator. Oligonucleotides in aqueous solution were diluted to 100 nM in 300 µL Opti-MEM (Gibco, 31985). Separately, 2.25 µL of Lipofectamine 2000 Transfection Reagent (Invitrogen, 11668) were diluted to a final volume of 300 µL in Opti-MEM and incubated for 5 min at room temperature. After 5 min, the diluted oligonucleotide and diluted transfection reagent were mixed and incubated for 20 min at room temperature to allow complex formation. After 20 min, the complexes were serially diluted in Opti-MEM to achieve final oligonucleotide concentrations of 50, 12.5, and 3.125 nM. At 16 h post-seeding, the complete media was aspirated from the cells and replaced with the diluted complexes or Opti-MEM alone (i.e., the 0 nM treatment or untreated cells). The cells were incubated for 4 h at 37 °C and 5% CO_2 in a humidified incubator. At 4 h post-transfection, the complexes were aspirated from the cells and replaced with complete media, and the cells were incubated for a further 44 h at 37 °C and 5% CO_2 in a humidified incubator.

Gymnosis experiments

Oligonucleotides in aqueous solution were diluted to 40 µM in 400 µL Opti-MEM and then serially diluted in Opti-MEM to achieve oligonucleotide concentrations of 40, 20, and 10 µM. Then, 50 µL of diluted oligonucleotide or Opti-MEM alone (i.e., the 0 µM treatment or untreated cells) and 50 µL HeLa pLuc/705 cells in DMEM supplemented with 6% FBS and 2X Antibiotic-Antimycotic were added to each well of a 96-well plate (Greiner, 655098) to achieve final oligonucleotide concentrations of 20, 10, 5, and 0 µM and a seeding density of 10,000 cells/well in 100 µL DMEM supplemented with 3% FBS and 1X Antibiotic-Antimycotic. The cells were incubated for 72 h at 37 °C and 5% CO_2 in a humidified incubator.

Micrographs

A representative well of treated cells was imaged at the assay end point indicated above for each biological replicate. Representative micrographs of cells treated at the highest dose of ON under both lipofection conditions (i.e., 50 nM) and gymnosis conditions (i.e., 20 µM) are shown below (**Supplementary Figures S157** and **S158**).

Luciferase assays

At the assay end point indicated above, the complete media was aspirated from the cells, and the cells were washed with phosphate buffered saline (PBS; Gibco, 10010) or PBS followed

by 0.1 mg/mL heparin (Merck, H3149) in PBS followed by PBS, respectively. Lysis was performed in 100 µL of Glo Lysis Buffer (Promega, E2661) for 10 min at room temperature with shaking. Then, 50 µL of lysate and 50 µL of Bright-Glo Reagent (Promega, E2620) were added to each well of a 96-well white plate (Greiner, 655075), and after 2 min, luminescence was measured using a CLARIOstar microplate reader (BMG Labtech, software version 5.21.R2). Total protein quantification was carried out using a detergent compatible (DC) Protein Assay (Bio-Rad, 5000111) according to the manufacturer's instructions. Briefly, a bovine serum albumin (BSA) standard (Thermo Scientific, 23209) was prepared at a concentration range of 20-20,000 µg/mL. Then, 5 µL of lysate or BSA standard were treated with 15 µL of Reagent A' and 120 µL of Reagent B and incubated for 20 min at room temperature. Absorbance at 750 nm was measured using a CLARIOstar microplate reader (BMG Labtech, software version 5.21.R2). Total protein quantities were calculated from the measured absorbances using the equation of the linear-fit standard curve in Microsoft Excel.

Both the lipofection and gymnosis experiments described above were performed in biological replicates, where each biological replicate was performed in technical triplicate. To calculate the fold increase over untreated values shown in the plots, the biological replicates were averaged. Data in the plots are means ± SEM for three or more biological replicates ($n>3$), except for MOP T6 under gymnosis conditions, where two biological replicates ($n=2$) are plotted. Statistical analyses were performed in GraphPad Prism 10 for macOS Version 10.4.2 (534).

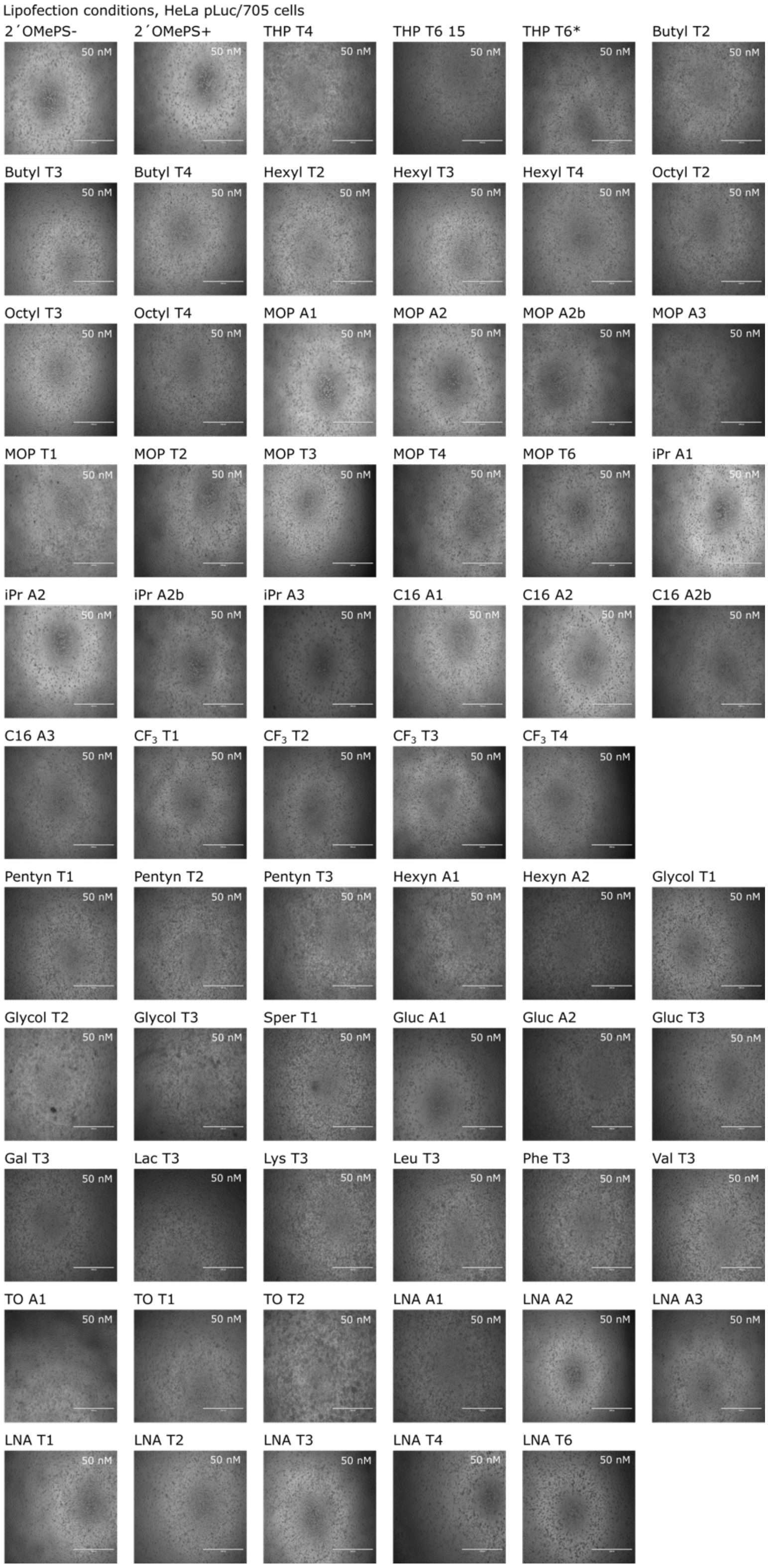

Supplementary Figure S157. Representative micrographs of HeLa pLuc/705 cells treated with ONs under lipofection conditions. ONs were transfected into the cells at 50 nM using Lipofectamine 2000, and images were captured at the assay endpoint 48 h later. Scale bar = 1000 μm.

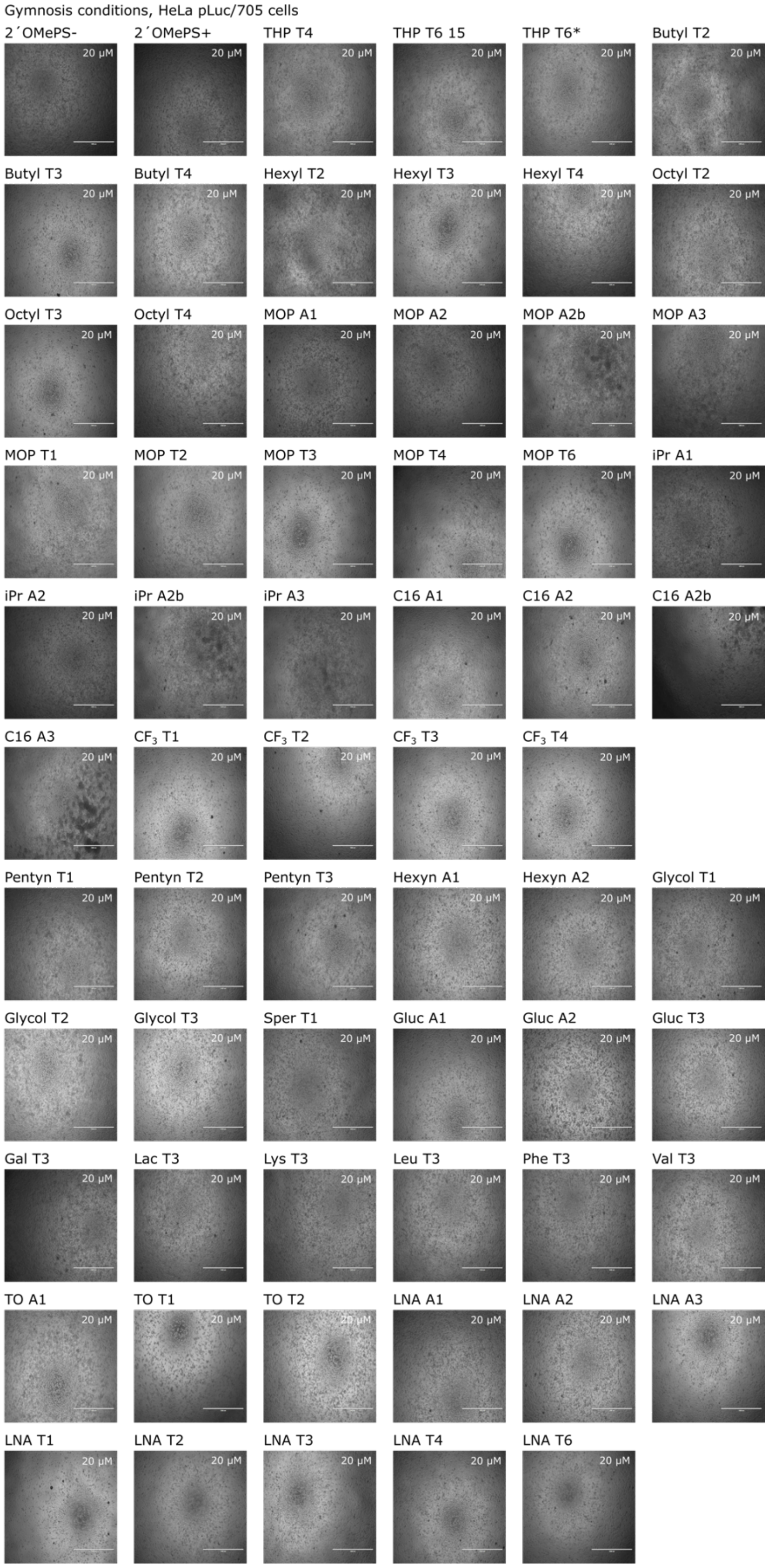

Supplementary Figure S158. Representative micrographs of HeLa pLuc/705 cells treated with ONs under gymnosis conditions. ONs were applied to the cells at 20 µM in the absence of a transfection reagent, and images were captured at the assay endpoint 72 h later. Scale bar = 1000 µm.

7.0 NMR spectra of compounds 7-11 and 13-17

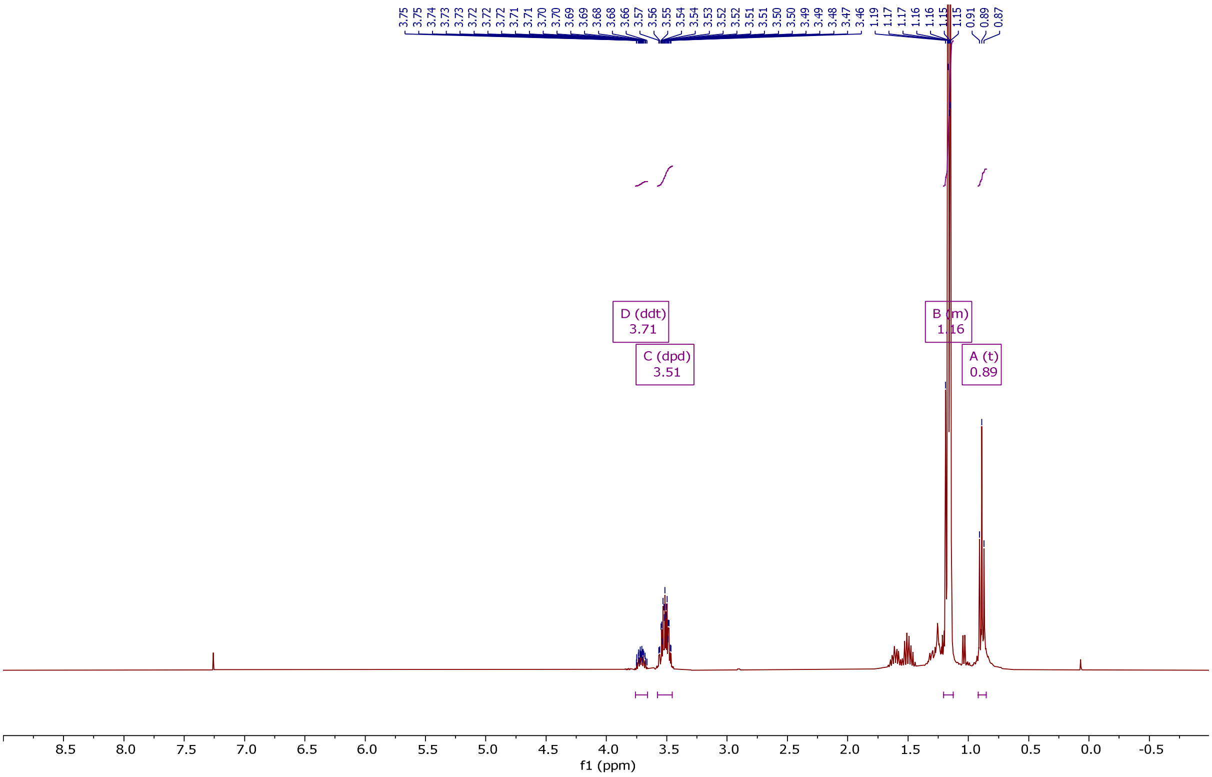

Supplementary Figure S159: ^{1}H NMR (400 MHz, $CDCl_3$) spectrum of **crude 7.**

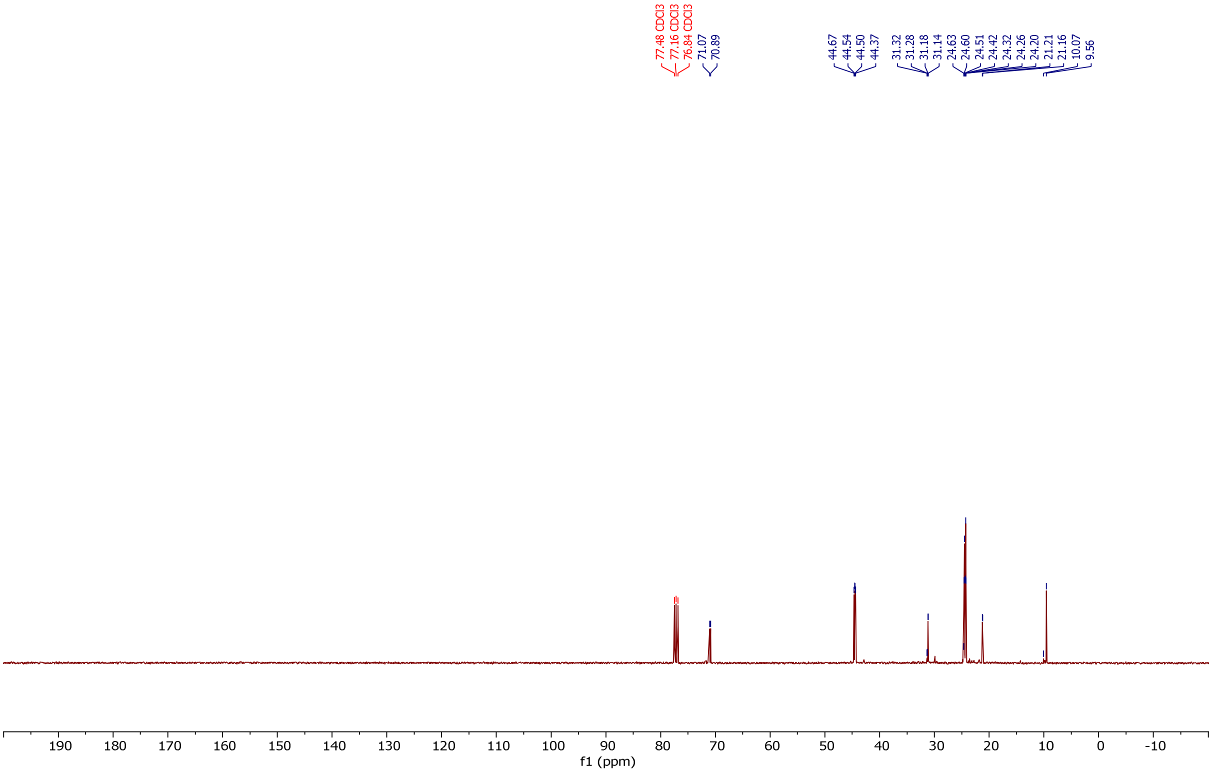

Supplementary Figure S160: ^{13}C NMR (101 MHz, $CDCl_3$) spectrum of **crude 7.**

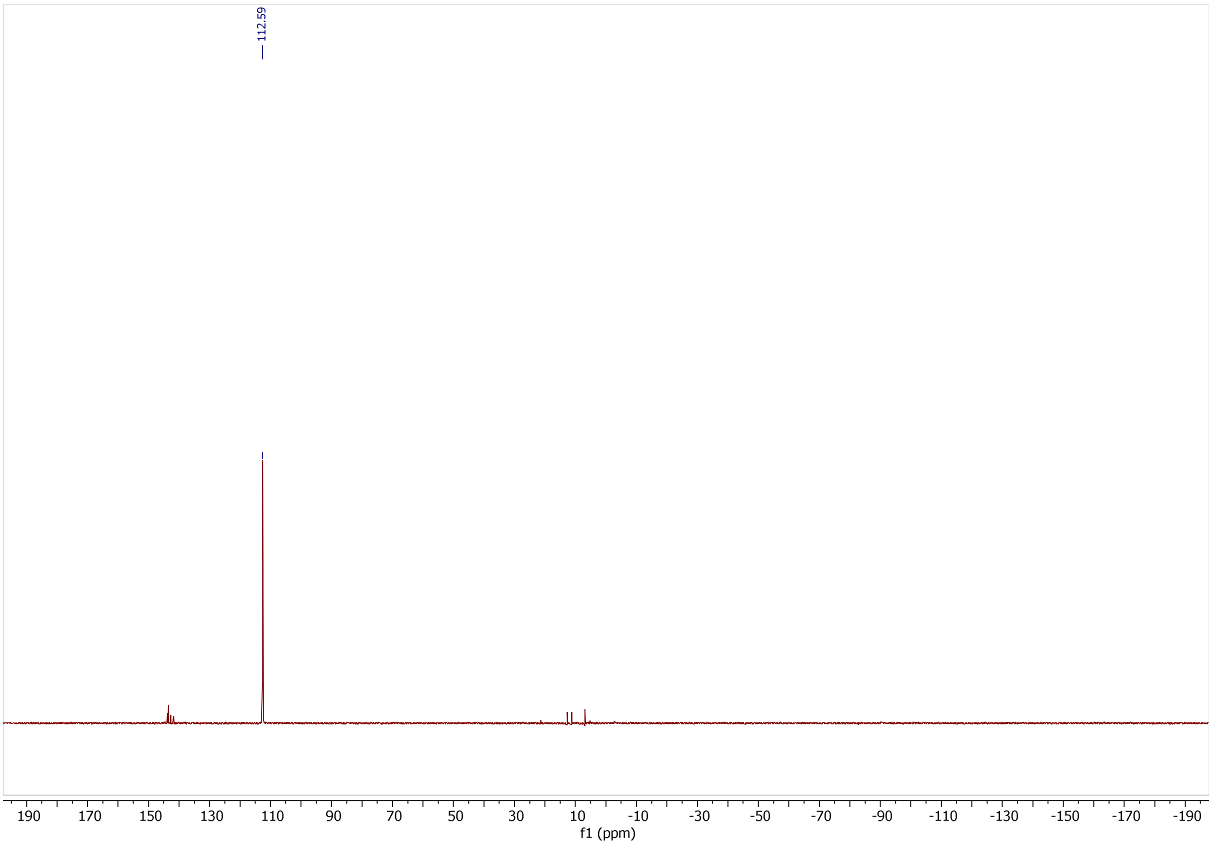

Supplementary Figure S161: ^{31}P NMR (162 MHz, $CDCl_3$) spectrum of **crude 7.**

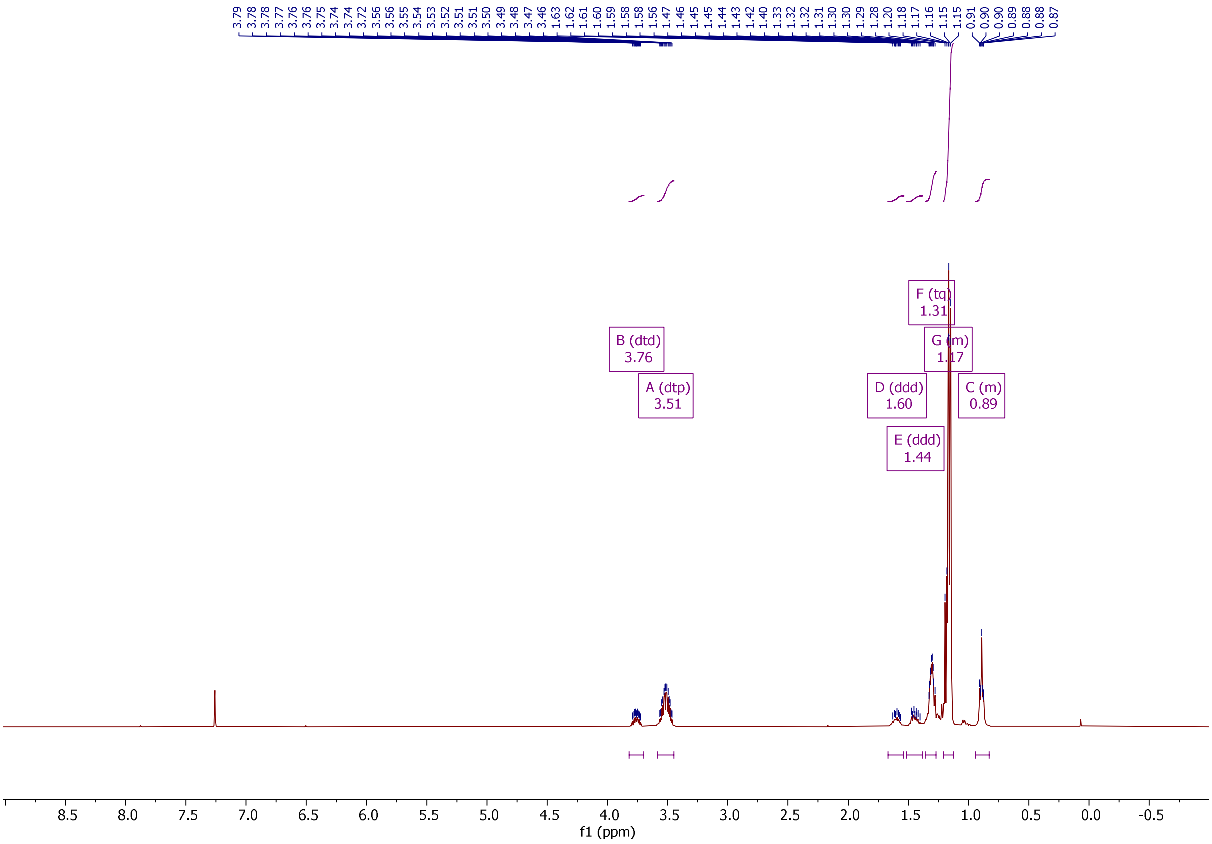

Supplementary Figure S162: ^{1}H NMR (400 MHz, $CDCl_3$) spectrum of **crude 8.**

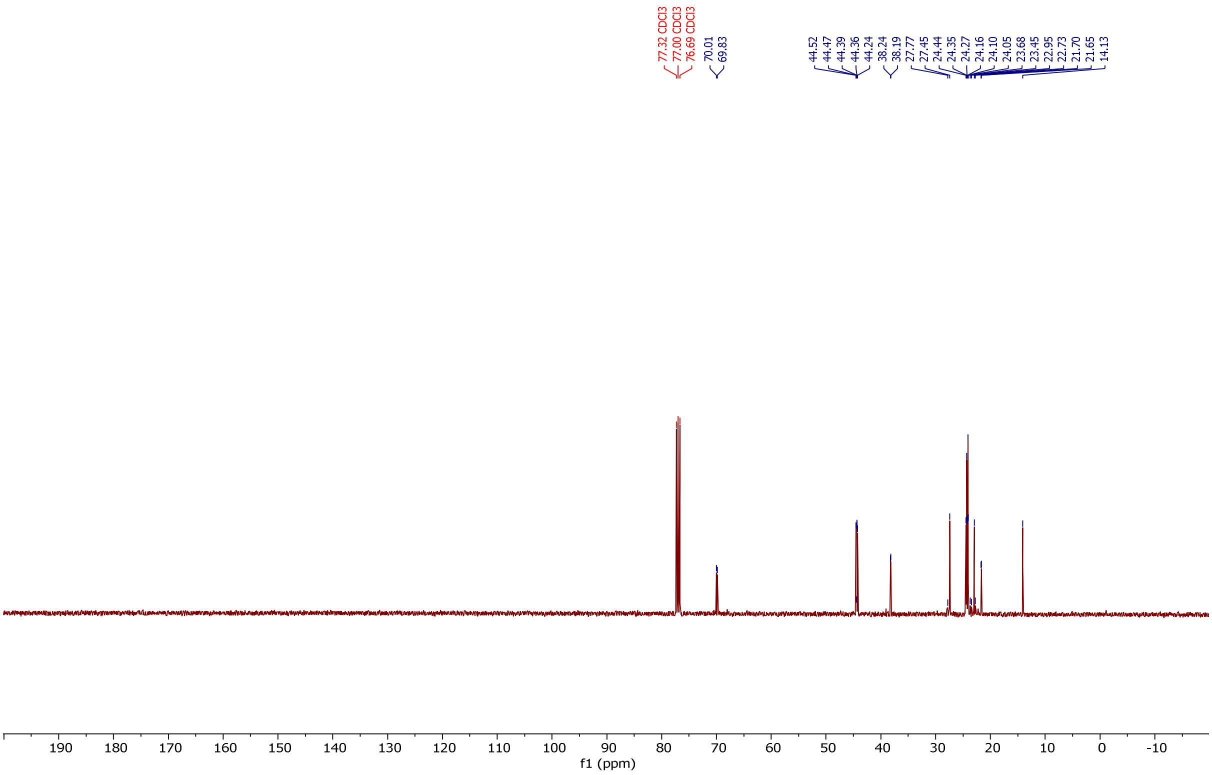

Supplementary Figure S163: ^{13}C NMR (101 MHz, $CDCl_3$) spectrum of **crude 8.**

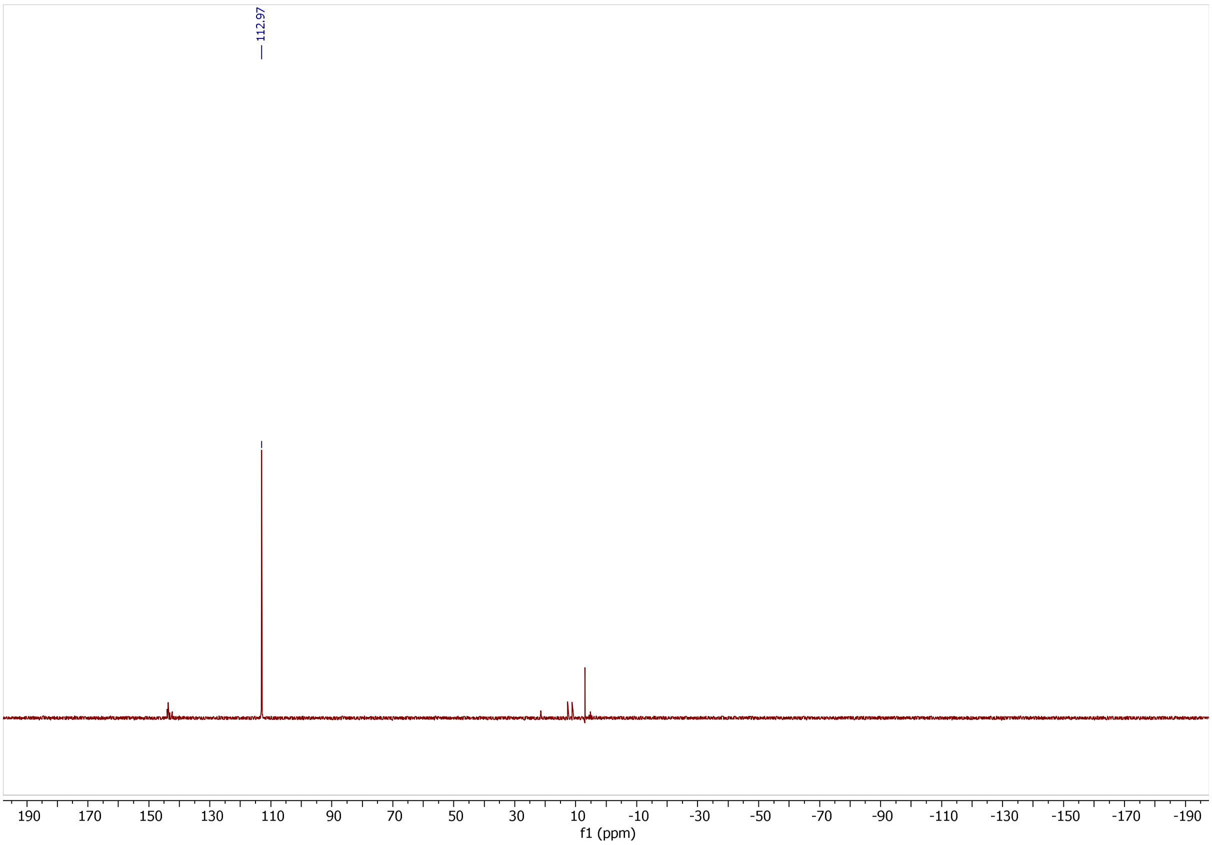

Supplementary Figure S164: ^{31}P NMR (162 MHz, $CDCl_3$) spectrum of **crude 8.**

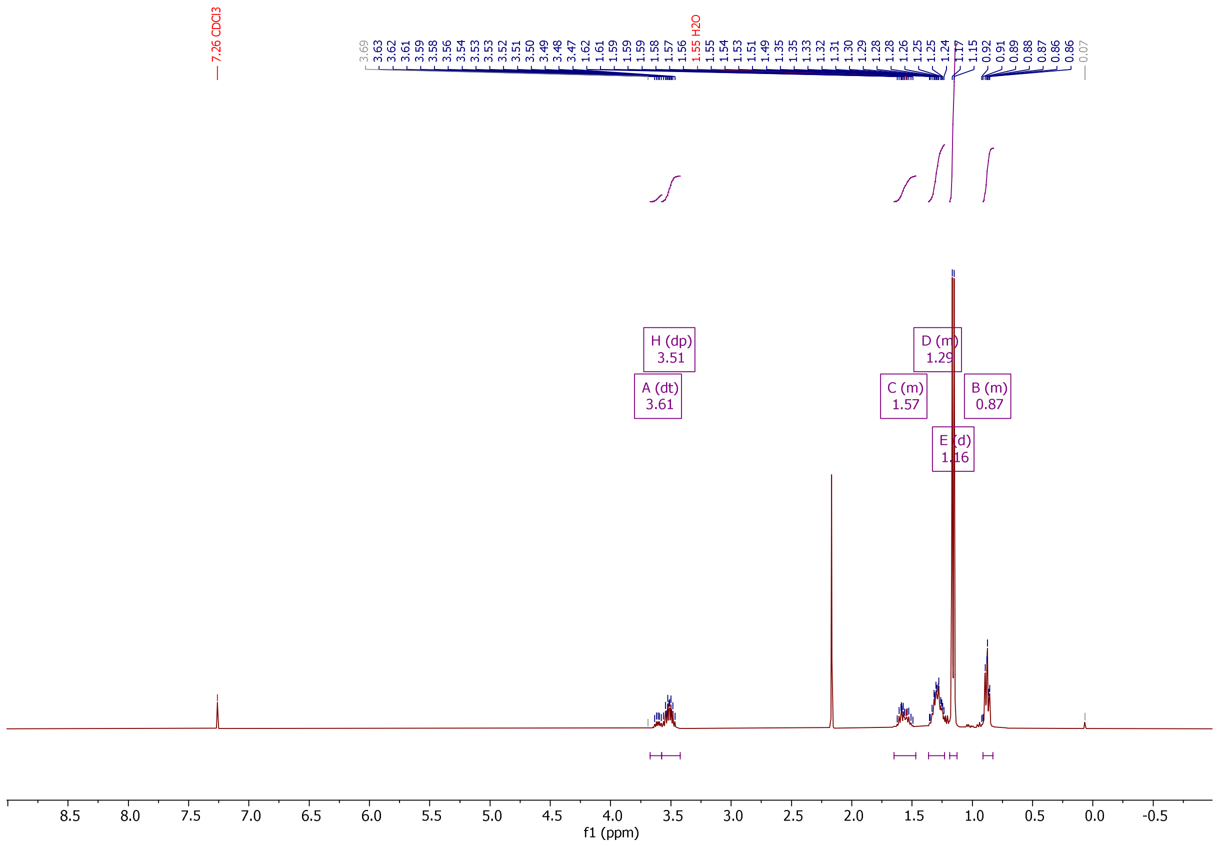

Supplementary Figure S165: ^{1}H NMR (400 MHz, $CDCl_3$) spectrum of **crude 9.**

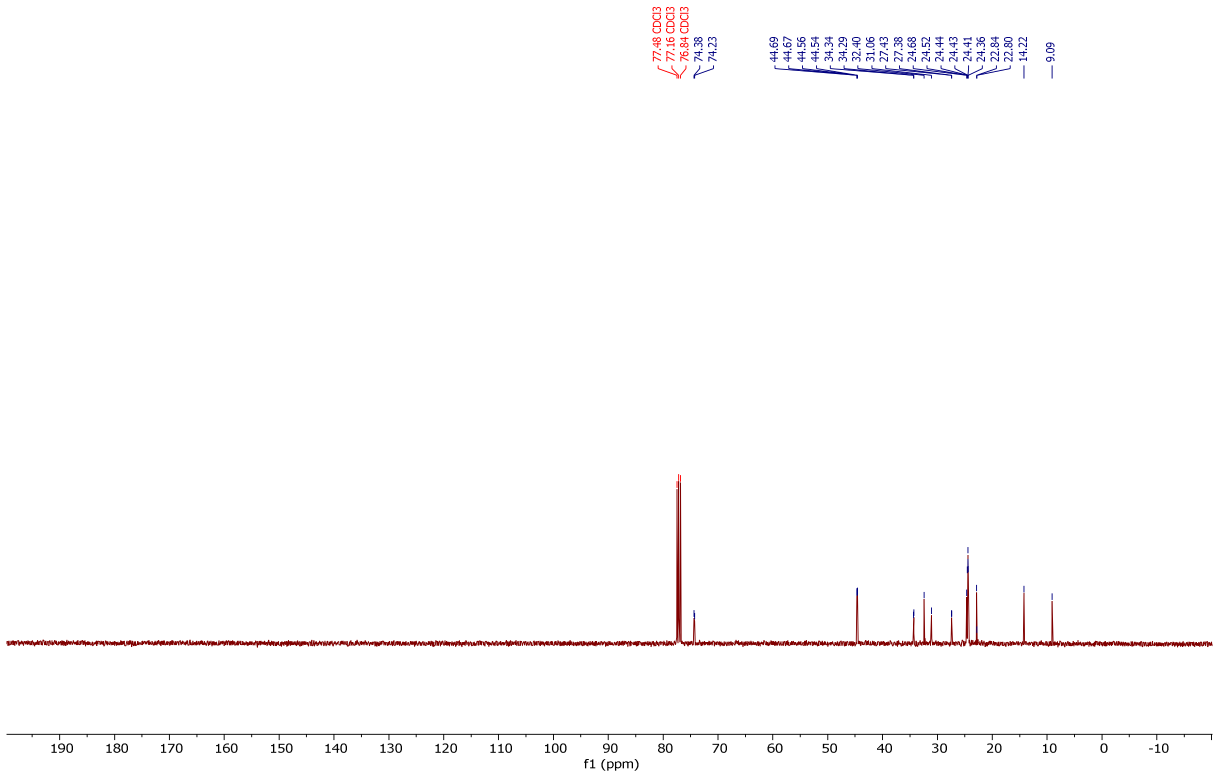

Supplementary Figure S166: ^{13}C NMR (101 MHz, $CDCl_3$) spectrum of **crude 9.**

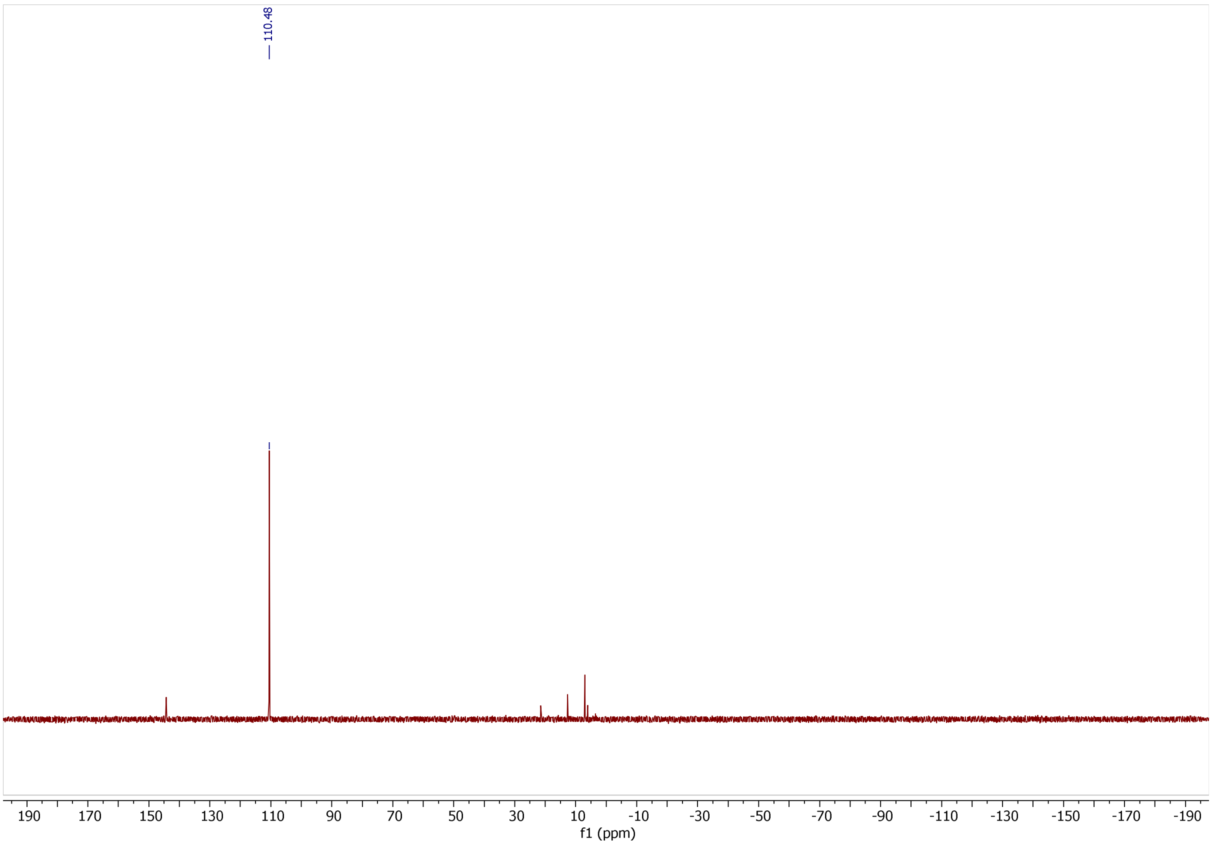

Supplementary Figure S167: ^{31}P NMR (162 MHz, $CDCl_3$) spectrum of **crude 9.**

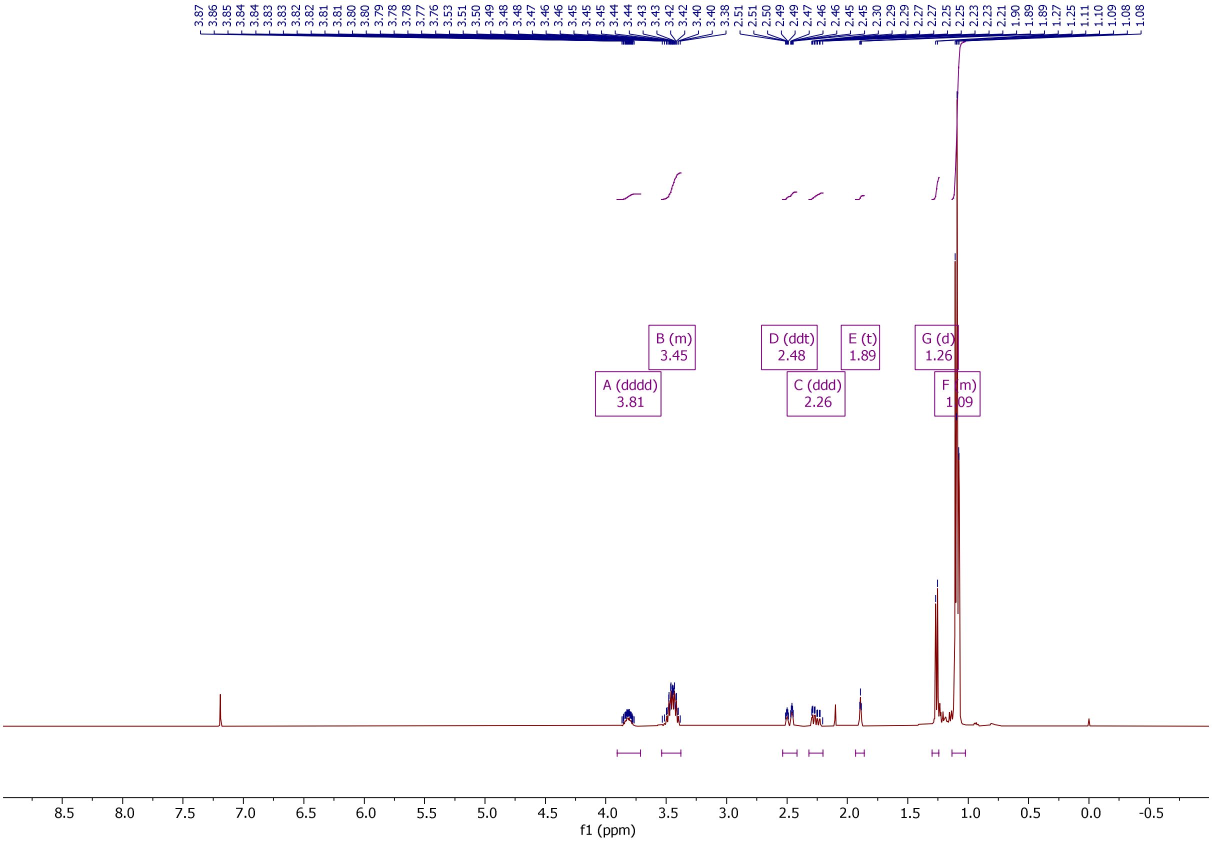

Supplementary Figure S168: ^{1}H NMR (400 MHz, $CDCl_3$) spectrum of **crude 10.**

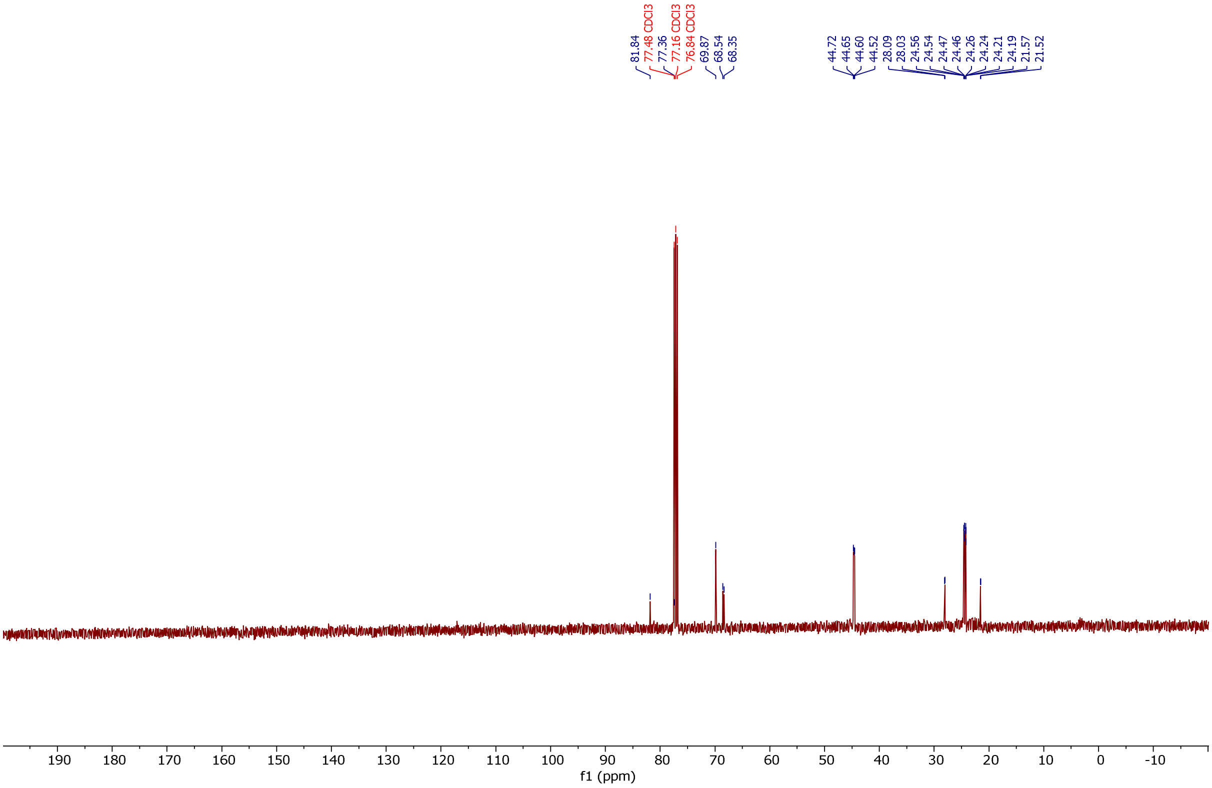

Supplementary Figure S169: ^{13}C NMR (101 MHz, $CDCl_3$) spectrum of **crude 10.**

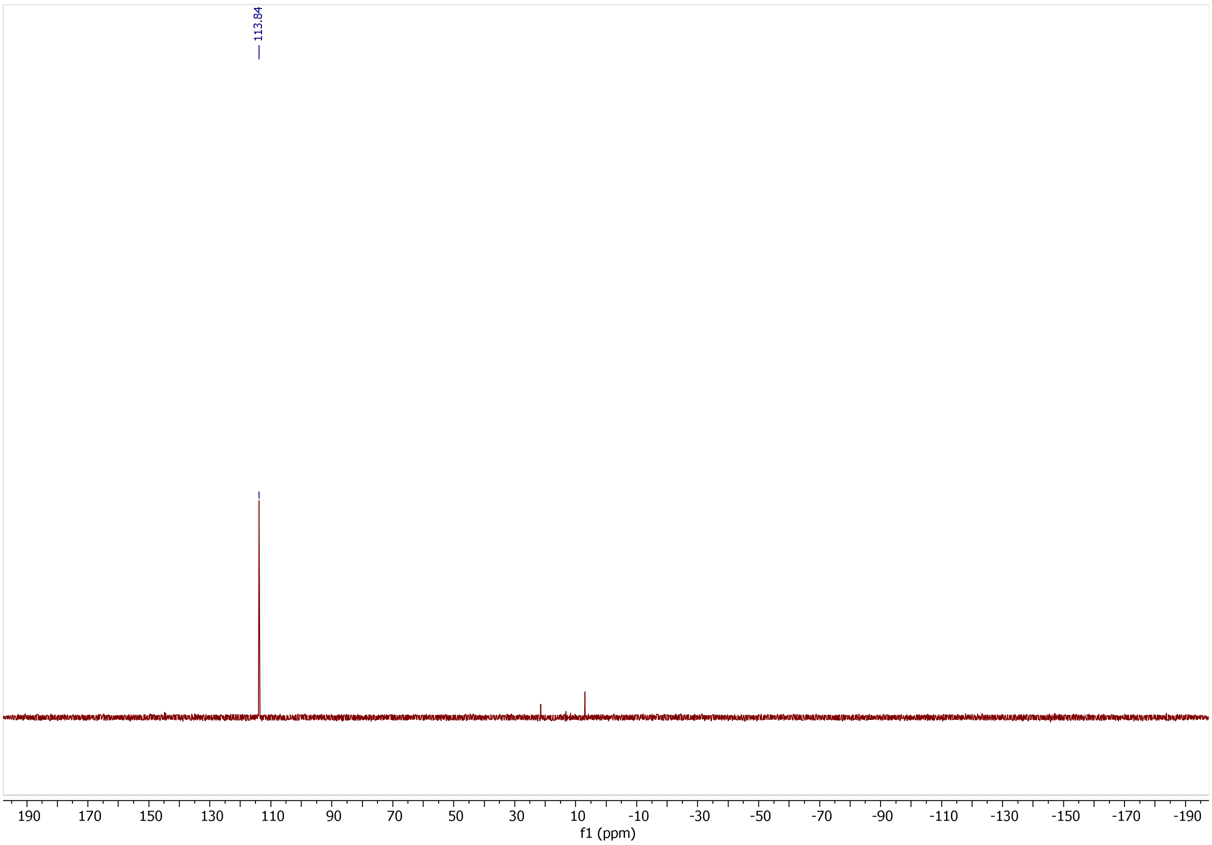

Supplementary Figure S170: ^{31}P NMR (162 MHz, $CDCl_3$) spectrum of **crude 10.**

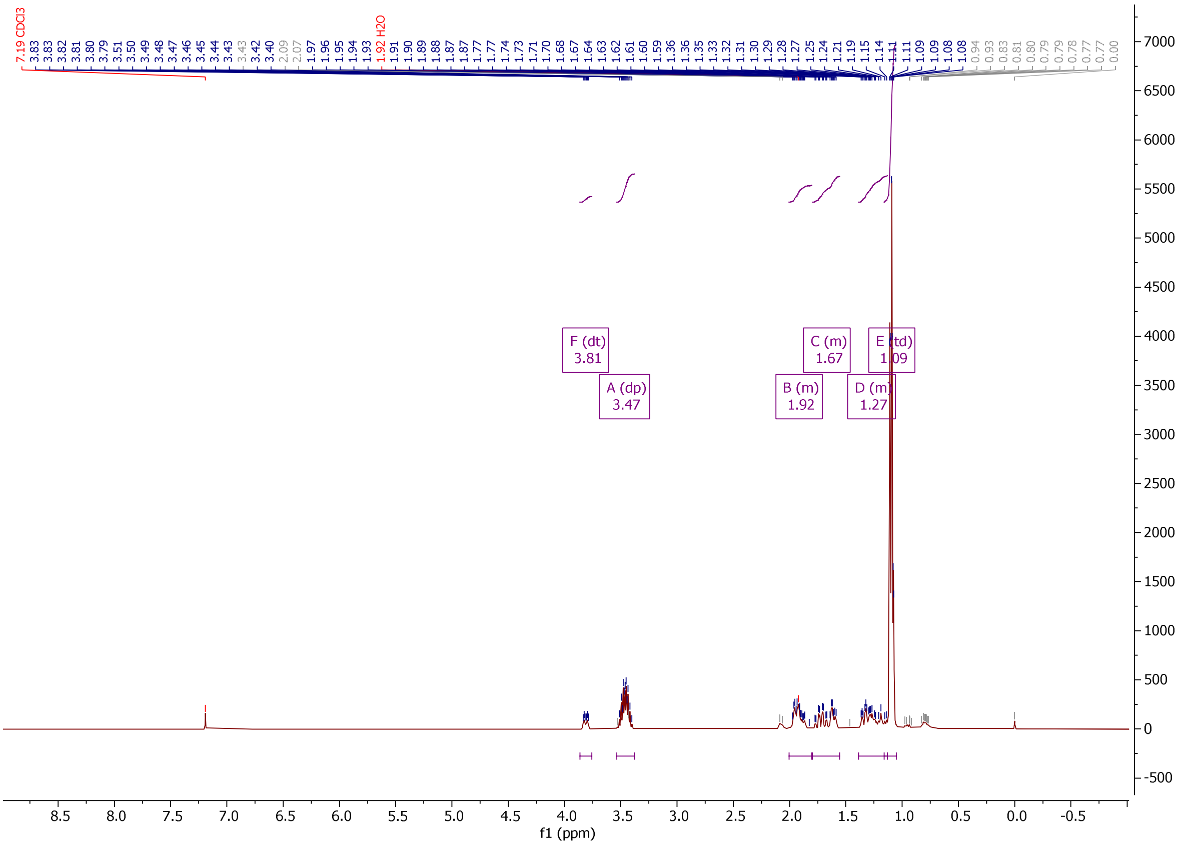

Supplementary Figure S171: ^{1}H NMR (400 MHz, $CDCl_3$) spectrum of **crude 11.**

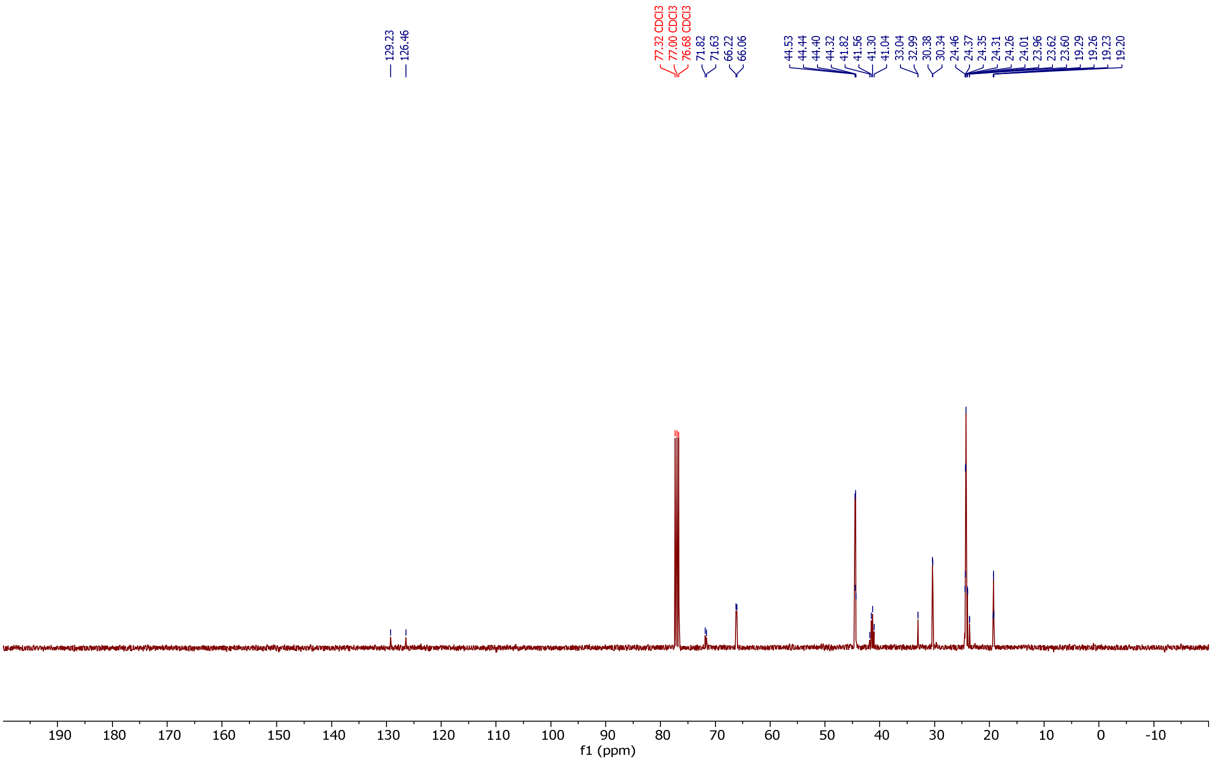

Supplementary Figure S172: ^{13}C NMR (101 MHz, $CDCl_3$) spectrum of **crude 11.**

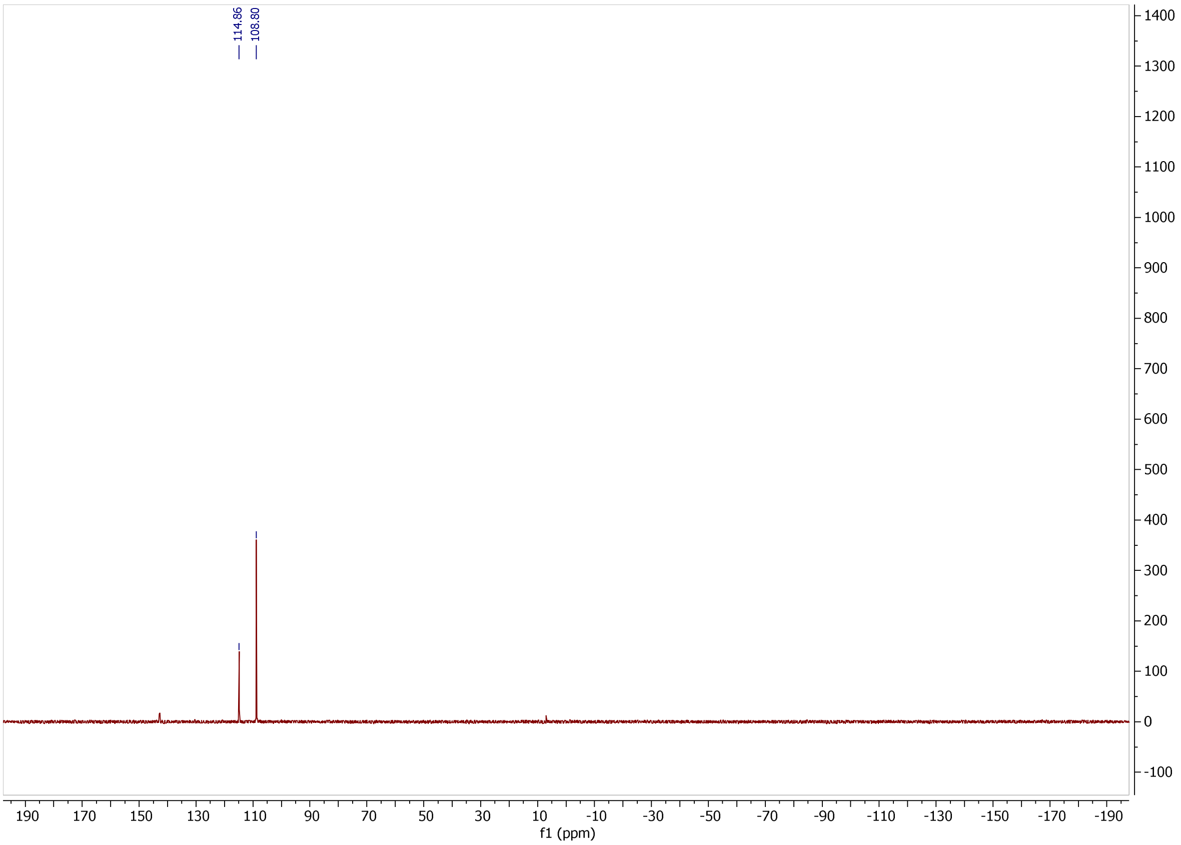

Supplementary Figure S173: ^{31}P NMR (162 MHz, $CDCl_3$) spectrum of **crude 11.**

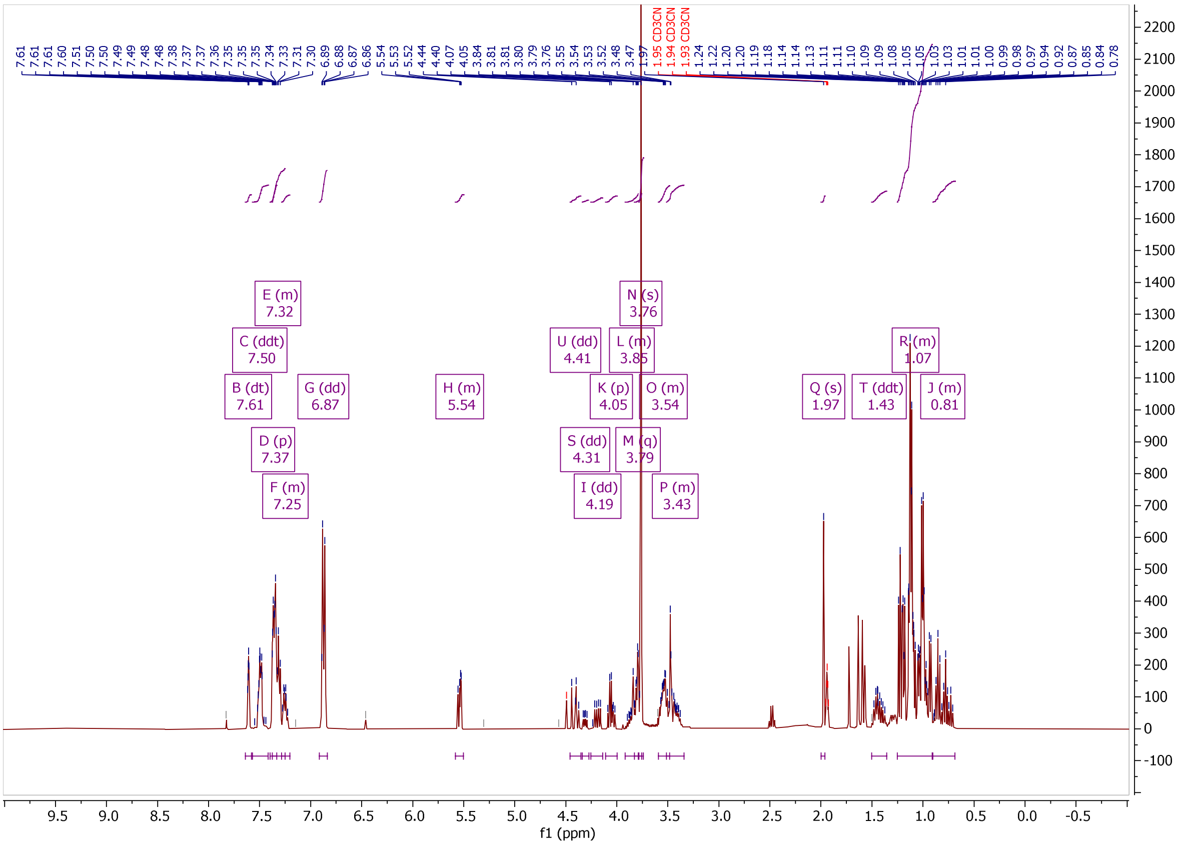

Supplementary Figure S174: ^{1}H NMR (400 MHz, CD_3CN) spectrum of **13.**

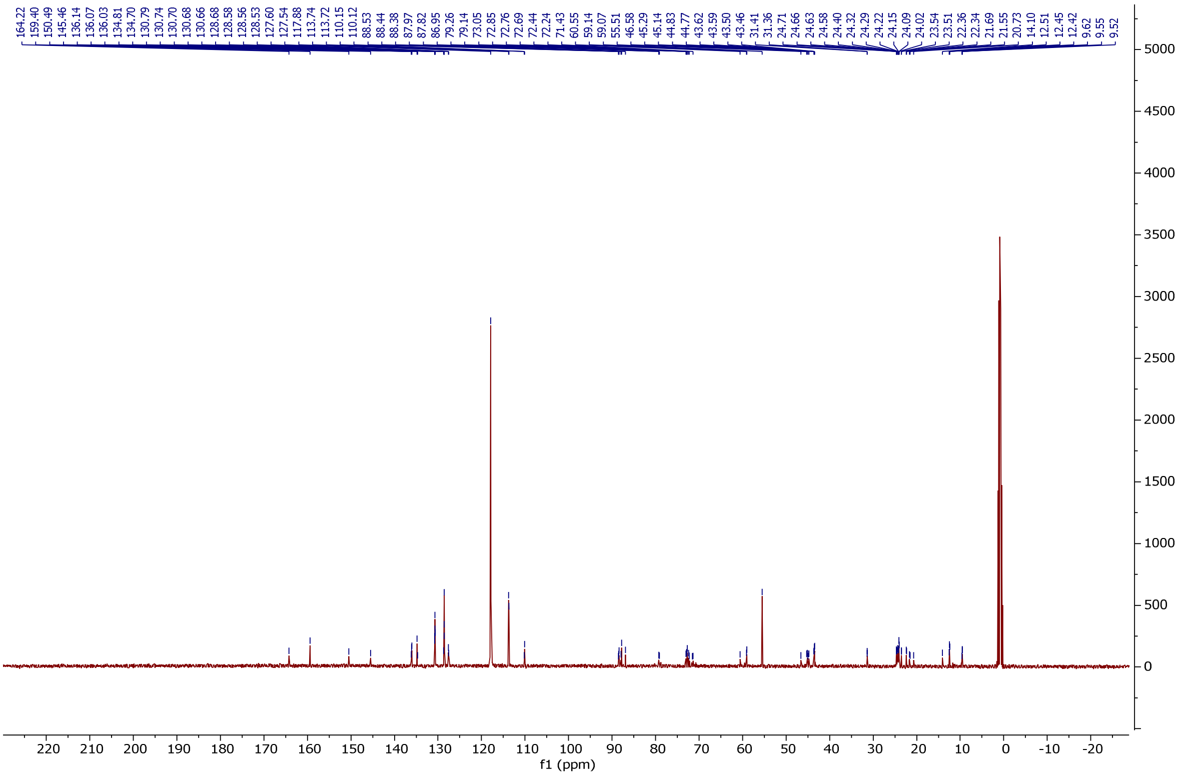

Supplementary Figure S175: ^{13}C NMR (101 MHz, CD_3CN) spectrum of **13.**

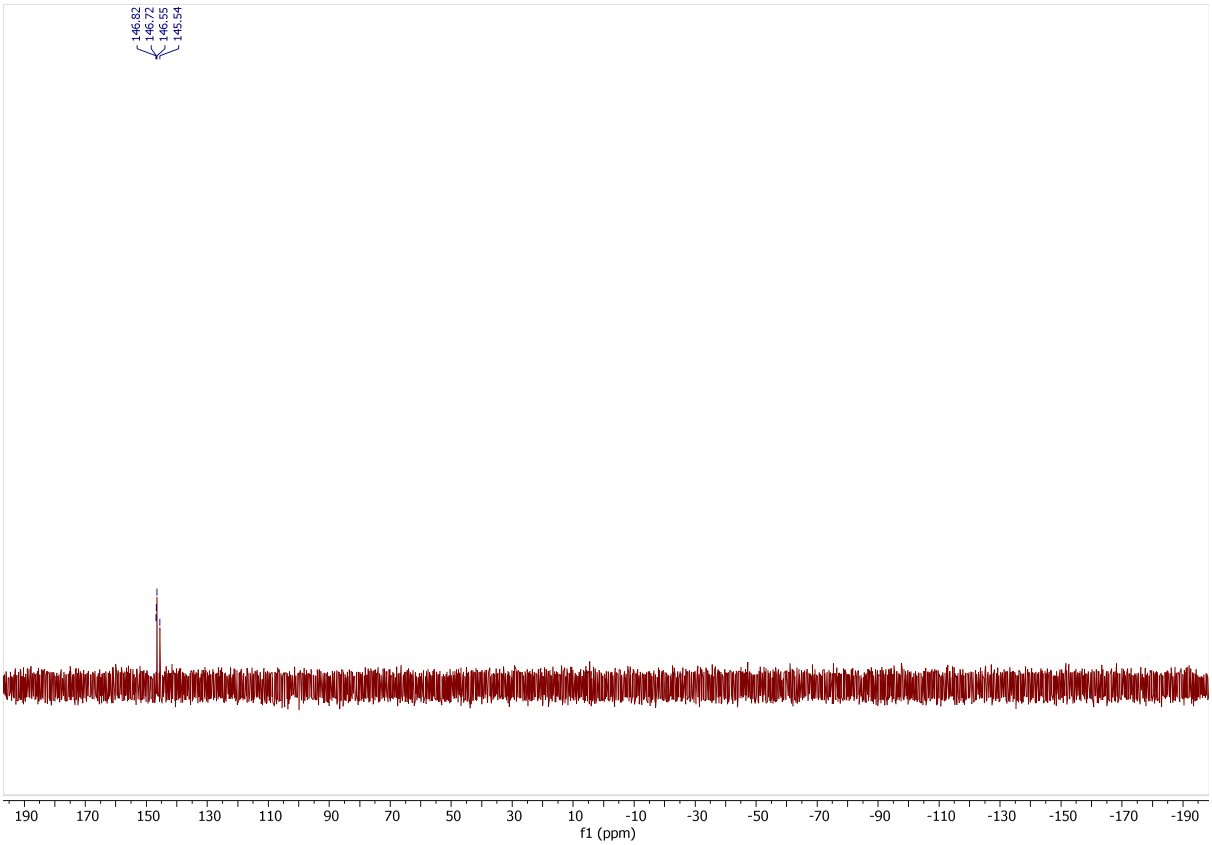

Supplementary Figure S176: ^{31}P NMR (162 MHz, $CDCl_3$) spectrum of **13.**

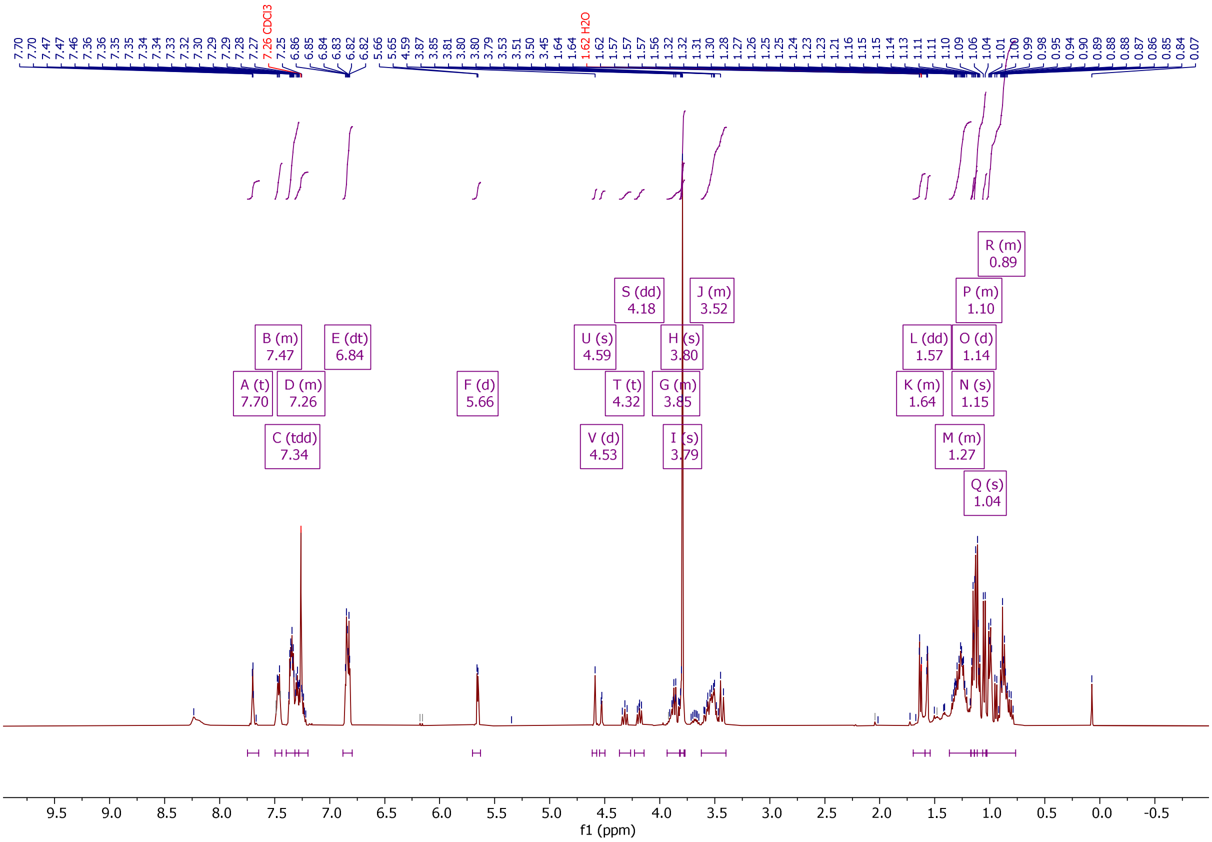

Supplementary Figure S177: ^{1}H NMR (400 MHz, $CDCl_3$) spectrum of **14.**

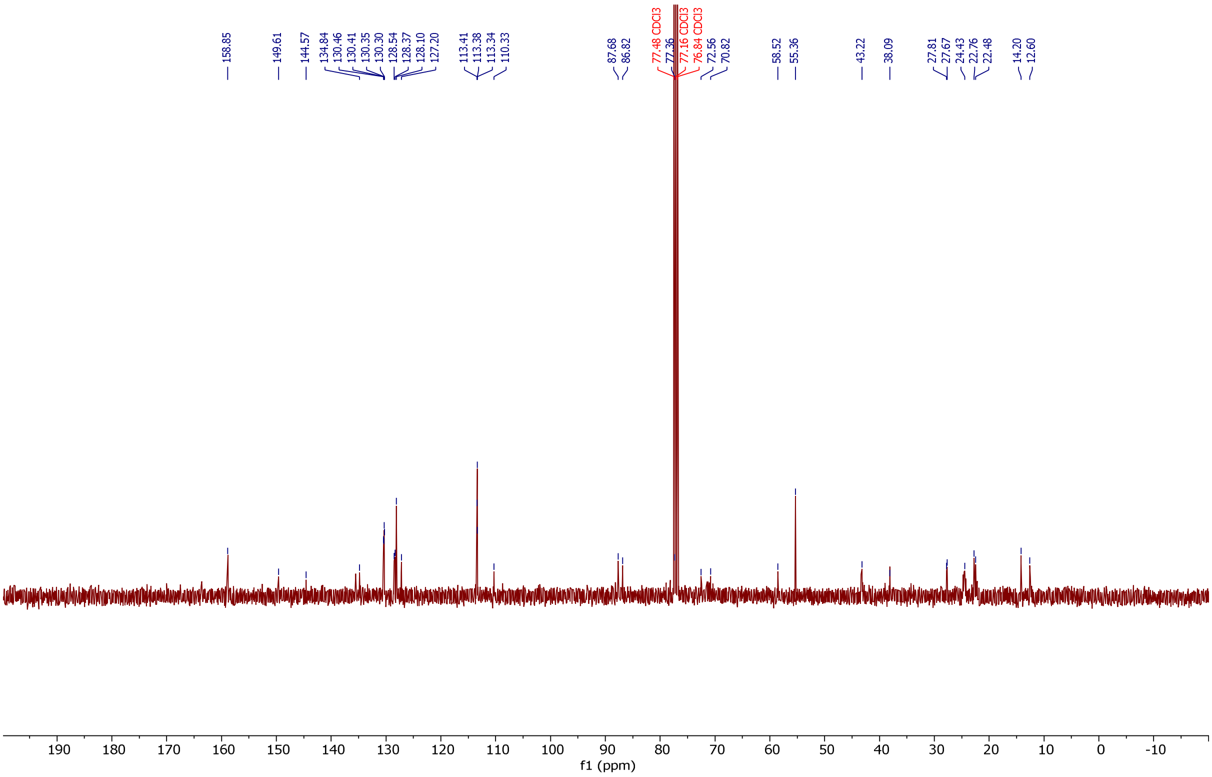

Supplementary Figure S178: ^{13}C NMR (101 MHz, $CDCl_3$) spectrum of **14.**

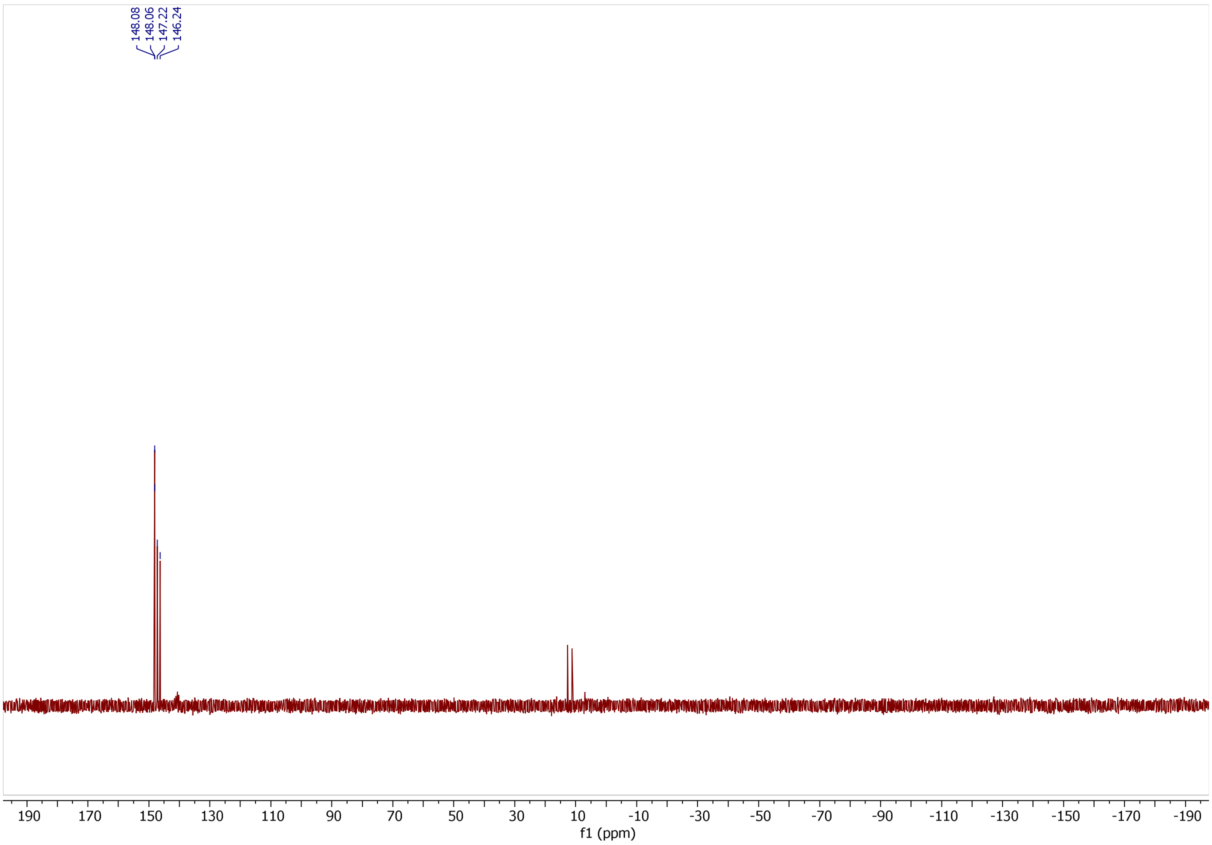

Supplementary Figure S179: ^{31}P NMR (162 MHz, $CDCl_3$) spectrum of **14.**

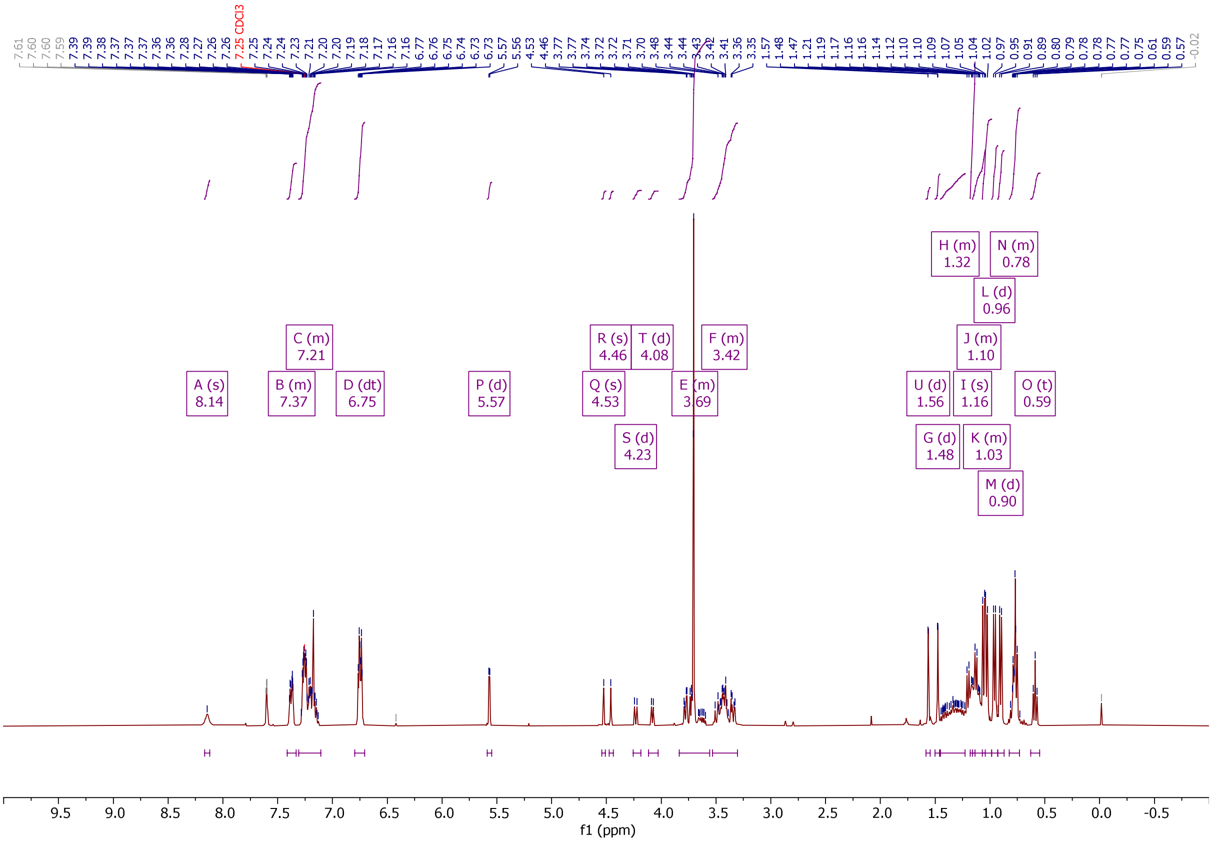

Supplementary Figure S180: ^{1}H NMR (400 MHz, $CDCl_3$) spectrum of **15.**

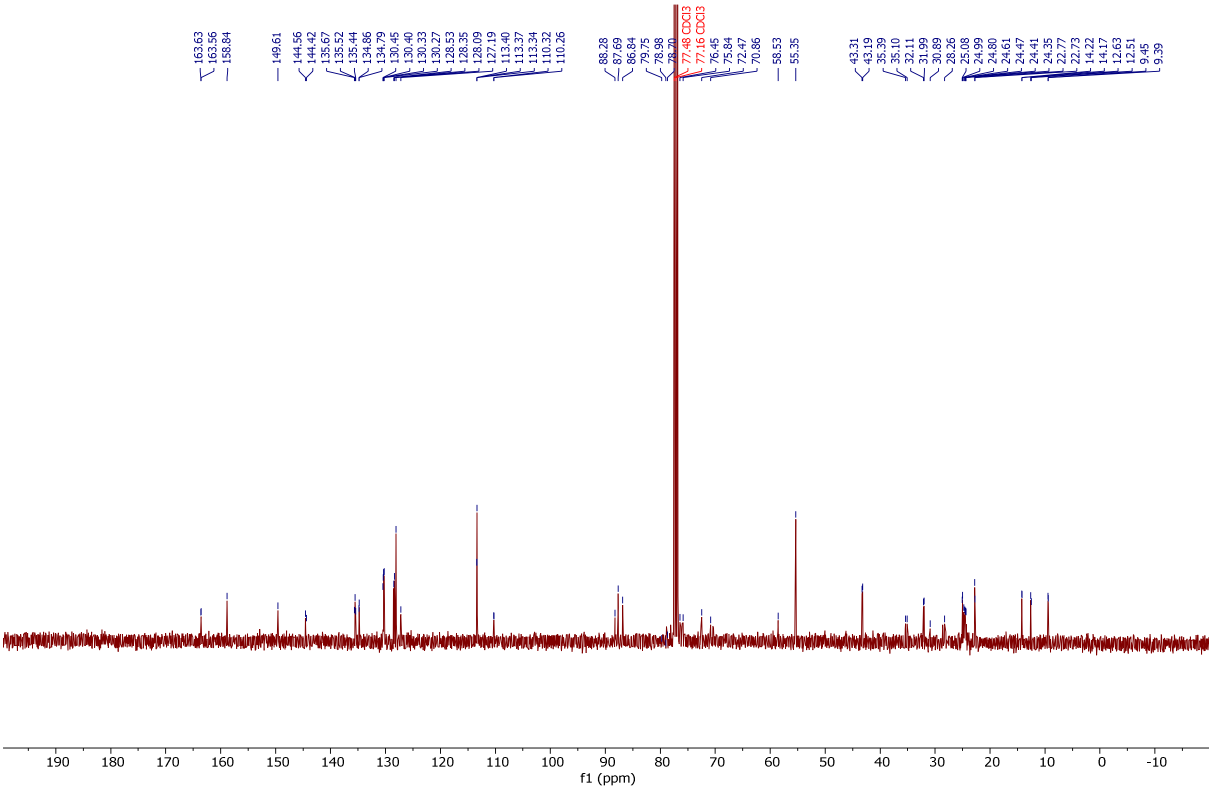

Supplementary Figure S181: ^{13}C NMR (101 MHz, $CDCl_3$) spectrum of **15.**

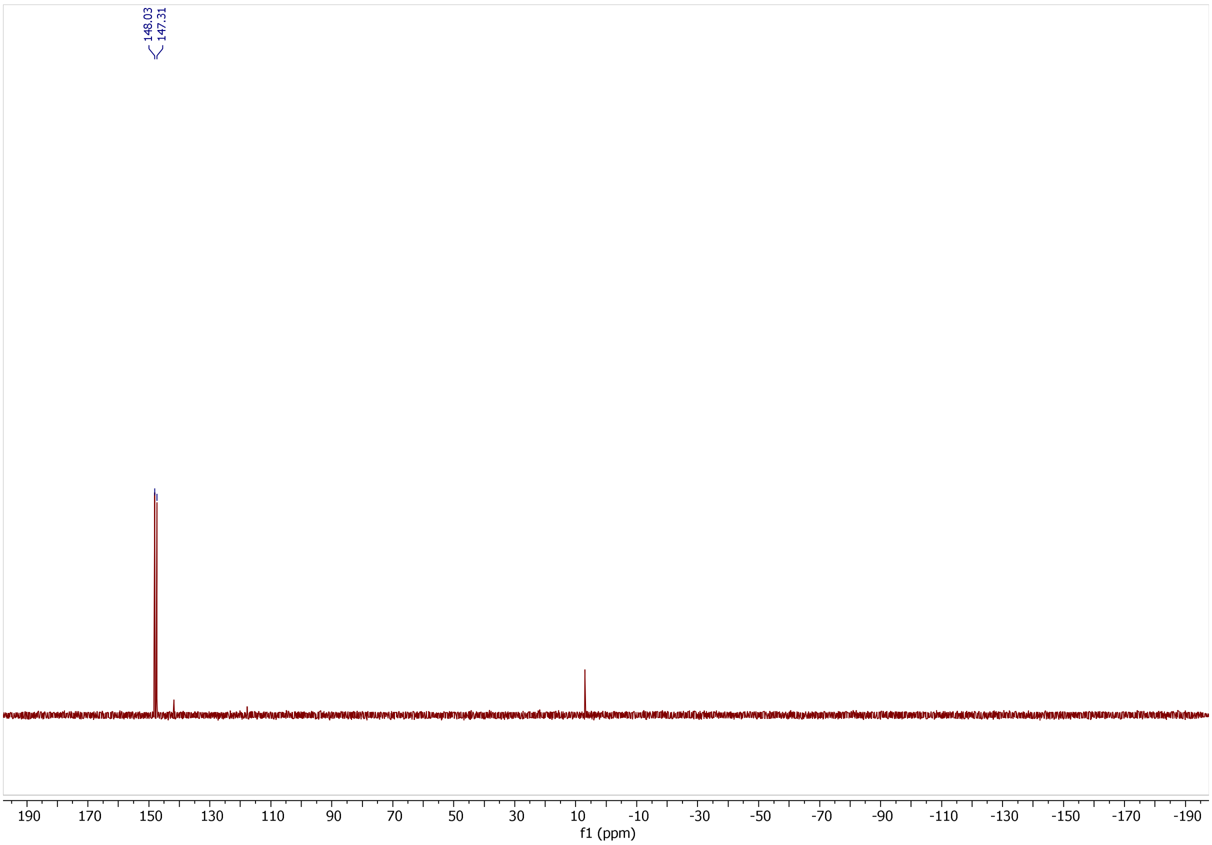

Supplementary Figure S182: ^{31}P NMR (162 MHz, $CDCl_3$) spectrum of **15.**

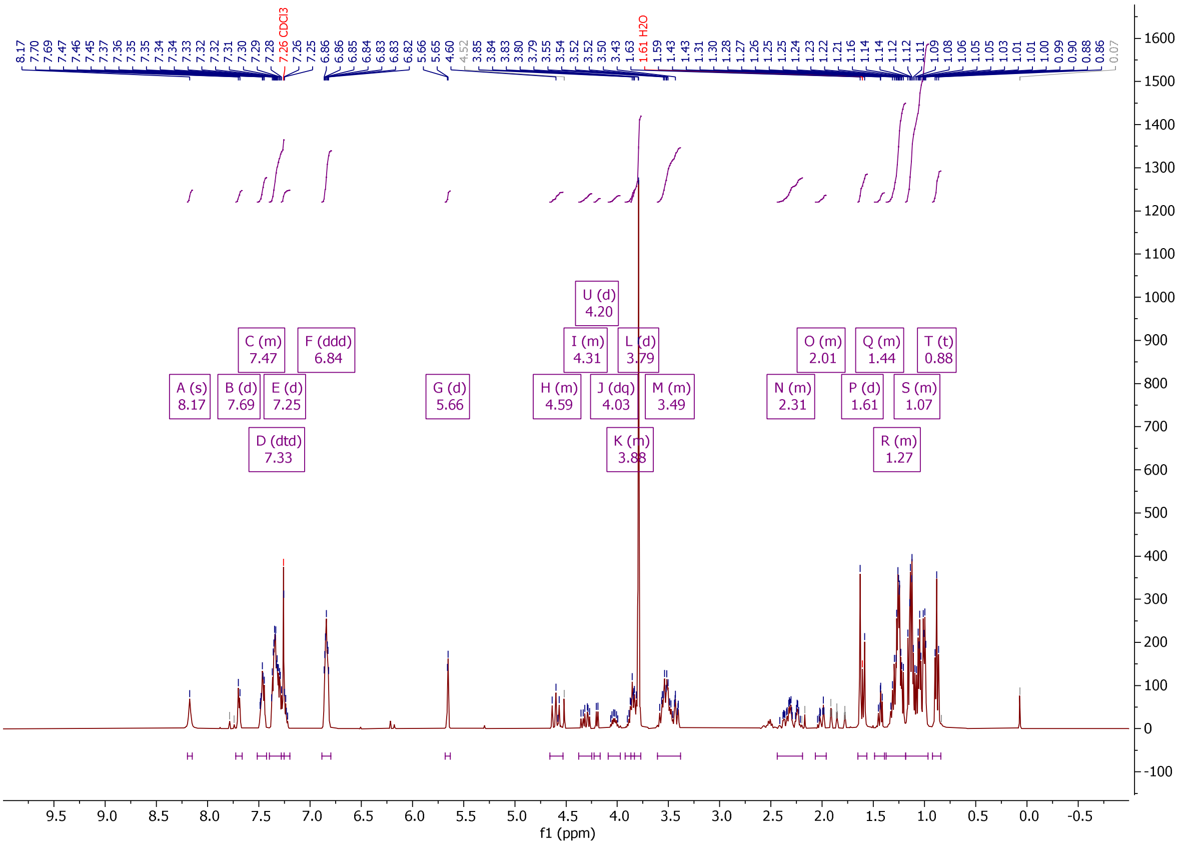

Supplementary Figure S183: ^{1}H NMR (400 MHz, $CDCl_3$) spectrum of **15.**

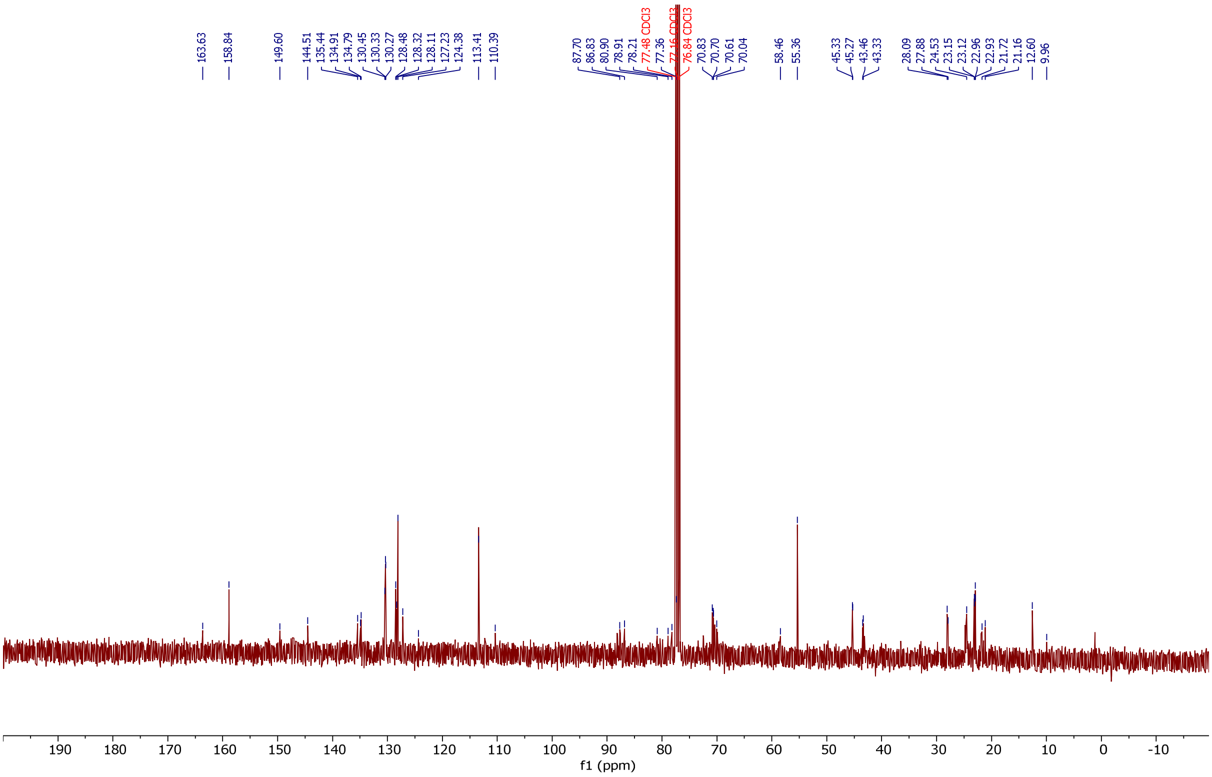

Supplementary Figure S184: ^{13}C NMR (101 MHz, $CDCl_3$) spectrum of **15.**

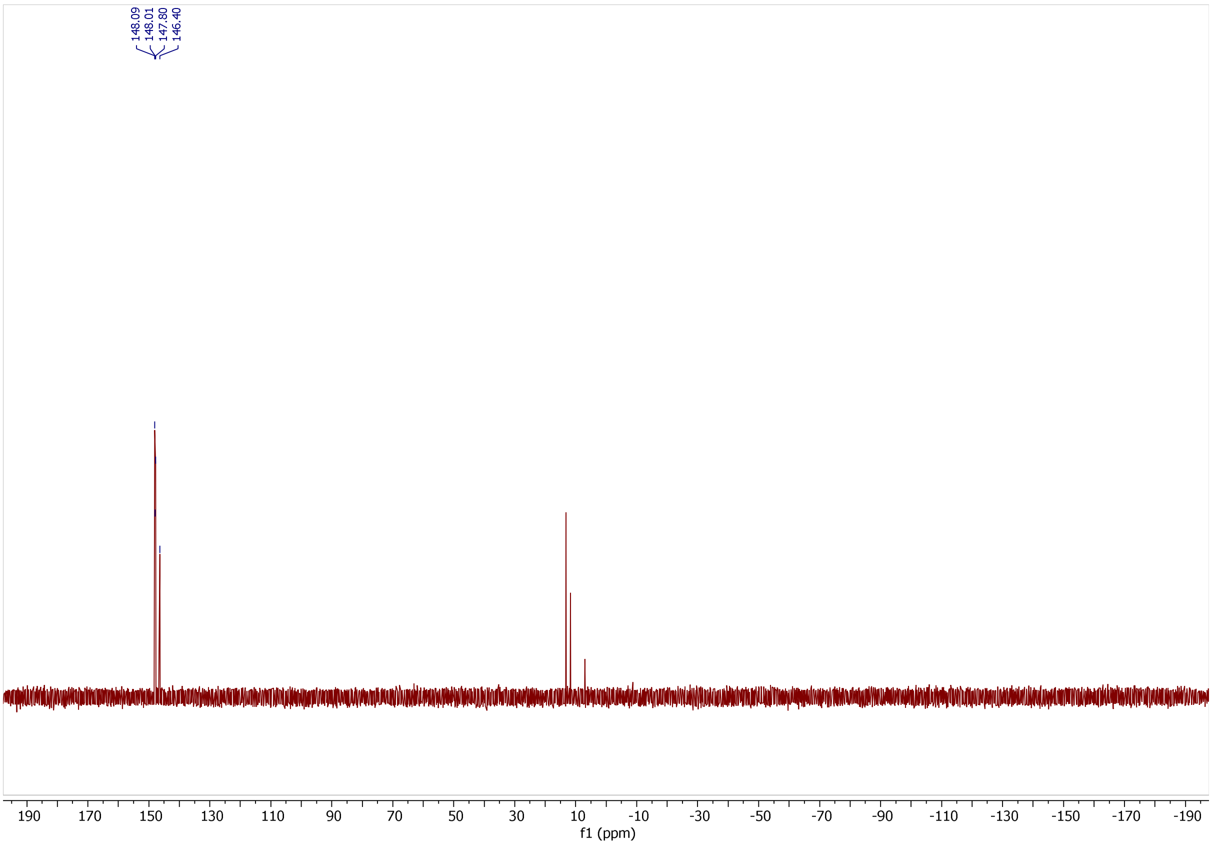

Supplementary Figure S185: ^{31}P NMR (162 MHz, $CDCl_3$) spectrum of **16.**

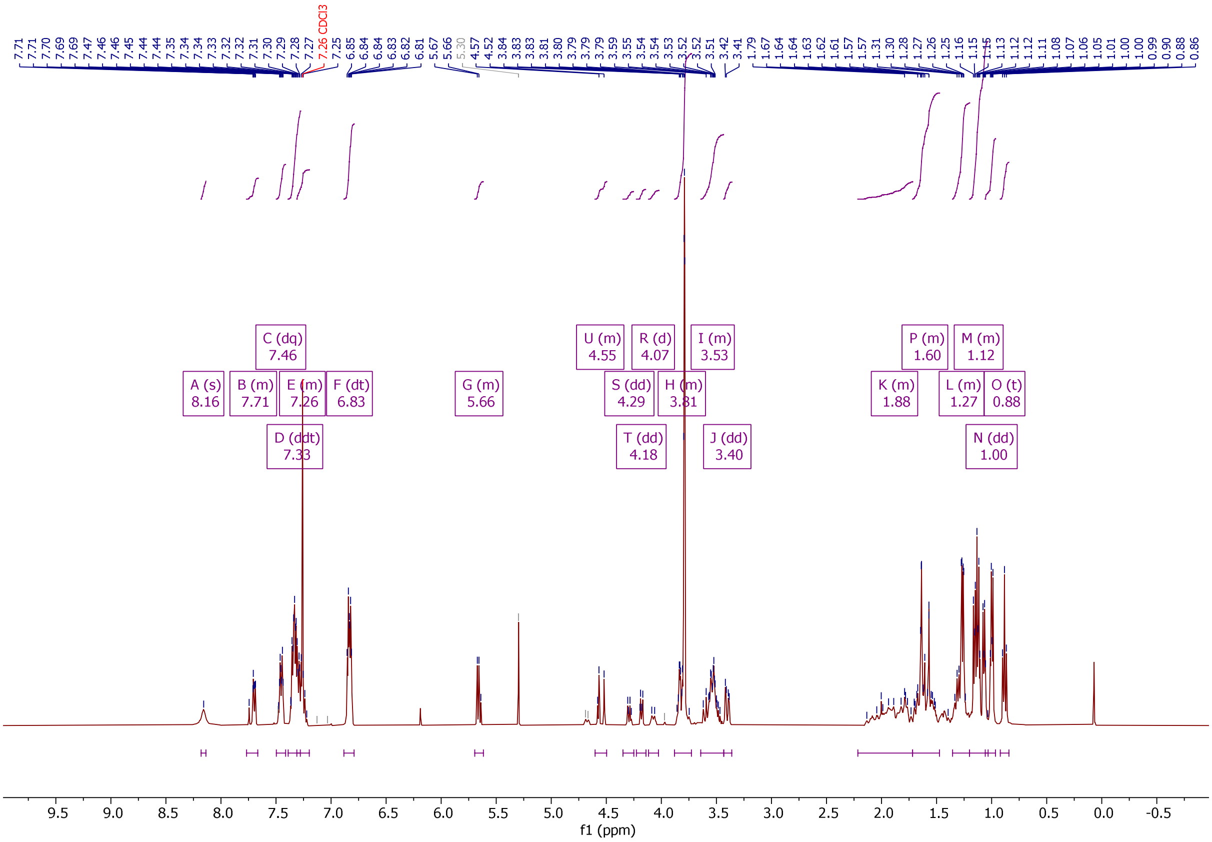

Supplementary Figure S186: ^{1}H NMR (400 MHz, $CDCl_3$) spectrum of **17.**

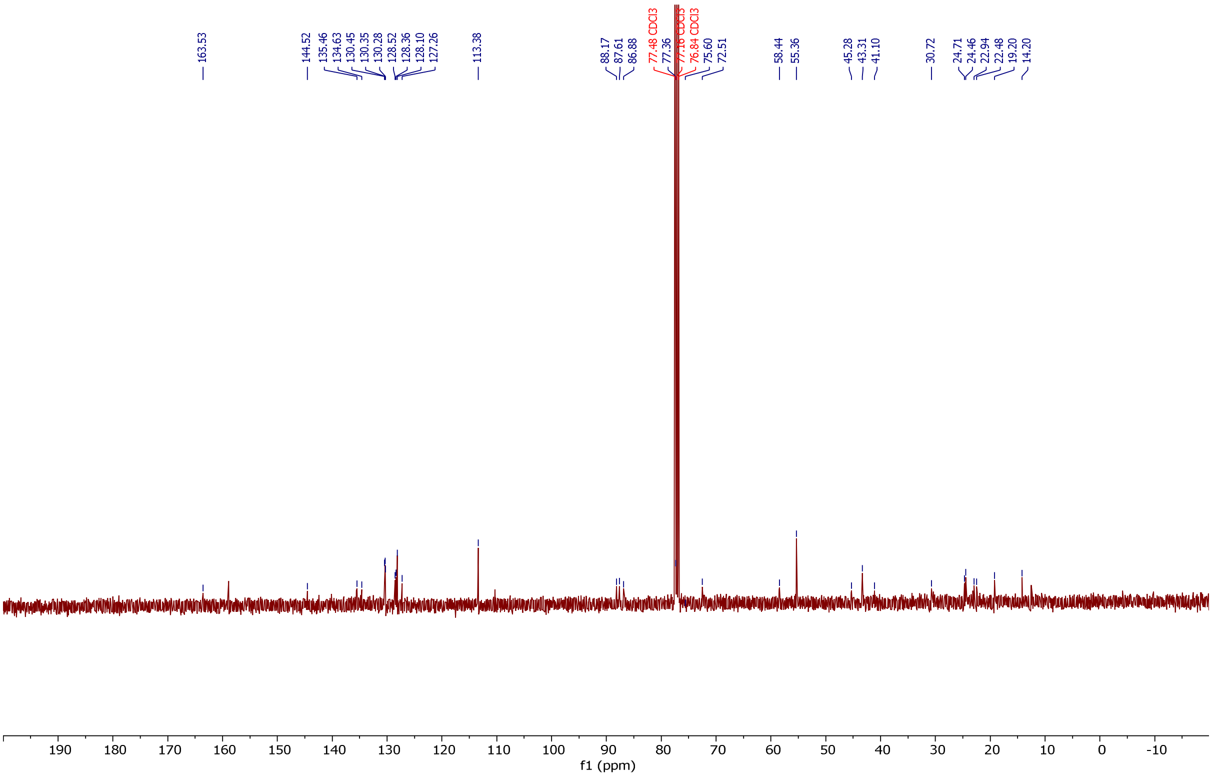

Supplementary Figure S187: ^{13}C NMR (101 MHz, $CDCl_3$) spectrum of **17.**

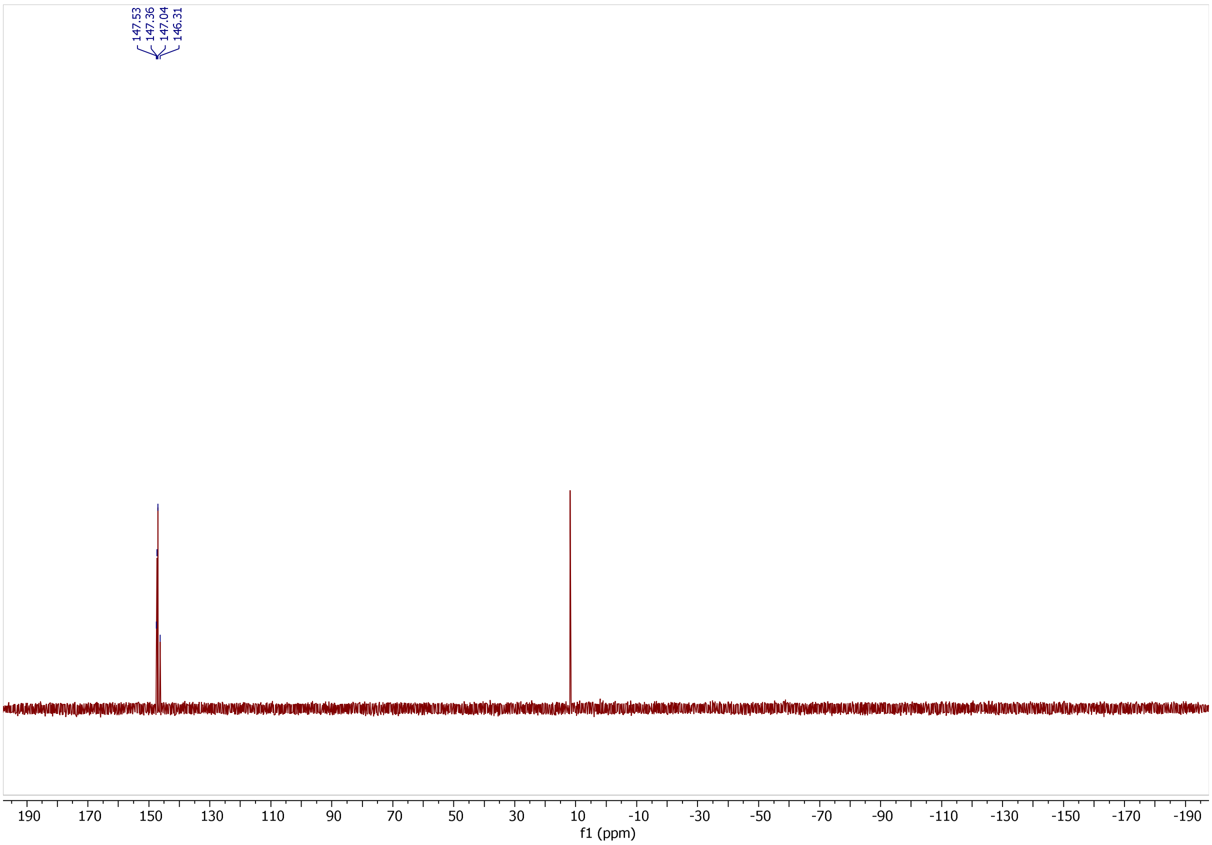

Supplementary Figure S188: ^{31}P NMR (162 MHz, $CDCl_3$) spectrum of **17.**

8.0 References

1. Dhara, D., Hill, A.C., Ramesh, A., Wood, M.J.A., El-Sagheer, A.H. and Brown, T. (2024) Synthesis, Biophysical and Biological Evaluation of Splice-Switching Oligonucleotides with Multiple LNA-Phosphothiotriester Backbones. *J. Am. Chem. Soc.*, **146**, 29773-29781.
2. Kang, S.-H., Cho, M.-J. and Kole, R. (1998) Up-Regulation of Luciferase Gene Expression with Antisense Oligonucleotides: Implications and Applications in Functional Assay Development. *Biochemistry*, **37**, 6235-6239.
